# Grouping of UVCB Substances with New Approach Methodologies (NAMs) Data

**DOI:** 10.14573/altex.2006262

**Published:** 2020-10-09

**Authors:** John S. House, Fabian A. Grimm, William D. Klaren, Abigail Dalzell, Srikeerthana Kuchi, Shu-Dong Zhang, Klaus Lenz, Peter J. Boogaard, Hans B. Ketelslegers, Timothy W. Gant, Fred A. Wright, Ivan Rusyn

**Affiliations:** 1 Bioinformatics Research Center, North Carolina State University, Raleigh, NC, USA; 2 Department of Veterinary Integrative Biosciences, Texas A&M University, College Station, TX, USA; 3 Public Health England, Centre for Radiation, Chemical and Environmental Hazards, Harwell Science Campus, Oxon, UK; 4 Northern Ireland Centre for Stratified Medicine, Ulster University, L/Derry, Northern Ireland, UK; 5 SYNCOM Forschungs- und Entwicklungsberatung GmbH, Ganderkesee, Germany; 6 SHELL International BV, The Hague, The Netherlands; 7 Concawe, Brussels, Belgium; 8 current address: Biostatistics & Computational Biology Branch, National Institute of Environmental Health Sciences, RTP, NC, USA; 9 current address: ExxonMobil Biomedical Sciences Inc., Annandale, NJ, USA; 10 current address: S.C. Johnson and Son, Inc., Racine, WI, USA; 11 current address: MRC-University of Glasgow Centre for Virus Research, Glasgow, Scotland, UK

## Abstract

One of the most challenging areas in regulatory science is assessment of the substances known as UVCB (unknown or variable composition, complex reaction products and biological materials). Because the inherent complexity and variability of UVCBs present considerable challenges for establishing sufficient substance similarity based on chemical characteristics or other data, we hypothesized that new approach methodologies (NAMs), including *in vitro* test-derived biological activity signatures to characterize substance similarity, could be used to support grouping of UVCBs. We tested 141 petroleum substances as representative UVCBs in a compendium of 15 human cell types representing a variety of tissues. Petroleum substances were assayed in dilution series to derive point of departure estimates for each cell type and phenotype. Extensive quality control measures were taken to ensure that only high-confidence *in vitro* data were used to determine whether current groupings of these petroleum substances, based largely on the manufacturing process and physico-chemical properties, are justifiable. We found that bioactivity data-based groupings of petroleum substances were generally consistent with the manufacturing class-based categories. We also showed that these data, especially bioactivity from human induced pluripotent stem cell (iPSC)-derived and primary cells, can be used to rank substances in a manner highly concordant with their expected *in vivo* hazard potential based on their chemical compositional profile. Overall, this study demonstrates that NAMs can be used to inform groupings of UVCBs, to assist in identification of representative substances in each group for testing when needed, and to fill data gaps by read-across.

## 1 Introduction

Substance identification is required before exposure, hazard or risk evaluations are performed by industry or regulatory authorities. Most substances that are evaluated with respect to human or ecological risks are of the “mono-constituent” type, i.e., they contain one main constituent in at least 80% (w/w), even after accounting for impurities (ECHA, 2015). Other types are deemed to be “multi-constituent” substances and UVCBs (unknown or variable composition, complex reaction products, and biological materials). The latter comprise about 20% of all recent substance registrations in the European Union (ECHA, 2017) under the Regulation on the Registration, Evaluation and Authorisation of Chemicals (REACH). UVCBs are challenging for regulatory decision-making because of few established frameworks for their evaluation under current chemical regulatory regimes (ECHA, 2017).

Petroleum substances are prototypical UVCBs (Clark et al., 2013), and their complex and variable nature is the consequence of their manufacturing processes. They are primarily produced by the distillation of petroleum feed stocks, typically followed by additional processing steps such as solvent extraction, hydro-desulfurization, or hydrogenation (McKee et al., 2015). As a result, these are complex substances containing a large number of individual hydrocarbon molecules that can be aliphatic/paraffinic (straight chain or branched), alicyclic/naphthenic (containing primarily cyclo-paraffinic constituents that are primarily saturated hydrocarbons), or aromatic. Petroleum substances of different types can vary significantly in their chemical complexity and diversity based on their degree of refinement and may contain any or all of these types of constituents in varying concentrations based on their respective manufacturing process. While petroleum substances of particular types and end-uses may have compositional differences, their compositional variation is limited to specified ranges based on the technical specifications of each product. The challenge of grouping petroleum substances is further complicated by the reality that the currently used substance nomenclature, due to the inherent chemical complexity of UVCBs, is not uniquely associated with chemical composition but relies on the manufacturing process, associated physico-chemical characteristics, and product performance specifications.

To identify the hazards of petroleum substances, toxicology testing is conducted on the whole substance (*in vivo*) or on exposure-relevant fractions of petroleum substances (*in vitro*), rather than on individual constituents or groups of constituents. Where no data are available for certain endpoints on a substance, alternative methods to fill data gaps in the registration requirements have been suggested, including the application of read-across (ECHA, 2015). Grouping of substances that are compositionally similar is one path to reduce animal testing, provided there is sufficient information on the related compounds and sufficient reason to believe that the related compounds may have similar toxicological properties. However, existing grouping approaches for petroleum substances, which are currently based on the manufacturing process, physico-chemical characteristics (CONCAWE, 2017), and hazard data (McKee et al., 2015), have been challenged by regulatory agencies (Ball et al., 2014).

Complementary approaches to grouping of petroleum-based UVCBs have been proposed based on *in vitro* testing (Grimm et al., 2016; Kamelia et al., 2019), structure-activity analysis (Kutsarova et al., 2019; Dimitrov et al., 2015), or novel analytical chemistry methods (Grimm et al., 2017; Onel et al., 2019). Similar approaches have been applied to other categories of UVCBs (Catlin et al., 2018). Because of the strong impetus to avoid unnecessary vertebrate animal testing, a number of recent changes to the regulatory requirements in the US and EU provide for *in vitro* testing or quantitative structure-activity relationship analysis as alternatives to whole-animal toxicity testing (Herrmann et al., 2019; Malloy et al., 2017; Kavlock et al., 2018).

Therefore, this study tested the hypothesis that the challenge of demonstrating substance similarity to support grouping of petroleum substances can be improved using data derived from new approach methodologies (NAMs) (i.e., *in vitro* bioactivity) linked to the available compositional data (physical and analytical chemistry data). We tested the effects of 141 petroleum substances in 15 human cell types to derive a comprehensive set of phenotypes that were used to group substances based on their *in vitro* bioactivity. We found that NAM data can be used to rank individual UVCBs in a manner that was highly concordant with their expected *in vivo* hazard potential based on their chemical composition. Bioactivity data-based groupings of petroleum substances were largely consistent with the manufacturing class-based categories. Furthermore, NAM data can be used to identify representative substances of each category for further testing and subsequent read-across.

## 2 Materials and methods

### Chemicals

**Fig. 1:**
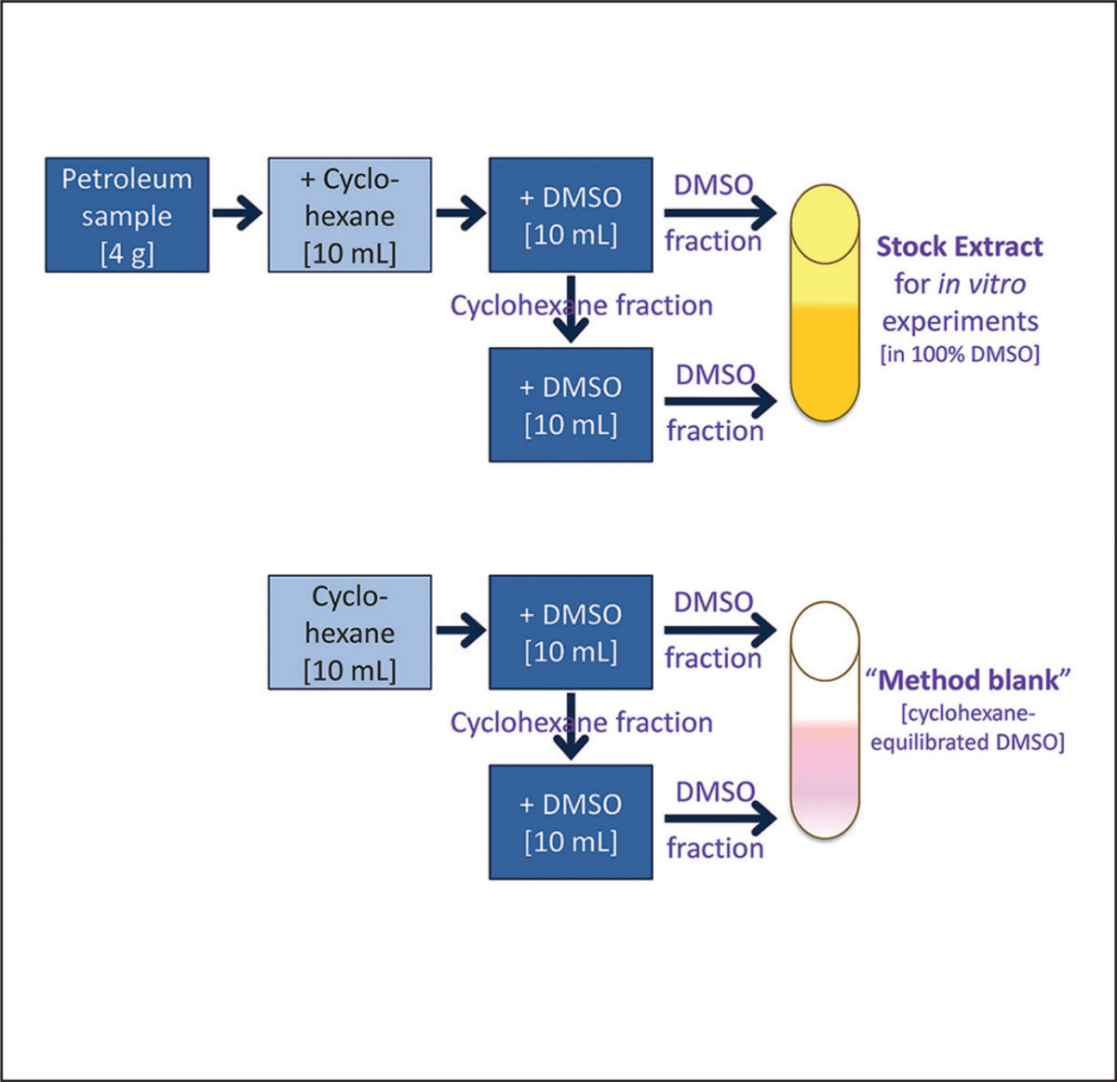
General schematic diagram of the extraction procedure that was used in these studies The procedure was based on ASTM International (2014) standard method (E1687-10).

All chemicals used in these studies, except for petroleum substances, were obtained from Sigma-Aldrich (St. Louis, MO) unless otherwise noted. Samples of petroleum substances were supplied by Concawe (Brussels, Belgium). To enable *in vitro* bioactivity profiling experiments of petroleum substances, extraction of petroleum substances into DMSO was performed using American Society for Testing and Materials standard procedure (ASTM International, 2014) as outlined in Figure 1. The DMSO extraction procedure used herein was designed to concentrate the “biologically active” fraction (i.e., mostly 3–7 ring polycyclic aromatics, but also other polar constituents) of each petroleum substance; the extracts obtained using this method are used routinely for safety testing (e.g., mutagenicity) and chemical characterization of the refinery streams (ASTM International, 2014). Briefly, 4 grams of each petroleum substance (Tab. 1, Tab. S1^1^) was first dissolved in 10 mL of cyclohexane; 10 mL of DMSO (Fisher Scientific, Waltham, MA) was added, and the mixture was vigorously shaken for several minutes. The DMSO layer was removed using a glass pipette and the cyclohexane was re-extracted with an additional 10 mL of DMSO. Both polycyclic aromatic compound (PAC)-enriched DMSO layers were combined and diluted 2:1 with two volumes of 4% (w/v) sodium chloride solution. Following subsequent extraction with 20 mL and 10 mL cyclohexane to isolate the PAC fraction, the organic layers were washed twice with distilled water and filtered through anhydrous sodium sulfate. Details of additional chemicals that were used as references (Tab. S2^1^), cell type-specific positive controls or reagents (Fig. S1, Supplemental Files 1 and 2^1^) are provided as specified.

### In vitro *study design*

**Fig. 2:**
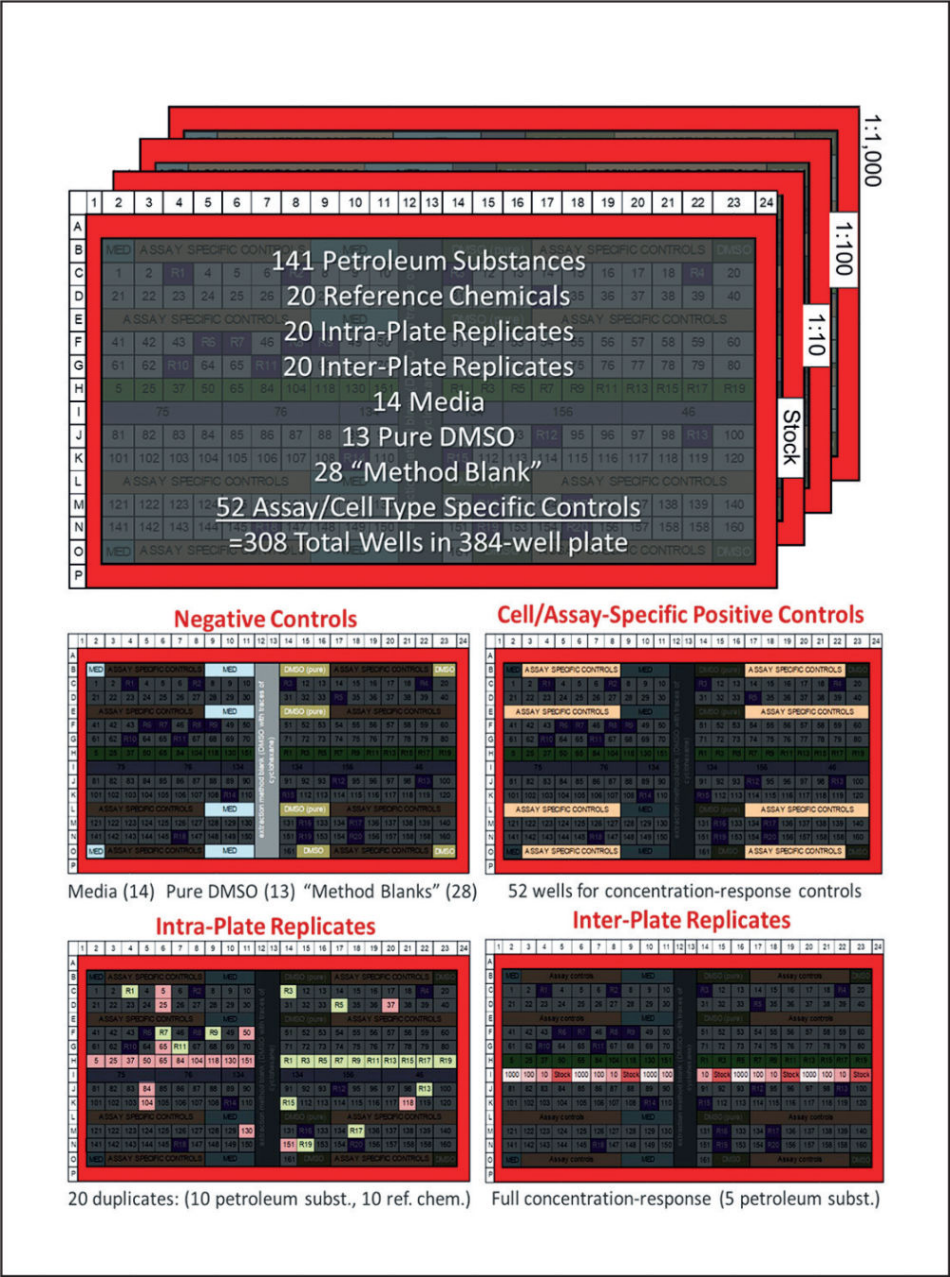
Experimental plate design Test substances (petroleum-based UVCBs) and other chemicals were arrayed on the individual plates for each dilution factor. Location of negative, positive and replication (intra- and inter-plate) controls on each plate are shown.

Petroleum substance extracts (Tab. S1^1^) and reference chemicals representing the major known structural classes of chemistries in petroleum substances (Tab. S2^1^) were processed to create a dilution series in DMSO. Overall, 4 serial 1-log_10_ dilutions of each extract and reference substance were created (Fig. S2^1^) and aliquoted into 384-well “master” plates (Masterblock 384-well, V bottom, Deepwell polypropylene plate; Cat. No. 781271; Greiner Bio-One North America, Monroe, NC) as follows: Plates (Fig. 2) contained 308 wells (all outer wells were filled with 200 μL of sterile distilled water to enhance temperature balance for the entire plate and were not used in the experiments) with one serial dilution of each of 141 petroleum substances and 20 reference chemicals, 20 intra-plate replicates (duplicate of the same dilution for 10 UVCBs and 10 reference chemicals), 20 inter-plate replicates (4 serial dilutions of 5 UVCBs), 55 negative controls (14 media, 13 DMSO (0.25–0.5%, final concentration identical to that in the assay wells for each cell type), and 28 “method blank” vehicle controls (see Fig. 1, 0.25–0.5% as for DMSO)). A total of 52 wells were left empty in the “master” plates so that cell type-specific positive controls (see Fig. S1 and Supplemental Files 1 and 2^1^ for details) could be added before experiments with each cell type. Plates were sealed with aluminum film and stored at −80°C until use. Copies of each master plate were prepared for use in all *in vitro* experiments. The final concentration of DMSO in assay wells following addition of test substances was 0.25–0.5% (v/v), depending on the cell type, as detailed in Supplemental Files 1 and 2^1^.

### In vitro *experiments*

A total of 15 human cell types were used in these experiments (Tab. 2). Cell type and vendor selections were based on the following considerations: Cells were chosen to be of human origin and to represent diverse organs/tissues. We used both “primary” cells, i.e., iPSC-derived cells, as well as a number of established cell lines. These *in vitro* models had to be reproducible (i.e., a particular cell/donor can be obtained from a commercial source) and suitable for evaluation of both “functional” and “cytotoxicity” endpoints so that we could assess the specificity of the effects of test compounds. Five of these cell types (hepatocytes, endothelial cells, neurons, cardiomyocytes and macrophages) were human induced pluripotent stem cell (iPSC)-derived (Fuji-Film-CDI, Madison, WI). One cell type was primary human umbilical vein endothelial cells (HUVEC) from Lonza (Basel, Switzerland). Eight cell types (A375, A549, HepG2, HLMVEC, HT29, LN229, MCF7, and SH-SY5Y) were from ATCC (Manassas, VA). HEPARG cells were from Sigma-Aldrich. All cells were cultured as recommended by their supplier (see Supplemental Files 1 and 2^1^ for details).

Cells were plated in 384-well plates in densities recommended by the supplier, using optimized media supplied by the same company or optimized for density by experimentation for each cell line. Cells were cultured without treatment for a period of time required to achieve functional capacity. Plating density, cell culture conditions and duration are detailed in Supplemental Files 1 and 2^1^. Cells were treated with petroleum substances and chemicals in a series of dilutions to evaluate concentration-response as described above (Fig. 2). For each cell line, a number of phenotypes (Tab. 2, Tab. S3^1^) were evaluated using high-content imaging and other read-outs as detailed in Supplemental Files 1 and 2^1^. Assay-specific controls (Fig. S2, Supplemental Files 1 and 2^1^) that were unique to each cell type were used to verify that each cell type exhibited expected functional and cytotoxicity responses.

### Data processing and quality assurance

The experimental design consisted of running all of the petroleum substances on a single plate at one concentration (Fig. 2). As a consequence, the concentration response can be evaluated only when considering all four plates (each at a different dilution). To account for this, a number of inter- and intra-plate controls were included to ensure that the concentration response was not affected by artifacts of the experimental design. Inter-plate controls consisted of 5 petroleum substances, which were present in all four dilutions on each plate. These responses could then be compared to the concentration response across the four plates to ensure that similar responses occurred within a plate and across plates. Due to running only a single replicate, intra-plate controls were added to ensure that the single values were consistent within a plate. Ten petroleum substances and 10 reference chemicals were assayed as a duplicate on each plate. These were arrayed at the same concentration as those normally-placed substances and were used to ensure reproducibility within a plate.

Raw data generated during *in vitro* experiments was normalized to the average of “method blank” vehicle control (Fig. 1) wells. The normalized values represent a percent response to the method blank. Normalization was performed for all raw values assessed, including the positive and negative controls. The normalization process followed the formula (1):

(1) NormalizedValue=(RawValue)/(Averageof“MethodBlank”Wells)×100

**Fig. 3:**
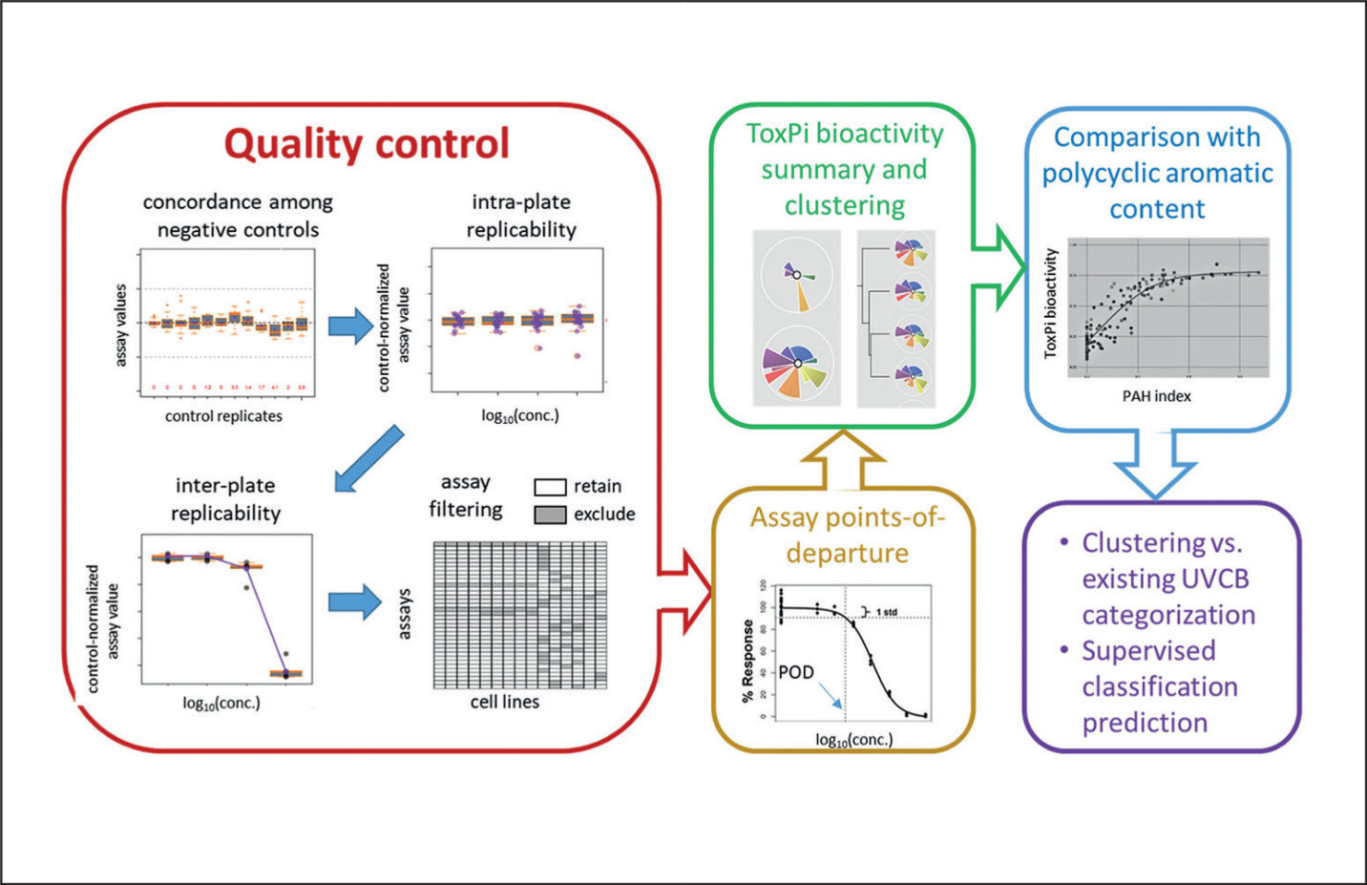
Data analysis workflow Extensive quality control (QC) steps were used to filter assay/cell line combinations to ensure high concordance among controls and high intra- and inter-plate reproducibility. For the assays passing QC, points of departure were estimated using logistic (Hill) function curve fitting, and overall and cell type-specific measures of bioactivity computed across the assays. Analysis of bioactivity was further grounded in comparisons of polycyclic aromatic content (PAC). Finally, existing UVCB categories were compared to unsupervised clustering of the emergent data, as well as using trained (supervised) models to “predict” the categories.

To ensure the integrity of the data, several metrics were calculated for each phenotype. All data for quality assurance (Fig. 3) of each cell type are included in Supplemental Files 1 and 2^1^. Quality control was programmatically conducted to identify excessive variation in 3 ways in this high-dimensional, high-throughput experiment. An assay was considered QC “fail” if any assay flag was called across three assessments of experimental variation. First, plate controls were examined. For a given plate (consisting of a single cell type/assay/dose combination), method blank controls were mean-centered to 100, while DMSO-/media-controls were normalized to method blanks. An assay was flagged for excessive control variation if the method blank inter-quartile range (IQR) exceeded the 75/125 boundary or if the entire IQR of DMSO-/media-controls existed outside the 80/120 limits of the mean-centered (mean = 100) method blank controls. Second, excessive inter-plate variation was assessed across dose response. Five substances were plated on each dose plate as complete dose response. This means each dose plate (1000× through 1×) had 5 substances that were plated for 1×, 10×, 100×, and 1000× concentrations each, yielding 4 data points at each dose. The single test substance data point on the plate was then compared to the mean and standard deviation (SD) of the other 4. A substance was flagged if its value exceeded 1.75×SD for 2 or more doses. If this occurred for 3 or more chemicals, the assay was flagged. Third, excessive intra-plate variation was assessed with 20 substances that were plated in duplicate on each plate. The IQR of method-blank controls for the plate were compared to the IQR of a scaled replicate difference (((rep1 - rep2)/sqrt(rep1*rep2))*100) of the 20 substances, and a concentration was flagged if the IQR (20 substances) was greater than 1.75×IQR of the method blank controls for the respective plate. The assay was flagged if more than 1 concentration was flagged. The number of pass/fail phenotypes for each cell type is shown in Table 2. Details on each quality control “flag” for cell type/phenotype are provided in Table S3^1^.

### Dose-response analysis and derivation of the points of departure

After normalization and quality control, a point-of-departure (POD) was calculated for all phenotypes that were determined to pass quality control (Tab. S3^1^). Vehicle control-scaled data for each test substance and phenotype were fitted to a curve with a nonlinear logistic (Hill) function to determine POD values, defined as the concentrations at which the fitted curve exceeds one standard deviation above or below the mean of vehicle-treated controls, using R software-based scripts (Supplemental File 3^1^) as previously reported (Sirenko et al., 2013). The choice of one standard deviation “benchmark response” was based on the US EPA guidance for dose-response modeling and determination of the point-of-departure values (U.S. EPA, 2012), as well as empirical testing of various thresholds as detailed in (Sirenko et al., 2013), which showed that a choice of one standard deviation generates consistently high classification accuracy. Each concentration-response graph with the logistic fit was visually inspected to ensure goodness of fit. Several aspects were considered including degree of fit, trend of data points, and consideration of removal of outlier data. The final POD was derived using a decision tree as shown in Figure S3^1^.

### Calculation of the Toxicological Priority Index (ToxPi)

ToxPi is a computational approach for data integration (Reif et al., 2010, 2013). The ToxPi Graphical User Interface (Marvel et al., 2018) was used to integrate and visualize data from different cell types and phenotypes. POD values for each phenotype passing quality control were inversely normalized on a 0–1 scale, with 0 representing the highest POD value in a given data set (i.e., the lowest observed bioactivity) and 1 representing the lowest POD value (i.e., the highest observed bioactivity) using formula (2):

(2) $$ToxPi\;Value=1-\frac{(log_{10}(POD)-log_{10}\left(POD_{min}\right)}{log_{10}\left(POD_{\max}\right)-log_{10}\left(POD_{min}\right)}$$

### Clustering and classification analyses

We used two approaches to grouping petroleum substances based on the biological profiling data produced in this study. In an unsupervised analysis, substances were grouped based on the similarity of their bioactivity profiles, without prior knowledge of manufacturing stream categories. To evaluate the outcome of such grouping, we included a quantitative metric into the unsupervised analysis workflow to assess the correspondence of the outcome to the original categories of each chemical. The details of the unsupervised analysis workflow are described elsewhere (Onel et al., 2019). Briefly, clustering was performed using the hclust function in R, using average linkage clustering applied to a Euclidean distance metric on centered, scaled data (essentially Pearson correlation), which we have previously found to be reasonably robust (Onel et al., 2019). The Fowlkes-Mallows (FM) index (Fowlkes and Mallows, 1983), a measure of similarity of two clusters, was calculated to enable quantitative comparative assessment between groupings achieved using each dataset to the known chemical categories. The higher the FM index, the more similar the grouping based on *in vitro* or chemical descriptor data was to the *a priori* determined grouping as shown in Table 1. The FM index ranges from 0.0 (no correspondence) to 1.0 (perfect correspondence). One-sided *P*-values for the FM index (using the null hypothesis of random assignment) can be obtained using a standard z-statistic (Fowlkes and Mallows, 1983). However, to improve confidence in the findings, and to compare FM indices, we adopted a permutation approach. Specifically, we performed 100,000 permutations of the actual sample groupings, and for each clustering computed the resulting FM index for each permutation to calculate a one-sided *P*-value. In order to compare the FM index for two clusterings (which we label A and B, respectively), we compared the observed FM_A_-FM_B_ value to the permutation distribution of |FM_A_-FM_B_| to obtain a two-sided *P*-value.

In the supervised analysis, 8 analytical measurements and 42 cell assay bioactivity profiles (see below) were used to train a machine-learning statistical model using the Prediction Analysis of Microarrays (PAM) package in R (Tibshirani et al., 2002) to predict the existing categorizations as shown in Table 1. In contrast to the unsupervised approach, a supervised model is trained to recognize the features that are most predictive of the pre-defined classes. Importantly, the approach can be used to identify substances that are difficult to classify, or pre-defined classes that are difficult to distinguish from each other. As some of the UVCB categories were small, *k*-fold cross-validation methods were difficult, as some random-fold outcomes might include zero instances of a category. Thus, our application of PAM used a leave-one-out cross-validation, and a shrinkage threshold of 1.28 (the 90% quantile of a standard normal distribution). In order to understand the “null” accuracy of a random classifier, we performed 1 million permutations of the categorizations, matched up with the actual category vector, recording the accuracy (mean number of category-matching UVCBs) for each permutation. The 95^th^ percentile of these permuted accuracy values (0.163 = 16%) was then used as a null significance threshold to compare the accuracy for the actual classification rules.

### Polycyclic aromatic compound (PAC) analysis

Weight percentages of the polycyclic aromatic compounds in all tested petroleum substance samples were determined by gas chromatography-coupled mass selective detection (GC/MSD) as detailed previously (Roy et al., 1988). Briefly, each substance was extracted as detailed above and dried. The amount of each extract was then determined using the weight difference of the empty flask and following solvent evaporation. The extract was then dissolved in cyclohexane to a final concentration of 50 mg/mL and used for analytical assays. Sample separation was achieved on a Zebron-5HT capillary column (30 m; 0.25 mm; 0.25 mm; Phenomenex, Torrance, CA). Quantitative integration of the chromatograms was achieved using standards of naphthalene, phenanthrene, 1,2-benzanthracene, benzo[a]pyrene, benzo[g,h,i]perylene, and coronene. The resulting PAC profiles consist of weight percentages by ring number and are listed in Table S4^1^.

## 3 Results

This study tested an integrative approach based on alternatives to animal models to support biological coherence and integrative grouping of complex petroleum substances. We reasoned that bioactivity “signatures” of these substances in a large number of human cell types will be informative to (i) assess the validity of existing groupings, and (ii) determine whether “representative” substances can be identified in each category so that they can be further considered for regulatory-required assays and that information used for read-across in each group. Both goals will increase effectiveness of any future testing and facilitate a NAM-informed approach to meet regulatory requirements. By including in this project a large number of petroleum substances that are registered under REACH in the European Union (CONCAWE, 2017), we aimed to provide broad coverage of the categories and individual substances. This dataset is unique insofar as it represents the largest standardized and tightly quality-controlled NAM dataset on petroleum substances.

Quality control analysis (Fig. 3) of the data that was collected on 141 petroleum substances for a total of 71 phenotypes in 15 cell types utilized various controls that were engineered into the experimental design (Fig. 2). All bioactivity assays were evaluated to flag assay and cell line combinations with potentially high signal-to-noise ratios (Tab. 2, Tab. S3^1^). First, during the data collection phase, several upstream quality control procedures using positive controls were implemented in order to determine that the cells were responding according to expectations in the published assays. Second, additional analyses to assess the overall quality of the bioactivity profiling data were based on three criteria: (i) concordance of three types of negative controls (media, DMSO, and “method blank” vehicle), (ii) inter-plate replicates, and (iii) intra-plate replicates. A total of 42 assays in 12 cell types were deemed as high quality, and reproducibility satisfied the QC thresholds. The quality control procedures were implemented as “flags” for each assay in each cell line so that downstream analyses could be compared in which flagged assays were either included or not included. Data from these 42 assays were used in further data analyses.

**Fig. 4:**
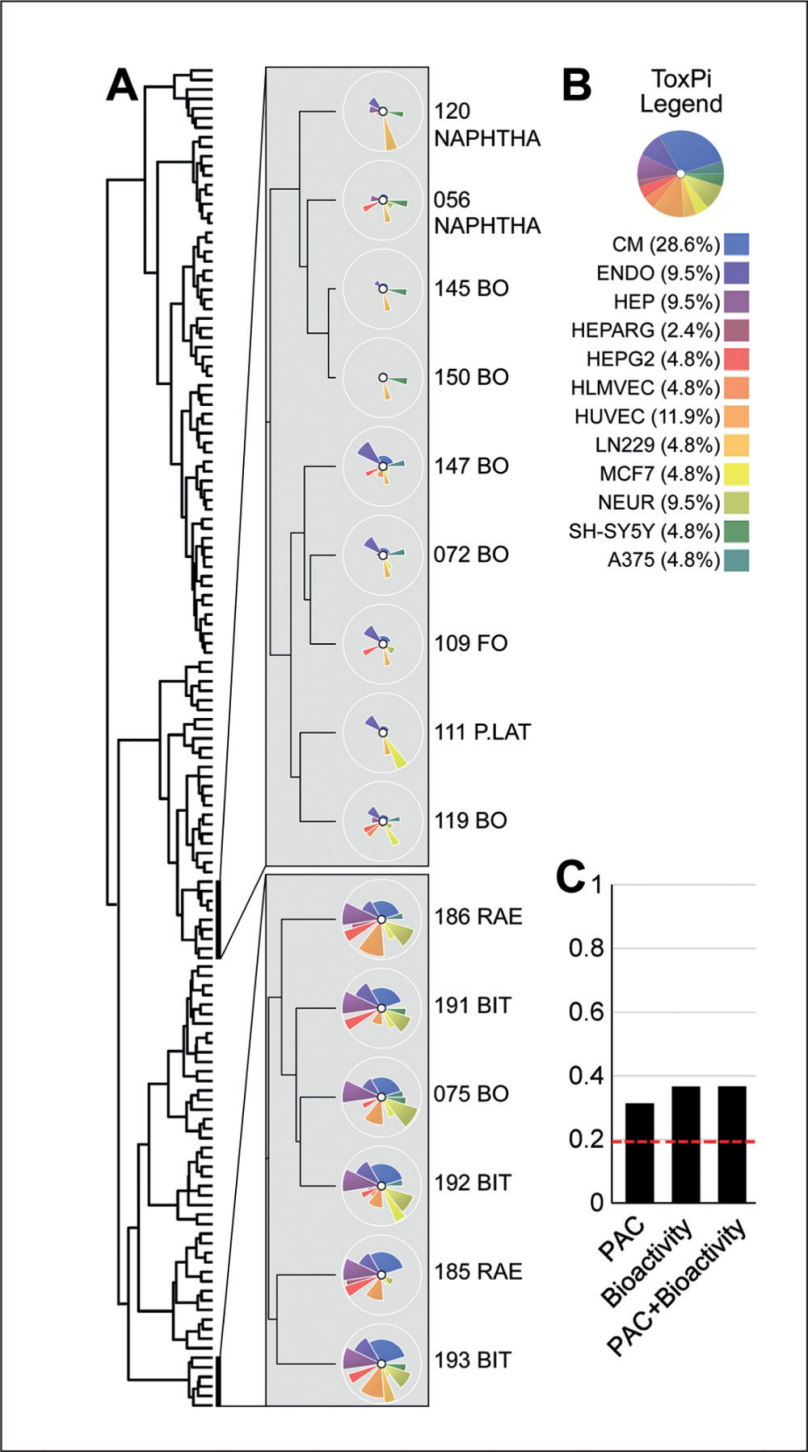
Unsupervised grouping of petroleum substances (A) Clustering dendrogram of the 141 UVCB samples, illustrating that clustering reflects both overall bioactivity and specific patterns of bioactivity across the cell types. See Table S5^1^ for ToxPi GUI input file that can be used to recreate the dendrogram and ToxPi images. (B) ToxPi legend representing each included cell type as a colored slice with weight indicated in parenthesis based on the number of cell type-specific phenotypes included (Tab. 2). (C) Fowlkes-Mallows index comparing the existing 16 UVCB category designation of tested petroleum substances to unsupervised clustering using polycyclic aromatic compound data (PAC, Tab. S4^1^), to the bioactivity summary based on the cell assays only (data shown in panel A), and to the combination of the two. Red dotted line shows the approximate permutation-based threshold of significance, which varies slightly for the three instances shown. Permutation-based p-values for clustering correspondence compared to a null model were less than 10^−5^ for each of PAC, bioactivity, and the combination. The accuracy of the three models did not differ significantly from each other.

Unsupervised clustering analysis of the data integrated with the ToxPi approach was used to determine whether petroleum substances can be grouped based on their bioactivity profiles across all cell types and phenotypes. Figure 4A shows the results of clustering with two insets depicting two representative clusters. One shows a low bioactivity cluster and another shows a high bioactivity cluster; in both, samples from the same/similar categories (Tab. 1, Tab. S1^1^) show very similar ToxPi profiles. The exception is sample 075, which was from the base oils (“BO”) category and is a clear outlier with respect to its bioactivity as compared to other BO samples. However, it is noted that the BO category has larger inherent compositional variability compared to other petroleum substance categories. Consequently, sample 075 is likely to represent a less extracted example and could guide selection of a “worst case” candidate for follow-up testing.

A quantitative comparison of the unsupervised analysis was conducted using the Fowlkes-Mallows (FM) index (Fowlkes and Mallows, 1983; Onel et al., 2019). The results of the bioactivity-based clustering, or the data on PAC of each sample, a common approach to define health risks of petroleum substances (Redman et al., 2014), were compared to the known chemical groupings (Tab. 1) that were used as a reference. Figure 4C shows that the correspondence of clustering to the known groupings was highly significant (more accurate than expected by chance) when using either PAC data, bioactivity data, or their combination (*P* < 10^−5^ for all comparisons). Although the clustering correspondence of bioactivity profiles was somewhat higher than that based on PAC (3–7 ring) data alone (Fig. 4C), there was no significant difference between the two. Among the individual cell types, iCell hepatocytes showed the highest FM index (FM = 0.41), albeit it was not significantly different from that for other cell types.

**Fig. 5:**
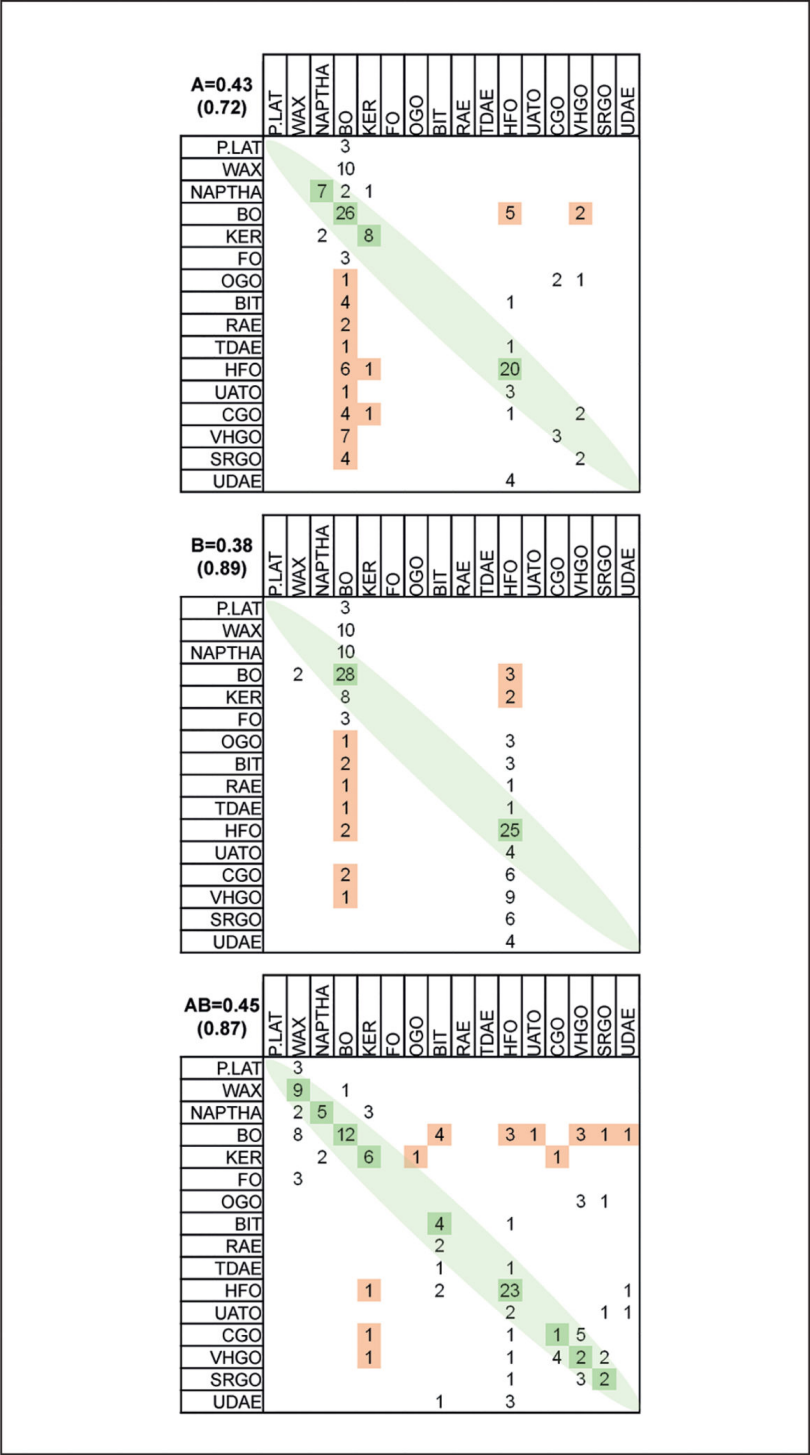
Analysis of the relationship between analytical (PAC) and summarized bioactivity data Top (A): The results of supervised analysis in which the UVCB category is predicted from the pattern of PAC analytic data using the PAM classification procedure. Rows refer to the true category, and columns to predicted category. Correct classification counts are shown in green as values on the diagonal. Categories are ordered according to median bioactivity score, so misclassifications near the diagonal are not severe, while misclassifications into categories with substantially different hazard profiles are shown in orange. Middle (B): correct classifications and misclassifications using bioactivity patterns only, which tends to predict as the most frequent categories of base oils and heavy fuel oils. Bottom (AB): correct classifications and misclassifications using both analytic and bioactivity data, which shows slightly higher accuracy than PAC analytical data alone. Numbers are correct classification rate when only exact matches are considered, or (in parenthesis) when misclassifications were not into a substantially different hazard category.

We also used the *in vitro* bioactivity data and PAC content to develop supervised predictive models for the manufacturing stream-based categories. The term “supervised” denotes that we use the existing categories to train a model and then apply a “leave-one-out” approach to predict in which category the specific UVCB belongs based on its aromatic ring class profile (i.e., PAC (3–7 ring)) and/or biological (i.e., ToxPi score from all data combined) profile. The leave-one-out approach ensures that the classification accuracy is informative, because each of the 141 UVCBs is held out in succession and not used in training the model. Figure 5 shows statistical classification accuracy for 3- to 7-ring PAC data (accuracy 43%) and bioactivity data (accuracy 38%). The combination of PAC and bioactivity data did not result in a significant increase in classification accuracy (45%). All of these values are considerably greater than the null accuracy threshold of 16% described earlier. The categories were ordered by mean PAC 3–7 relative content, and exact matches are marked in green. One salient feature of classification using bioactivity data alone is that predictions tend to concentrate on the two largest categories, BO and HFO. It is also apparent that relatively few of the category-assignment errors are in fact assignments to categories of very different PAC 3–7 content (shown in orange). When assignments to “distant” categories are considered as most consequential misclassification, the correct classification rate of the bioactivity data alone, or in combination with PAC data, is close to 90%, well above that for the PAC data alone (72%). The combination of PAC data and biological data not only yields the highest accuracy, but the spread of classifications across the categories is more easily explainable in terms of the 3–7 ring PAC content of the substances related to their observed biological activity.

**Fig. 6:**
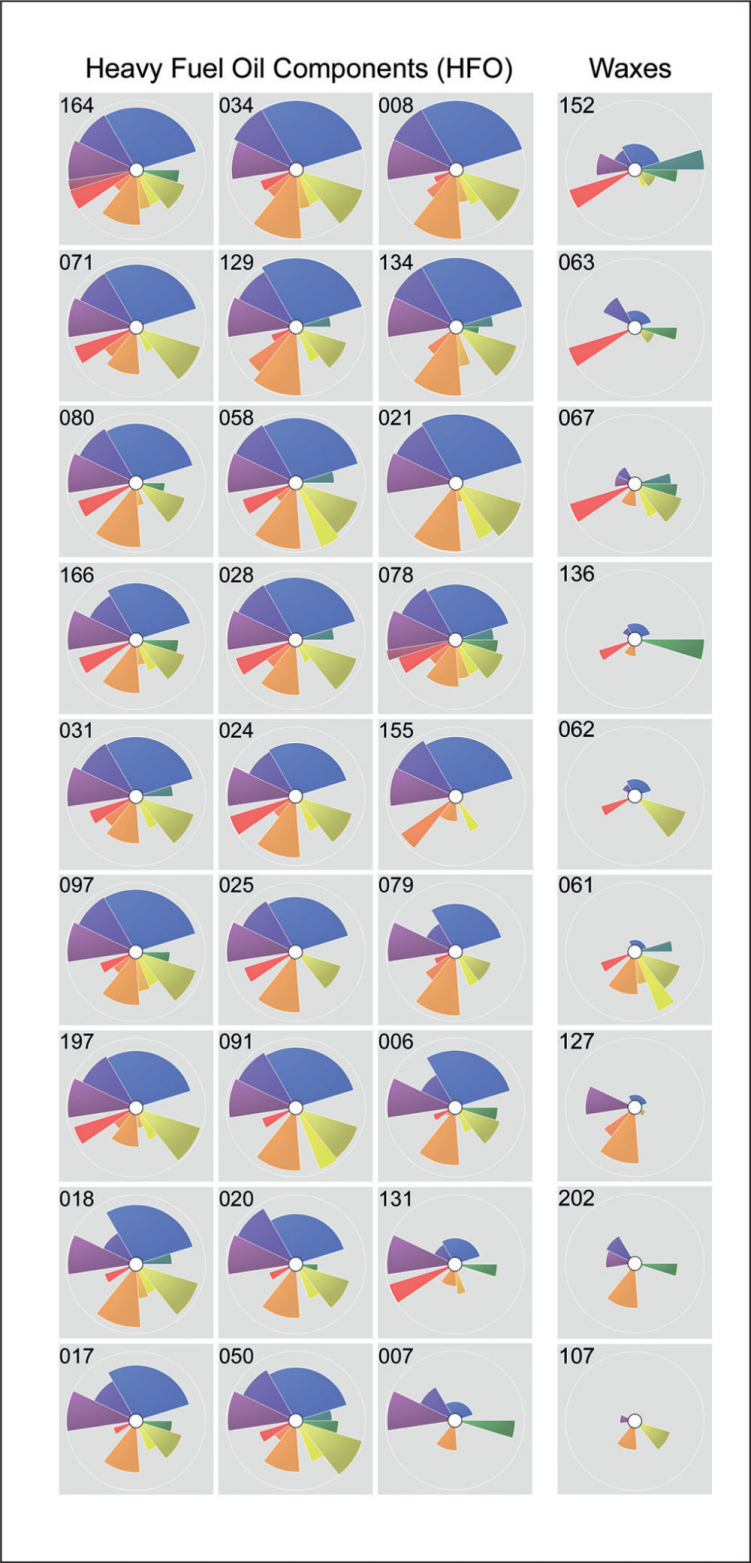
ToxPi plots show striking similarity of bioactivity patterns among heavy fuel oil (HFO) components, and among some waxes

A different question that can be asked with the bioactivity data on the petroleum-based UVCBs is whether substances that belong to a manufacturing stream-based category exhibit similar profiles, as this type of information is available to provide additional contextualization. Figure 6 shows examples of two categories, heavy fuel oil components (HFO, 27 individual UVCBs) and waxes (9 individual UVCBs). The HFO category is defined (CONCAWE, 2017) as streams obtained as either distillates or residues from distillation and cracking processes and containing saturated, aromatic and olefinic hydrocarbons in a wide boiling point range. The waxes category in this study included substances from three closely related groups – slack waxes, and paraffin and hydrocarbon waxes. These wax substances are derived from vacuum distilled fractions and separated as a solid by chilling. Many of these substances are subject to further intermediate processing such as de-oiling or treatment with acid, clay, active carbon or hydrogenation to remove most of the polycyclic aromatic hydrocarbons (PACs). ToxPi profiles of most UVCBs that are classified as belonging to the HFO category look very similar; these substances have relatively high bioactivity across most iPSC-derived cell types, especially iCell hepatocytes, commensurate with their high content of PACs. Only two substances, 131 and 007, show a qualitatively different ToxPi profile with little effect on iCell cardiomyocytes and neuronal cells; therefore, these two substances may be considered as not representative of the overall bioactivity of the HFO category. In the waxes category, much less similarity is evident among the individual members; however, all of these substances had a markedly lower bioactivity compared to HFOs.

**Fig. 7:**
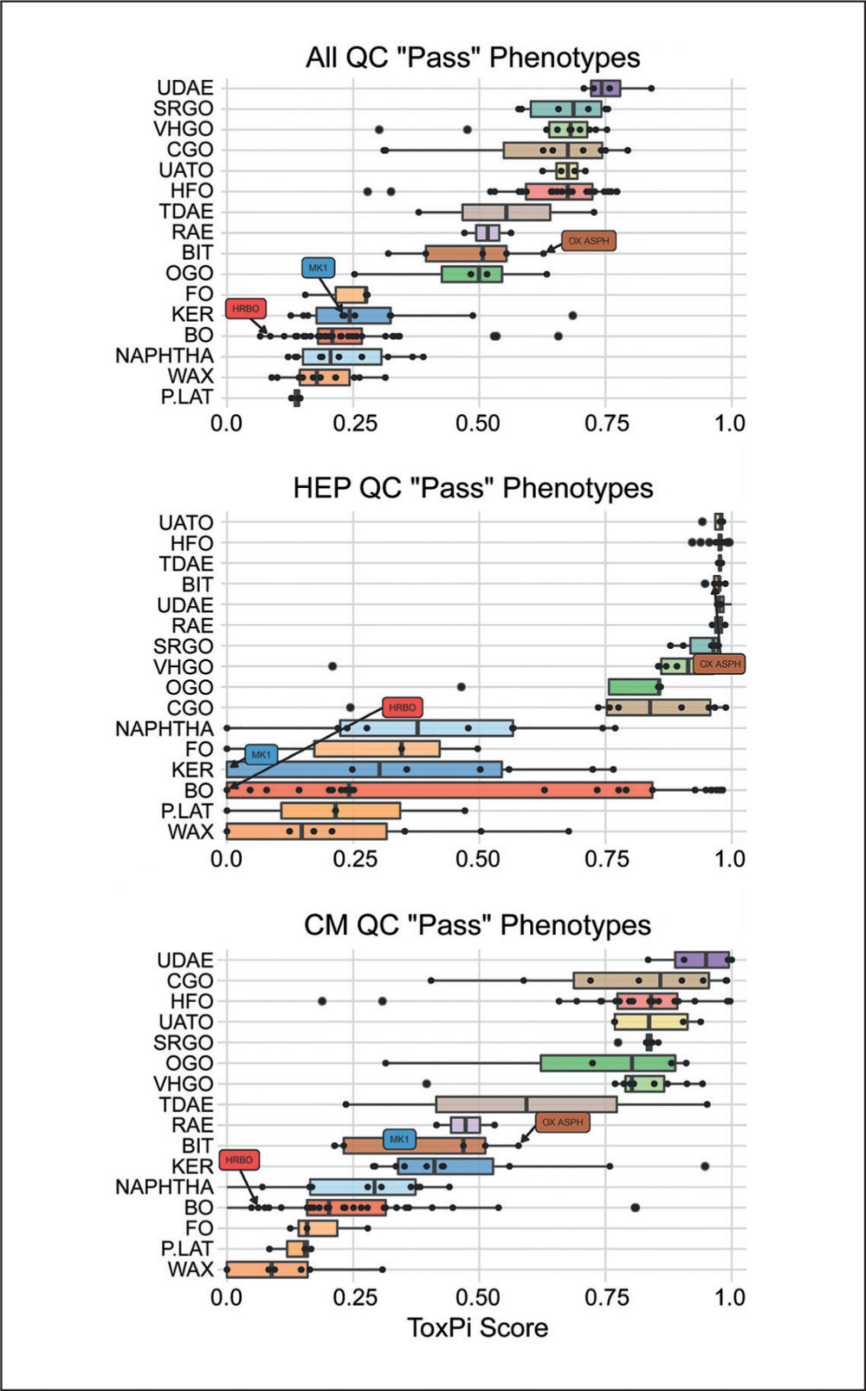
Manufacturing stream-based grouping of the bioactivity for individual petroleum substances Using the ToxPi score as an overall measure of bioactivity for each UVCB, striking differences are observed across UVCB categories (top). This phenomenon differs by cell type, with iCell hepatocytes showing two clear groups of bioactivity (middle), and iCell cardiomyocytes showing a gradation across the categories (bottom). Each dot represents a UVCB sample total ToxPi score derived from all phenotypes (top) or cell-specific phenotypes. Box is the inter-quartile range, vertical line is the median, and whiskers extend in most instances to the min-max range of values, and otherwise to the corresponding quartile plus 1.5X the interquartile range. See Figure S4^1^ for the same data for other cell types.

To determine the overall bioactivity of each manufacturing stream-based category and the heterogeneity of the individual UVCBs in each category, we grouped ToxPi scores for all 141 substances (Fig. 7). When all high-quality phenotypes are combined to derive a ToxPi score as shown in Figures 4 and 6, a clear pattern in the bioactivity of each category emerges – aromatic extracts and gas oils have high mean bioactivity scores, while highly refined and chemically treated substances such as petrolatums and waxes have the lowest bioactivity. When data were examined for each cell type separately (Fig. 7, Fig. S4^1^), additional patterns were discernible. For example, the iCell hepatocytes showed separation into two broad bioactivity regions, whereas the iCell cardiomyocytes showed a gradient of bioactivity among the categories in the bottom half of bioactivity. At the same time, the data from many other tested cell types were not informative with respect to grouping (Fig. S4^1^). It is noteworthy that a high degree of heterogeneity was present within each category, especially among HFO and BO categories. This finding is interesting because current manufacturing stream-based categories include substances with widely varying PAC content and considerable overlap exists between categories that are not always very similar from a refining perspective.

**Fig. 8:**
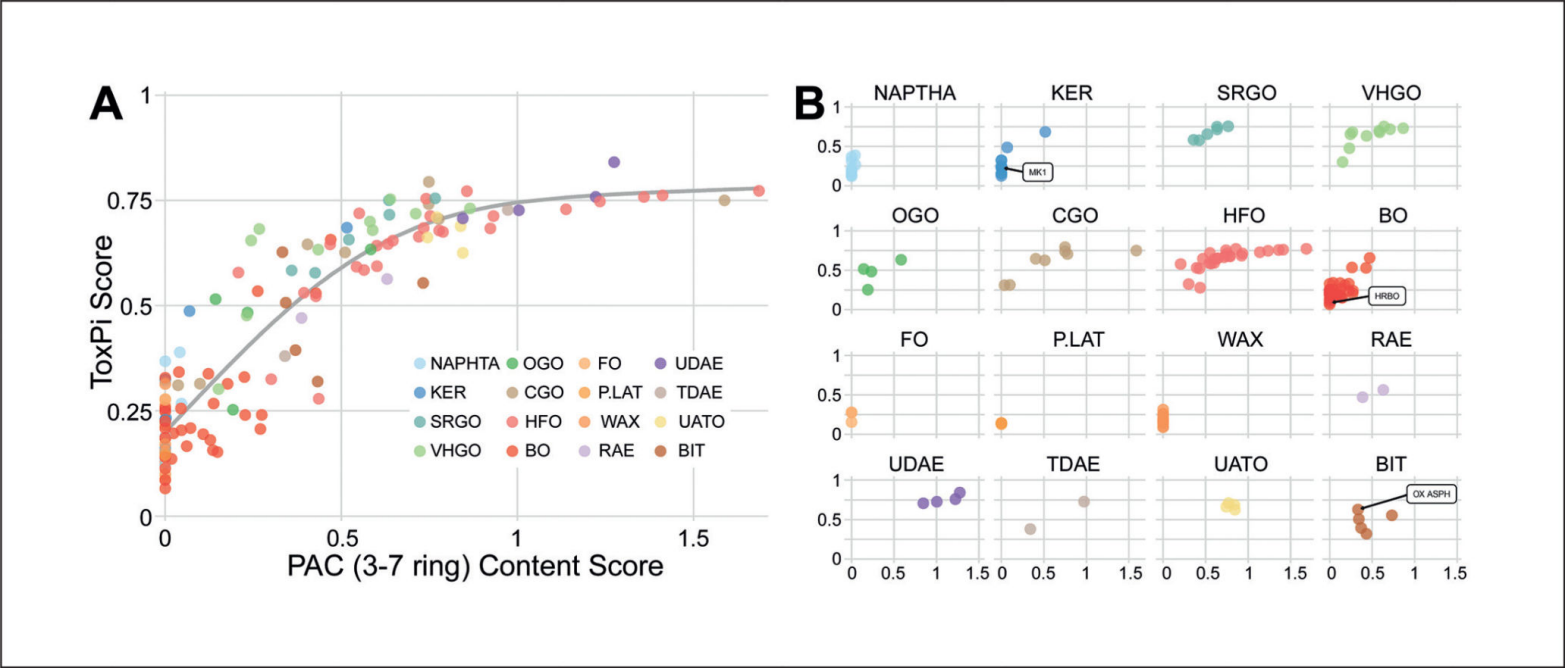
Polycyclic aromatic compound (PAC) score for 3–7 ring compounds in each UVCB sample is highly correlated with the overall ToxPi bioactivity score (A) For all UVCB categories, PAC 3–7 ring content explains ∼80% of the variation in ToxPi bioactivity. (B) The relationships depicted for each grouped category. See Figure S5^1^ for cell type-specific correlations.

To test the hypothesis that *in vitro* bioactivity profiles may be associated with PAC content of each individual UVCB (Tab. S4^1^), we examined the correlation between these two parameters (Fig. 8). The bioactivity, expressed as a total ToxPi score for each substance, was compared for each UVCB to the 3–7 ring PAC content expressed as a proportion of DMSO-extractable PACs. Specifically, 3–7 ring PAC content score was calculated by taking the sum of aromatic ring content (for 3 through 7 ring-containing constituents) multiplied by the percent total weight of DMSO-extractable PAC (Gray et al., 2013). Consistent with the hypothesis, the overall fit based on all *in vitro* data combined (Fig. 8A) showed a strong positive correlation (Spearman rho = 0.89) with the relative PAC 3–7 ring content of each substance. Figure 8B shows examples of the same relationships when UVCBs were grouped using their manufacturing stream-based categories. For those categories with relatively higher 3–7 ring PAC content, or spanning a wide range of PAC content, strong trends in the categories showing increased bioactivity correlated to increased 3–7 ring PAC content are observed. In contrast, these trends were not observed for categories with low to negligible 3–7 ring PAC content. We also examined the relationship between PAC content across aromatic ring compounds and bioactivity (Tab. 3). We found that 3–7 ring PAC content had the strongest correlation, followed by PAC 3-ring content and PAC 4-ring content. These results corroborate the known relationship between the content of PAC, especially of 3–7 ring type, in the petroleum refining products with their potential health hazard (McKee et al., 2015; Gray et al., 2013). Interestingly, when the ToxPi scores for each cell type were correlated with PAC (3–7 ring) content of each substance, we found that data from iPSC-derived cell types and HUVECs were as informative as all data combined (Tab. 4, Fig. S5^1^).

## 4 Discussion

Regulators are tasked with assessing the risk to human and environmental health from substance exposure, including complex substances, while reducing the use of animal testing. In order to facilitate these objectives, we aimed to determine whether NAMs that are based on *in vitro* bioactivity can be used to ascertain substance similarity among complex petroleum substances. These substances are manufactured in high volumes and have widespread uses and as such are subject to stringent regulatory scrutiny (McKee et al., 2015, 2018). A large number of mammalian toxicology studies have been generated on these substances based on the requirements of previous regulatory frameworks, such as the Dangerous Substances Directive in the EU, and the High Production Volume Program in the USA. In general, lower boiling petroleum streams that do not contain PAC are known to exhibit lower tier toxicological effects, such as mild skin irritation and in some cases central nervous system effects at higher dose levels. In contrast, heavier and high boiling petroleum substances, starting from some of the gas oils, have increased amounts of PAC constituents commensurate with potential to cause systemic toxicity as well as carcinogenicity and reproductive toxicity (Roth et al., 2013; Feder and Hertzberg, 2013; McKee and White, 2014; McKee et al., 2014). These data have been used to fulfill requirements in the REACH dossiers, and the knowledge of the composition and refining processes, coupled with the existing health hazard data, formed the basis for developing the grouping, read-across and testing hypothesis.

Although registrations for the petroleum substances were submitted for the 2010 REACH high tonnage deadline, multiple data gaps were identified. Where read-across could not be applied, testing proposals to fill these data gaps were included in the respective REACH dossiers. An alternative to new testing is the use of read-across, which requires detailed analytical data, ideally describing the full substance or all of its constituents (CONCAWE, 2019). However, the European Chemicals Agency (ECHA) is concerned about the paucity of available information on chemical composition and as a result has challenged the read-across assumptions in petroleum substance submissions. This has led to requests for additional analytical chemistry and toxicology data to better characterize similarity, justify the read-across, and address data gaps. A representative decision can be found in (ECHA, 2020). One approach to establish substance similarity is to consider chemical composition and/or physico-chemical properties. The process of grouping petroleum substances for regulatory decision-making and read-across traditionally relies on the physical/chemical properties, manufacturing process, and similar end uses (McKee et al., 2015). However, given the inherent chemical complexity of petroleum substances, as well as a lack of regulatory guidance as to what data may conclusively demonstrate substance similarity, defining chemical groupings and applying the read-across remains challenging.

This project hypothesized that NAM-based biological activity fingerprints, in conjunction with the existing grouping strategy (i.e., manufacturing process, physico-chemical characteristics, and performance specifications), would strengthen the justification for substance similarity (or disparity). Specifically, we argue that availability of the orthogonal data (i.e., physical, chemical, and biological) on the same substance(s) should enhance confidence in the application of read-across for petroleum substances. Indeed, integration of chemical structure, physico-chemical properties, and biological data (*in vivo*, *in vitro* and *in silico*) has been shown to offer a number of advantages (Low et al., 2011, 2013; Rusyn and Greene, 2018; Zhu et al., 2016) and was encouraged by the US National Academies (National Research Council, 2014; National Academies of Sciences Engineering and Medicine, 2017). We suggest that expanding the regulatory principle of “read-across” hypotheses to include *in vitro* bioactivity data could address uncertainties and increase confidence and the transparency of decisions. Still, it should be noted that NAM-based data are intended to support grouping of petroleum substances and are not to be used for hazard evaluation. To subsequently support hazard evaluations and read-across, testing and read-across hypotheses may be developed using newly collected *in vitro* and existing *in vivo* data connected to the petroleum substance based on the chemical composition. This will facilitate selection of representative substances for further *in vivo* testing, if needed, and the read-across from these substances.

Previous studies (Grimm et al., 2016; Chen et al., 2020) have shown that incorporation of the bioactivity data helps underpin substance grouping and prioritize substances for which further work is needed to inform regulatory assessments. The present work provides further evidence that bioactivity profiling of complex UVCBs is a feasible path towards characterization of “sufficient similarity” for complex substances. It is the largest “case study” to date aimed at testing whether and how *in vitro* bioactivity data can be used to inform grouping of UVCBs. By including the large number of substances, cell types, and endpoints, we show that the approach is broadly applicable, not only with respect to grouping of petroleum substances but also other UVCBs and mixtures. Indeed, our study demonstrates clear (clustering) relationships between *in vitro* bioactivity profiles and the class assignment of DMSO extracts of petroleum substances.

It is acknowledged that using a DMSO extract of the substance means these biological activity data are not representative of the full substance. Even though DMSO selectively extracts lipophilic constituents including PACs from test substances, the chemical profiles of PACs across molecular classes remain consistent after extraction (Luo et al., 2020). Modelling results from *in vivo* testing of a range of petroleum substances indicated that the higher-tier toxicological profile of high-boiling petroleum substances is related to the types and levels of PAC (Nicolich et al., 2013). Thus, the DMSO extracts represent the “biologically active” fraction, i.e., [3–7 ring] aromatics, of the refinery streams (ASTM International, 2014; Roy et al., 1988), although additional constituents (i.e., all polar molecules) are also extracted, which explains that certain refining streams with low to no (3–7 ring) PAC content still have low levels of extractable materials. Furthermore, the extracts obtained using this method are used routinely for safety (e.g., mutagenicity) testing and chemical characterization of the refinery streams (CONCAWE, 1994; Carrillo et al., 2019).

Because this project was not aimed at hazard identification of petroleum substances, the use of a normalized control allowed direct comparison of the results both within and across substance categories. ToxPi scores were based on a relative comparison of the cumulative effects of the analyzed substances. ToxPi scores are informative only in the context of a particular dataset. ToxPi profiles of the individual substances aid in visualizing the patterns in the effects of each substance on *in vitro* cell-based models. Indeed, we observed overall strong correlation between bioactivity and the categories of UVCBs. For example, HFOs, which have overall much higher PAC content than waxes, in general showed high bioactivity, whereas waxes showed low bioactivity.

We also tested whether multi-dimensional *in vitro* bioactivity and analytical data on petroleum substances can be used to classify them into categories. We found that each of these data streams individually is statistically significant in its ability to predict the category the substance may belong to, even though some misclassifications can occur due to the complex nature of these substances. Importantly, combinations of these data, a so-called chemical-biological read-across, appeared most powerful in eliminating misclassifications. These data offer strong support for the utility of orthogonal supporting biological and physico-chemical data streams to increase confidence in grouping of complex UVCBs.

It is well established that even within manufacturing categories of petroleum substances (e.g., HFO), the 3–7 ring PAC content can be variable between its category members. Existing groupings of petroleum substances contain category members with considerable inter- and intra-category overlap as expected based on their physico-chemical characteristics and manufacturing processes (CONCAWE, 2017). Petroleum substances are a continuum in terms of their chemical composition. With “adjacent” streams overlapping to some extent, there will be overlap between the heavy end of a low boiling stream and the light end of the adjacent higher boiling stream. It is therefore significant that the bioactivity data collected in this study were able to clearly identify substances that should not be placed into the same category based on their refining properties and/or product specifications. Specifically, we found considerable variation in bioactivity within some categories, for example HFO have a large range from low to high PAC-containing substances, which is reflected in the spread of bioactivity observed in this category. This trend was enforced by the fact that the biological data separate out the two foots oils from the HFOs. This is well explainable as foots oils are much closer to waxes from a refining perspective.

It is also noteworthy that both overall, and even within groupings, the 3–7 ring PAC content of tested substances correlated strongly with bioactivity. This finding suggests that petroleum substances can be ranked in the chemical-biological space representing the continuum of petroleum substances. On the one hand, this finding is in agreement with the “PAC hypothesis” for petroleum substances, which states that certain specific toxicological effects observed in heavier (average molecular weight) substances are associated with the level of 3–7 ring PAC in these substances (Murray et al., 2013; Goyak et al., 2016; Kamelia et al., 2019). On the other hand, our finding of strong correlation was independently derived using *in vitro* data from many cell types. Interestingly, unlike in cell types that retain basic physiological functions, such as iPSC-derived cells, no significant correlation was observed in cancer-derived cell lines. It can be speculated that genomic alterations in cancer cell lines may result in a wide range of impacts on physiologic function that may interfere with chemical stimulus-specific responses.

This study adds valuable information to the overall weight of evidence for selecting complex petroleum substances for grouping and then identifying the “representative” substance(s) for additional testing. Trends in the total ToxPi scores and their correlation with PAC 3–7 ring content are helpful for selection of the most representative substances from each petroleum substance category for further testing. This is needed for an overall integrative testing strategy that limits the need for testing in animals for toxicological assessments of petroleum substances. For example, a substance that is believed to have the highest potential to show a positive finding in an *in vivo* study based on its biological activity data linked to its chemical composition can be selected for a specific endpoint, and the data generated on this sample can then be conservatively applied to the entire category. Still, to cover the full chemical composition of petroleum substances, additional data may be needed for other molecular classes of constituents.

The read-across approach that is applied here is slightly different from “classical” read-across approaches that are not applicable to UVCB substances and might be more similar to the “bridging principle” as referred to in the classification and labelling regulation (UNECE, 2020). Because the physical chemistry of petroleum refining leads to a continuum of substances, there will be significant overlap between different substance categories: the heavy end of a lower boiling refining stream will overlap with the light end of the neighboring higher boiling refining stream. These are concepts that are of critical importance for chemical-biological grouping and read-across of petroleum substances. These insights will help facilitate an adapted read-across framework specific to UVCBs by applying the bridging principle.

Another important outcome of this study was a determination of what kind of *in vitro* models are most informative in terms of decision-making for complex petroleum substances. In this study, we found that data derived from assays of biological activity in iPSC-derived models were highly informative. This finding may be the product of the retained organotypicity of these cell types as compared to established cancer cell lines. Specifically, data from iPSC-derived hepatocytes was most informative for separating the substances in terms of their overall bioactivity trends, which is consistent with the ability of these cells to metabolize PAC-containing substances to reactive intermediates. Also, assays based on iPSC-derived cardiomyocytes provided separation between the UVCB categories with substances that have low to negligible PAC content, which suggests that cardiomyocytes can be a highly informative *in vitro* model (Chen et al., 2020) for substances without a defined toxicity pathway. These data also suggest that different types of molecules other than PAC play a role in the observed biological responses. More research is needed on whether generating and adding data to integrative analysis on the biological activity of the non-PAC fraction of petroleum substances can further improve overall grouping. Therefore, inclusion of additional cell types may be beneficial to address a broader range of potential health effects. Finally, studies on the experimental approaches that may aid in integration of biokinetic information in the grouping of complex substances are needed. For example, a previous study of bioavailability of the hydrocarbon fractions showed that extraction procedure, protein binding in cell culture media, and dilution factors prior to *in vitro* testing can all contribute to determining the bioavailable concentrations of bioactive constituents of petroleum substances (Luo et al., 2020).

In summary, we show that the use of biological activity parameters across multiple cell types of different origins, combined with extant physico-chemical properties, improves the ability to group and rank order petroleum substances for subsequent regulatory evaluation and data gap analysis. The data presented herein support the use of the current categories of petroleum substances, which are based on refining history, but add additional critical biological insights to these groupings in terms of chemical-biological activity that are important for generating read-across hypotheses.

## Supplementary Material

Supplemental Figures

Supplemental File 3

Supplemental File 2

Supplemental File 1

Supplemental Table 1

Supplemental Table 2

Supplemental Table 3

Supplemental Table 4

Supplemental Table 5

## Figures and Tables

**Tab. 1:** Petroleum substance categories and substances used in this study

Petroleum substance category	Abbreviation	N of samples in category
Petrolatums	P.LAT	3
Paraffin and hydrocarbon waxes/slack waxes	WAX	10
Low boiling point naphthas (gasolines)	NAPHTHA	10
Other lubricant base oils/highly refined base oils	BO	33
Kerosines/MK1 diesel fuel	KER	10
Foots oils	FO	3
Other gas oils	OGO	4
Bitumens/oxidized asphalt	BIT	5
Residual aromatic extracts	RAE	2
Treated distillate aromatic extracts	TDAE	2
Heavy fuel oil components	HFO	27
Unrefined/acid treated oils	UATO	4
Cracked gas oils	CGO	8
Vacuum gas oils, hydrocracked gas oils & distillate fuels	VHGO	10
Straight-run gas oils	SRGO	6
Untreated distillate aromatic extracts	UDAE	4

See Table S1^1^ for a complete listing of substance names, CAS and EC numbers and other information.

**Tab. 2:** Cell types used in these studies

Organ/Tissue	Origin	Cell type name	Number of phenotypes	QC “Pass” phenotypes	QC “Fail” phenotypes
Skin	Malignant melanoma	A375	3	2	1
Lung	Epithelial carcinoma	A549	3	0	3
Liver	Cholangiosarcoma	HEPARG	3	1	2
Liver	Hepatocellular carcinoma	HEPG2	3	2	1
Lung	Microvascular endothelial cells	HLMVEC	4	2	2
Gut	Colorectal adenocarcinoma	HT29	4	0	4
Brain	Glioblastoma	LN229	4	2	2
Breast	Epithelial adenocarcinoma	MCF7	3	2	1
Bone marrow	Neuroblastoma	SH-SY5Y	4	2	2
Heart	iPSC-derived cardiomyocytes	CM	14	12	2
Liver	iPSC-derived hepatocytes	HEP	6	4	2
Blood vessel	iPSC-derived endothelial cells	ENDO	9	4	5
Blood vessel	Umbilical cord endothelial cells	HUVEC	6	5	1
Brain	iPSC-derived neuronal cells	NEUR	4	4	0
Blood	iPSC-derived macrophages	MACRO	1	0	1
			**71**	**42**	**29**

See Table S3^1^ for a complete list of assays, phenotypes, time points, and quality control criteria.

**Tab. 3:** Spearman (rank) correlations of PAC content summarized across sets of ring classes suggests that the summary of PAC 3–7 rings is most predictive of overall bioactivity

PAC content	Correlation with ToxPi bioactivity
Rings 3–7	0.89
Rings 4–7	0.70
Rings 5–7	0.51
Rings 1–2	0.36
3 Ring	0.84
4 Ring	0.73
5 Ring	0.55
6 Ring	0.43
7 Ring	0.29

The greatest individual contributions arise from 3 ring and 4 ring content.

**Tab. 4:** Cell-specific relationships between the bioactivity and polycyclic aromatic compound (PAC, 3–7 ring) content of petroleum UVCBs tested in this study

Cell type name	Spearman correlation (ρ) with PAC (3–7 ring)
A375	0.12
A549	n/a*
HEPARG	0.18
HEPG2	0.25
HLMVEC	0.52
HT29	n/a
LN229	0.10
MCF7	0.52
SH-SY5Y	−0.09
CM	0.83
HEP	0.81
ENDO	0.82
HUVEC	0.76
NEUR	0.74
MACRO	n/a
All QC “pass” phenotypes	0.89

See Figure S5^1^ for cell type-specific correlation plots.

* No QC “pass” phenotypes were available from this cell type. See Table 2 for explanation.

## Footnotes

1 doi:10.14573/altex.2006262s1
