# Supplementary material for: Grouping of UVCB Substances with New Approach Methodologies (NAMs) Data
Source: ALTEX. Author manuscript; Available in PMC 2021 Feb 23. (PMC7900923; doi:10.14573/altex.2006262)

**Supplemental Figure 1.** iPSC-derived cell types (including FujiFilm-CDI [Madison, WI] catalogue numbers), positive and vehicle controls that were used for each cell type. See Supplemental File 1 for details on each cell type and chemical listed herein.


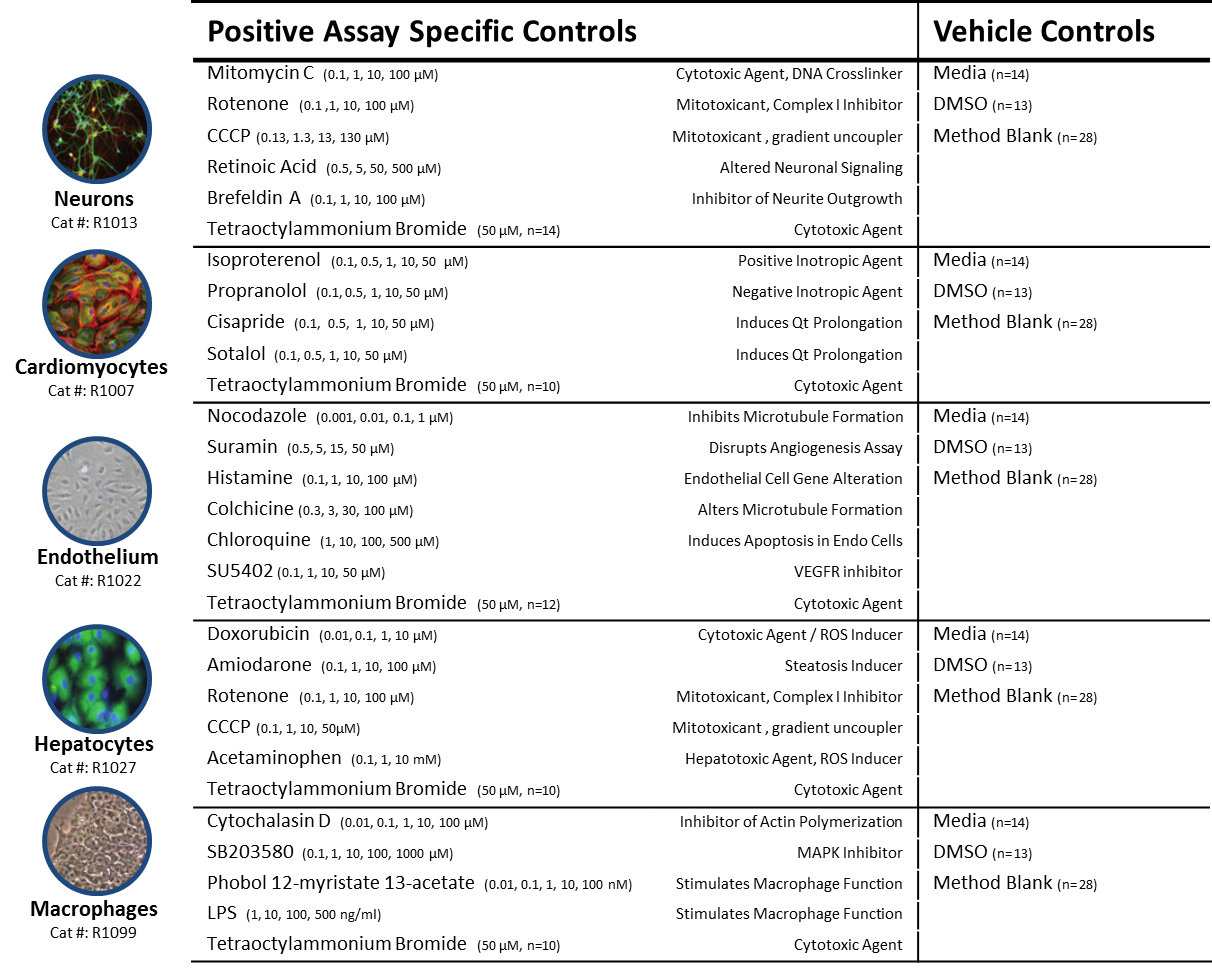


**Supplemental Figure 2.** Schematic diagram for preparation of serial dilutions for petroleum substance extracts from a master plate for subsequent use in bioactivity profiling experiments.


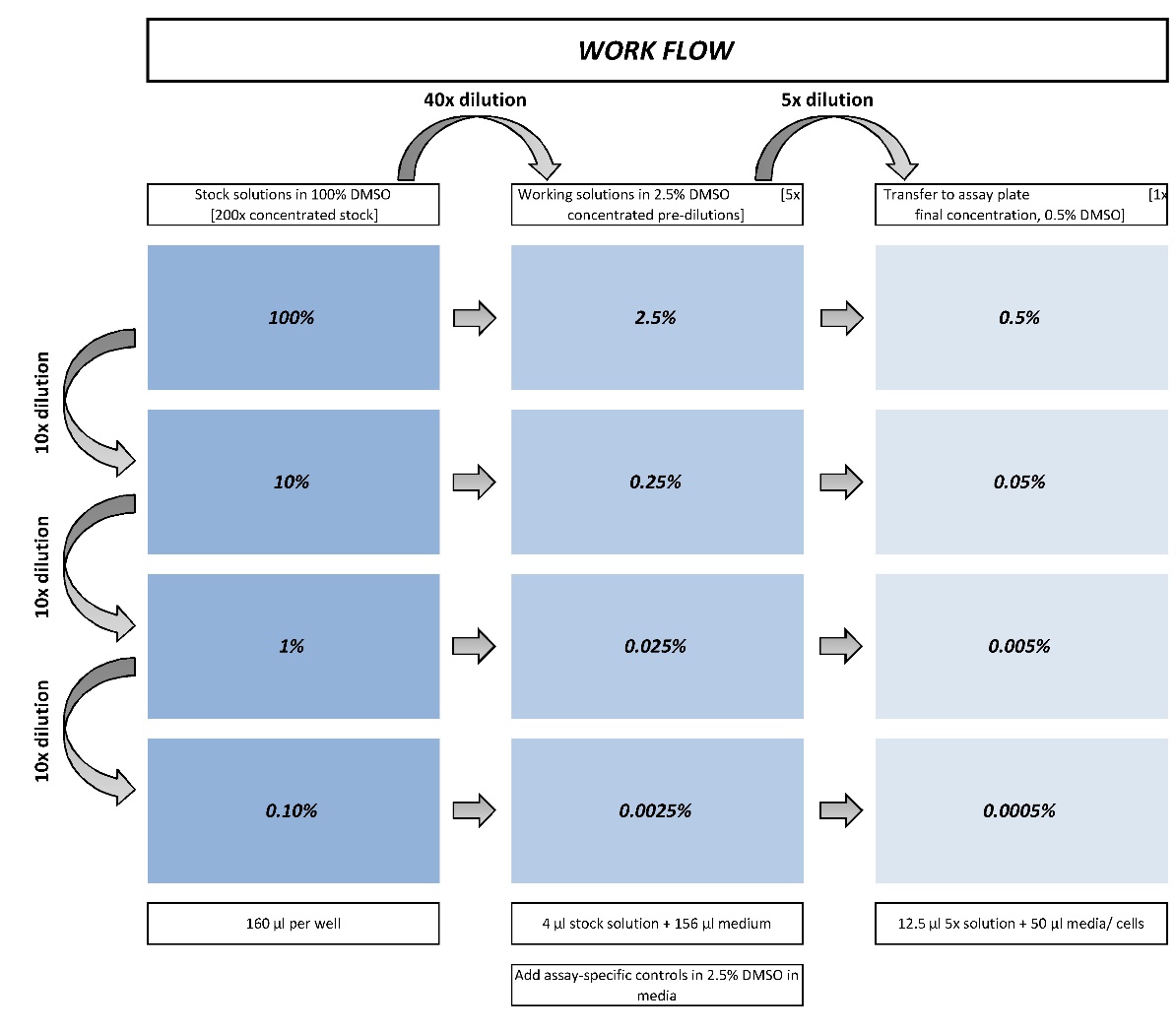


**Supplemental Figure 3.** Decision-tree for derivation of the point of departure (POD) values from *in vitro* data. See Supplemental File 3 for the R scripts used in these calculations.


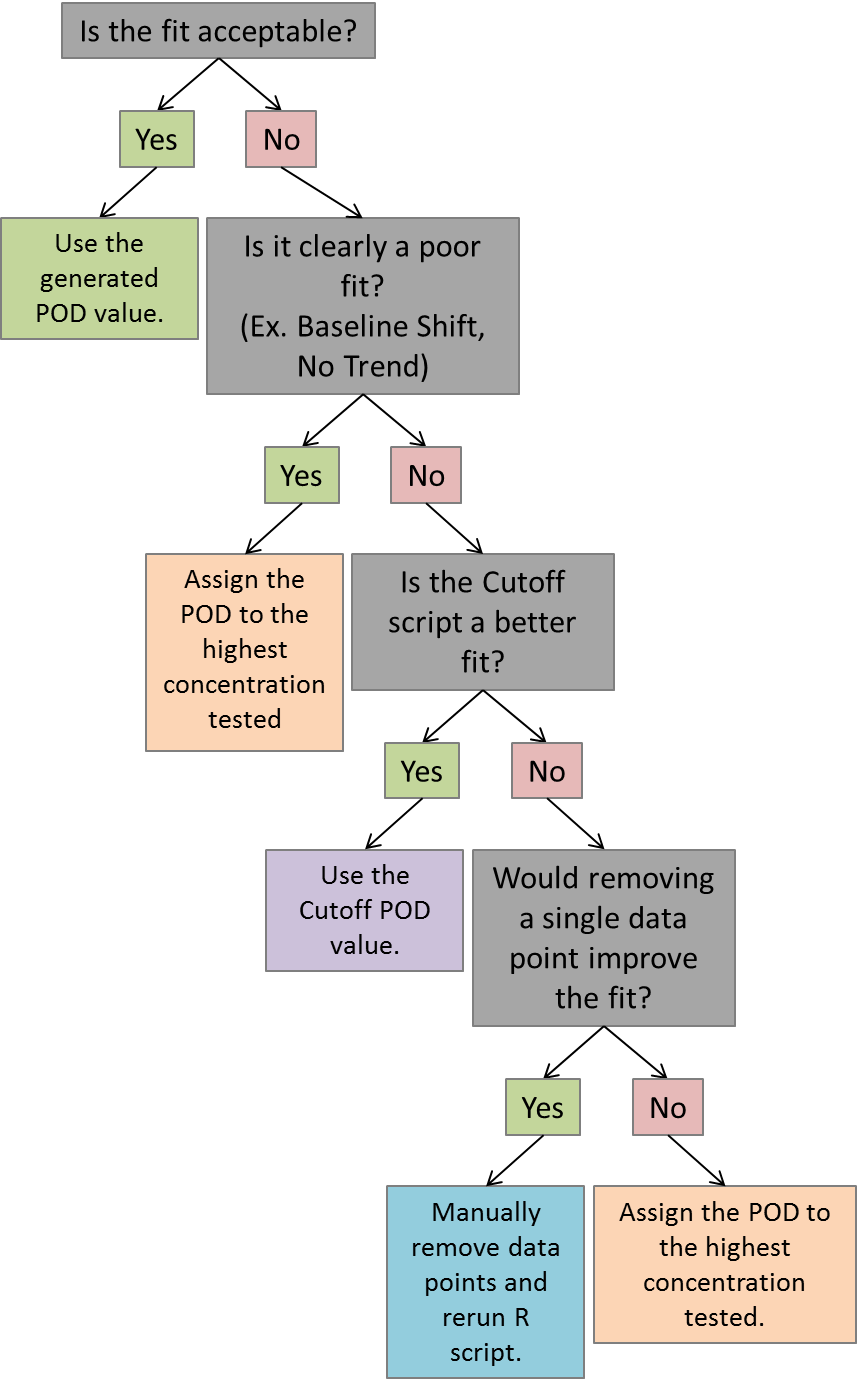


**Supplemental Figure 4.** Manufacturing stream-based grouping of the bioactivity for individual petroleum-derived UVCBs using data on the individual cell types not shown in Figure 6. Cell type abbreviations are defined in Table 2. Each dot represents a UVCB sample total ToxPi score derived from all phenotypes (top) or cell-specific phenotypes. Box is the inter-quartile range, vertical line is the median, and whiskers are min-max range of values. X-axis in all plots is the ToxPi score for each cell type.

A. A375 cells.


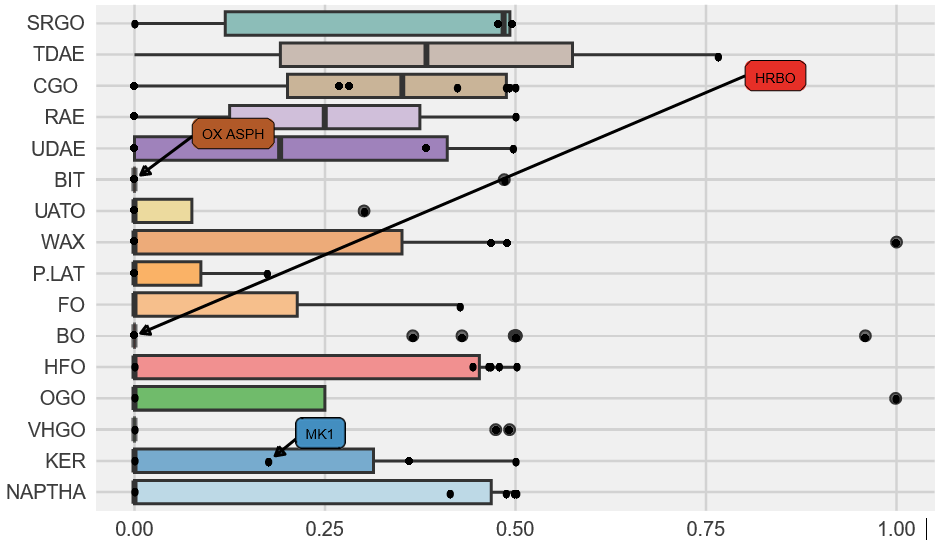


B. ENDO cells.


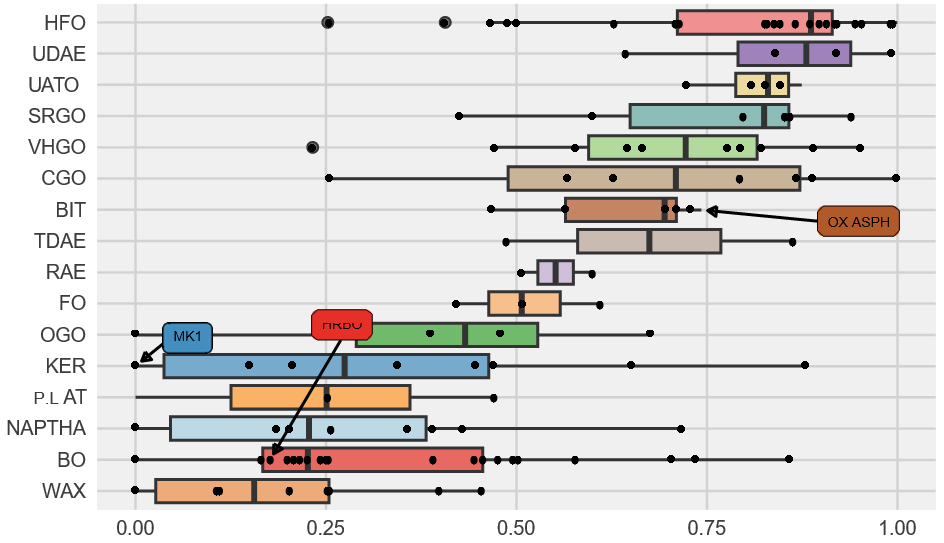


C. HEPARG cells.


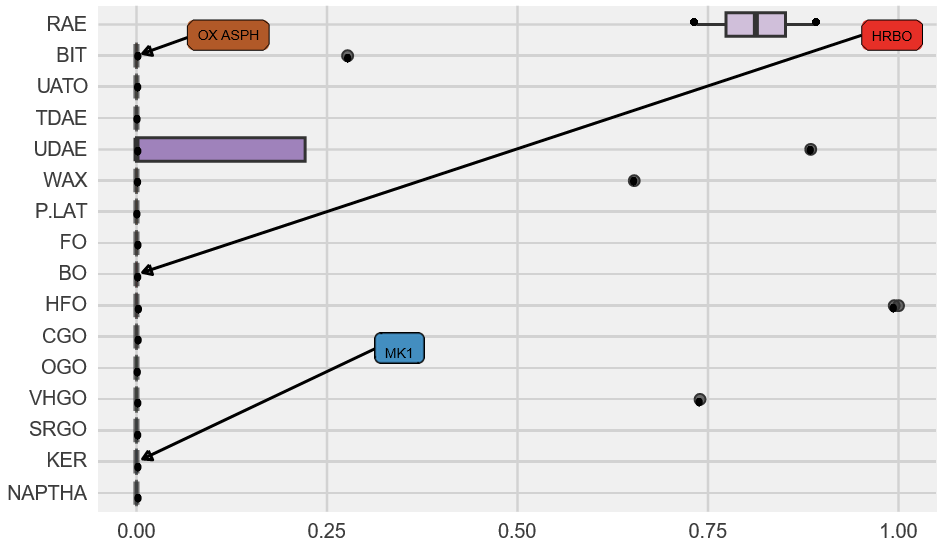


D. HepG2 cells.


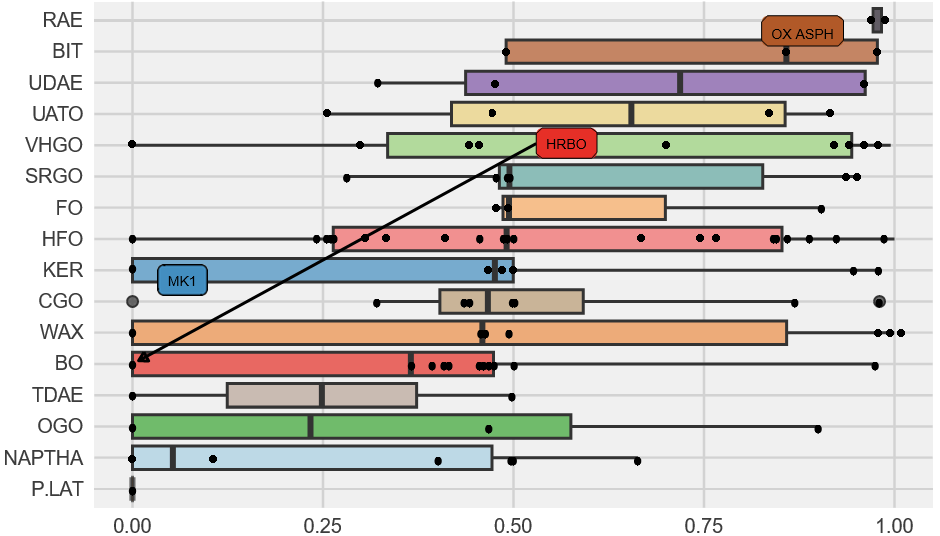


E. HLMVEC cells.


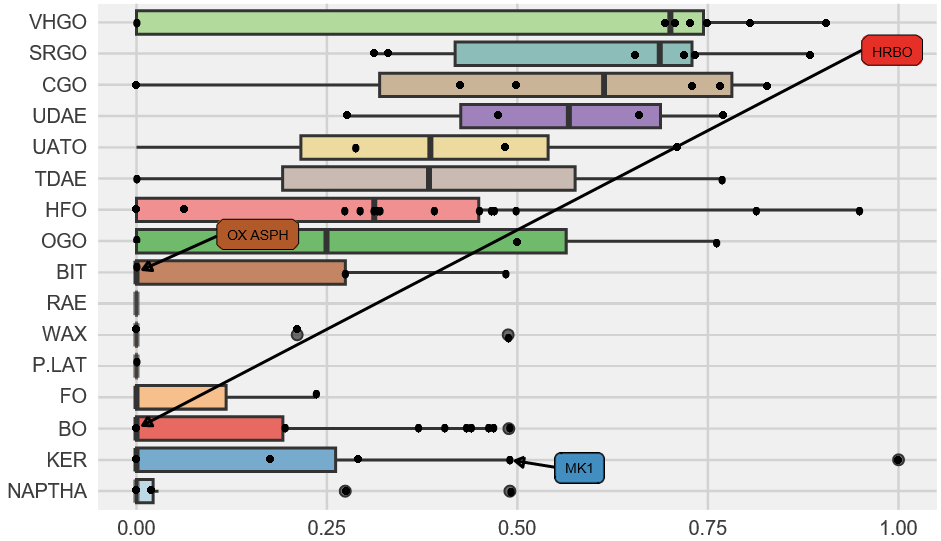


F. HUVEC cells.


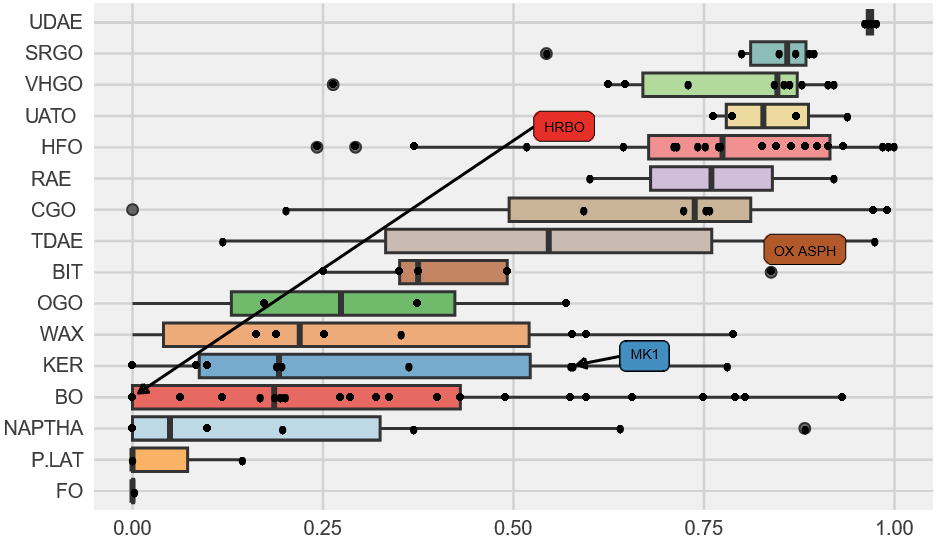


G. LN229 cells.


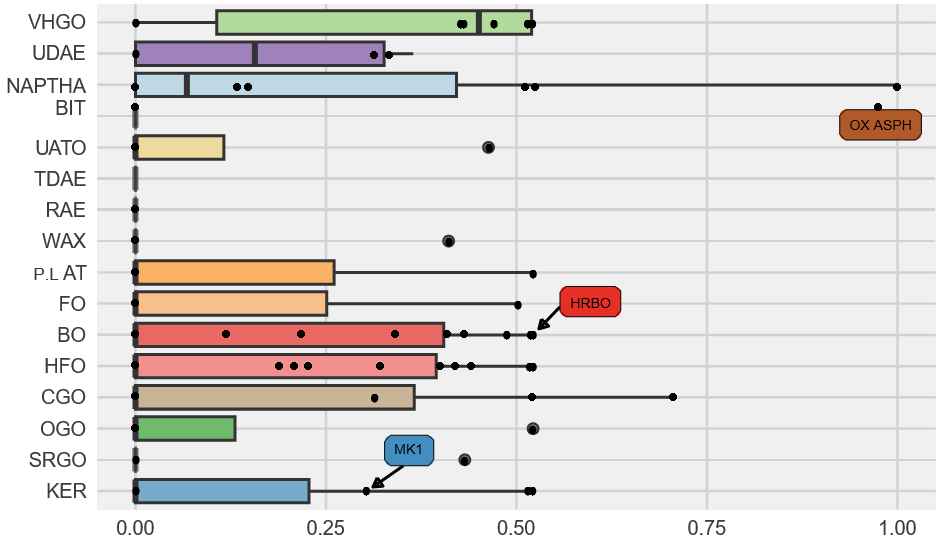


H. MCF7 cells.


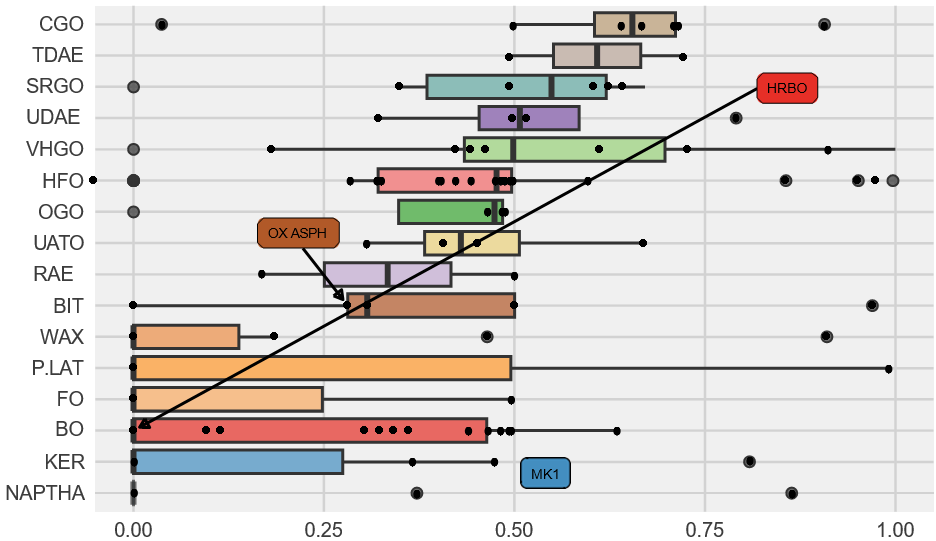


I. NEUR cells.


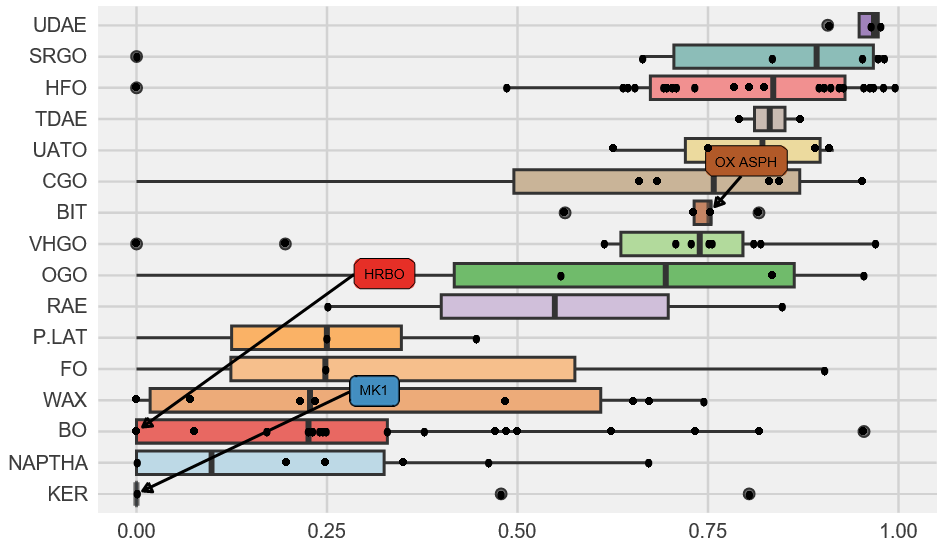


J. SH-SY5Y cells.


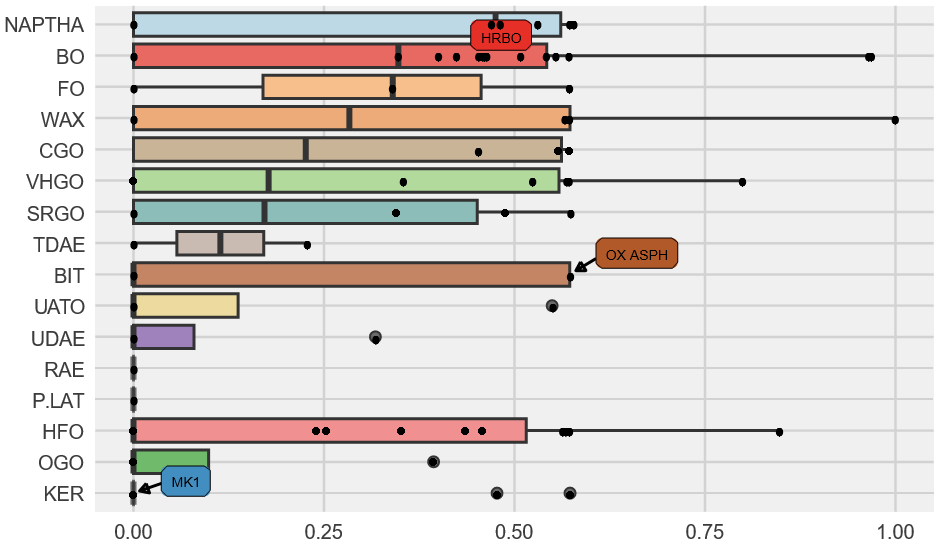


**Supplemental Figure 5.** Correlation of polycyclic aromatic compound (PAC) score for 3-7 ring compounds in each UVCB sample with cell-specific ToxPi bioactivity scores. Spearman Rho correlation is shown on each plot.

A. A375 cells.


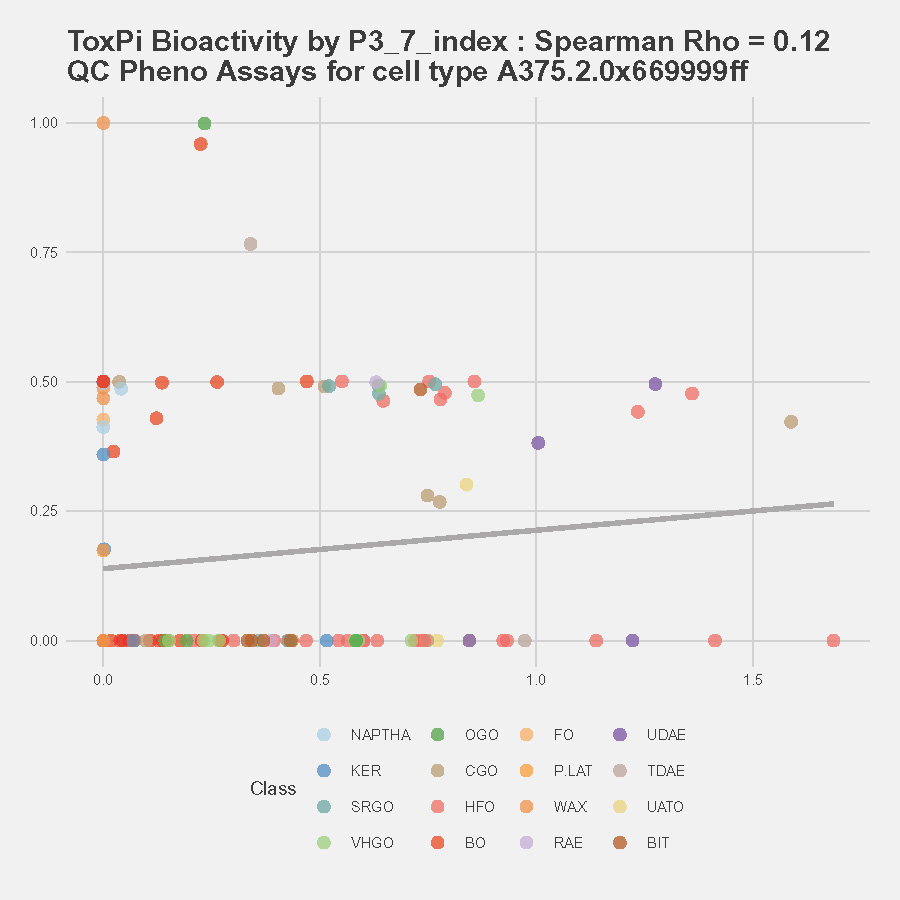

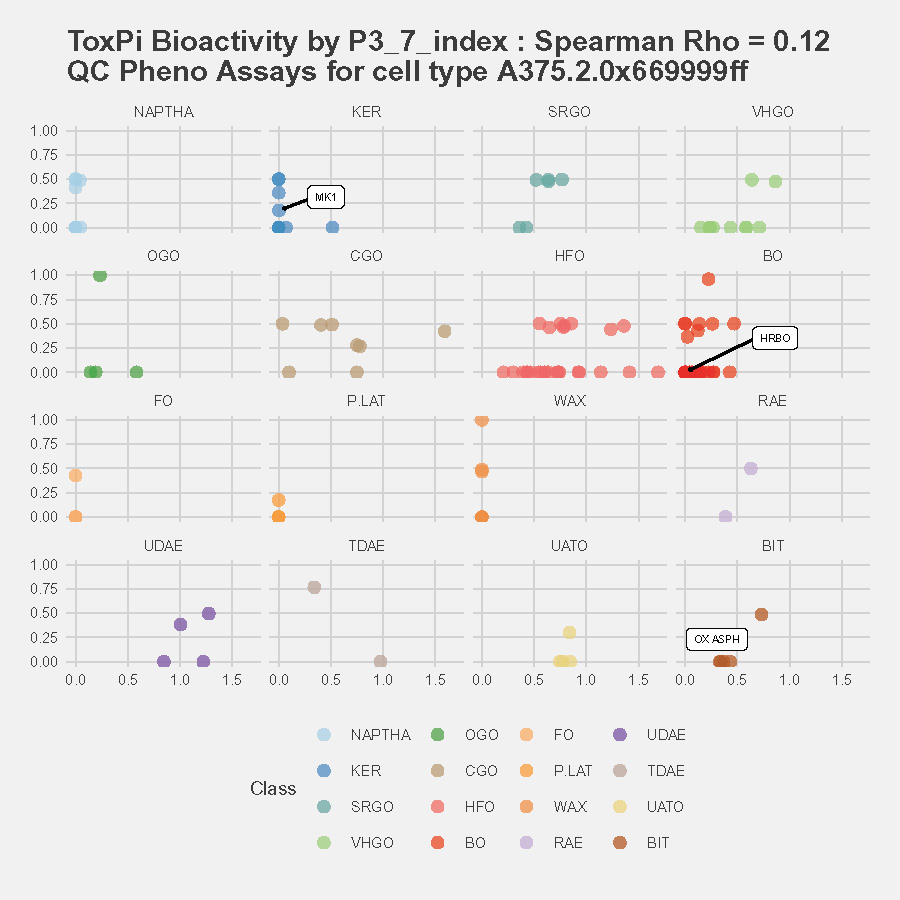


B. CM cells.


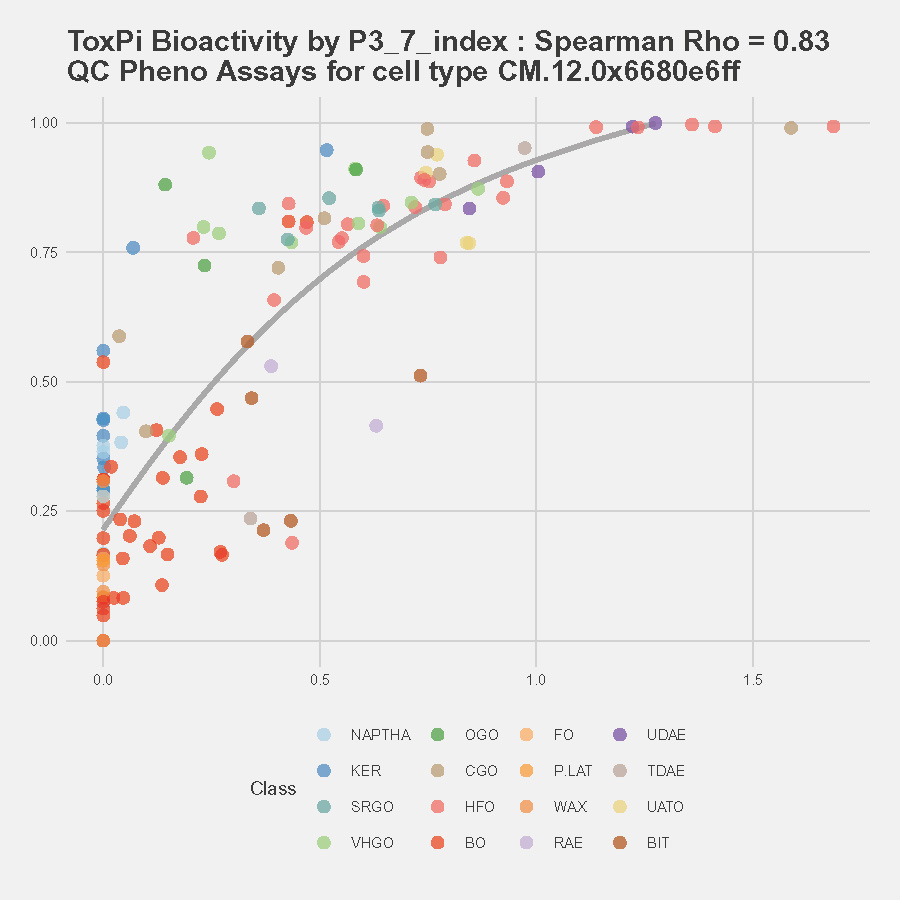


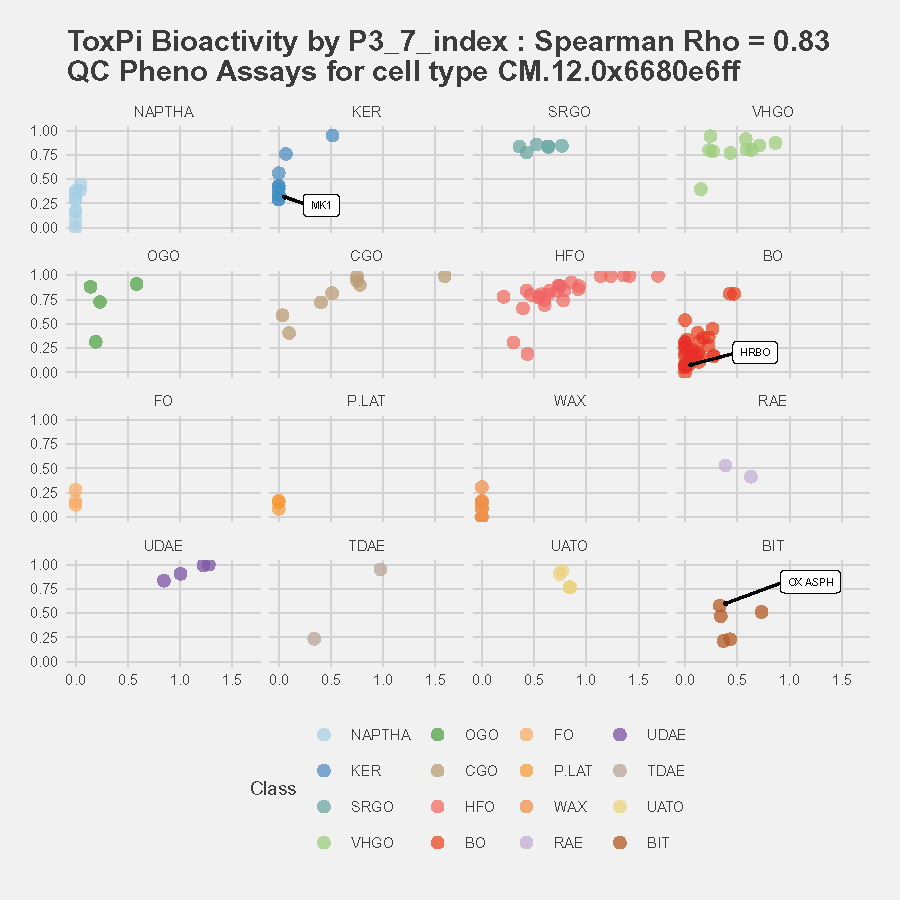


C. ENDO cells.


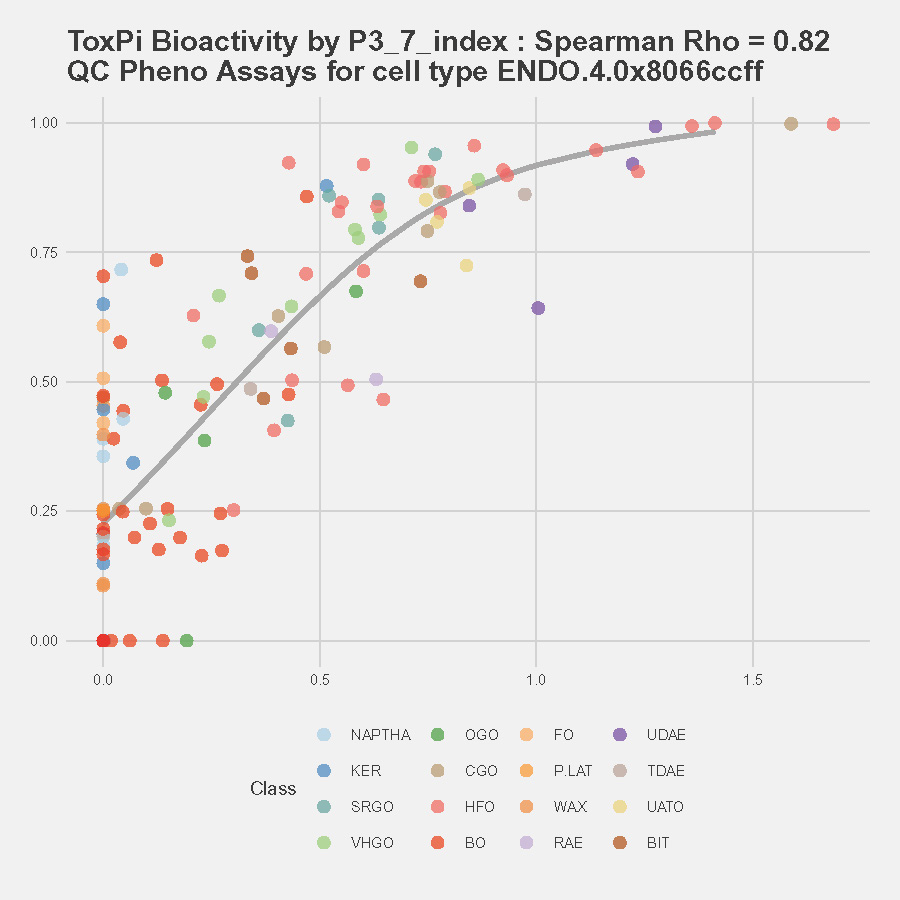

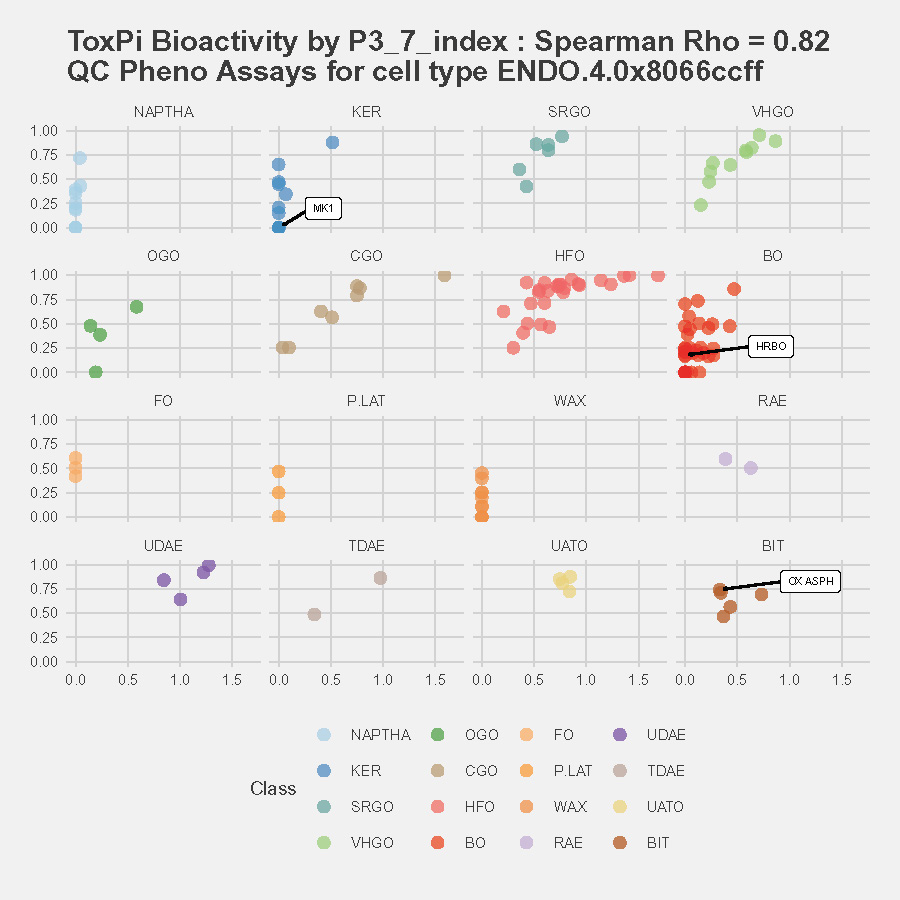


D. HEP cells.


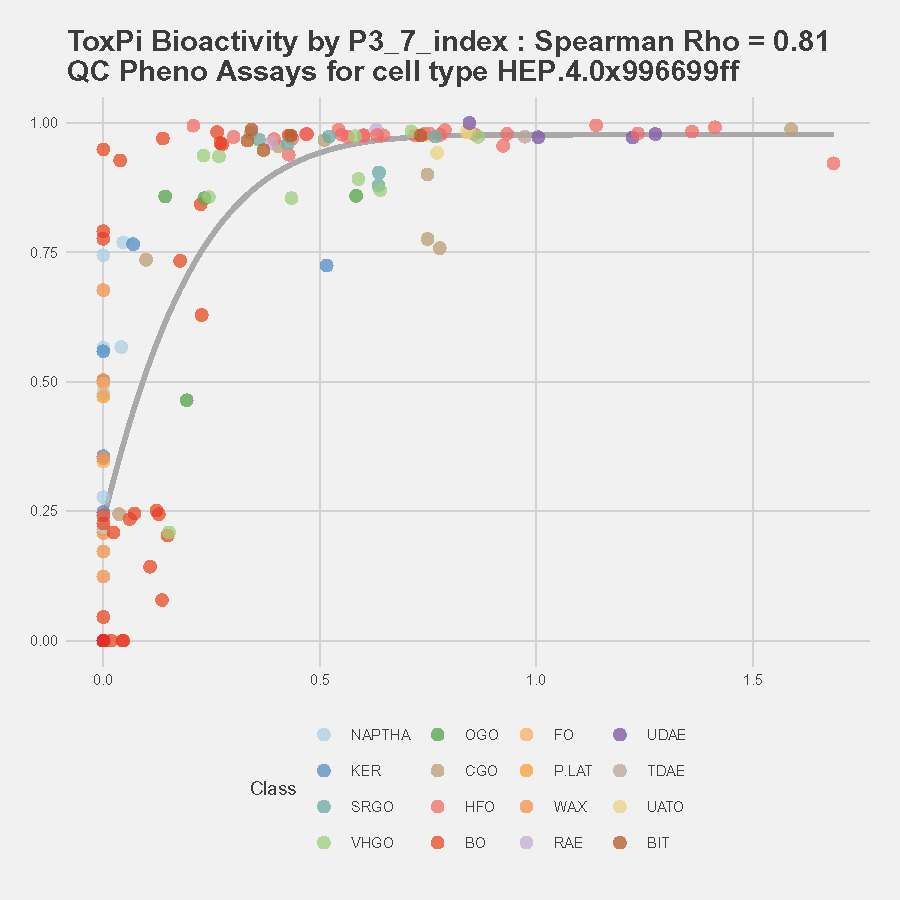

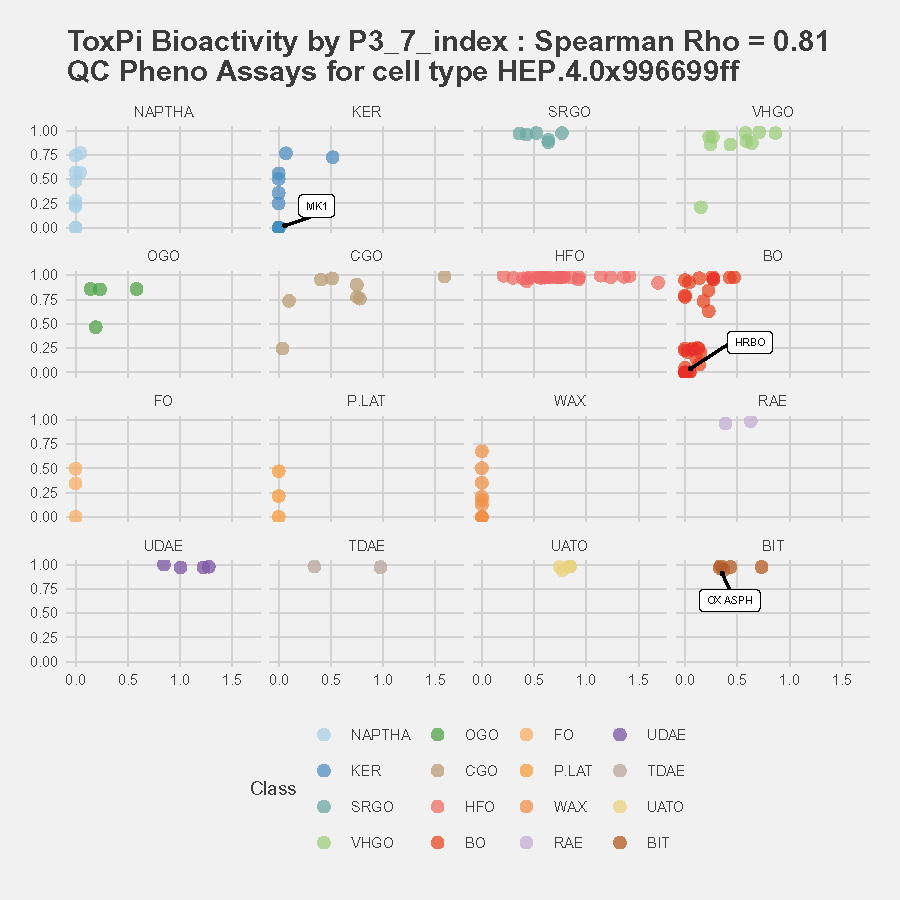


E. HEPARG cells.


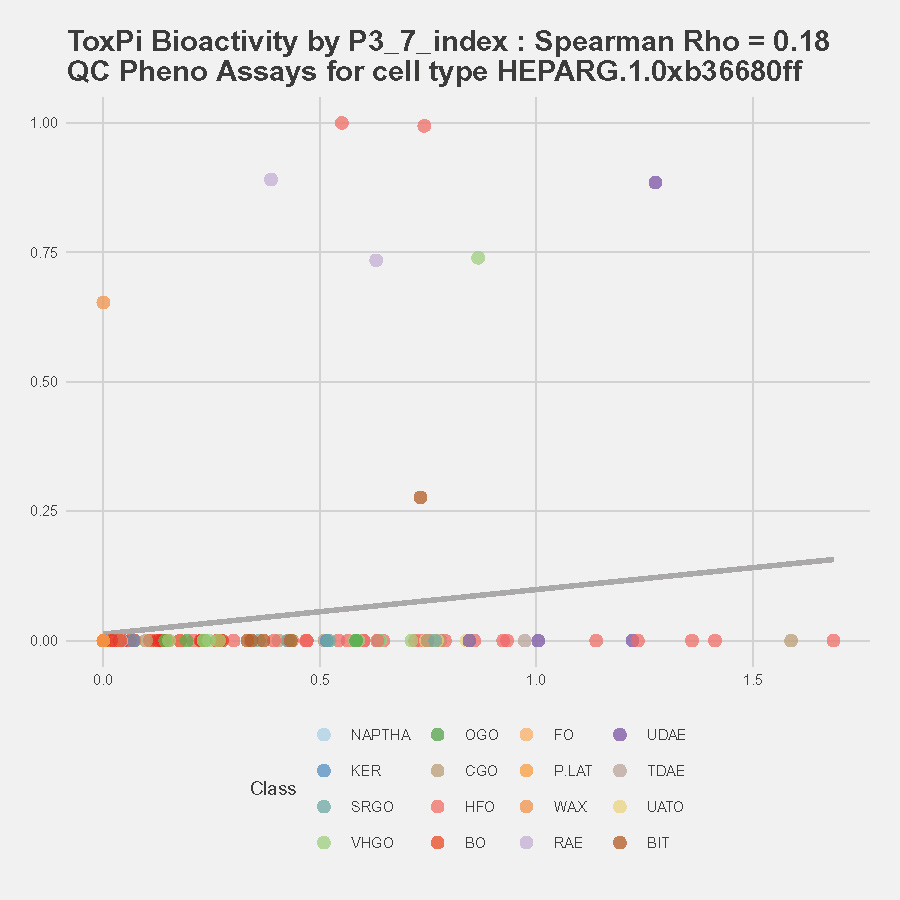

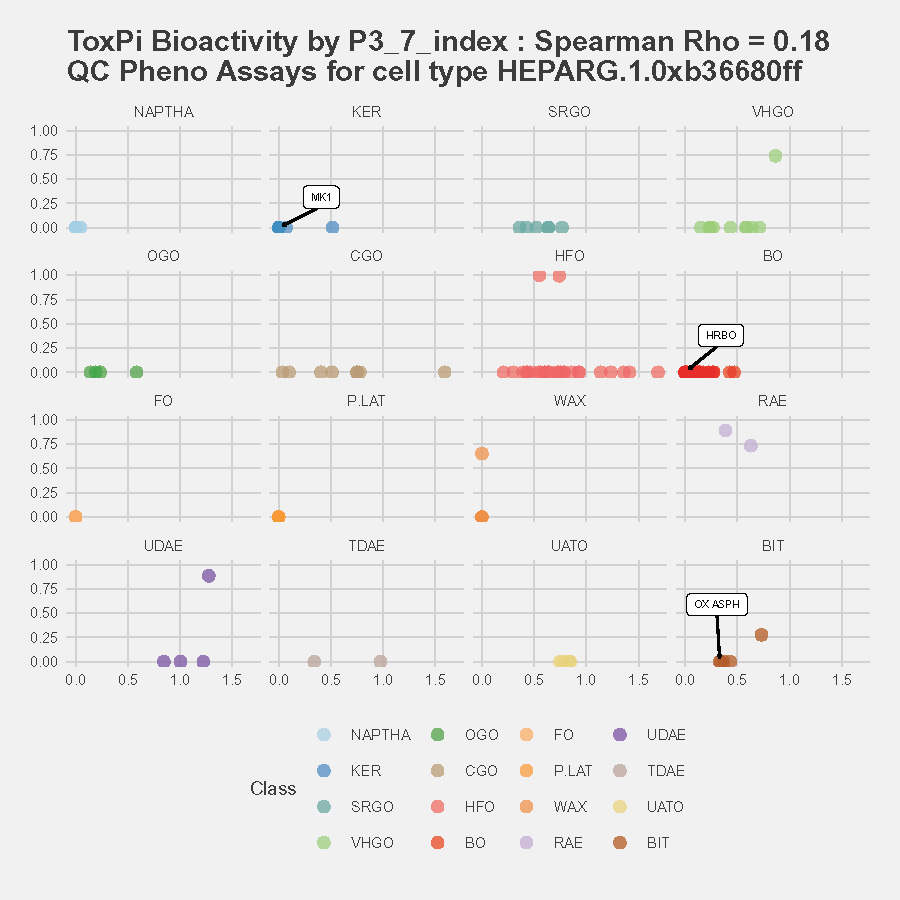


F. HepG2 cells.


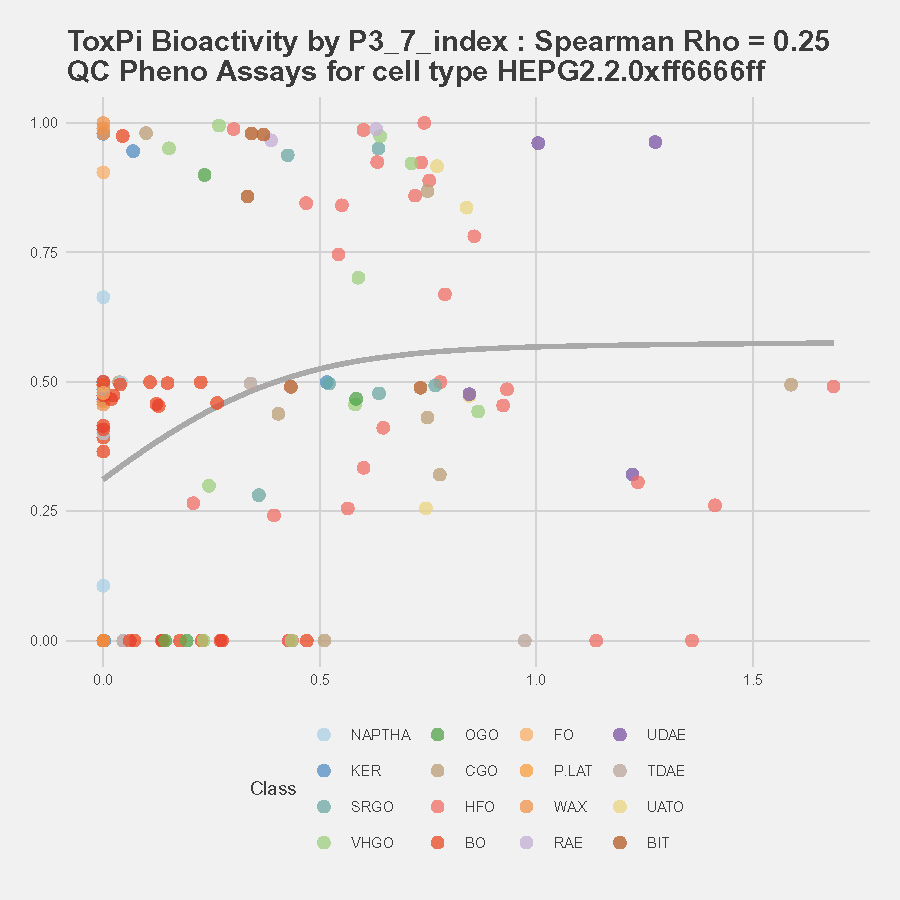

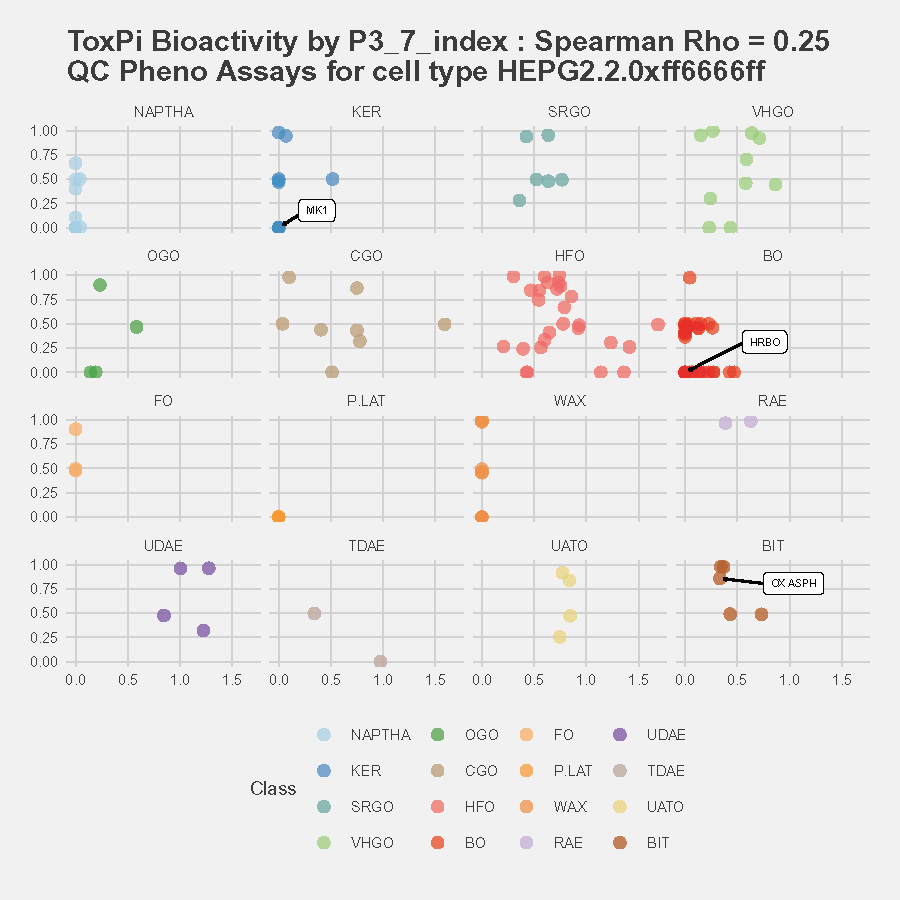


G. HLMVEC cells.


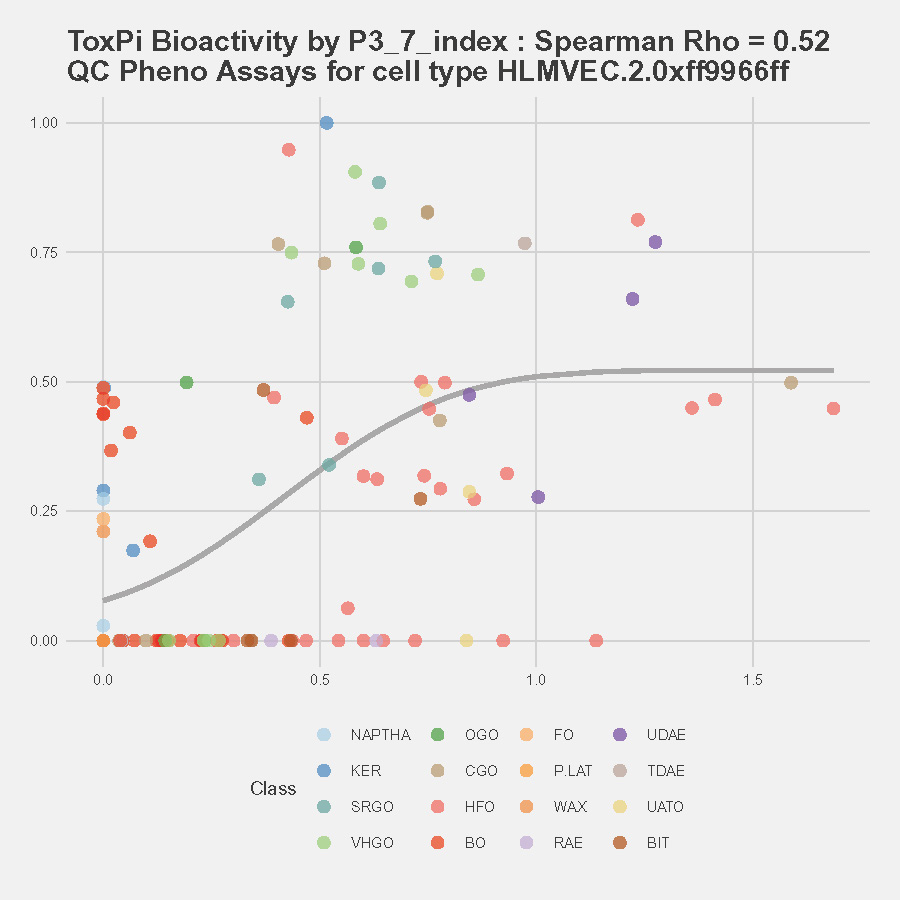

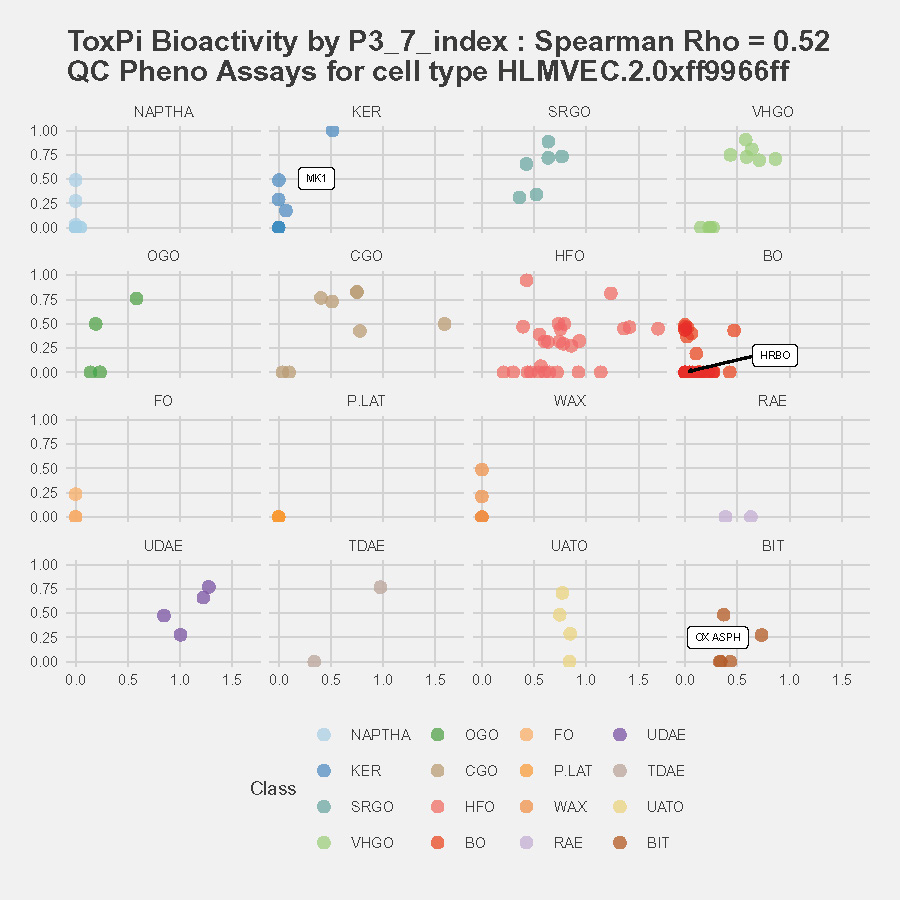


H. HUVEC cells.


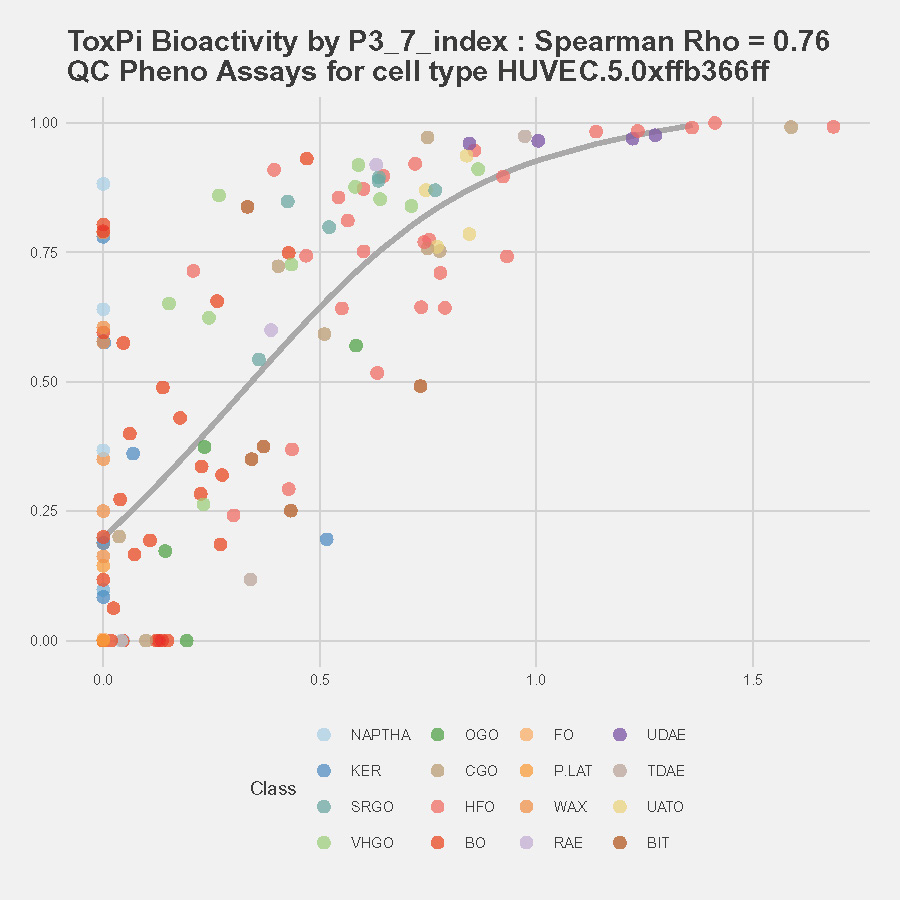

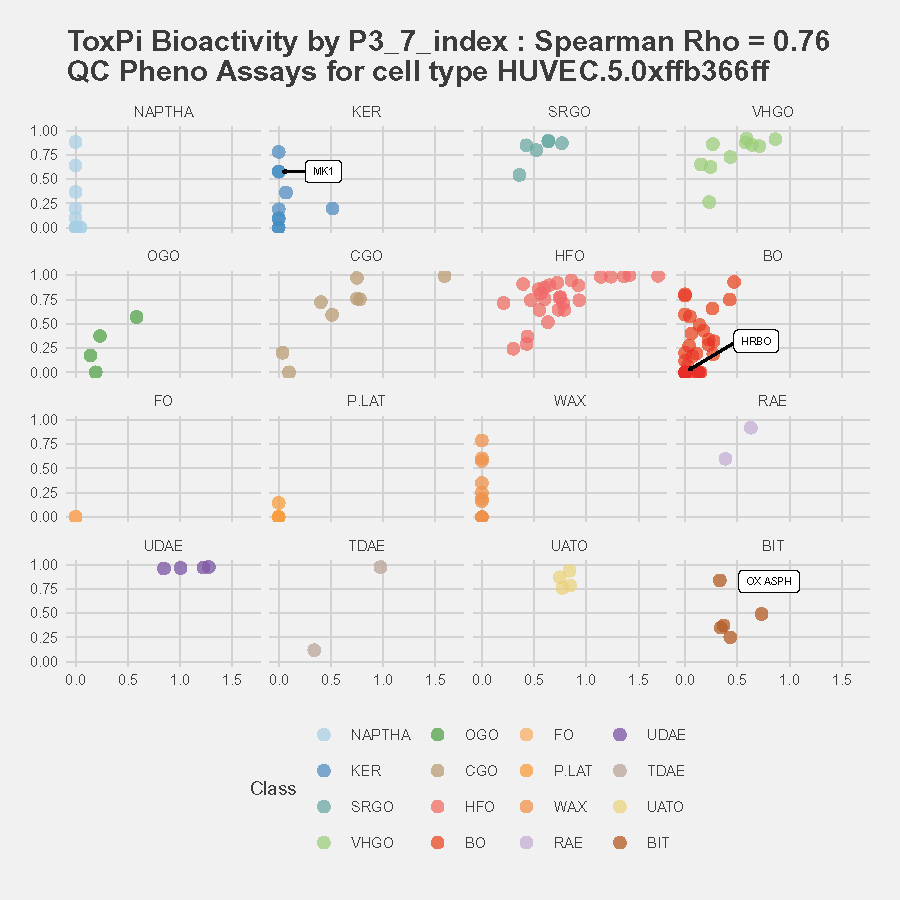


I. LN229 cells.


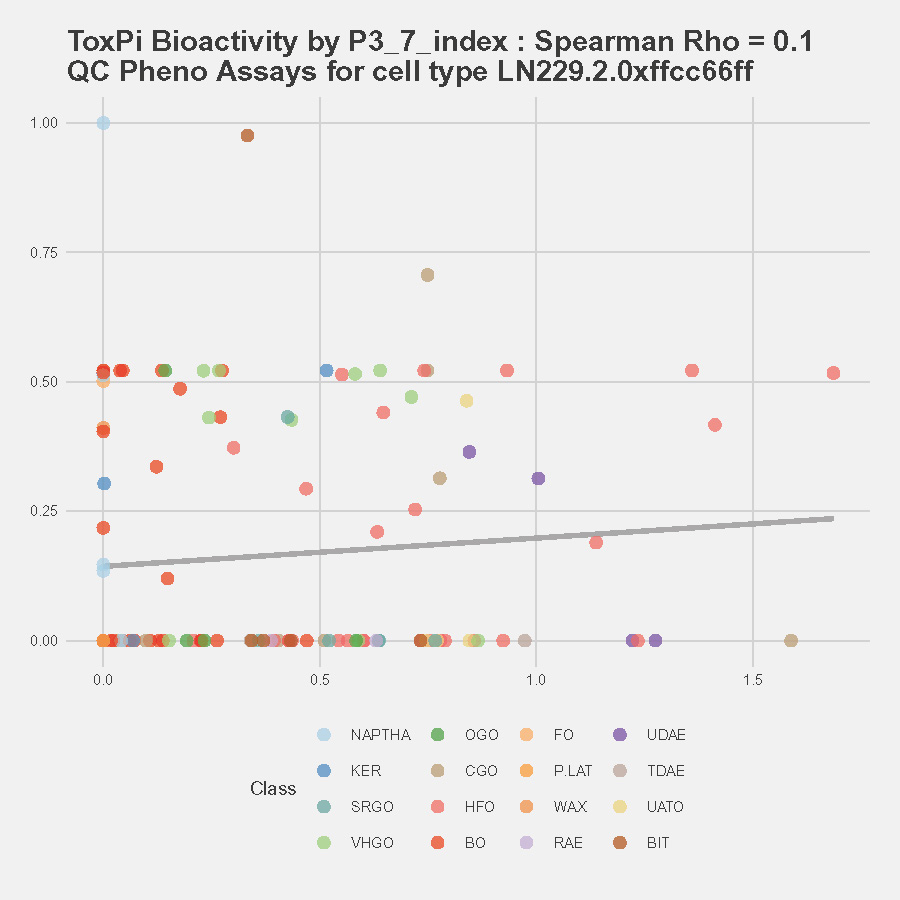

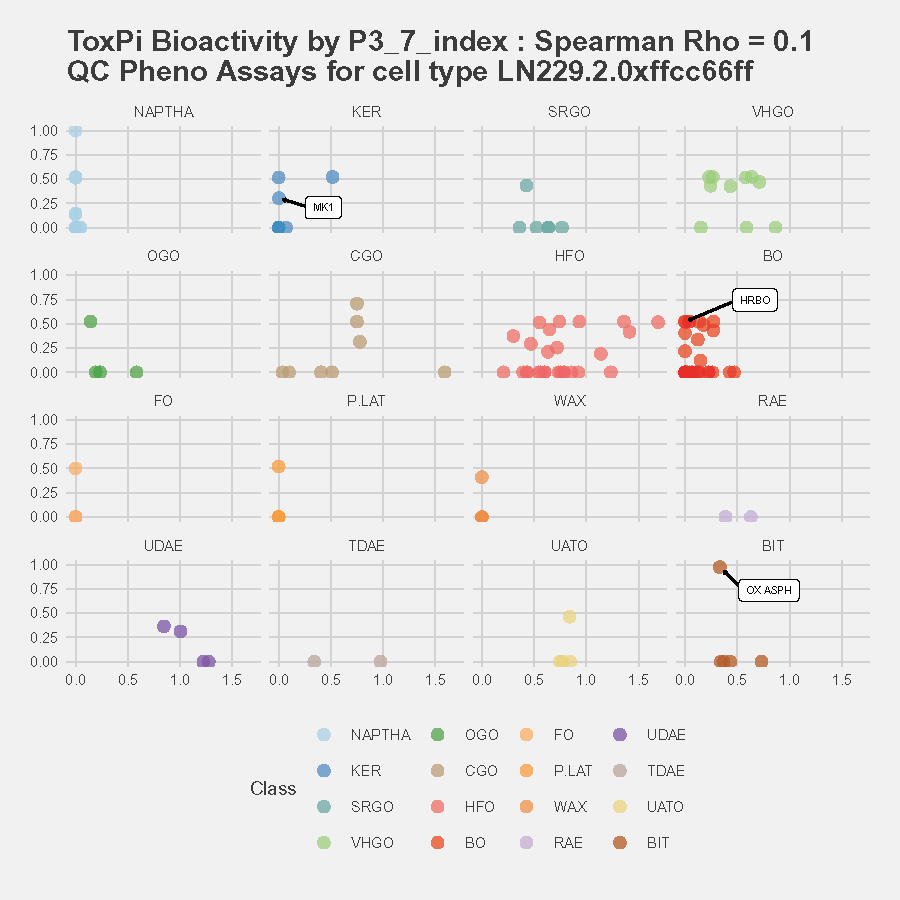


J. MCF7 cells.


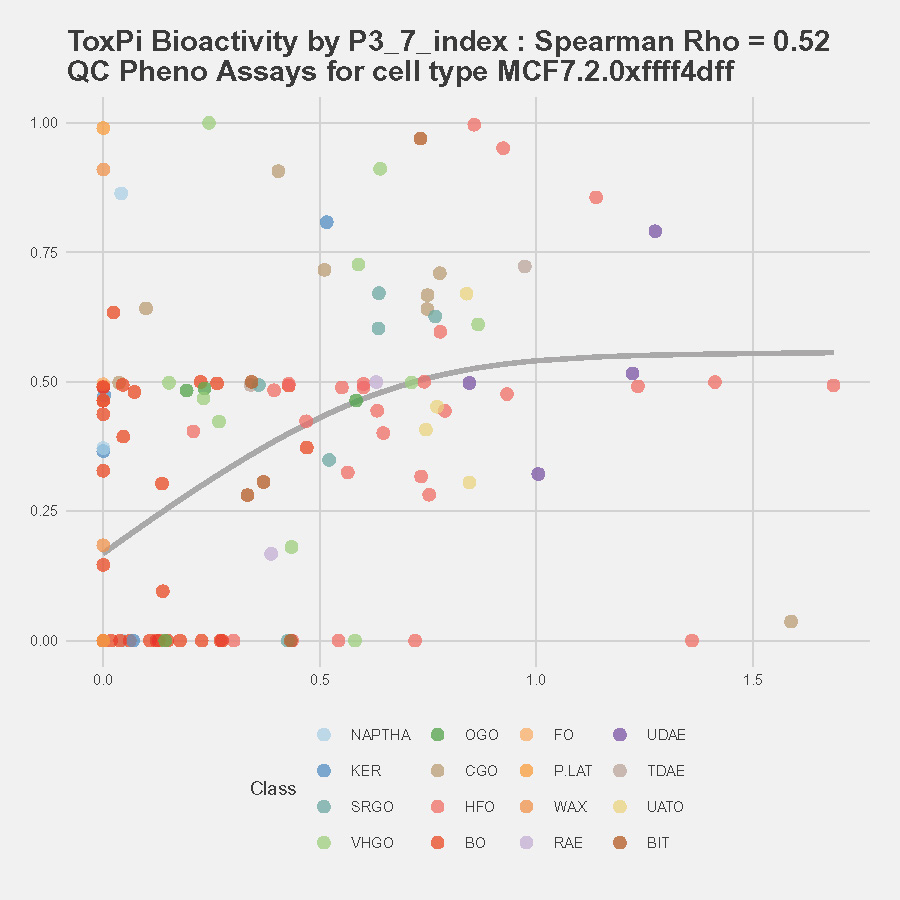

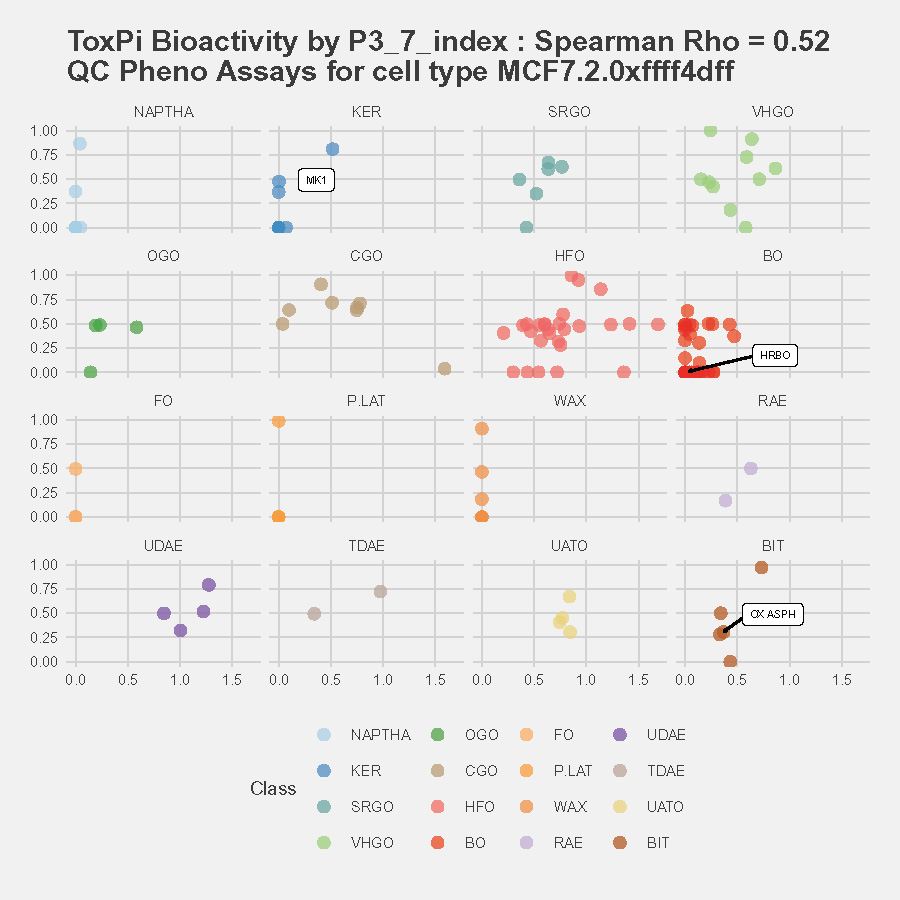


K. NEUR cells.


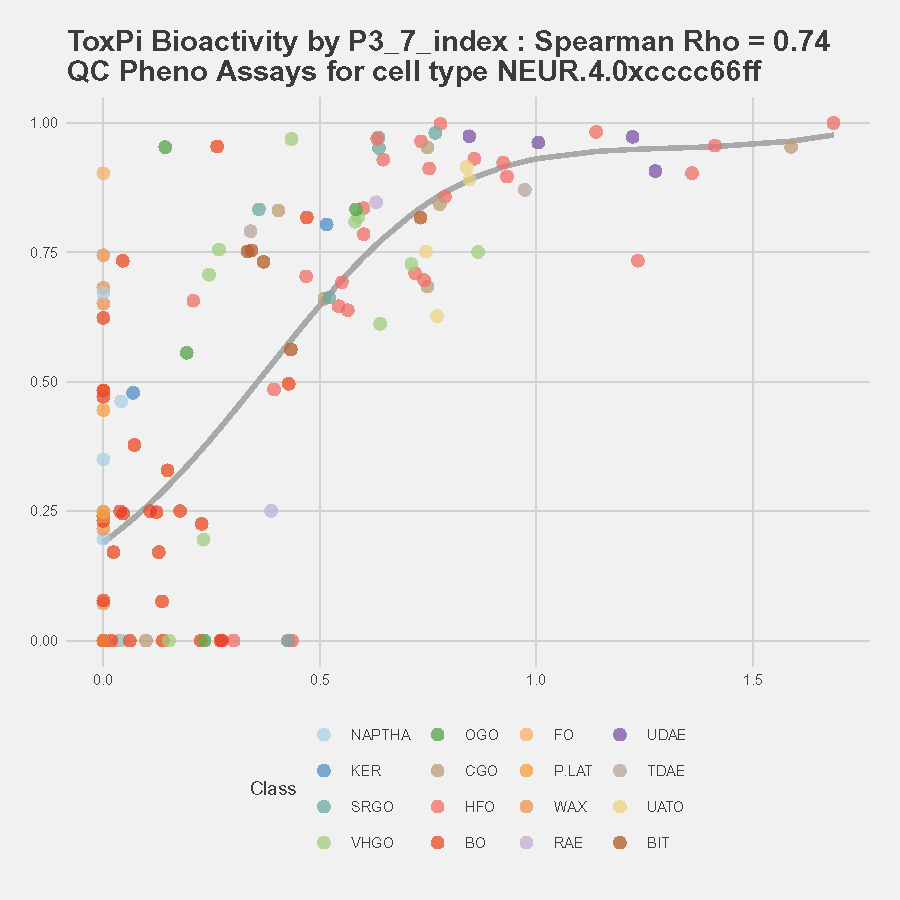

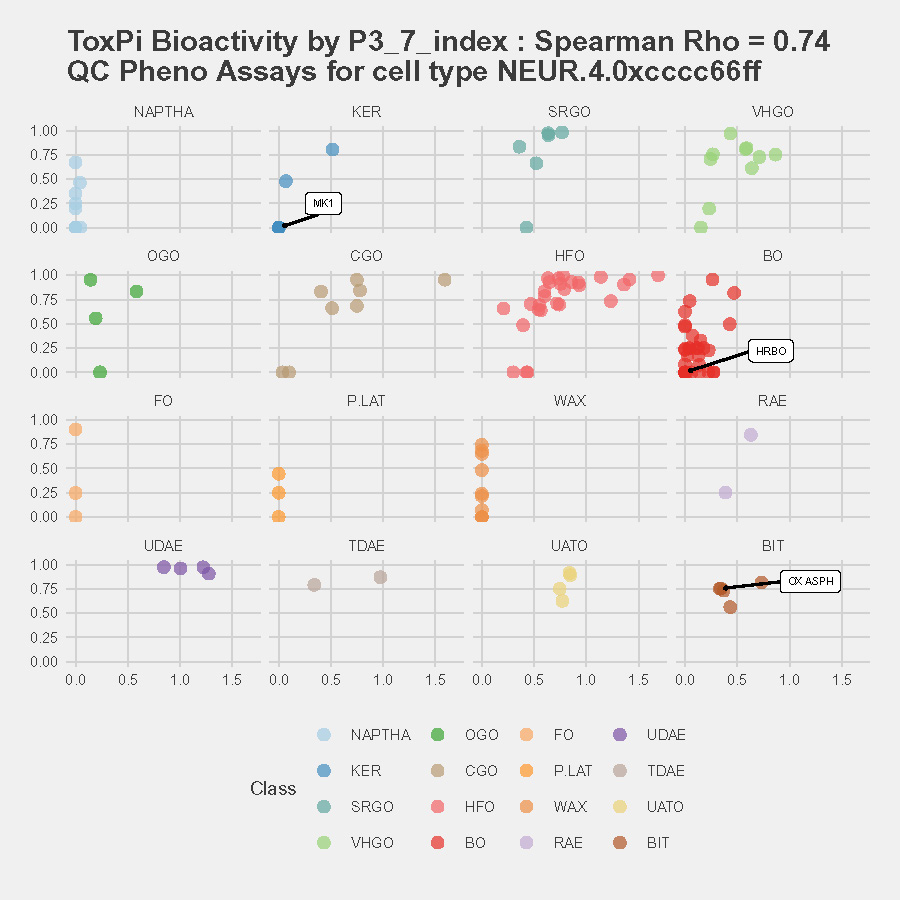


L. SH-SY5Y cells.


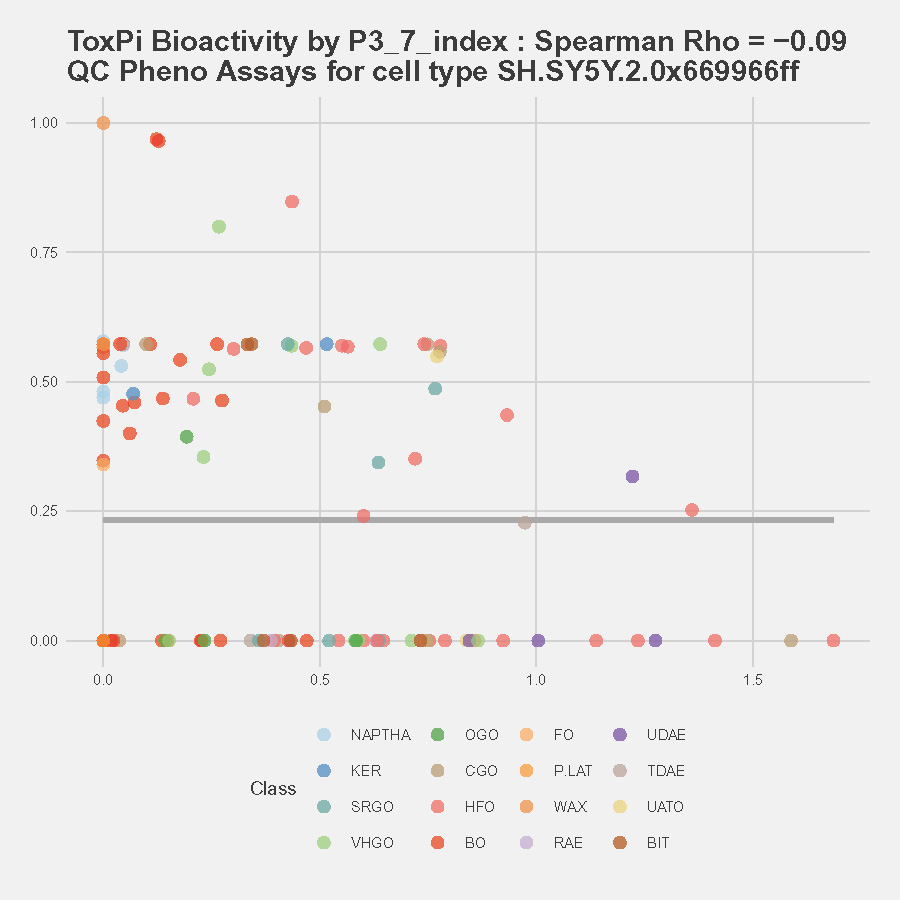

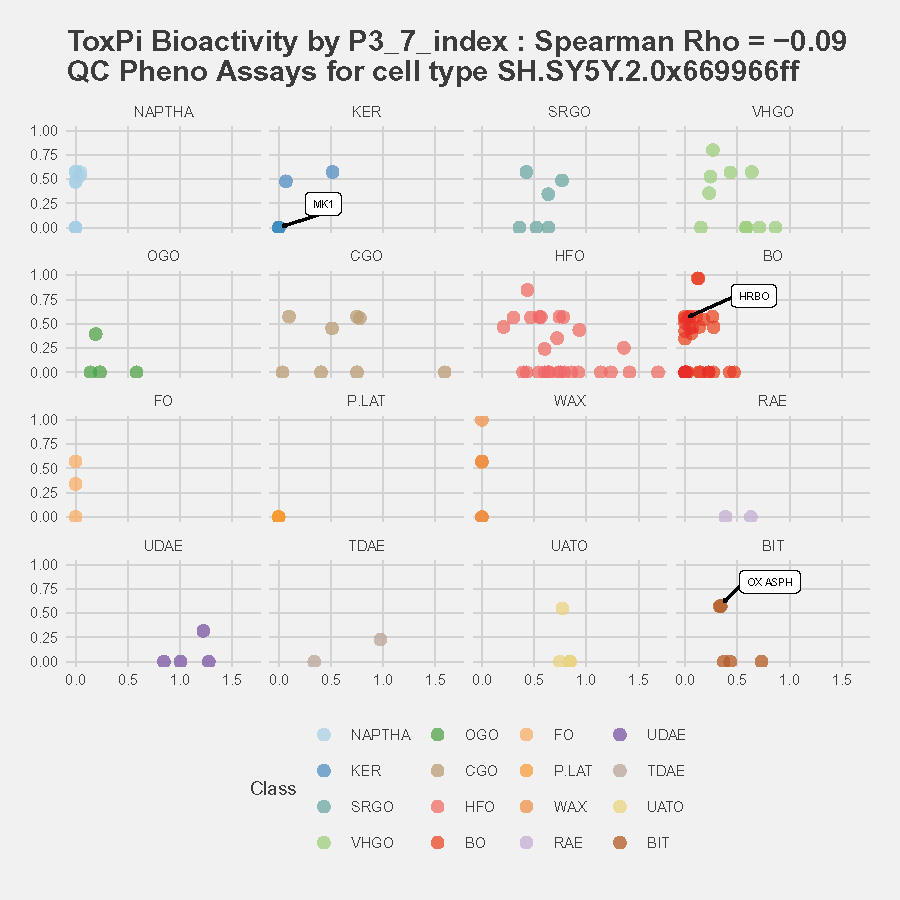

Supplement: Supplemental Figures [file NIHMS1670764-supplement-Supplemental_Figures.docx]
