# Supplementary material for: Grouping of UVCB Substances with New Approach Methodologies (NAMs) Data
Source: ALTEX. Author manuscript; Available in PMC 2021 Feb 23. (PMC7900923; doi:10.14573/altex.2006262)
Supplement: Supplemental File 3 [file NIHMS1670764-supplement-Supplemental_File_3.pdf]

Supplemental File 3. R scripts that were used for derivation of the point-of-departure (POD) values from in vitro data.

Data import and export code:

```
setwd("Z:\\")
source("fitfunctions.txt")

install.packages("pROC")
library(pROC)

w2=read.csv("Data_PS.csv")
dmso=read.csv("Vehicle.csv")
rowblanks=grep(0,nchar(as.vector(dmso[,1])))
if (length(rowblanks)>0){dmso=dmso[-rowblanks,]}
rowblanks=grep(T,nchar(as.vector(w2[,1]))==0)
if (length(rowblanks)>0){w2=w2[-rowblanks,]}

controls=as.vector(unlist(dmso[,2])) # the 9:14 may change
hist(controls)

conc=c(rep(0.1,length(controls)),rep(1,1),rep(10,1),rep(100,1),rep(1000,1))

startcol=2
endcol=5

x=log10(conc)
numchem=dim(w2)[1]
w2pod=matrix(NA,dim(w2)[1],3)
w2podupper=matrix(NA,dim(w2)[1],3)
EC=matrix(NA,dim(w2)[1],3)
direction=rep(NA,dim(w2)[1])
mychem=w2$Chemical_name

#for (i in (1:numchem)){
pdf("Data_PS.pdf",height=5,width=5)
for (i in (1:numchem)){
 noncontrols=unlist(w2[i,startcol:endcol])
 if (sd(noncontrols)>0){
 y=c(controls,noncontrols)
 #polyfit(x,y,plot.it=T)
 #title(mytitle)
 #locator(1)
 mytitle=paste(as.character(w2[i,1]),i)
 result=together.logit4(x,y,plot.it=T,threshold=-25)
 w2pod[i,]=result$bmd
 w2podupper[i,]=result$bmdupper
 EC[i,]=result$EC
 direction[i]=result$direction
 title(mytitle)
 #locator(1)
 }
 print(i)
 }
dev.off()
```

```
######### AUC NOT CHECKED BELOW!!! 2/18/15
########## Now look at activity vs. POD
par(mfrow=c(3,4))
active=1-w2$Control
final.auc=rep(0,12)
for (j in (1:12)){
 currentroc= roc(active,w2pod[,j],auc=T,plot=T)
 title(round(currentroc$auc,4))
 final.auc[j]=currentroc$auc
 }
###############
###############

## Now output results
colnames(w2pod)=paste("pod.sd",1:3,sep="")
colnames(w2podupper)=paste("pod.sd.upper",1:3,sep="")
colnames(EC)=c("EC10","EC50","EC90")
rownames(w2pod)=mychem
rownames(w2podupper)=mychem
rownames(EC)=mychem
final.df=data.frame(direction,w2pod,w2podupper,EC)
final.notlog10.df=data.frame(direction,10^w2pod,10^w2podupper,10^EC)
names(final.auc)=seq(.25,3,by=.25)
write.csv(final.df,"Data_PS_log10.csv")
write.csv(final.notlog10.df,"Data_PS_originalscale.csv")
```

fitfunctions code:

```
logit4=function(param,x,y){
 # THIS FUNCTION RETURNS THE -LOG LIKELIHOOD FOR THE 4 PARAMETER (5 IF YOU
INCLUDE THE VARIANCE)
 #                 LOGISTIC                 MODEL                 y=min+(max-
min)*(exp(beta0+beta1*x)/(1+exp(beta0+beta1*x)))+error
 minimum=param[1]
 maximum=param[2]
 beta0=param[3]
 beta1=param[4]
 logsigma2=param[5]
 sigma2=exp(logsigma2)
 top=exp(beta0+beta1*x)
 bottom=1+exp(beta0+beta1*x)
 exp=minimum+(maximum-minimum)*top/bottom
 loglike=sum(log(dnorm(y,exp,sqrt(sigma2))))
 return(-loglike)
 }

find.fit=function(x,y,minimum,plot.it=F){
 # THIS     FUNCTION PROVIDES STARTING VALUES FOR THE FITTING
 # OF LOGIT4
 startmin=min(y)-1
 startmax=max(y)+1
 scaledy=(y-startmin)/(startmax-startmin)
 yprime=log(scaledy/(1-scaledy))
 linfit=summary(lm(yprime~x))
 startbeta0=linfit$coefficients[1,1]
```

```
 startbeta1=linfit$coefficients[2,1]
 fit=startmin+(startmax-
startmin)*exp(startbeta0+startbeta1*x)/(1+exp(startbeta0+startbeta1*x))
 startlogsigma2=log(var(y-fit))
 if (plot.it==T){
   plot(x,y)
   lines(x,fit) }
 return(list(startmin=startmin,startmax=startmax,

startbeta0=startbeta0,startbeta1=startbeta1,startlogsigma2=startlogsigma2))
 }

inverse.logit=function(z){
 return(exp(z)/(1+exp(z)))}

together.logit4=function(x,y,plot.it=F,threshold=NULL){
 xrange=range(x,na.rm=T) # compute range before dropping points
 which.missing=grep(T,is.na(y))
 which.drop=grep(T,y<=threshold)
 if (length(threshold)>0) {
   if (length(which.drop)>0) {
       xdropped=x[which.drop];ydropped=y[which.drop]
       x=x[-which.drop];y=y[-which.drop]}
  }
 if (length(which.missing)>0) {x=x[-which.missing];y=y[-which.missing]}
 param.start=unlist(find.fit(x=x,y=y))

result=optim(logit4,par=param.start,x=x,y=y,method="BFGS",control=list(abstol
=1e-12),hessian=T)
 best=result$par
 minimum=best[1]
 maximum=best[2]
 beta0=best[3]
 beta1=best[4]
 sigma2=exp(best[5])
 se=sqrt(diag(solve(result$hessian))) # the logit4 function return -log-like,
so here things are positive
 fit=minimum+(maximum-minimum)*inverse.logit(beta0+beta1*x)
 z=beta1/se[4]
 p=2*pnorm(-abs(z))
 if (plot.it==T){
  plot(x,y,pch=16,xlim=xrange) # the plotted points
  if (length(which.drop)>0){
        x0=xdropped;x1=xdropped
        offset=seq(0,.05,length.out=length(which.drop))
        y0=min(y)+(.1+offset)*(max(y)-min(y));y1=min(y)+offset*(max(y)-
min(y))
        arrows(x0,y0,x1,y1,col=2)
        }
  mtext(paste("num dropped=",length(which.drop)),3,col=2)
  fancyx=seq(min(x),max(x),length.out=1000)
  fancyy=minimum+(maximum-minimum)*inverse.logit(beta0+beta1*fancyx)
  lines(fancyx,fancyy,col=1,lwd=3)} # to change line fit, modify
 controlmean=mean(y[x==min(x)])
 controlsd=sd(y[x==min(x)])
 ncontrol=length(y[x==min(x)])
 controlsdupper=sqrt((ncontrol-1)*controlsd^2/qchisq(.01,ncontrol-1))
```

```
 sdcount=c(1:3)
 ECprop=c(0.1,0.5,0.9)
 sdnames=paste("sd",sdcount,sep="")
 ECnames=paste("EC",round(ECprop*100),sep="")
 if (beta1>0){
  direction="up"
  w=controlmean+sdcount*controlsd
  wupper=controlmean+sdcount*controlsdupper
  for (i in (1:length(w))){abline(h=w,lty=2,col=1:length(w))}
  wprime=(w-minimum)/(maximum-minimum)
  xprime=(log(wprime/(1-wprime))-beta0)/beta1
  wprimeupper=(wupper-minimum)/(maximum-minimum)
  xprimeupper=(log(wprimeupper/(1-wprimeupper))-beta0)/beta1
  EC=(log(ECprop/(1-ECprop))-beta0)/beta1
   if (plot.it==T){
     abline(v=xprime,col=1:length(w),lty=2)
     abline(v=EC,col=c("black","grey","light grey"))
     text(xprime,min(y)+.05*(max(y)-min(y)),sdnames,cex=0.75,col=1:length(w))
     text(EC,min(y),ECnames,cex=0.75,col=c("black","grey","light grey"))
   }
  }
 if (beta1<0){
  direction="down"
  w=controlmean-sdcount*controlsd
  wupper=controlmean-sdcount*controlsdupper
  for (i in (1:length(w))){abline(h=w,lty=2,col=1:length(w))}
  wprime=(w-minimum)/(maximum-minimum)
  xprime=(log(wprime/(1-wprime))-beta0)/beta1
  wprimeupper=(wupper-minimum)/(maximum-minimum)
  xprimeupper=(log(wprimeupper/(1-wprimeupper))-beta0)/beta1
  EC=(log((1-ECprop)/(ECprop))-beta0)/beta1
   if (plot.it==T){
         abline(v=xprime,col=1:length(w),lty=2)
         abline(v=EC,col=c("black","grey","light grey"))
         text(xprime,min(y)+.05*(max(y)-
min(y)),sdnames,cex=0.75,col=1:length(w))
         text(EC,min(y),ECnames,cex=0.75,col=c("black","grey","light grey"))
         }
  }
  xprime[is.na(xprime)]=max(xrange)
  xprime[xprime>max(x)]=max(xrange)
  xprimeupper[is.na(xprimeupper)]=max(xrange)
  xprimeupper[xprimeupper>max(x)]=max(xrange)

return(list(best=best,z=z,p=p,direction=direction,EC=EC,bmd=xprime,bmdupper=x
primeupper))
 }
polyfit=function(x,y,grid=500,plot.it=F){
 # this function returns a third polynomial fit, and
 # finds a bmd-like value at which we are 1 sd beyond the control
 # and also ECx -like values
 which.control=grep(T,x==min(x))
 sd.control=sd(y[which.control])
 mymax=mean(y[x==max(x)])
 mymin=mean(y[x==min(x)])
 x1=x
 x2=x^2
```

```
 x3=x^3
 betahat=summary(lm(y~x1+x2+x3))$coef[,1]
 xgrid=seq(min(x),max(x),length.out=grid)
 fit=betahat[1]+betahat[2]*xgrid+betahat[3]*xgrid^2+betahat[4]*xgrid^3
  prop=abs((fit-fit[1])/(fit[length(fit)]-fit[1]))
 EC10=xgrid[which.min((prop-0.1)^2)]
 EC50=xgrid[which.min((prop-0.5)^2)]
 bmd=xgrid[which.min((abs(fit-fit[1])-sd.control)^2)]

 if (plot.it==T){plot(x,y,pch=16)
                lines(xgrid,fit)
                abline(v=EC10,col=1);abline(v=EC50,col=2);

abline(v=bmd,col=3);abline(h=fit[1]+sd.control,lty=2);abline(h=fit[1]-
sd.control,lty=2)
                }
 return(list(EC10=EC10,EC50=EC50,bmd=bmd))
 }
```
