# Supplementary material for: Grouping of UVCB Substances with New Approach Methodologies (NAMs) Data
Source: ALTEX. Author manuscript; Available in PMC 2021 Feb 23. (PMC7900923; doi:10.14573/altex.2006262)

Supplemental File 2. Experiment description and QC data for A375, A549, HepG2, HepaRG, MCF7, HT29, LN229, HLMVEC and SH-SY5Y cells.

Table 1 – Cell lines.

**Cell name**	**Tissue**	**Sex**	**Description**	**Karyotype**
A375 (ECACC 88113005)	Skin	F	From 54 year old female with malignant melanoma.	62 Chromosomes - hypotriploid
A549 (ECACC 86012804)	Lung epithelial	M	From 58 year old Caucasian male	
HepG2 (ECACC 85011430)	Hepatocyte carcinoma	M	Isolated from a liver biopsy of a male Caucasian aged 15 years, with a well differentiated hepatocellular carcinoma.	
HepaRG (Biopredic International)	Liver	F	Terminally differentiated hepatic cells derived from a human hepatic progenitor cell line. Differentiated by hydrocortisone (50µM).	2n=46, translocation t(12;22) and remodelled Ch7. Loos of 12p.
MCF7 (ECACC 86012803)	Breast	F	From the pleural effusion from a 69 year Caucasian. Cells express both the wild type and variant oestrogen receptors as well as progesterone receptor.	2n=46 (normal) chromosomes
HT29 (ECACC 91072201)	Colon	F	from a primary tumour in a 44 year old Caucasian female. Forms a well- differentiated adenocarcinoma consistent with colony primary, grade I. Mutated P53	Chromosomes range 68-72 , mode 71
LN229 (ATCC-CRL- 2611	Glio-blastoma	F	From a 60 year old Caucasian-brain/right frontal parietooccipital cortex Mutated p53.	
HLMVEC (540-05A SIGMA)	Lung endothelial	M/F	A model of the lung endothelium and useful for studying the pathology and biology of the pulmonary microvasculature in vitro	2n=46 (normal)
SH-SY5Y (ECACC 94030304)	Neuro-blastoma	F	Established from a metastatic bone tumor.	Modal chromosomes = 47. Trisomy 1q.

TREATMENTs

All experiments were conducted in 384 well plates with the outer wells containing 1x PBS (total of 308 wells for screening). Cells were maintained in stock culture prior to use and transferred to the 384 well plates (12-15000 cells per well) at least 24 hours before use. They were allowed to adhere and grow for 24 hours or more if they appeared too sub-confluent in a media containing foetal bovine serum (FBS). Before treatment the media was replaced with one that was FBS free and to this was added the stock chemicals. An Eppendorf epiMotion robotic 96 well pipettor was used for the liquid transfers. Briefly, four 100% DMSO stock plates which contained the petroleum substances at 200x final exposure concentration, prepared by Texas A&M University, was used for all screens. Each plate consisted of necessary controls and had a single concentration of petroleum substance. Four plates were needed to obtain the four point concentration range. Working plates were then prepared of a 40x dilution of petroleum substance extracts in 2.5% DMSO by adding 4uL of stock chemical to 156uL FBS-free media and mixing 4x after addition (40uL mix volume) using the Epmotion pipettor. 5uL of this pre- dilution was added to the 20uL media on the cells to attain a 1x concentration, mixing 4x with 20uL mix volume. For the majority of extracts this resulted in a concentration range of 1 µg/ml, 10 µg/ml,

100 µg/ml, 1000 µg/ml of petroleum substance in media containing 0.5% DMSO. Treated plates were then incubated for 24 hours exposure time at 37◦C in 5% CO_2 for all assays.

ASSAY-SPECIFIC POSITIVE CONTROLS

Assay specific positive controls were used for all cell types listed in Table 1. These consisted of agents with known effects in the assays being used to confirm that the assay was functioning as expected.

Table 2. Assay specific positive control chemicals.

Cell membrane integrity	CMFDA	Triton X100
Reactive Oxygen Species	ROS	DMNQ
Apoptosis	CASP	Mitomycin C/Etopiside
Protein synthesis inhibition	PROT	Hygromycin B
ATP Quantitation Assay	ATP	Rotenone

QA/QC METRICS

To ensure the integrity of the data, several aspects were assessed for each endpoint; all graphs were generated in R. First, vehicle effects were determined by comparing method blank, DMSO, and media wells. This was done by calculating the IQR for the normalized method blank values across all four plates (112 wells) and comparing this with a the IQR of a scaled ratio between the grouped intraplate replicates (row 8) and their pairs scattered throughout the plate (dispersed group) across the four plates. The scaled variable was rep1-rep2/sqrt(rep1*100) where rep 1 is the replicate in the grouped set and rep2 the replicate in the dispersed set.

Tetraoctylammonium bromide (TBA) was also included as a generally cytotoxic control. This should be positive with the cytotoxic phenotypic marker (Alamar Blue) but not necessarily with the other end points. These data were aggregated into a box-and-whisker plot with the four treatment conditions depicting any potential vehicle effects. Second, assay specific positive control chemicals used for each cell type were plotted for concentration response with a nonlinear line fit to the data. This ensured that the assay was functioning as expected. Third, intra-plate replicate controls were plotted as a scatterplot with one replicated as the x-value and the other replicated as the y-value. Pearson's r, least squares (r^2) and Spearman's (monotonic rank) ρ correlations were calculated and are denoted in the scatterplot along with a p-value of significance. Lastly, inter-plate replicates were plotted as concentration response with a nonlinear fit applied to the date. Each concentration response from the four plates were plotted separately as its own concentration response to determine if the concentration response from four different plates were similar. All figures were compiled and visually assessed to determine if the assay screen worked as expected and whether endpoints should be included for further evaluation.

Supplemental File 2. Experiment description and QC data for A375, A549, HepG2, HepaRG, MCF7, HT29, LN229, HLMVEC and SH-SY5Y cells.

1. Cell Viability

These are the assays that measured cell viability using the Alamar Blue assay that produces a blue precipitate in live cells as a result of reduction by the cells. This does not happen in dead cells.
- A– controls normalised to method blank. Media (columns 1 to 4, DMSO (columns 5-8) and method blank (columns 9-12). IQR is that for all the methods blanks across the four plates.
- B – TAB controls normalise against method blank for each location (column, row) across the four plates of the assay set
- C – scatter plot of the intraplate controls normalised to the method blank with Pearsons (dashed) and least mean squares plot lines (solid) included
- D – Scaled ratios of the intraplate replicates with the IQR for the values across all four plates included.
- E – boxplot of the triton x100 response normalised to the method blank
- F – line plot of the triton X100 response normalised to the method blank showing SDs and t-tests for each response against the method blanks

Supplemental File 2. Experiment description and QC data for A375, A549, HepG2, HepaRG, MCF7, HT29, LN229, HLMVEC and SH-SY5Y cells.

1.1. **HepG2 (Viability)**

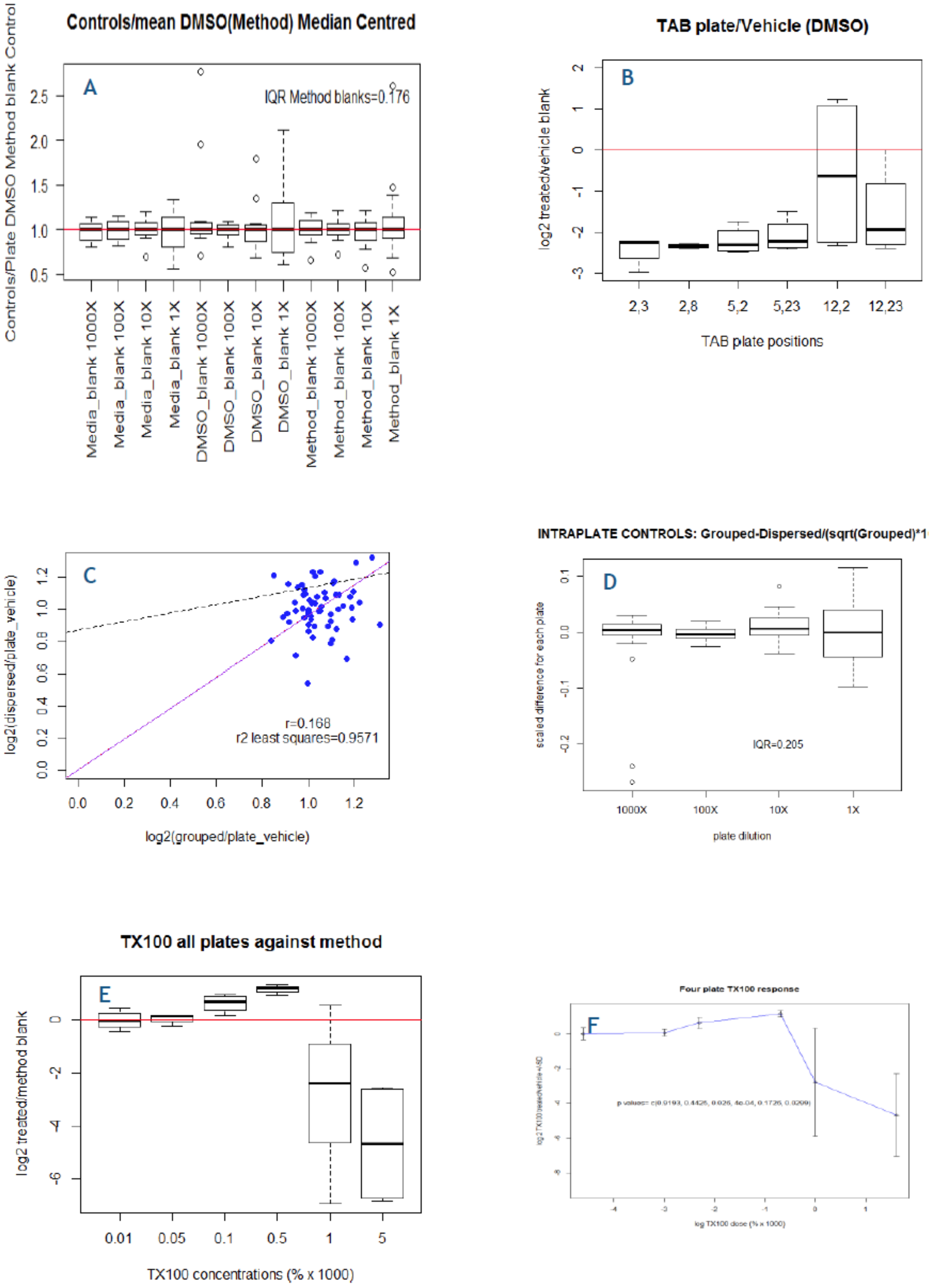

Supplemental File 2. Experiment description and QC data for A375, A549, HepG2, HepaRG, MCF7, HT29, LN229, HLMVEC and SH-SY5Y cells.

1.2 A375 (viability)

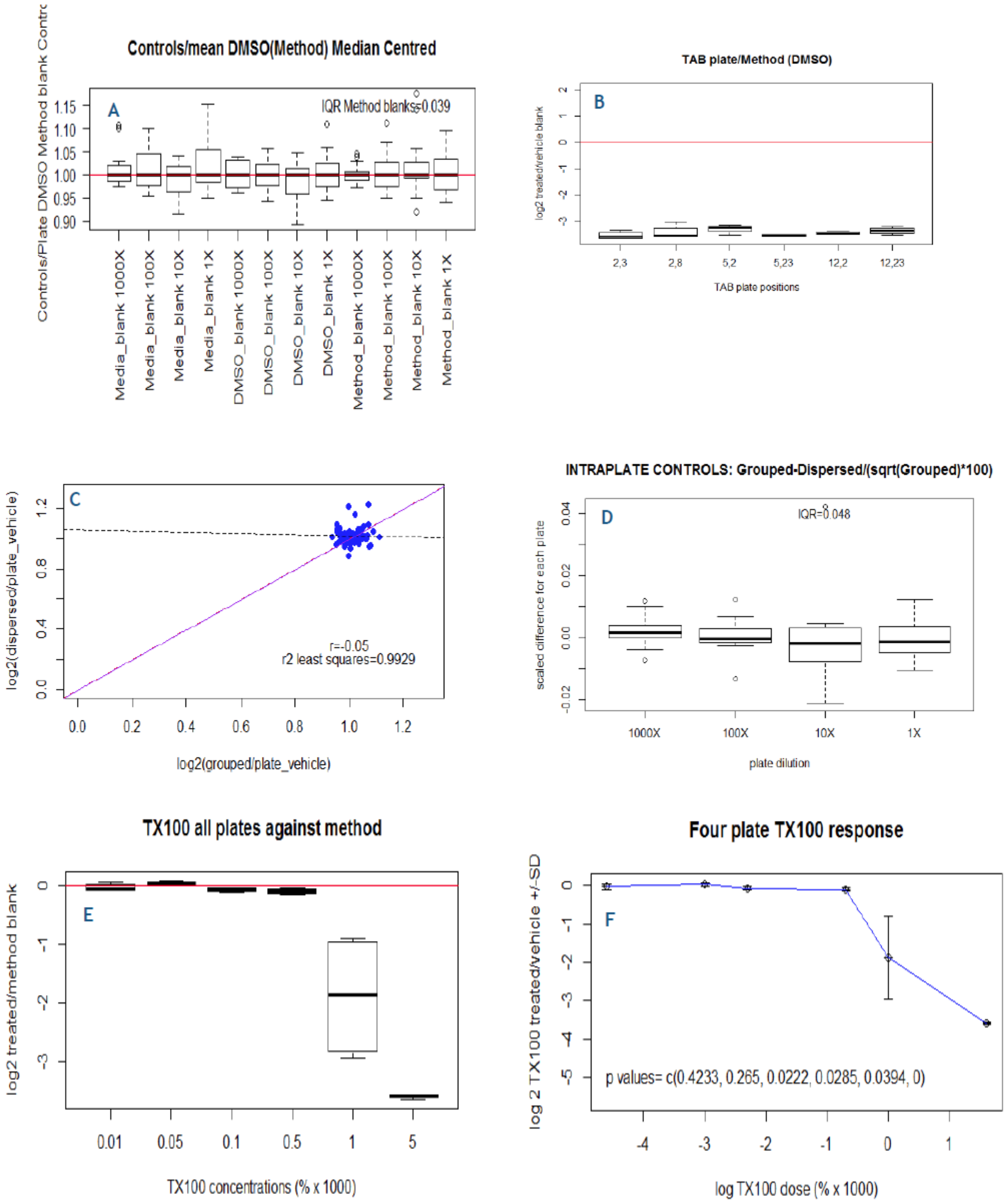

Supplemental File 2. Experiment description and QC data for A375, A549, HepG2, HepaRG, MCF7, HT29, LN229, HLMVEC and SH-SY5Y cells.

1.3. A549 (viability)

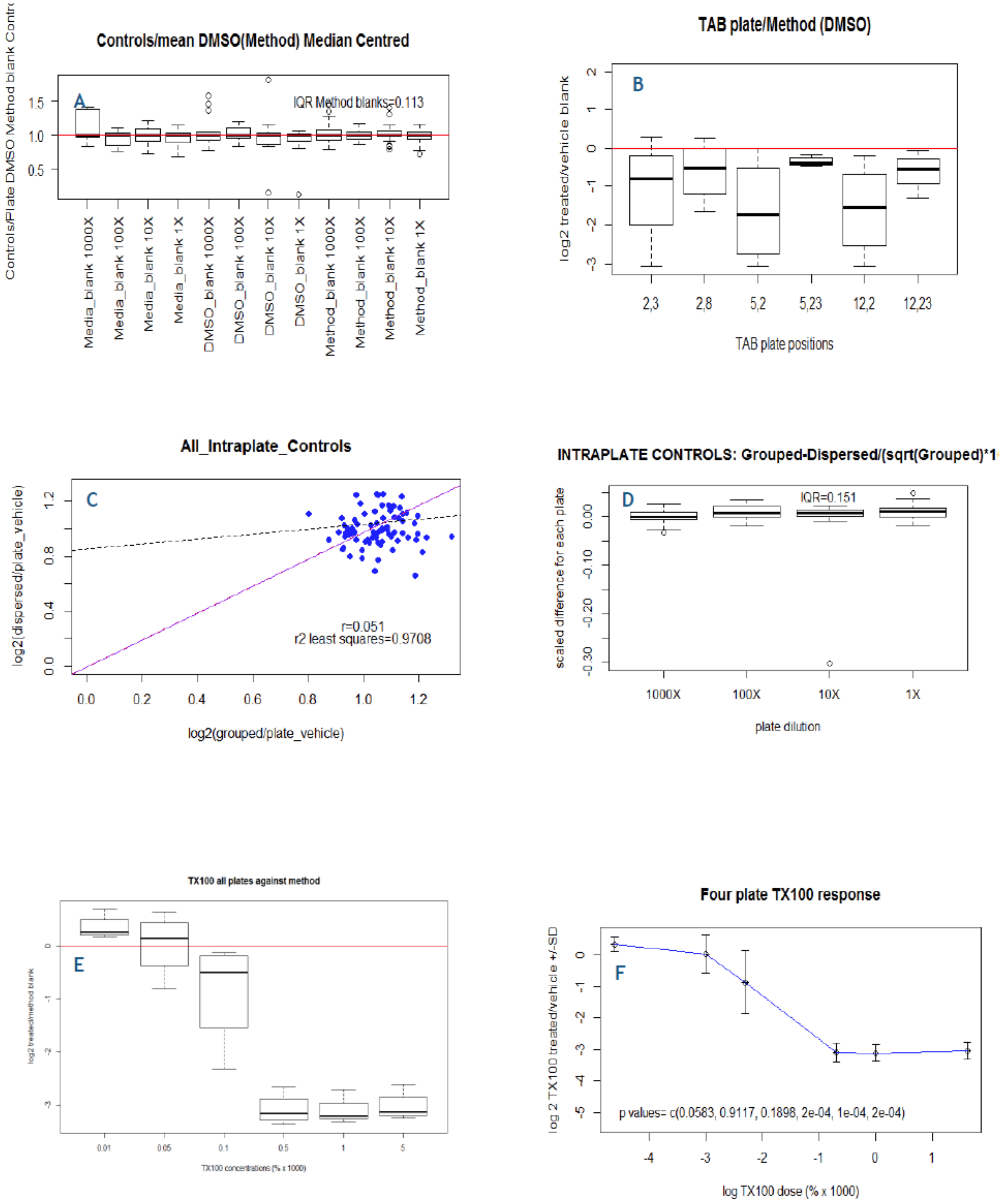

Supplemental File 2. Experiment description and QC data for A375, A549, HepG2, HepaRG, MCF7, HT29, LN229, HLMVEC and SH-SY5Y cells.

1.4. MCF7 (viability)

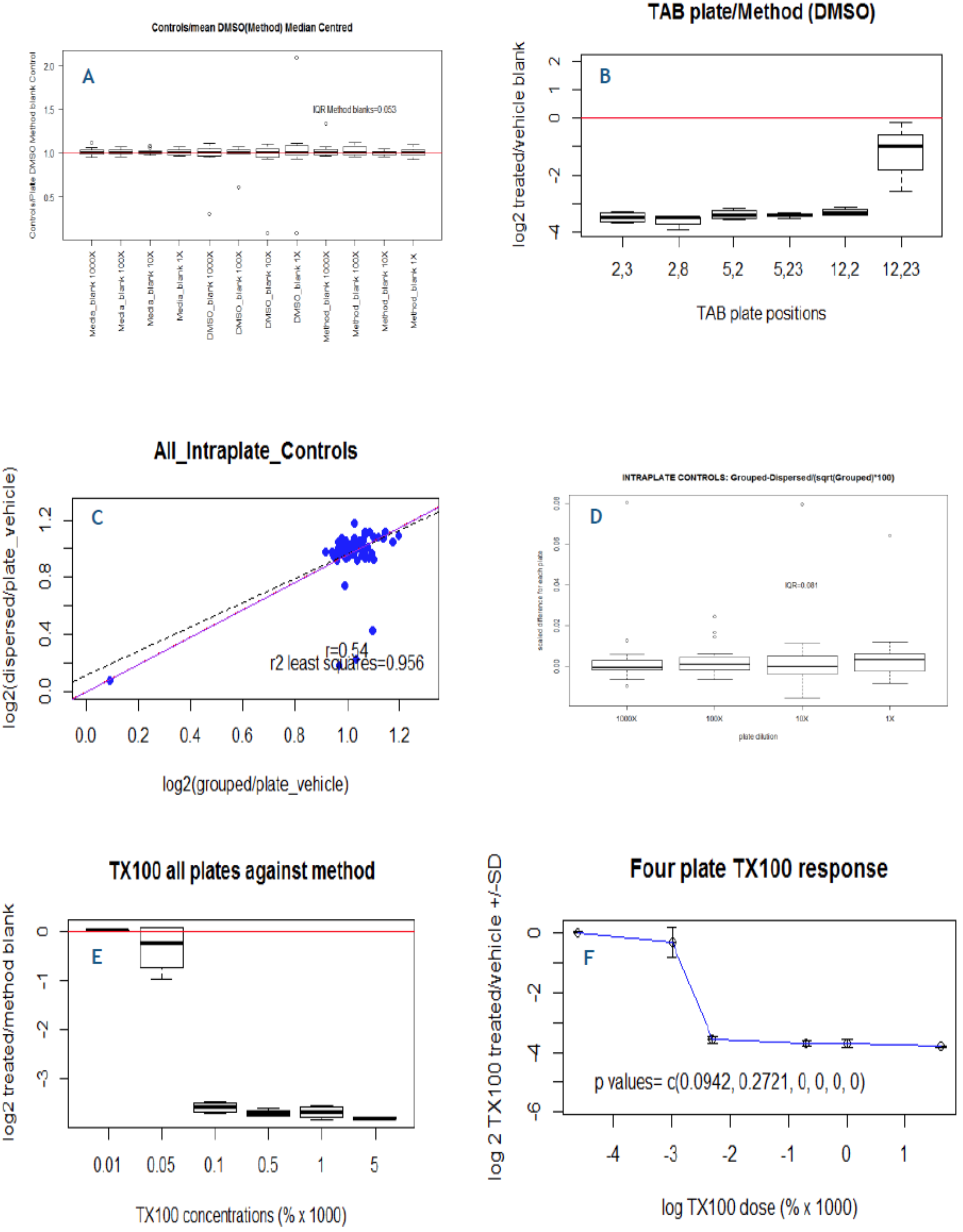

Supplemental File 2. Experiment description and QC data for A375, A549, HepG2, HepaRG, MCF7, HT29, LN229, HLMVEC and SH-SY5Y cells.

1.5. HLMVEC (viability)

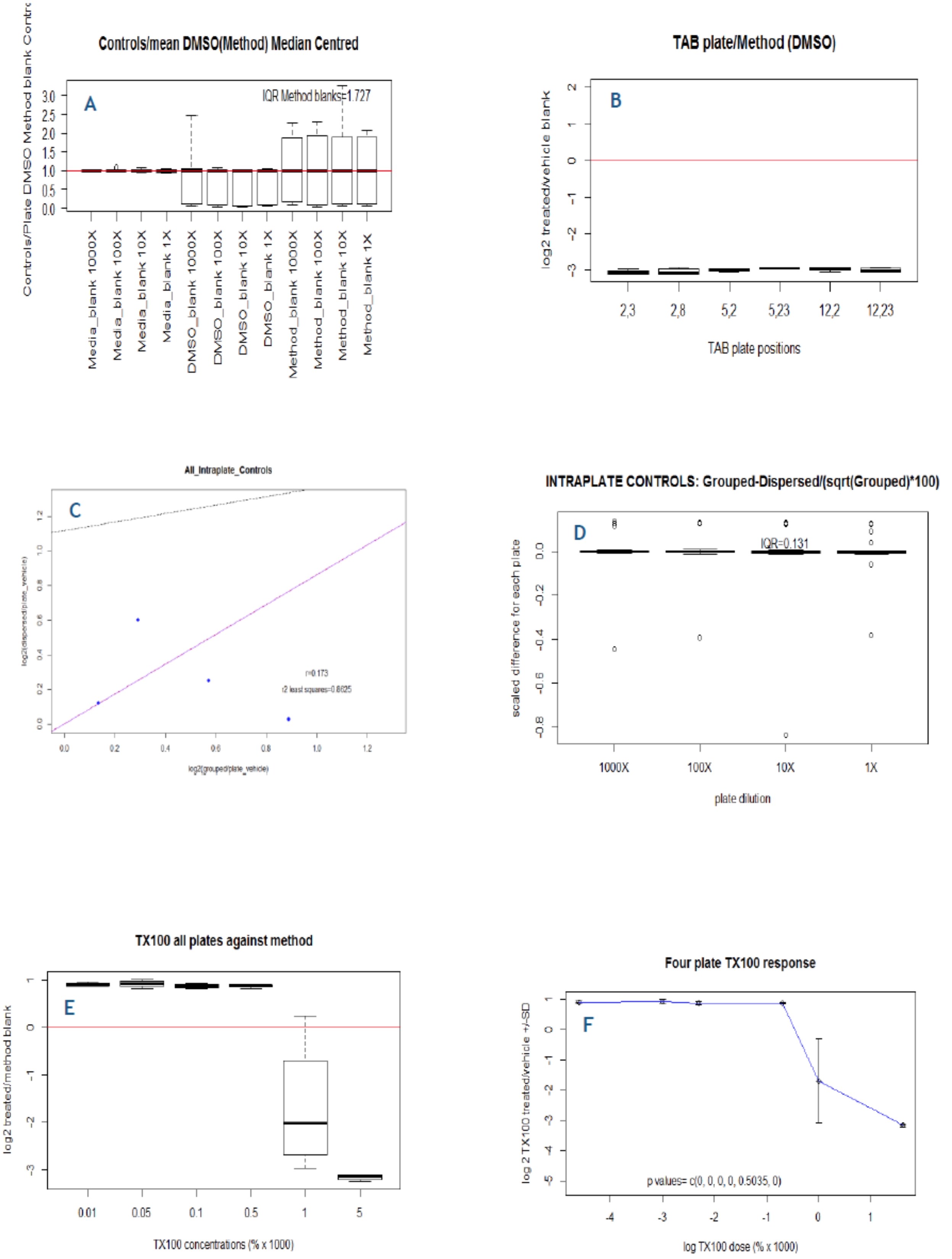

Supplemental File 2. Experiment description and QC data for A375, A549, HepG2, HepaRG, MCF7, HT29, LN229, HLMVEC and SH-SY5Y cells.

1.6 HEPARG (viability)

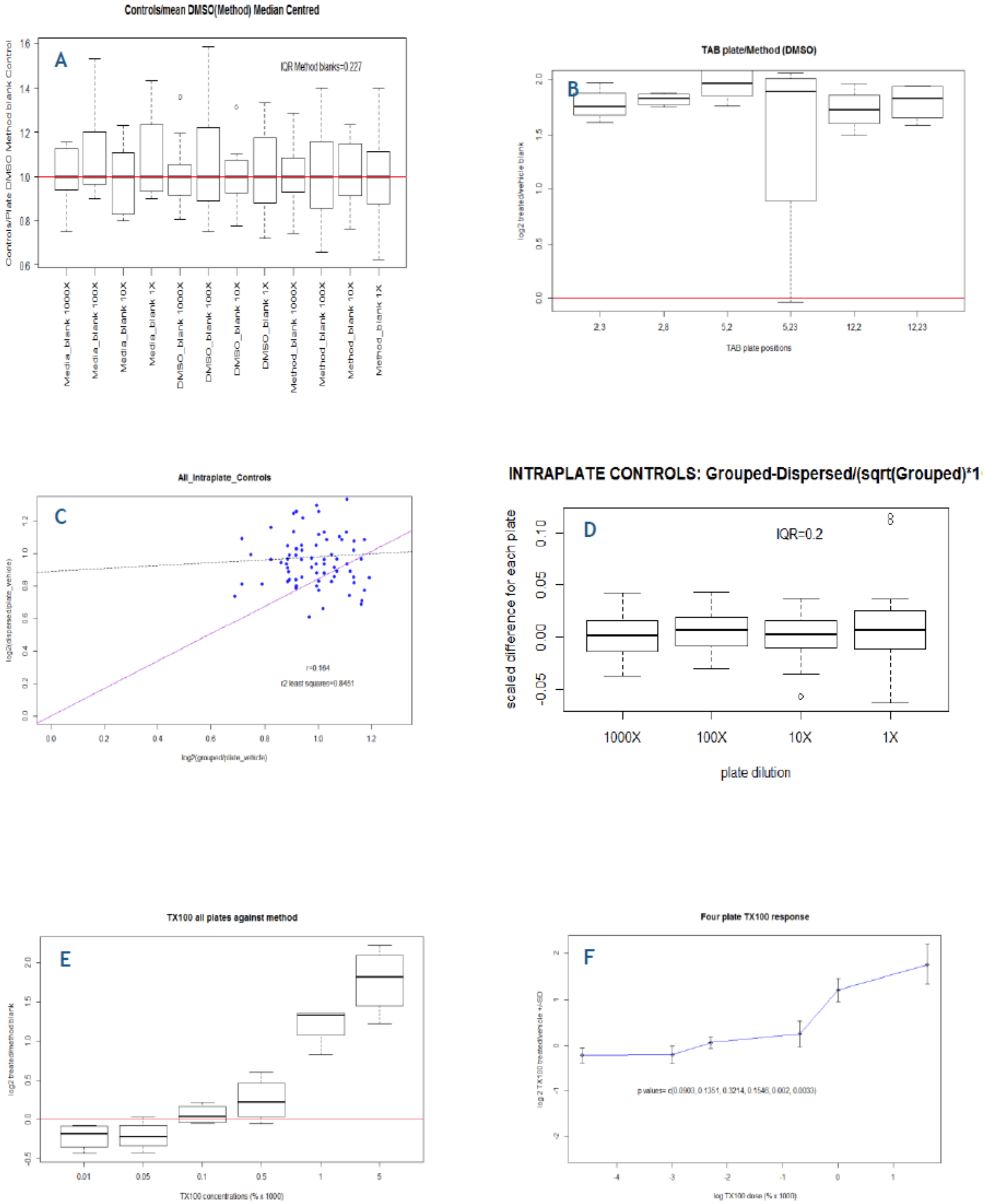

Supplemental File 2. Experiment description and QC data for A375, A549, HepG2, HepaRG, MCF7, HT29, LN229, HLMVEC and SH-SY5Y cells.

1.7. HT29 (viability)

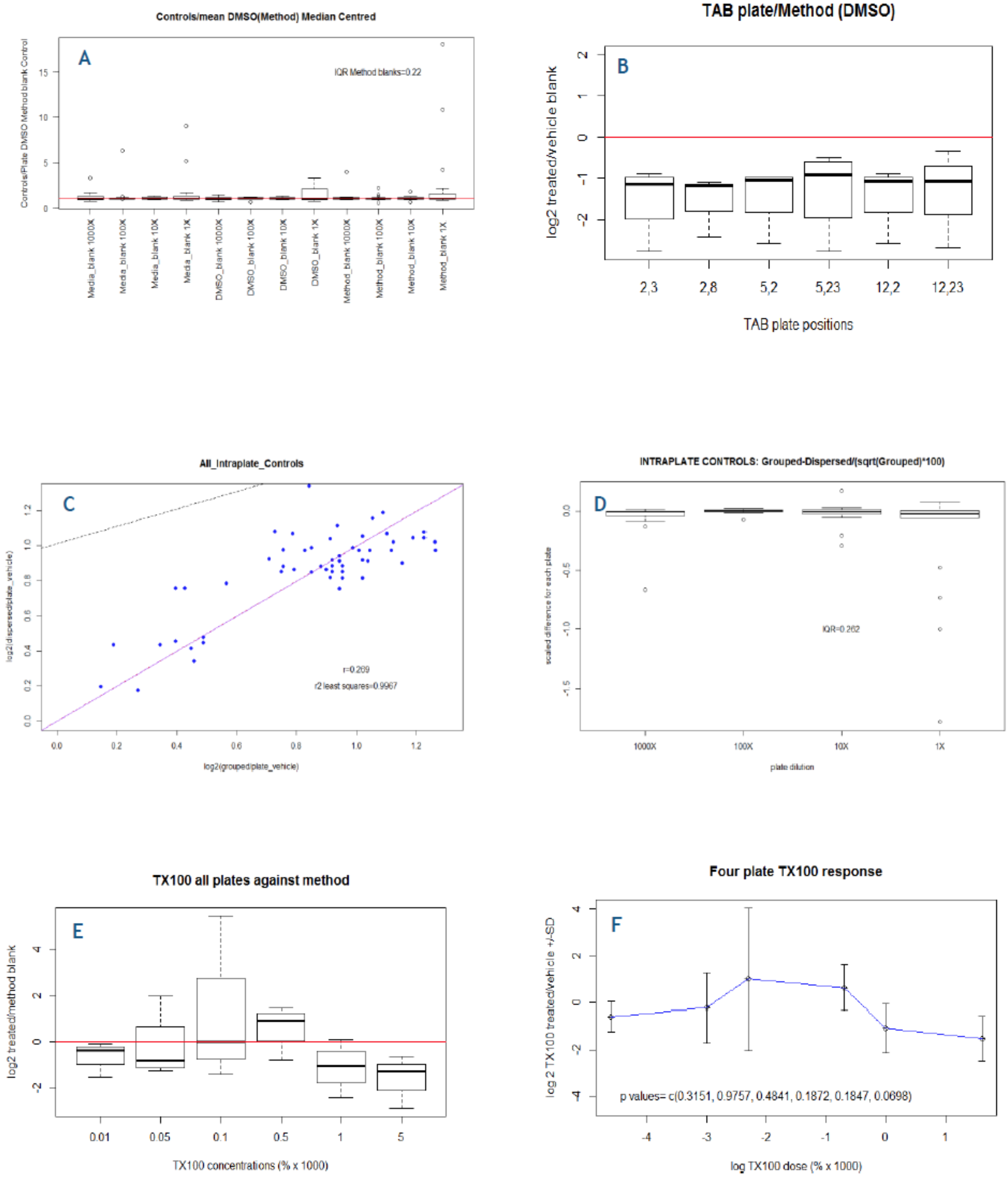

Supplemental File 2. Experiment description and QC data for A375, A549, HepG2, HepaRG, MCF7, HT29, LN229, HLMVEC and SH-SY5Y cells.

1.8. SH-SY5Y (viability)

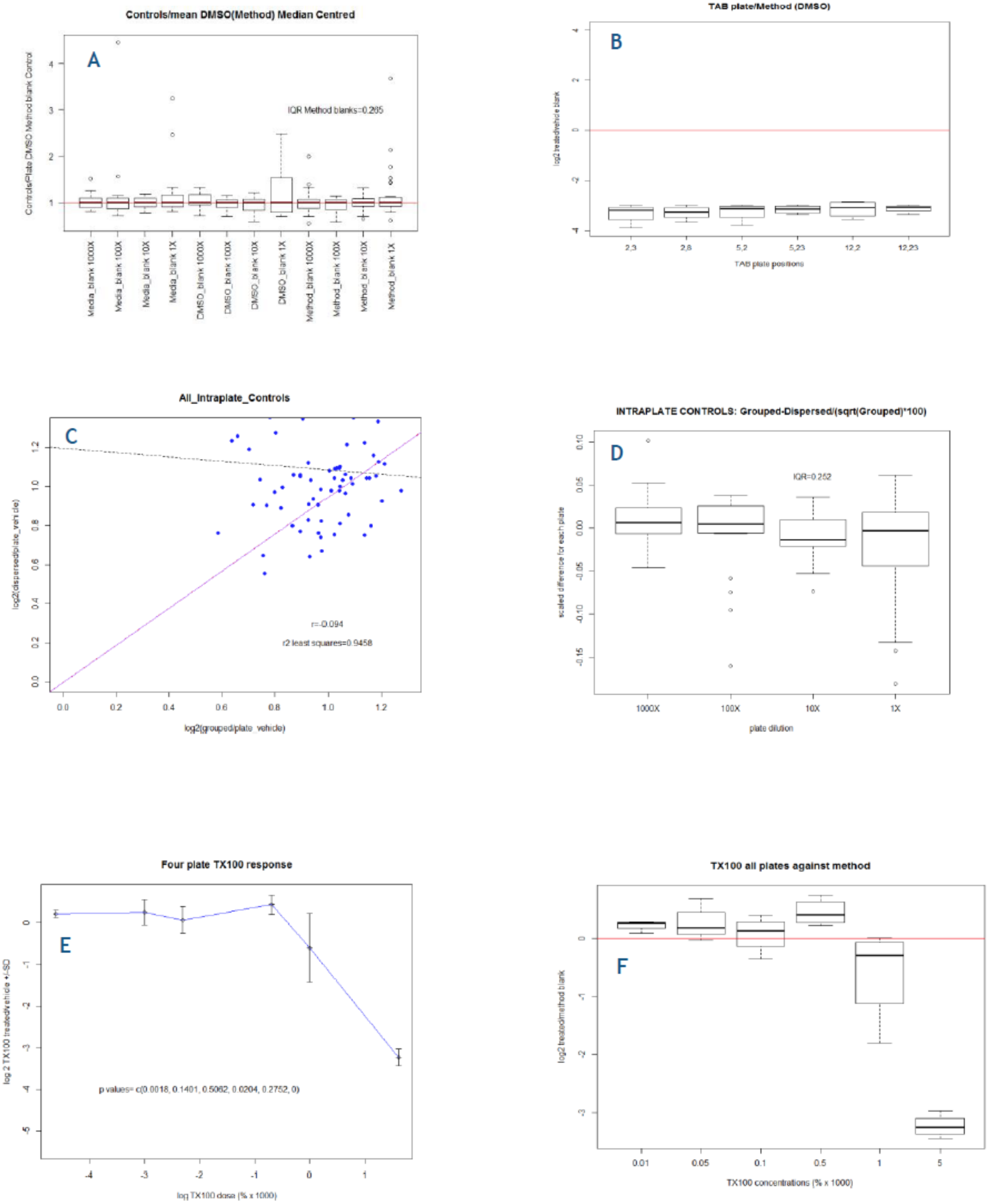

Supplemental File 2. Experiment description and QC data for A375, A549, HepG2, HepaRG, MCF7, HT29, LN229, HLMVEC and SH-SY5Y cells.

1.9. LN229 (viability)

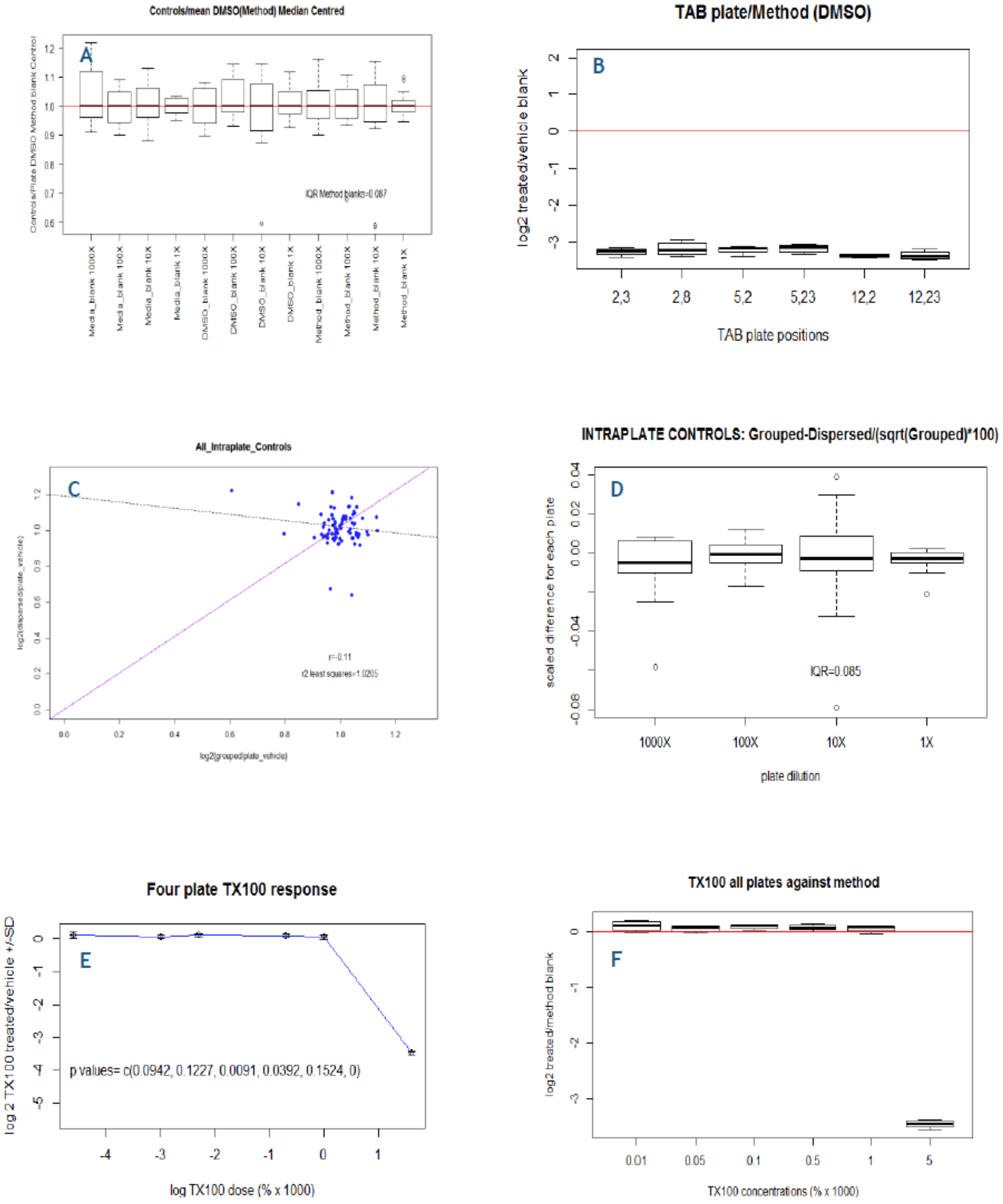

Supplemental File 2. Experiment description and QC data for A375, A549, HepG2, HepaRG, MCF7, HT29, LN229, HLMVEC and SH-SY5Y cells.

2. Reactive Oxygen Species (ROS)

- A – controls normalised to method blank. Media (columns 1 to 4, DMSO (columns 5-8) and method blank (columns 9-12). IQR is that for all the methods blanks across the four plates.
- B – TAB controls normalised against method blank for each location (column, row) across the four plates of the assay set
- C – scatter plot of the intraplate controls normalised to the method blank with Pearsons (dashed) and least mean squares plot lines (solid) included
- D – Scaled ratios of the intraplate replicates with the IQR for the values across all four plates included.
- E – Box plot of the DMNQ response normalised to the method blanks
- F- line plot of the DMNQ response normalised to the method blank showing SDs and t-tests for each response against the method blanks
- G – boxplot of the rotenone response normalised to the method blank
- H – line plot of the rotenone response normalised to the method blank showing SDs and t-tests for each response against the method blanks

Supplemental File 2. Experiment description and QC data for A375, A549, HepG2, HepaRG, MCF7, HT29, LN229, HLMVEC and SH-SY5Y cells.

2.1. HEPG2 (ROS)

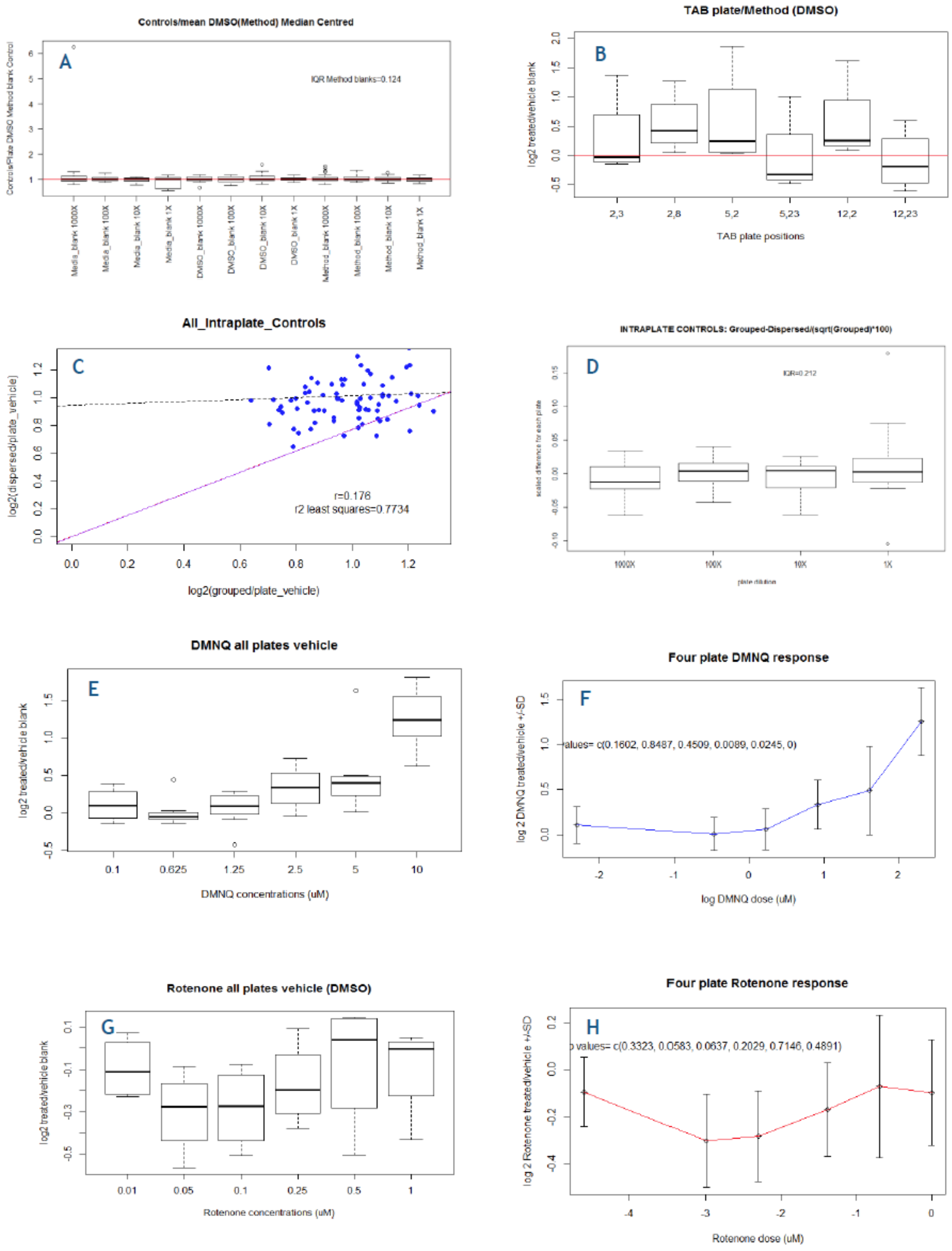

Supplemental File 2. Experiment description and QC data for A375, A549, HepG2, HepaRG, MCF7, HT29, LN229, HLMVEC and SH-SY5Y cells.

2.2. A375 (ROS)

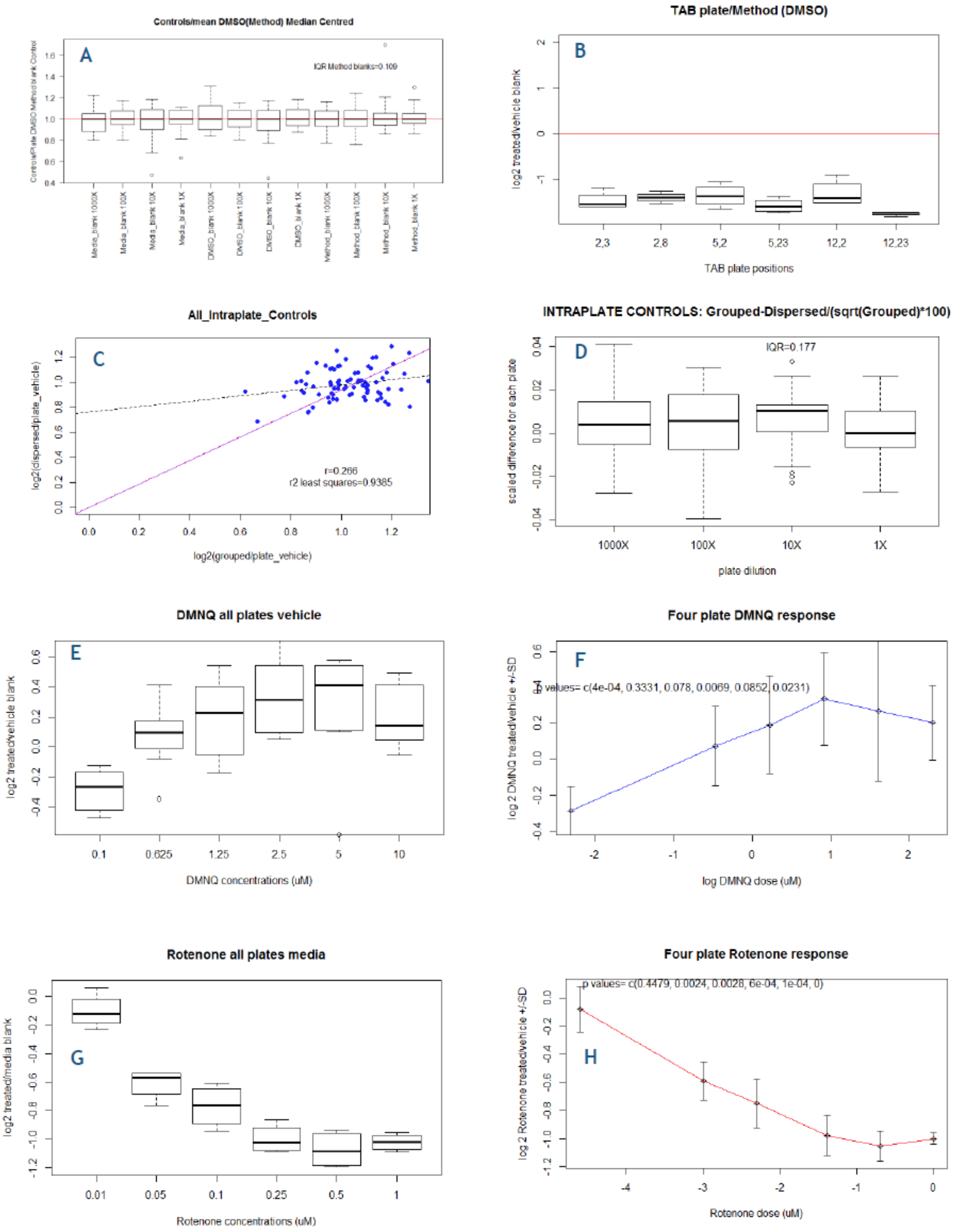

Supplemental File 2. Experiment description and QC data for A375, A549, HepG2, HepaRG, MCF7, HT29, LN229, HLMVEC and SH-SY5Y cells.

2.3. A549 (ROS)

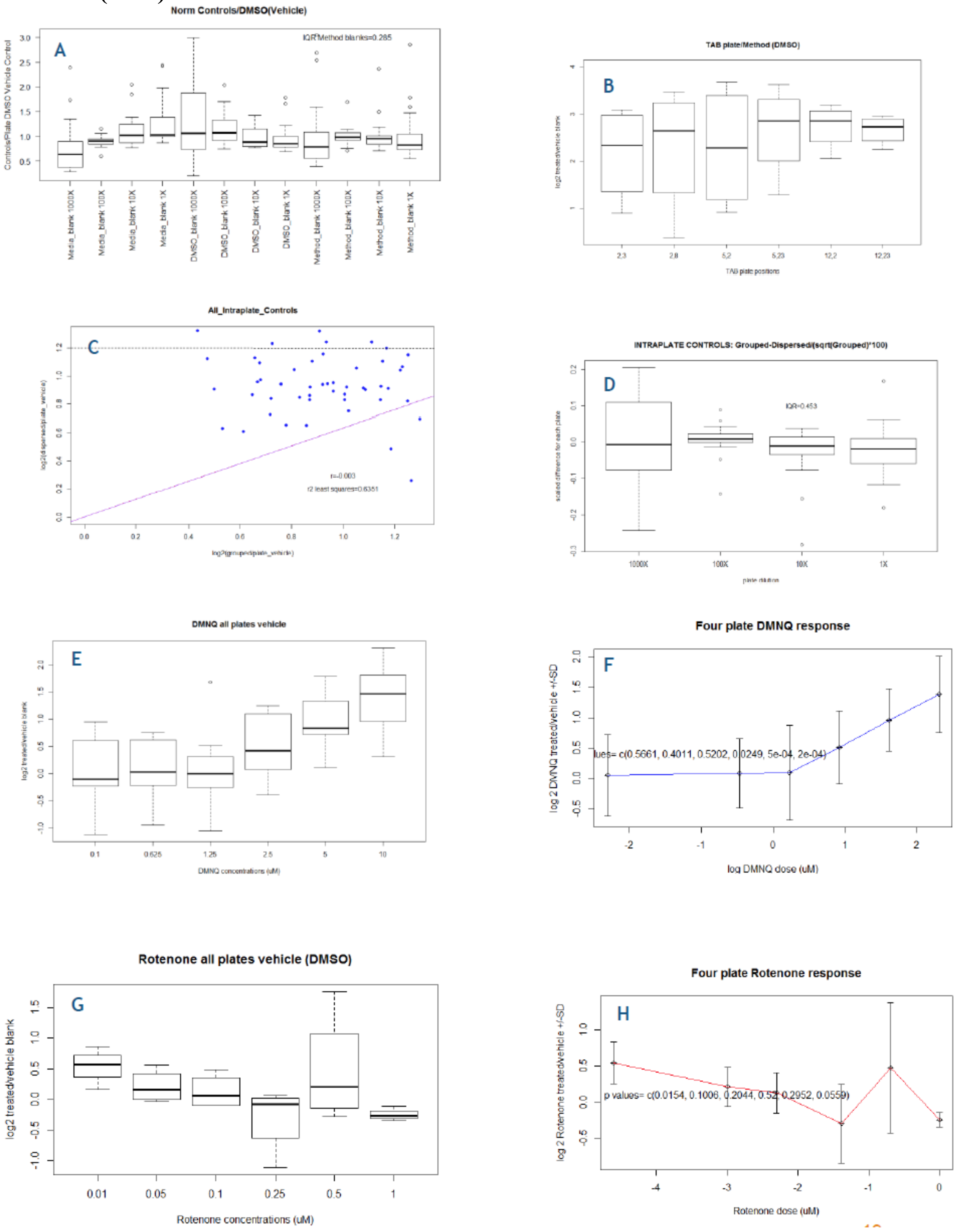

Supplemental File 2. Experiment description and QC data for A375, A549, HepG2, HepaRG, MCF7, HT29, LN229, HLMVEC and SH-SY5Y cells.

2.4. MCF7 (ROS)

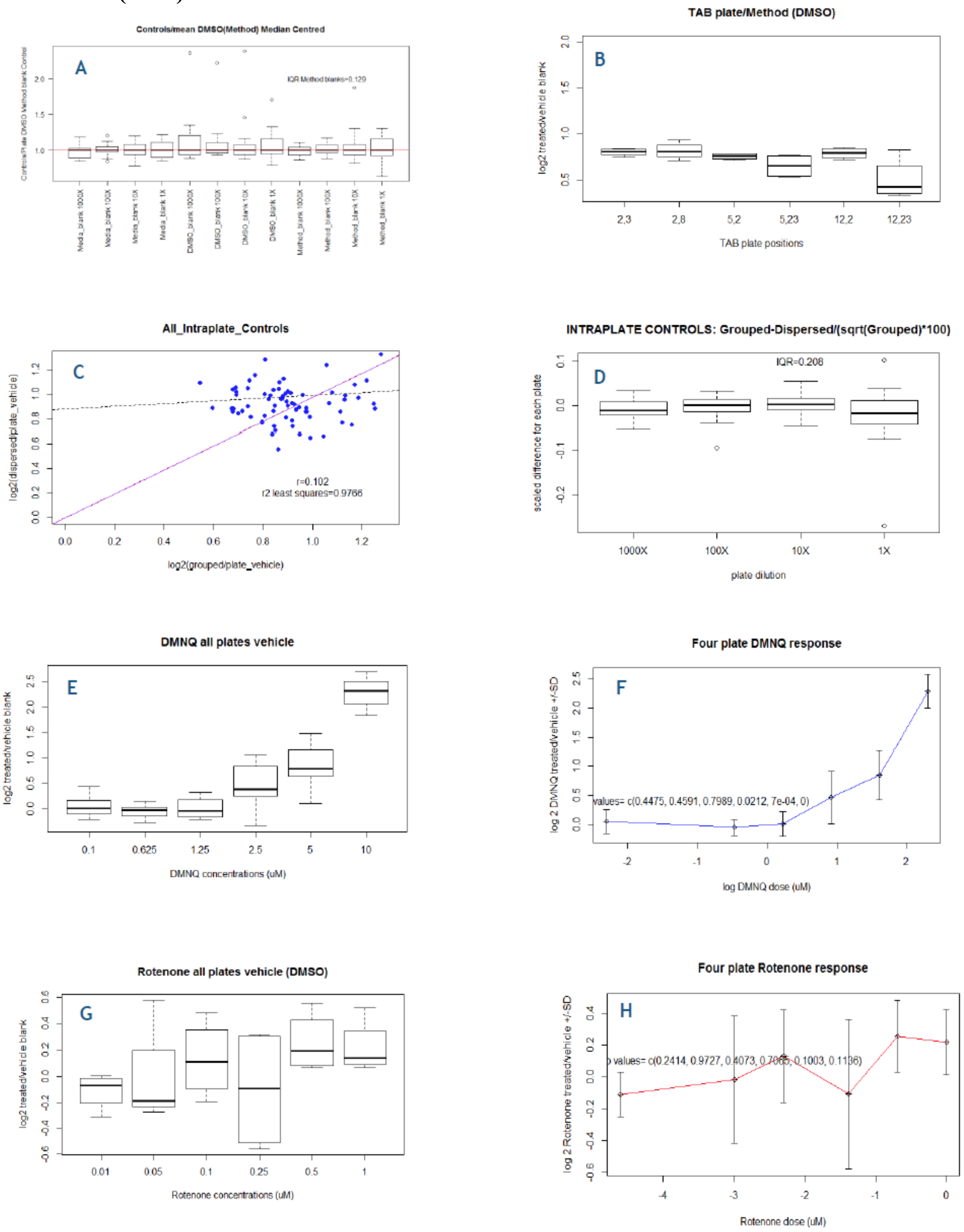

Supplemental File 2. Experiment description and QC data for A375, A549, HepG2, HepaRG, MCF7, HT29, LN229, HLMVEC and SH-SY5Y cells.

2.5. HLMVEC (ROS)

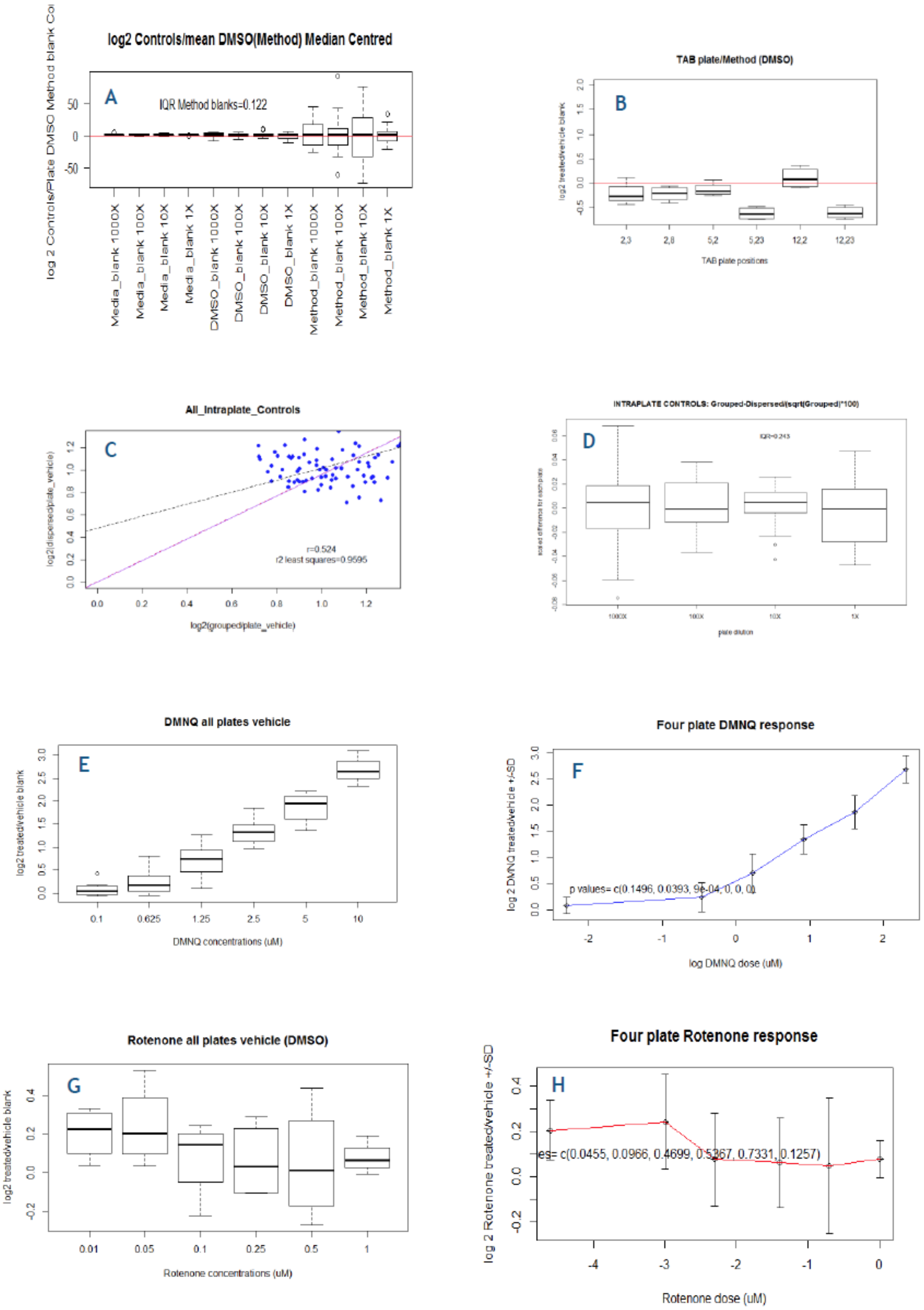

Supplemental File 2. Experiment description and QC data for A375, A549, HepG2, HepaRG, MCF7, HT29, LN229, HLMVEC and SH-SY5Y cells.

2.6. HEPARG (ROS)

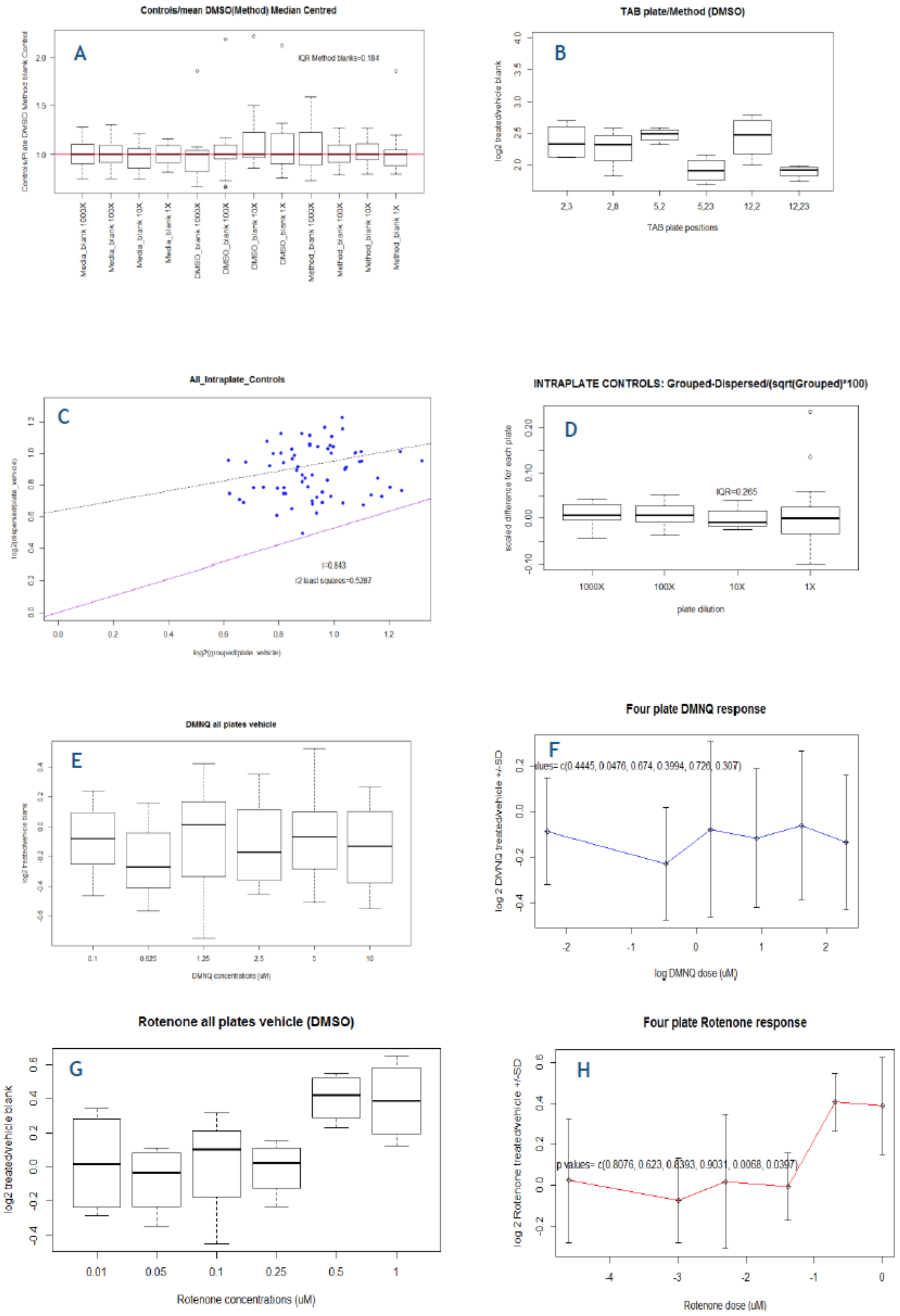

Supplemental File 2. Experiment description and QC data for A375, A549, HepG2, HepaRG, MCF7, HT29, LN229, HLMVEC and SH-SY5Y cells.

2.7. HT29 (ROS)

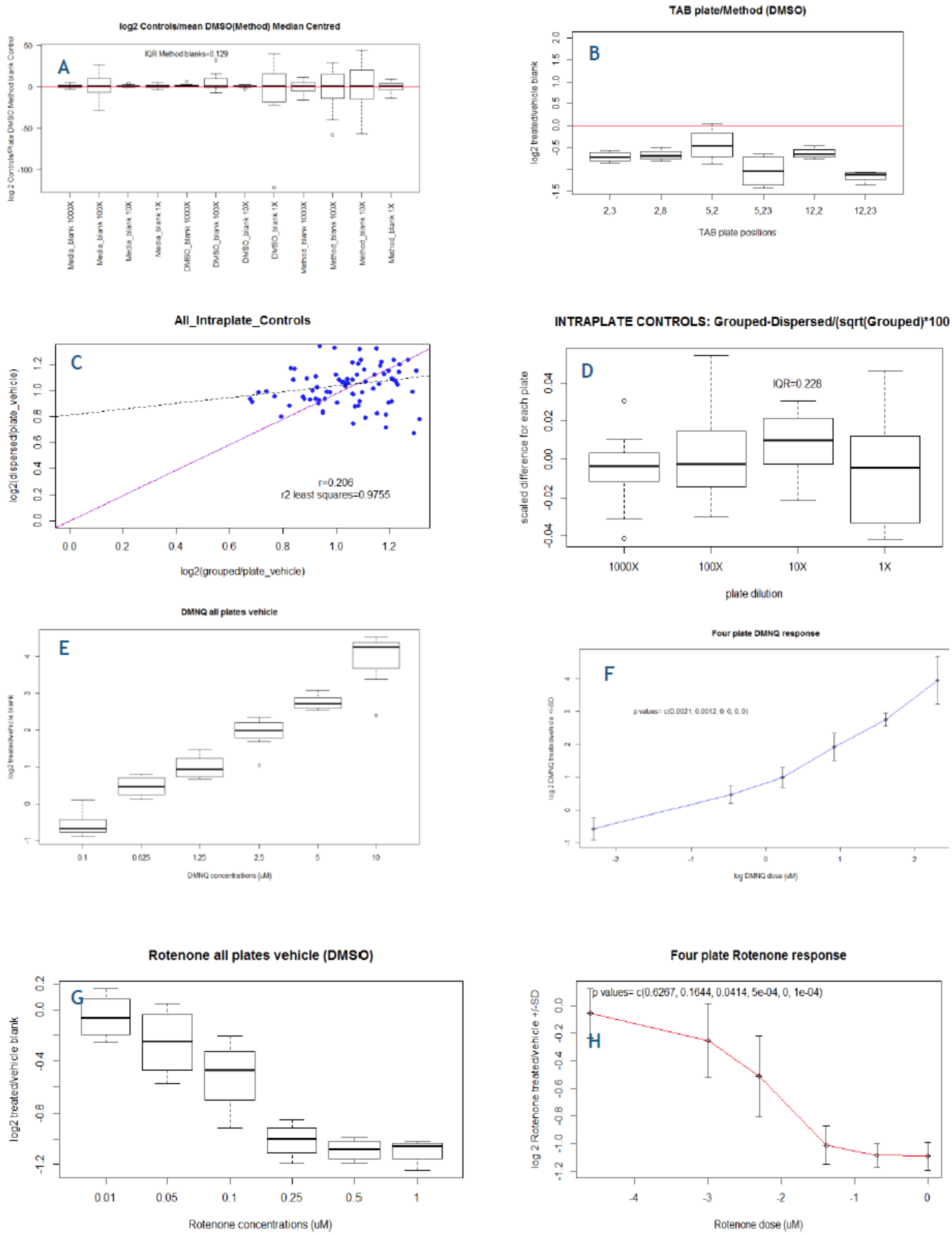

Supplemental File 2. Experiment description and QC data for A375, A549, HepG2, HepaRG, MCF7, HT29, LN229, HLMVEC and SH-SY5Y cells.

2.8. SH-SY5Y (ROS)

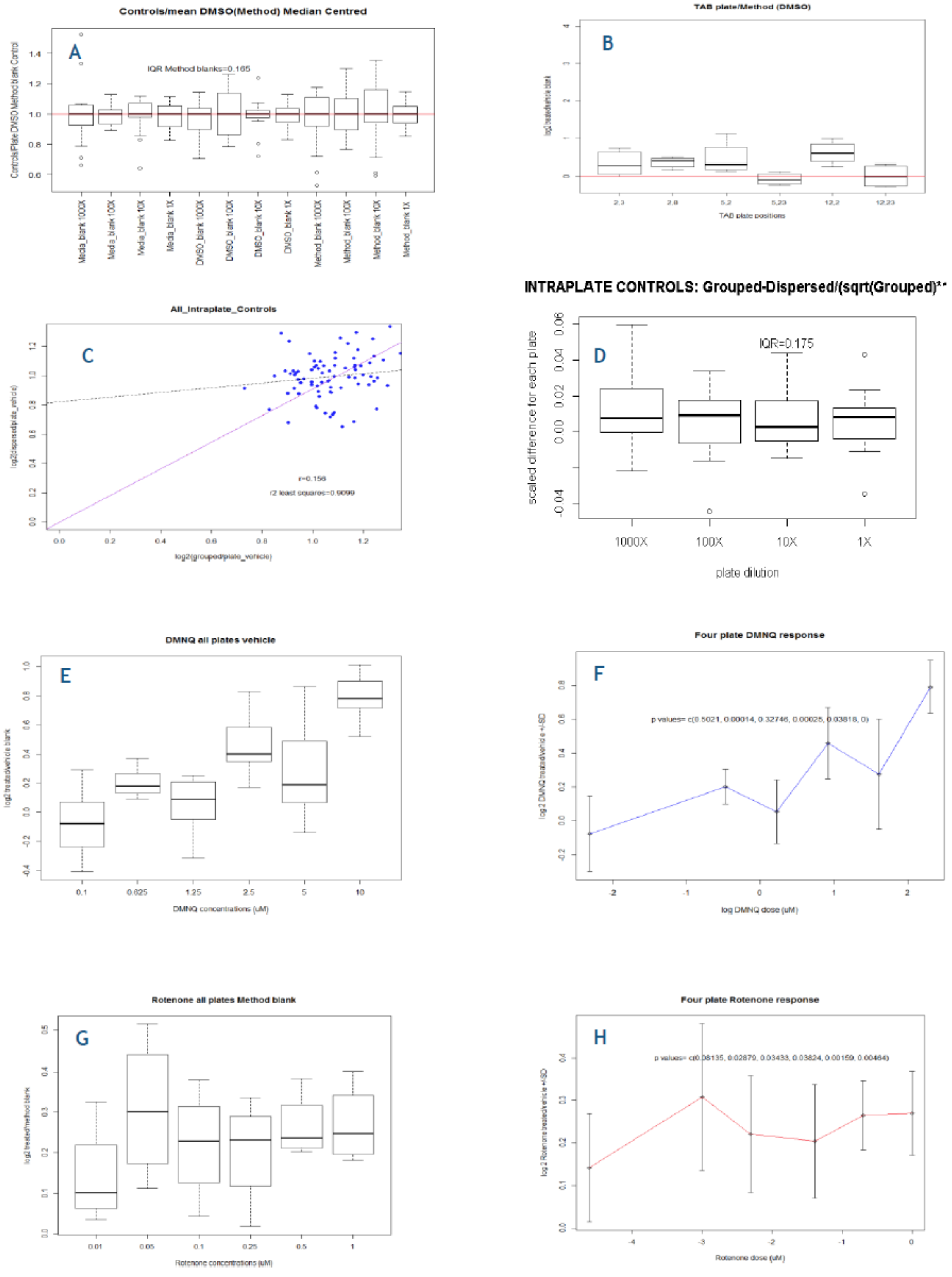

Supplemental File 2. Experiment description and QC data for A375, A549, HepG2, HepaRG, MCF7, HT29, LN229, HLMVEC and SH-SY5Y cells.

2.9. LN 229 (ROS)

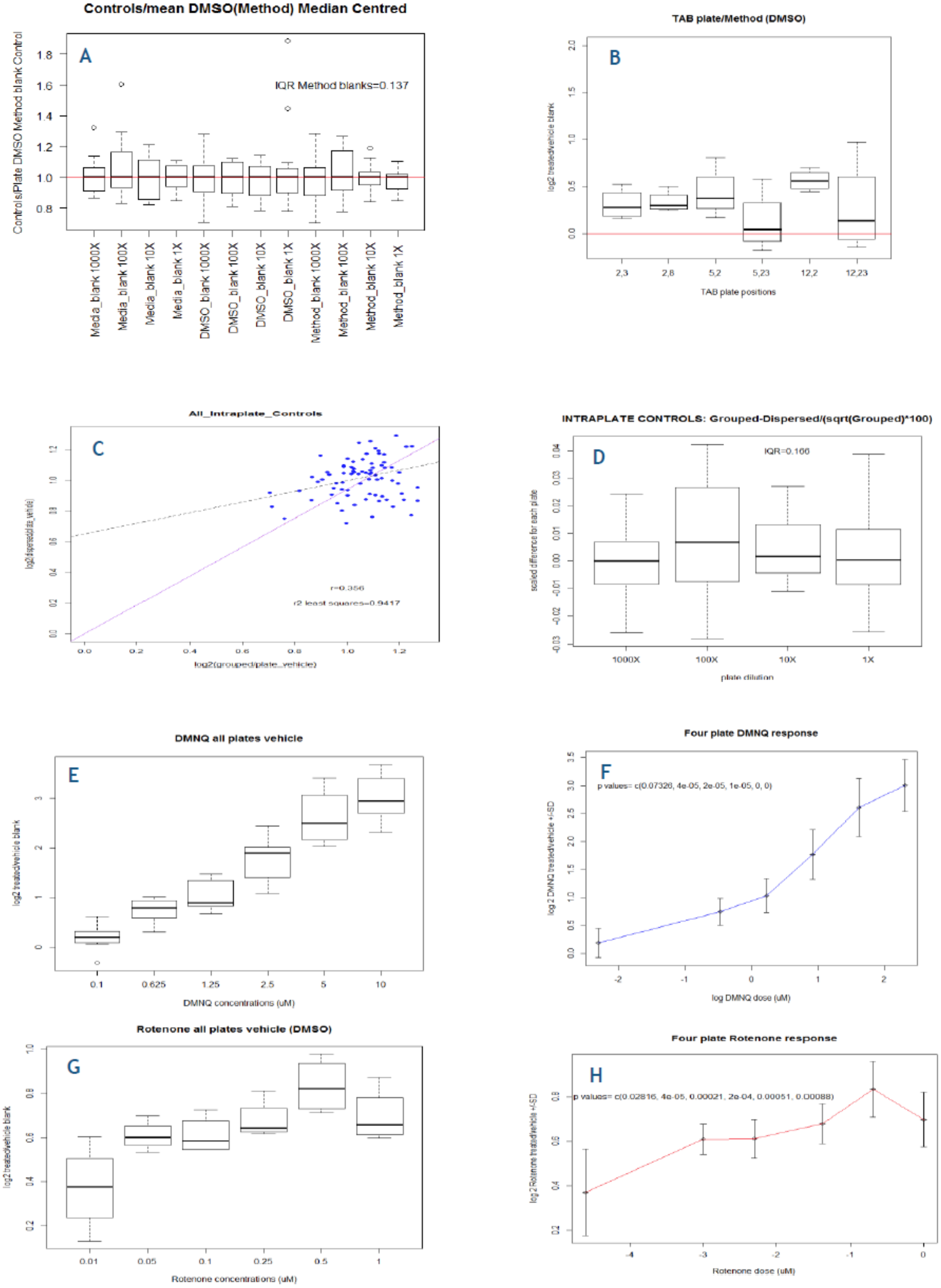

Supplemental File 2. Experiment description and QC data for A375, A549, HepG2, HepaRG, MCF7, HT29, LN229, HLMVEC and SH-SY5Y cells.

3. Apoptosis

- A – controls normalised to method blank. Media (columns 1 to 4, DMSO (columns 5-8) and method blank (columns 9-12). IQR is that for all the methods blanks across the four plates.
- B – TAB controls normalise against method blank for each location (column, row) across the four plates of the assay set
- C – scatter plot of the intraplate controls normalised to the method blank with Pearsons (dashed) and least mean squares plot lines (solid) included
- D – Scaled ratios of the intraplate replicates with the IQR for the values across all four plates included.
- E – Box plot of the mitomycin C response normalised to the method blanks
- F - line plot of the mitomycin C response normalised to the method blank showing SDs and t-tests for each response against the method blanks
- G – boxplot of the hygromycin B response normalised to the method blank
- H – Boxplot of the responses of the single chemicals, left to right, Beta naphthoflavone, etopiside, allylalcohol and rifampicin.

Supplemental File 2. Experiment description and QC data for A375, A549, HepG2, HepaRG, MCF7, HT29, LN229, HLMVEC and SH-SY5Y cells.

3.1. HEPG2 (Apoptosis)

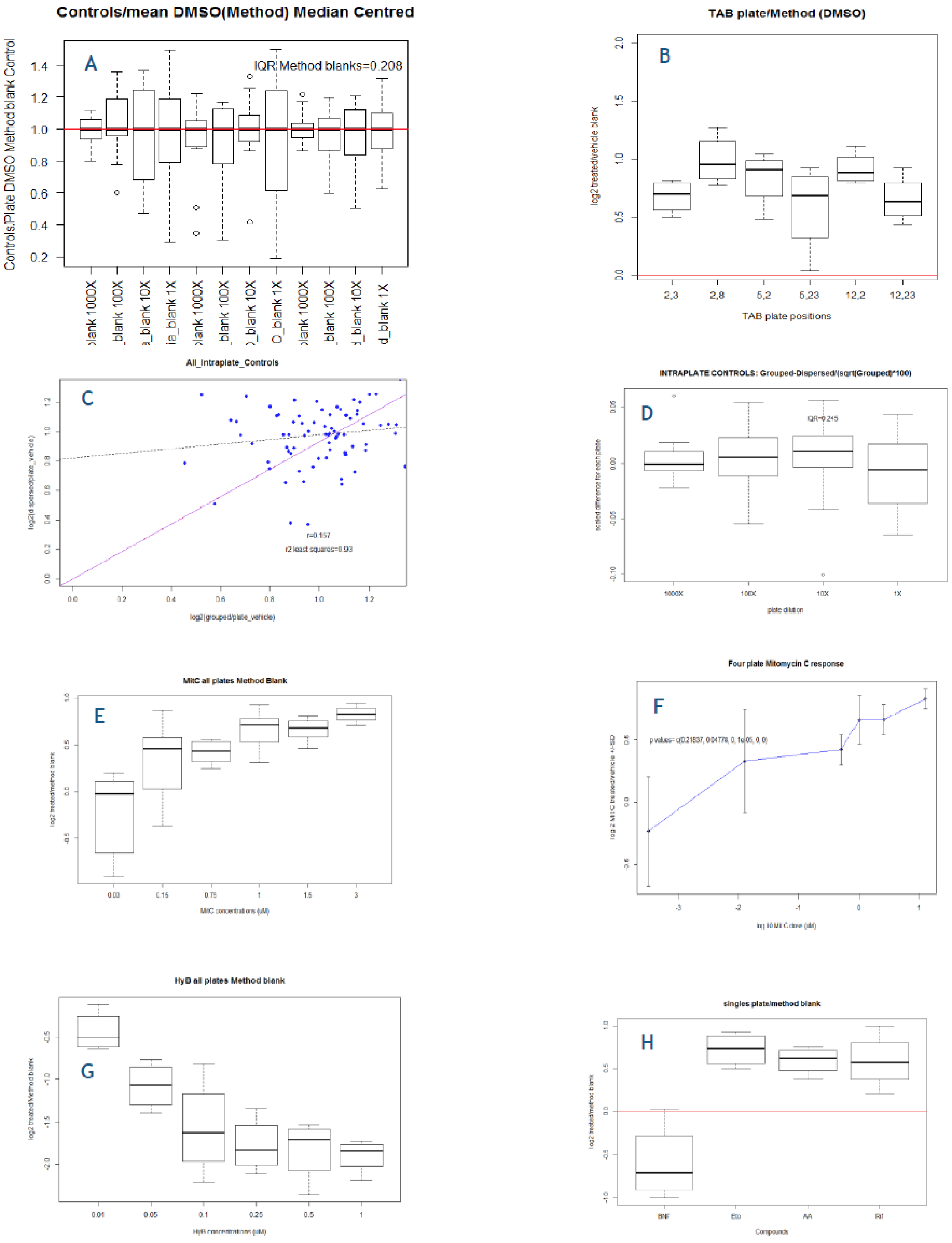

Supplemental File 2. Experiment description and QC data for A375, A549, HepG2, HepaRG, MCF7, HT29, LN229, HLMVEC and SH-SY5Y cells.

3.2. A375 (APOPTOSIS)

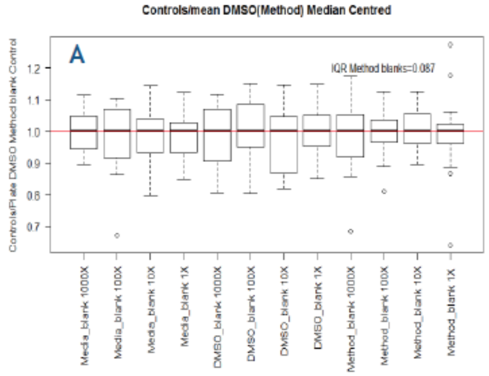

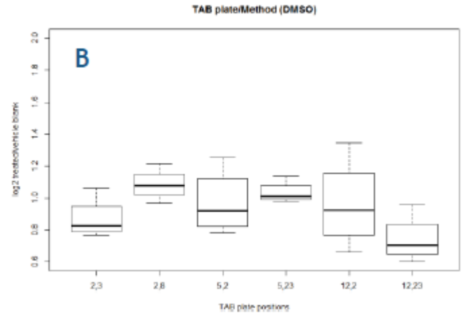

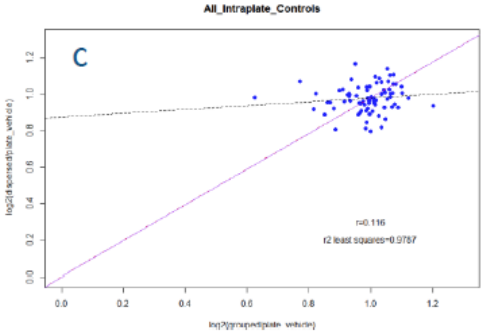

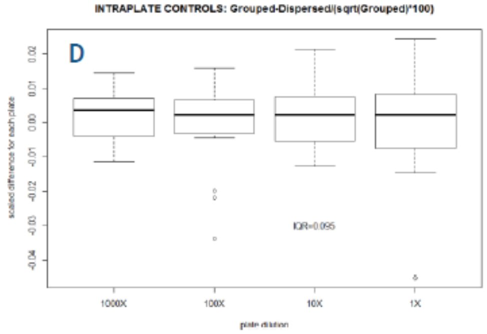

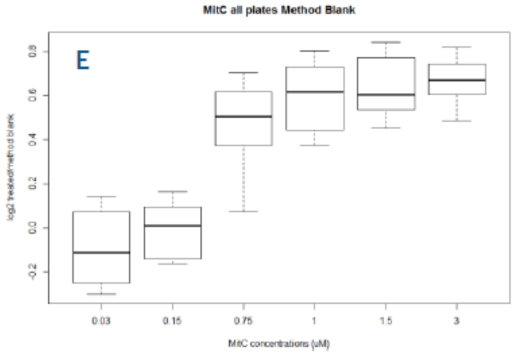

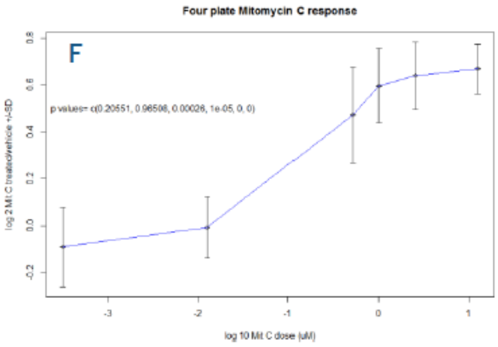

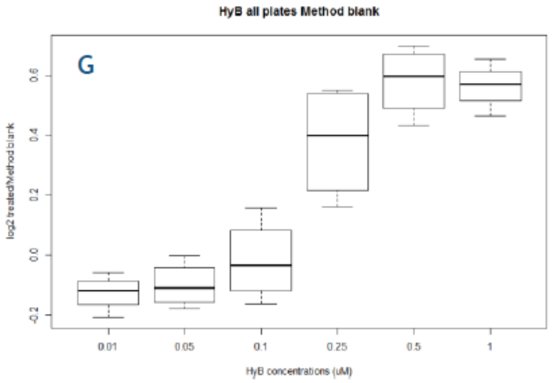

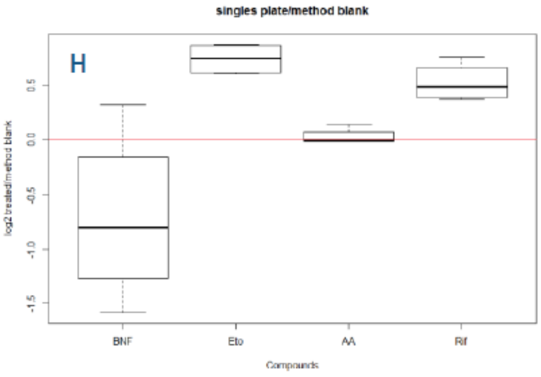

Supplemental File 2. Experiment description and QC data for A375, A549, HepG2, HepaRG, MCF7, HT29, LN229, HLMVEC and SH-SY5Y cells.

3.3. A549 (apoptosis)

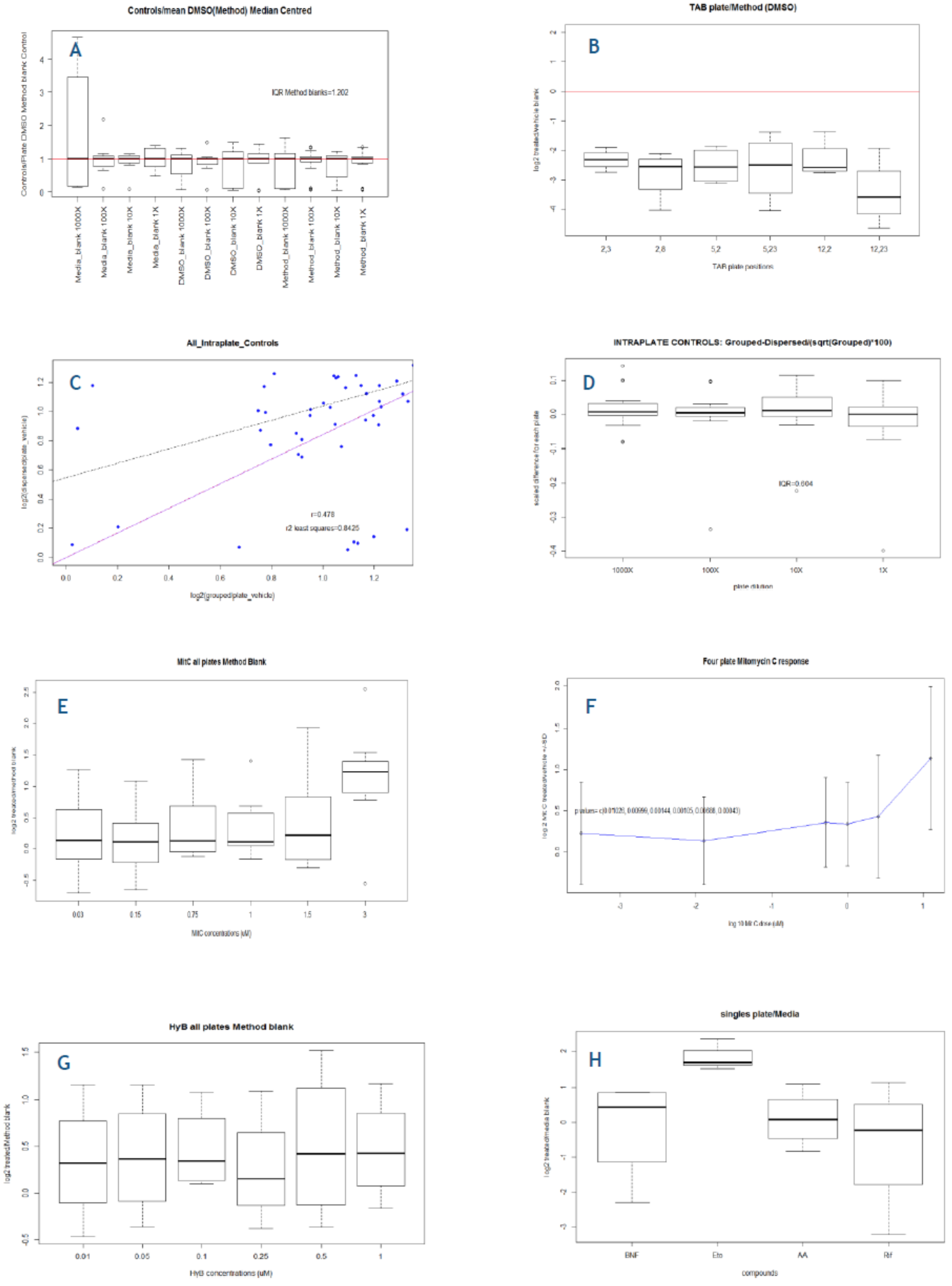

Supplemental File 2. Experiment description and QC data for A375, A549, HepG2, HepaRG, MCF7, HT29, LN229, HLMVEC and SH-SY5Y cells.

3.4. MCF7 (apoptosis)

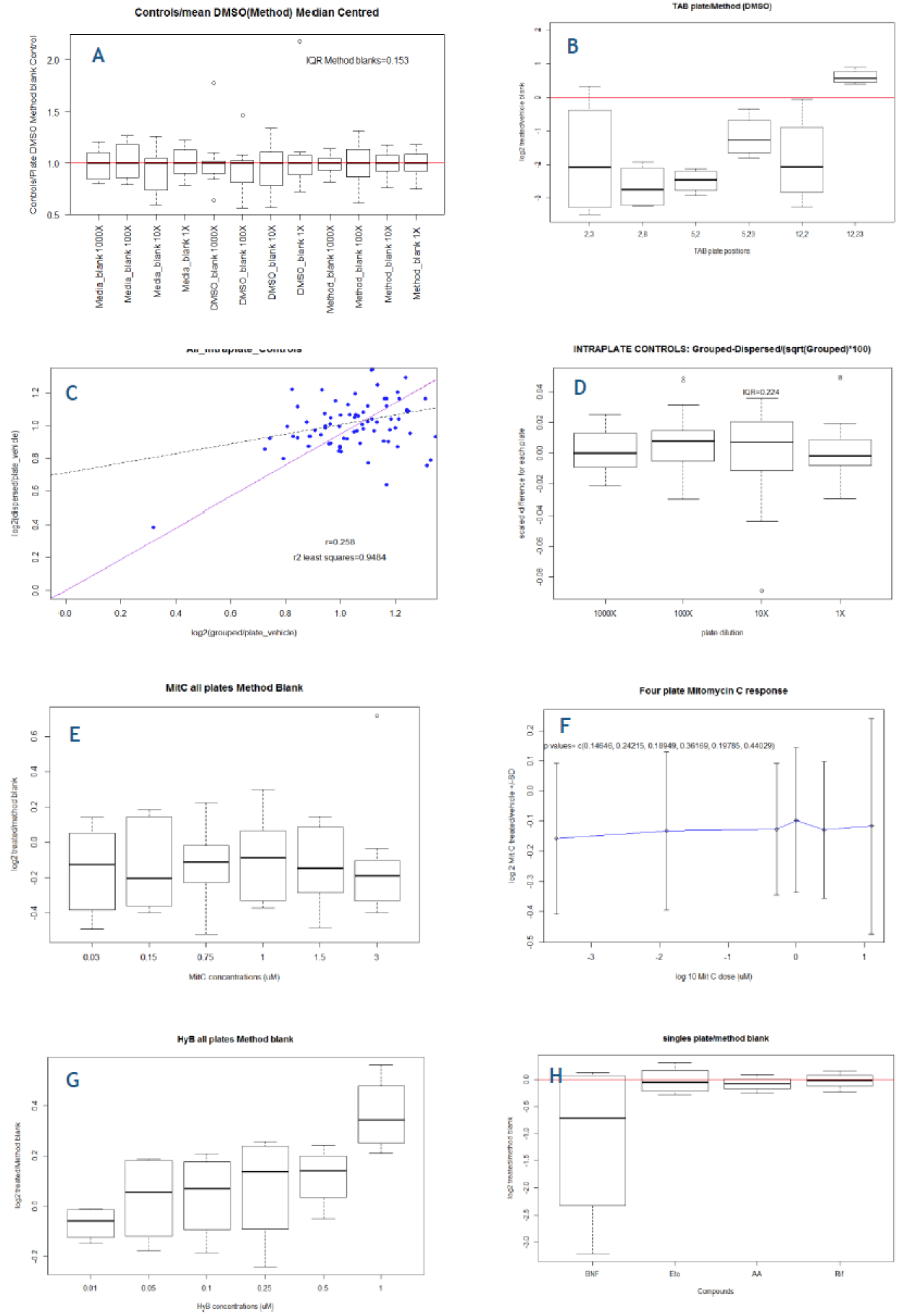

Supplemental File 2. Experiment description and QC data for A375, A549, HepG2, HepaRG, MCF7, HT29, LN229, HLMVEC and SH-SY5Y cells.

3.5. HLMVEC (apoptosis).

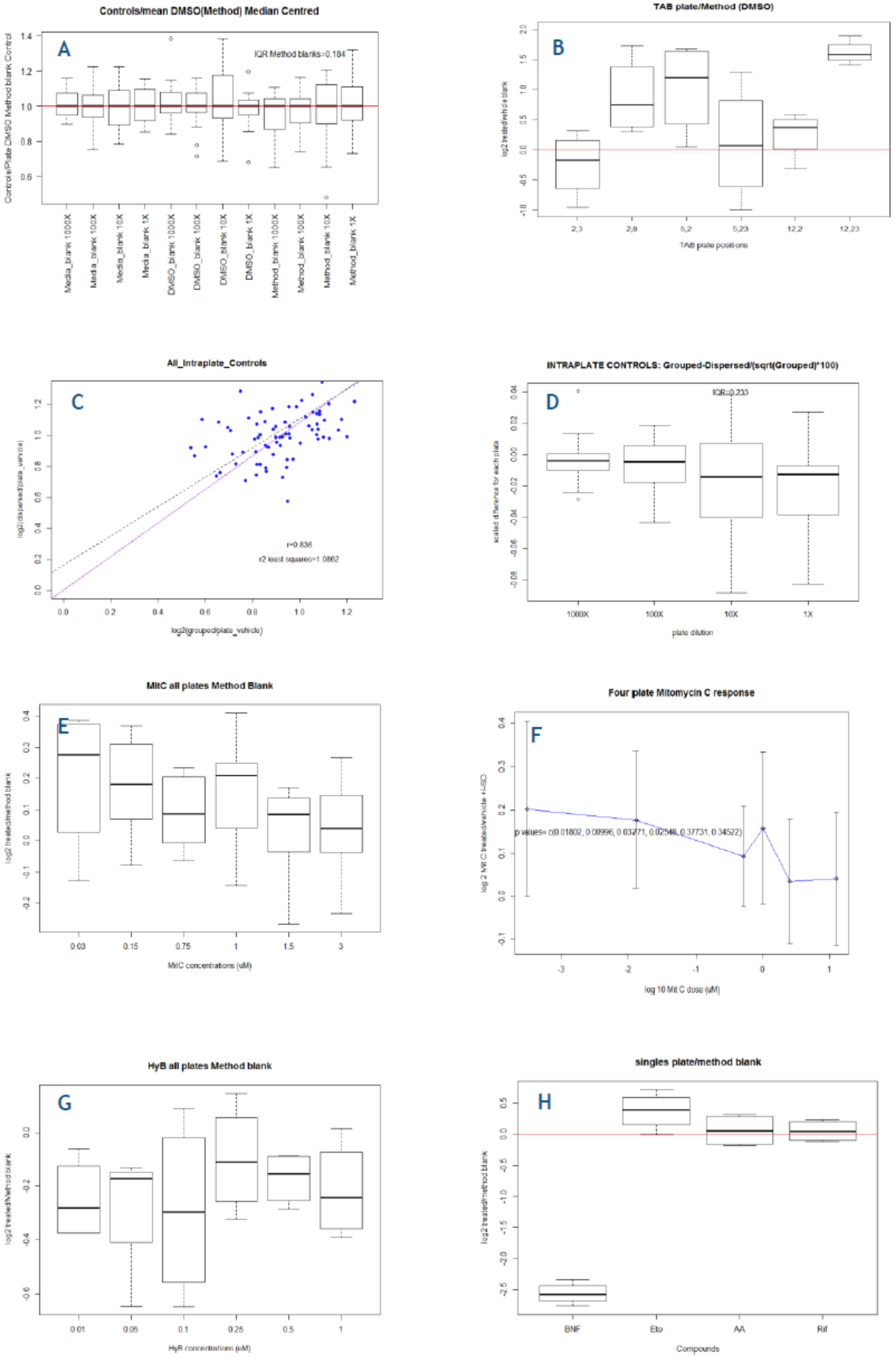

Supplemental File 2. Experiment description and QC data for A375, A549, HepG2, HepaRG, MCF7, HT29, LN229, HLMVEC and SH-SY5Y cells.

3.6. HEPARG (apoptosis)

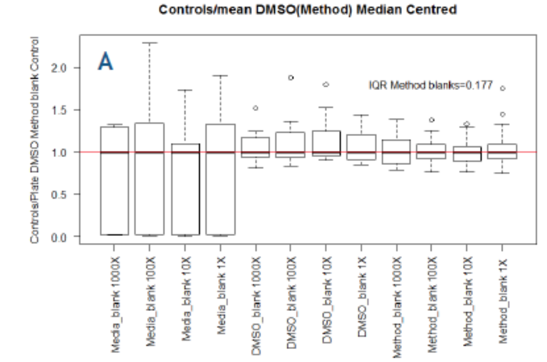

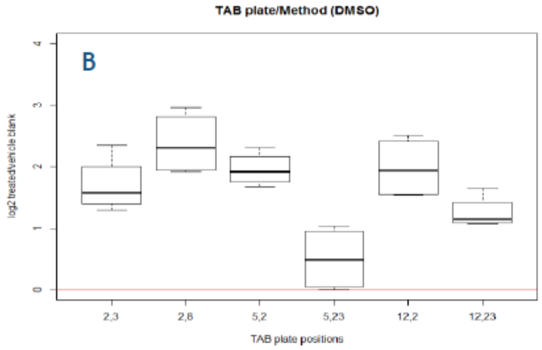

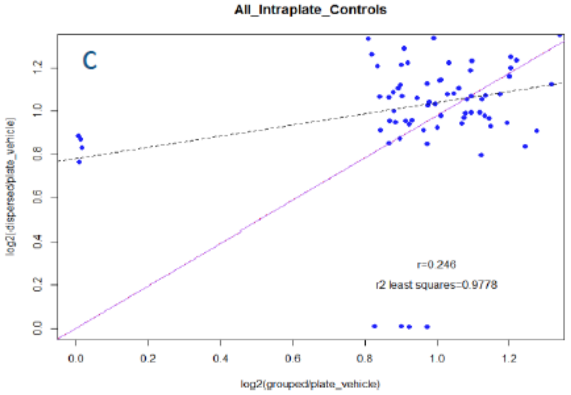

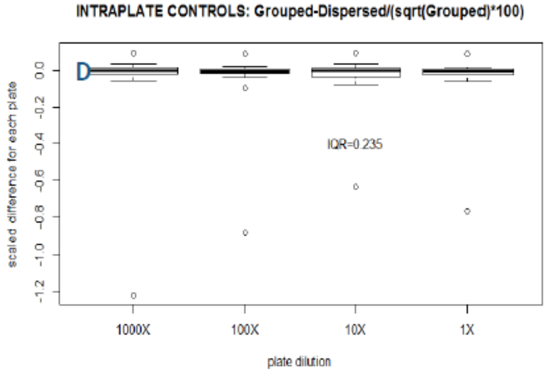

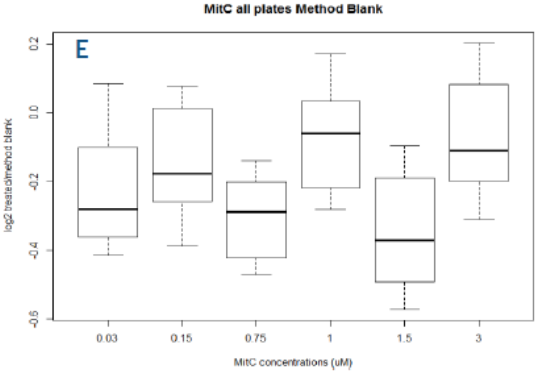

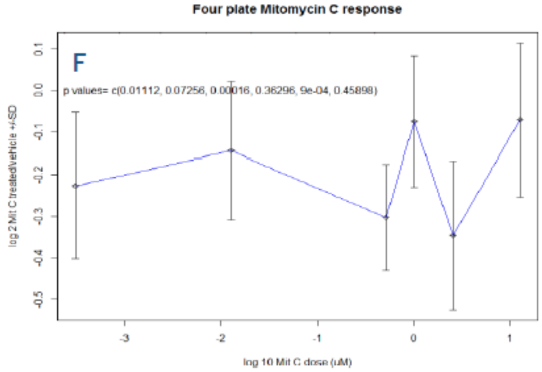

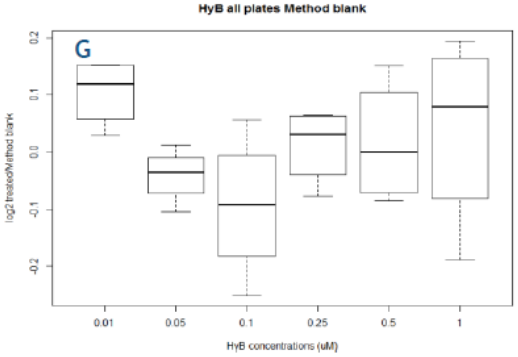

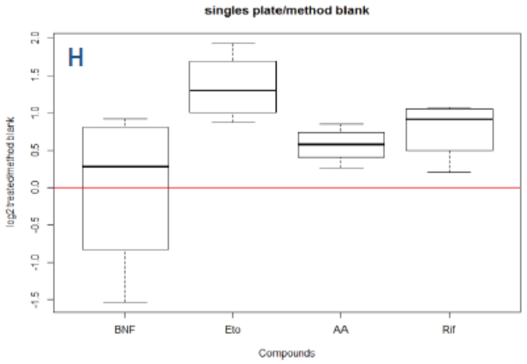

Supplemental File 2. Experiment description and QC data for A375, A549, HepG2, HepaRG, MCF7, HT29, LN229, HLMVEC and SH-SY5Y cells.

3.7. HT29 (apoptosis)

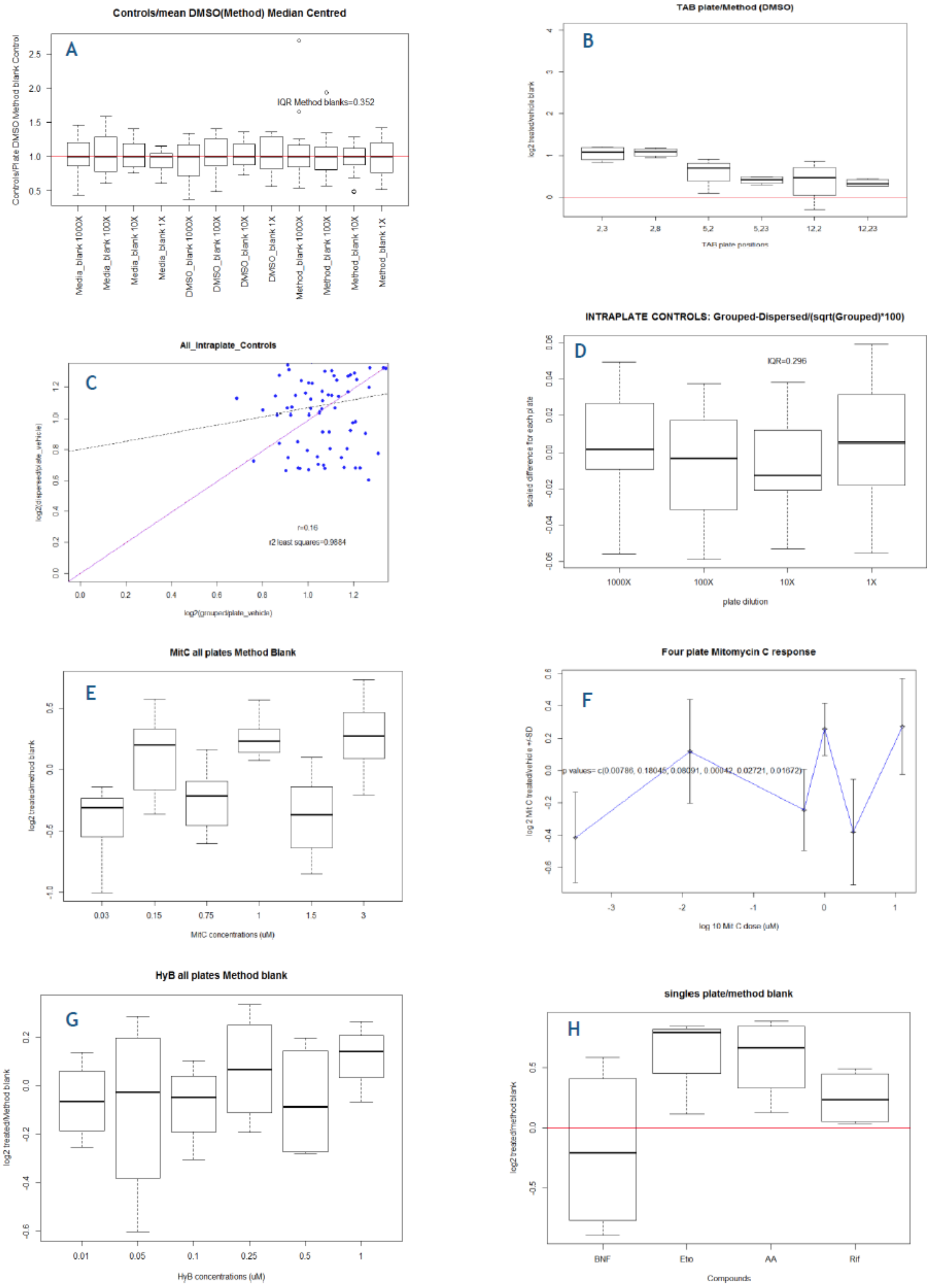

Supplemental File 2. Experiment description and QC data for A375, A549, HepG2, HepaRG, MCF7, HT29, LN229, HLMVEC and SH-SY5Y cells.

3.8. SH-SY5Y (apoptosis)

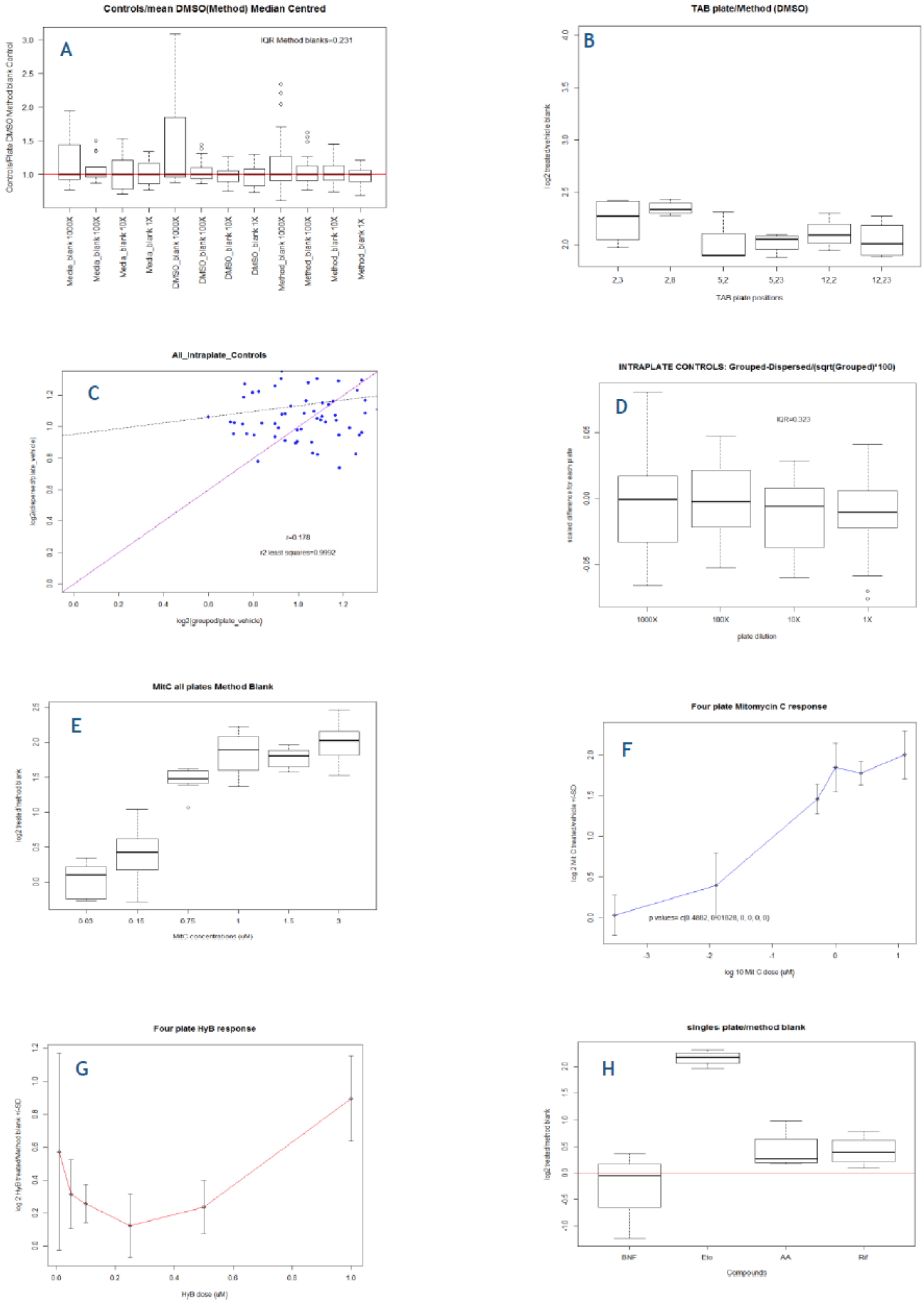

Supplemental File 2. Experiment description and QC data for A375, A549, HepG2, HepaRG, MCF7, HT29, LN229, HLMVEC and SH-SY5Y cells.

3.9. LN229 (apoptosis).

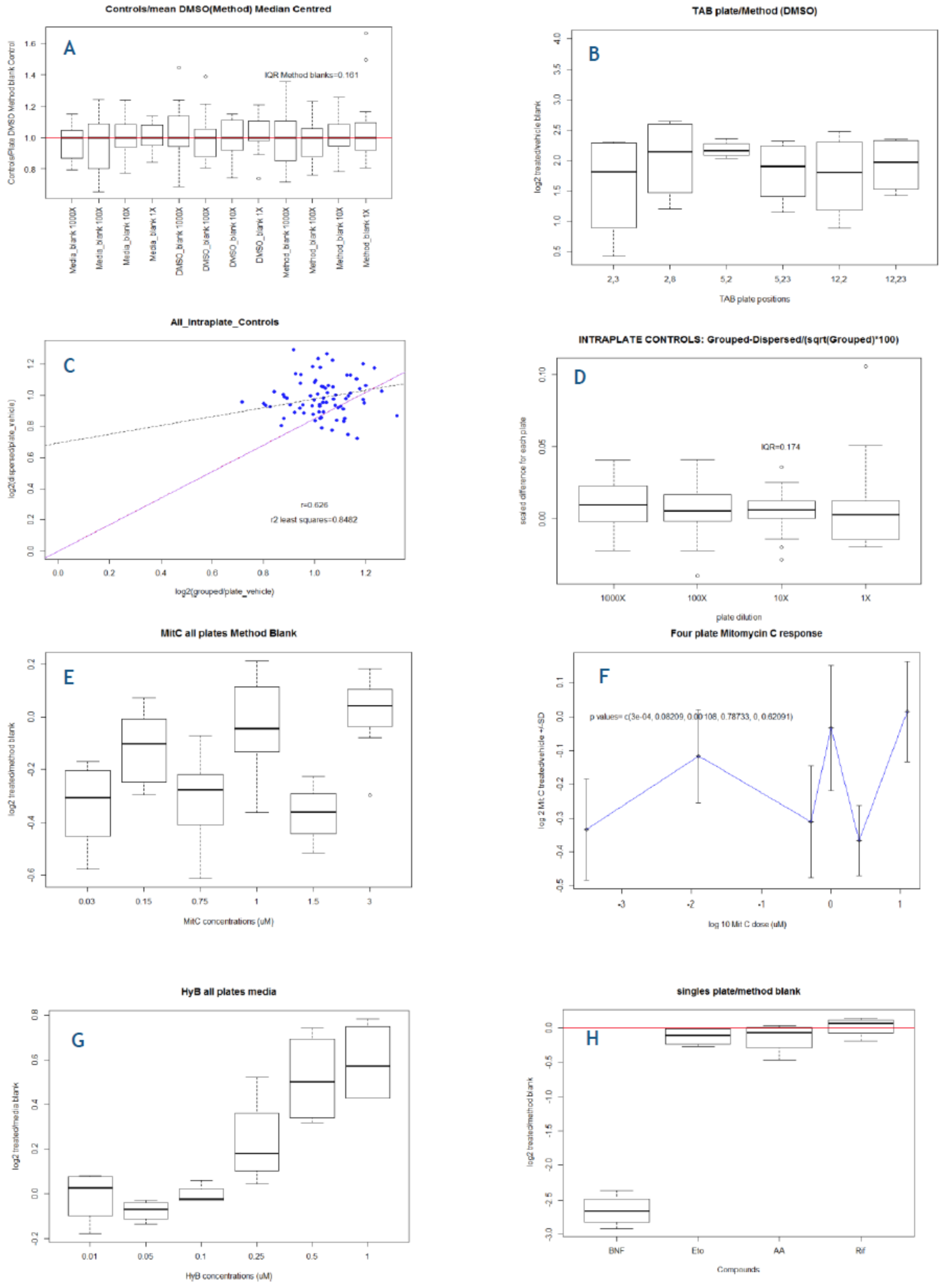

Supplemental File 2. Experiment description and QC data for A375, A549, HepG2, HepaRG, MCF7, HT29, LN229, HLMVEC and SH-SY5Y cells.

4. ATP

- A – controls normalised to method blank. Media (columns 1 to 4, DMSO (columns 5-8) and method blank (columns 9-12). IQR is that for all the methods blanks across the four plates.
- B – TAB controls normalise against method blank for each location (column, row) across the four plates of the assay set
- C – scatter plot of the intraplate controls normalised to the method blank with Pearsons (dashed) and least mean squares plot lines (solid) included
- D – Scaled ratios of the intraplate replicates with the IQR for the values across all four plates included.
- E – Box plot of the rotenone response normalised to the method blanks
- F - line plot of the rotenone response normalised to the method blank showing SDs and t-tests for each response against the method blanks
- G – line plot of the hygromycin B response normalised to the method blank showing SDs and t-tests for each response against the method blanks
- H – line plot of the DMNQ response normalised to the method blank showing SDs and t-tests for each response against the method blanks

Supplemental File 2. Experiment description and QC data for A375, A549, HepG2, HepaRG, MCF7, HT29, LN229, HLMVEC and SH-SY5Y cells.

4.1. HEPG2 (ATP)

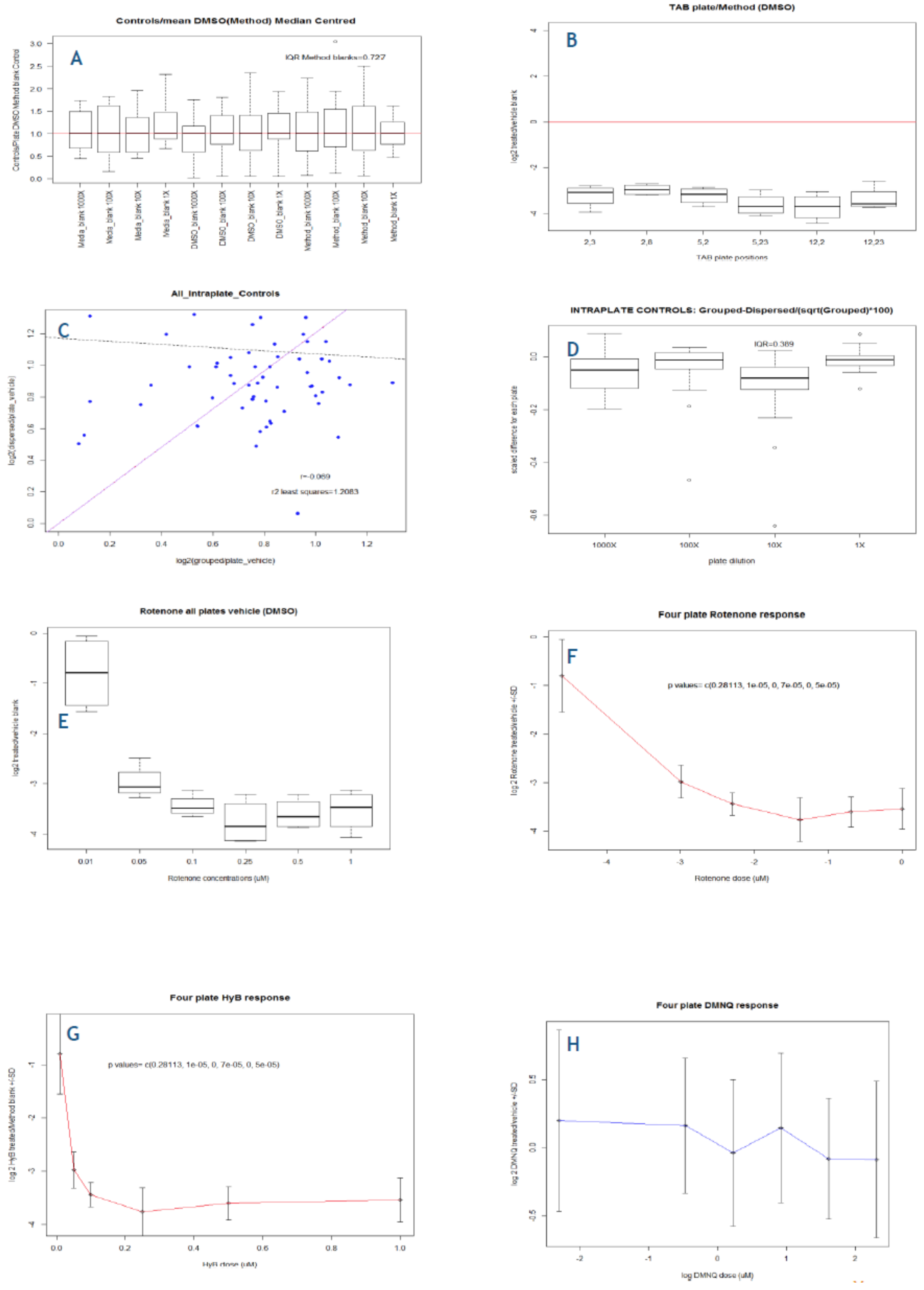

Supplemental File 2. Experiment description and QC data for A375, A549, HepG2, HepaRG, MCF7, HT29, LN229, HLMVEC and SH-SY5Y cells.

4.2. A375 (ATP)

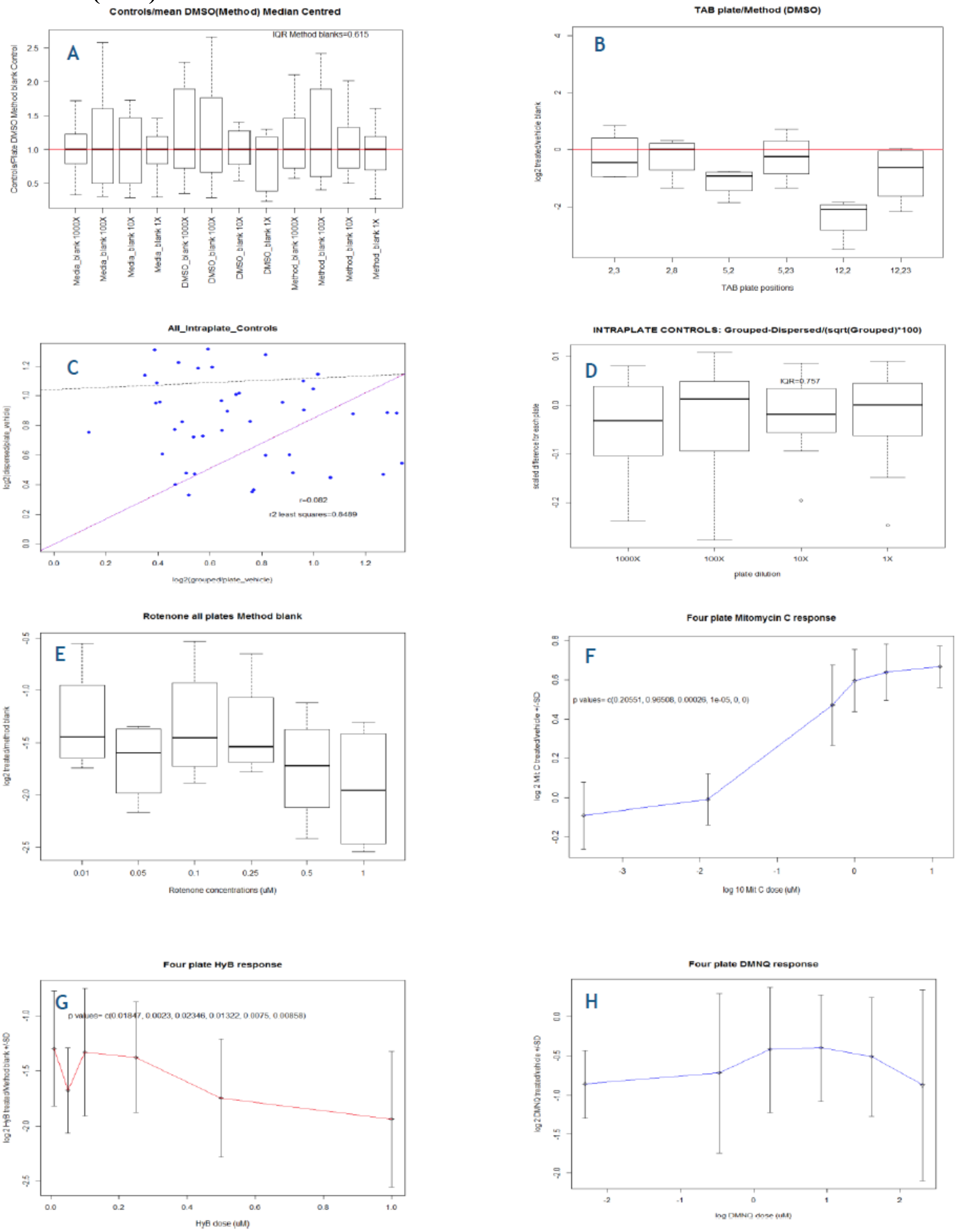

Supplemental File 2. Experiment description and QC data for A375, A549, HepG2, HepaRG, MCF7, HT29, LN229, HLMVEC and SH-SY5Y cells.

4.3. A549 (ATP)

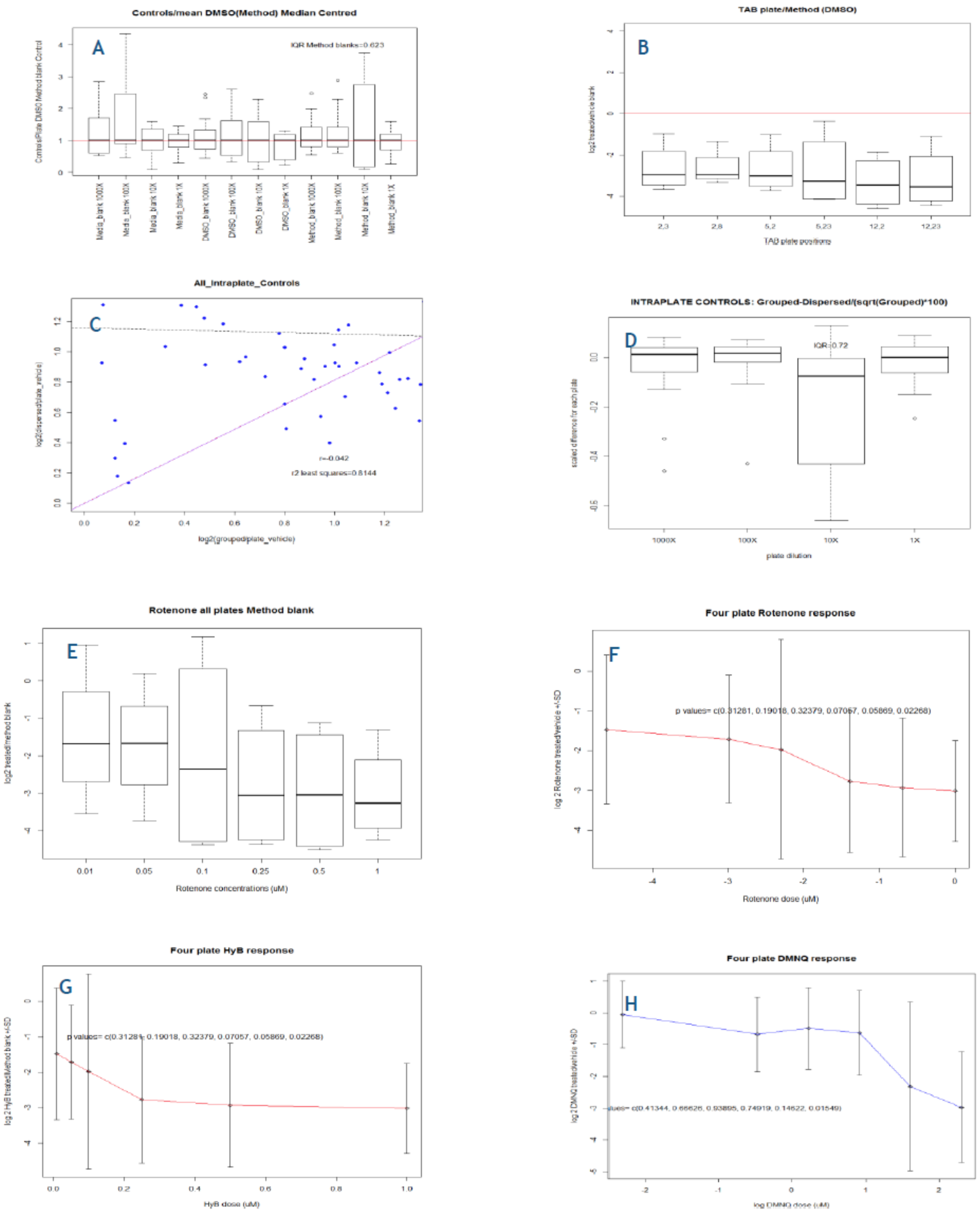

Supplemental File 2. Experiment description and QC data for A375, A549, HepG2, HepaRG, MCF7, HT29, LN229, HLMVEC and SH-SY5Y cells.

4.4. MCF7 (ATP)

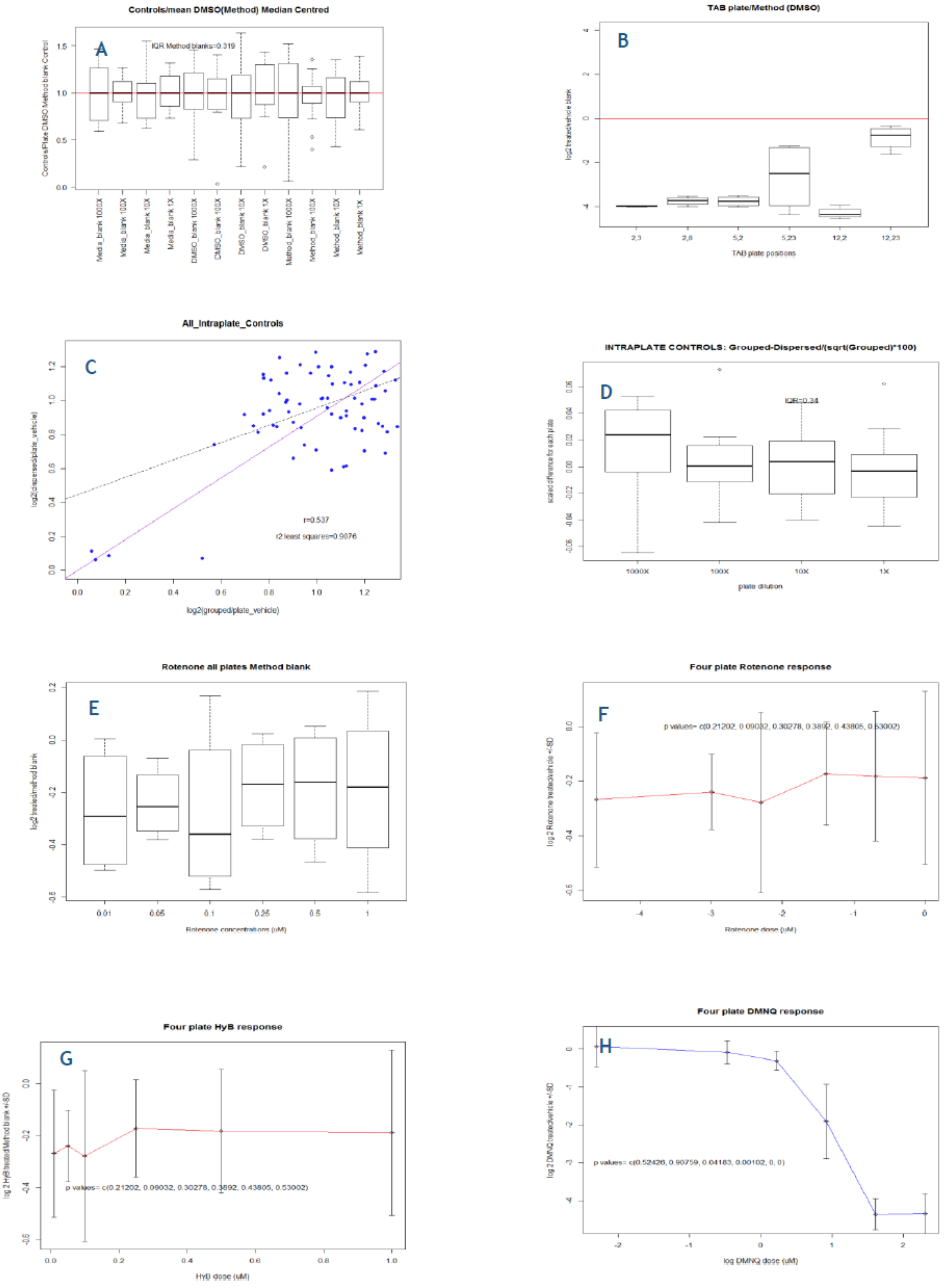

Supplemental File 2. Experiment description and QC data for A375, A549, HepG2, HepaRG, MCF7, HT29, LN229, HLMVEC and SH-SY5Y cells.

4.5. HLMVEC (ATP)

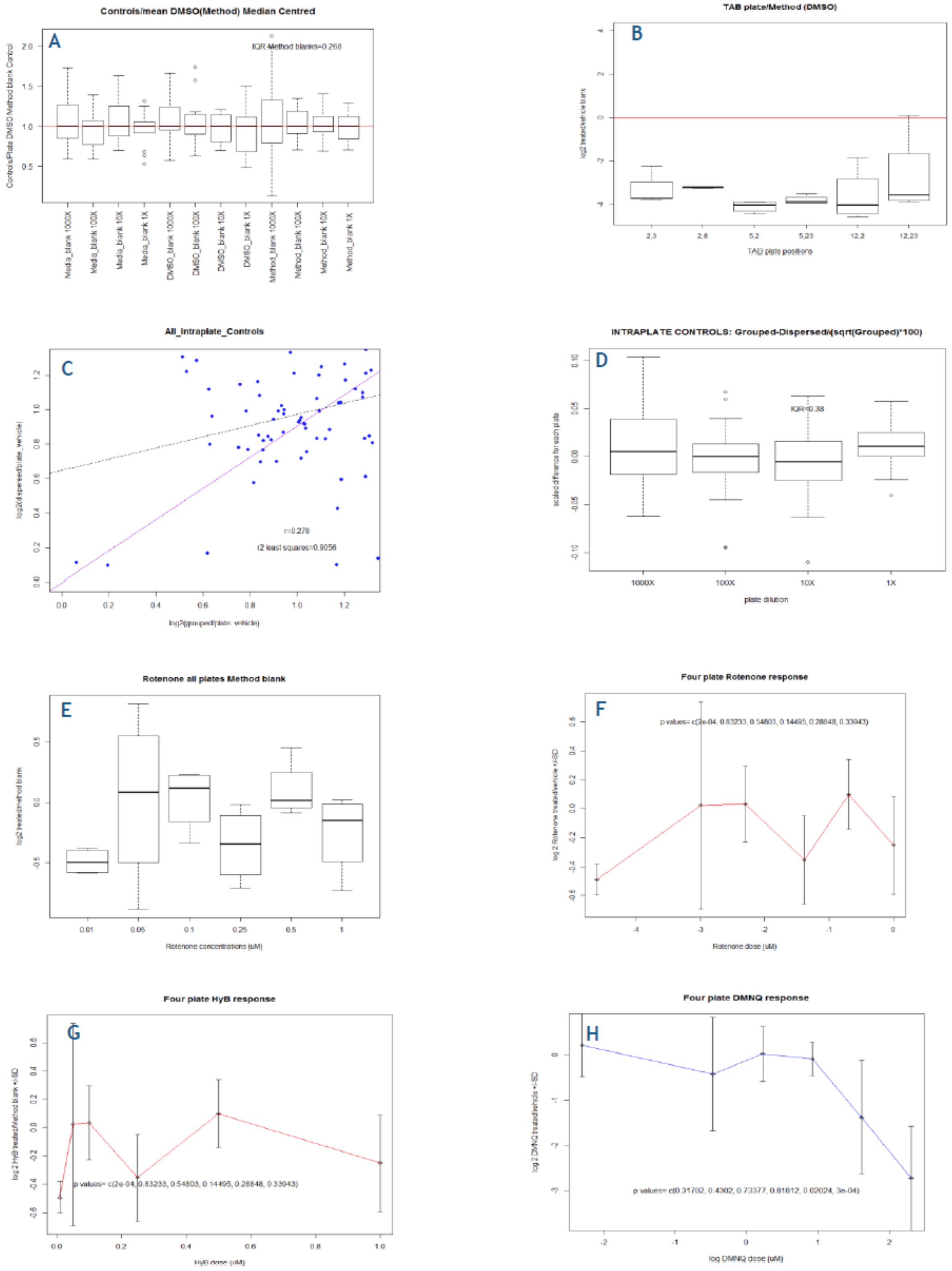

Supplemental File 2. Experiment description and QC data for A375, A549, HepG2, HepaRG, MCF7, HT29, LN229, HLMVEC and SH-SY5Y cells.

4.6. HEPARG (ATP)

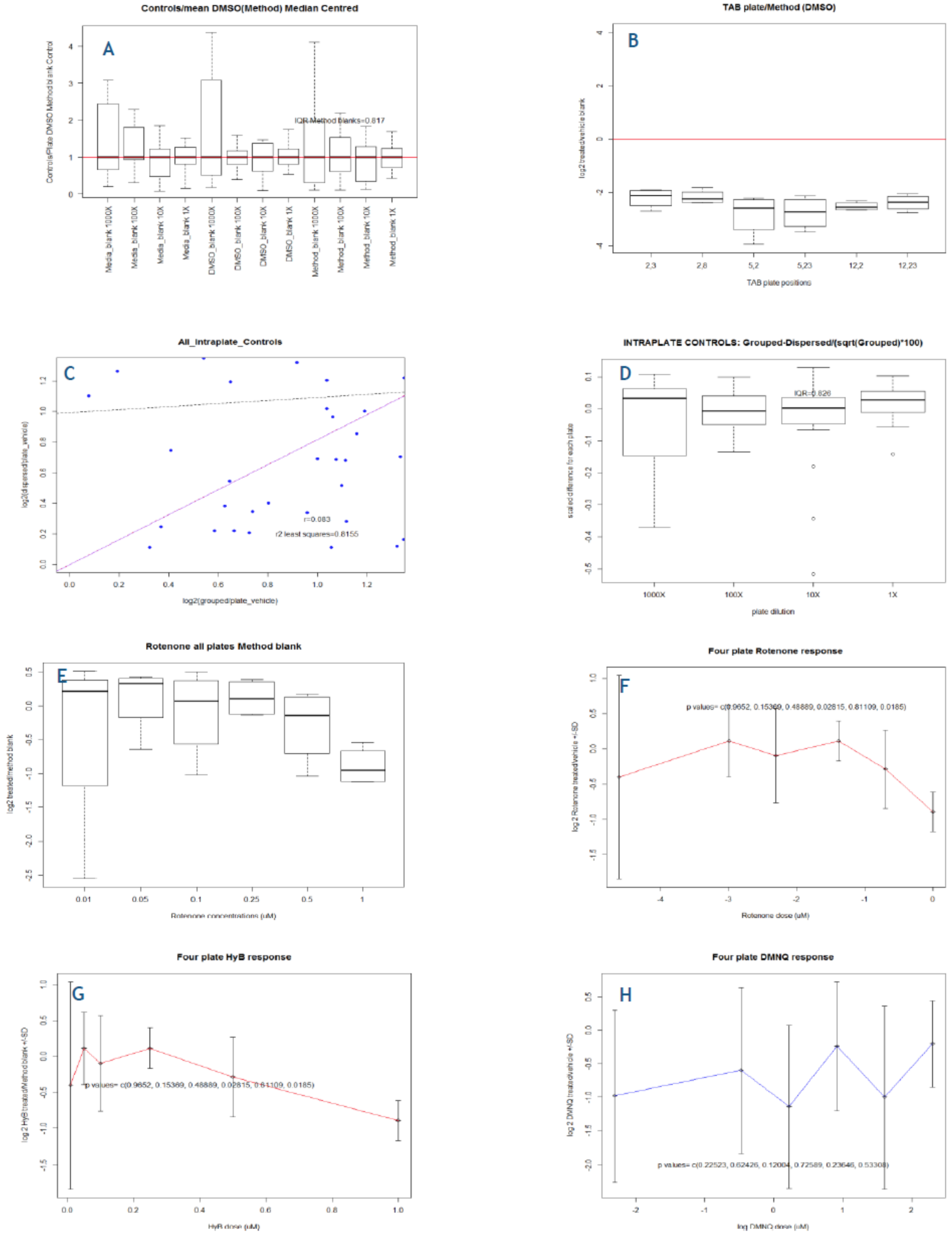

Supplemental File 2. Experiment description and QC data for A375, A549, HepG2, HepaRG, MCF7, HT29, LN229, HLMVEC and SH-SY5Y cells.

4.7. HT29 (ATP)

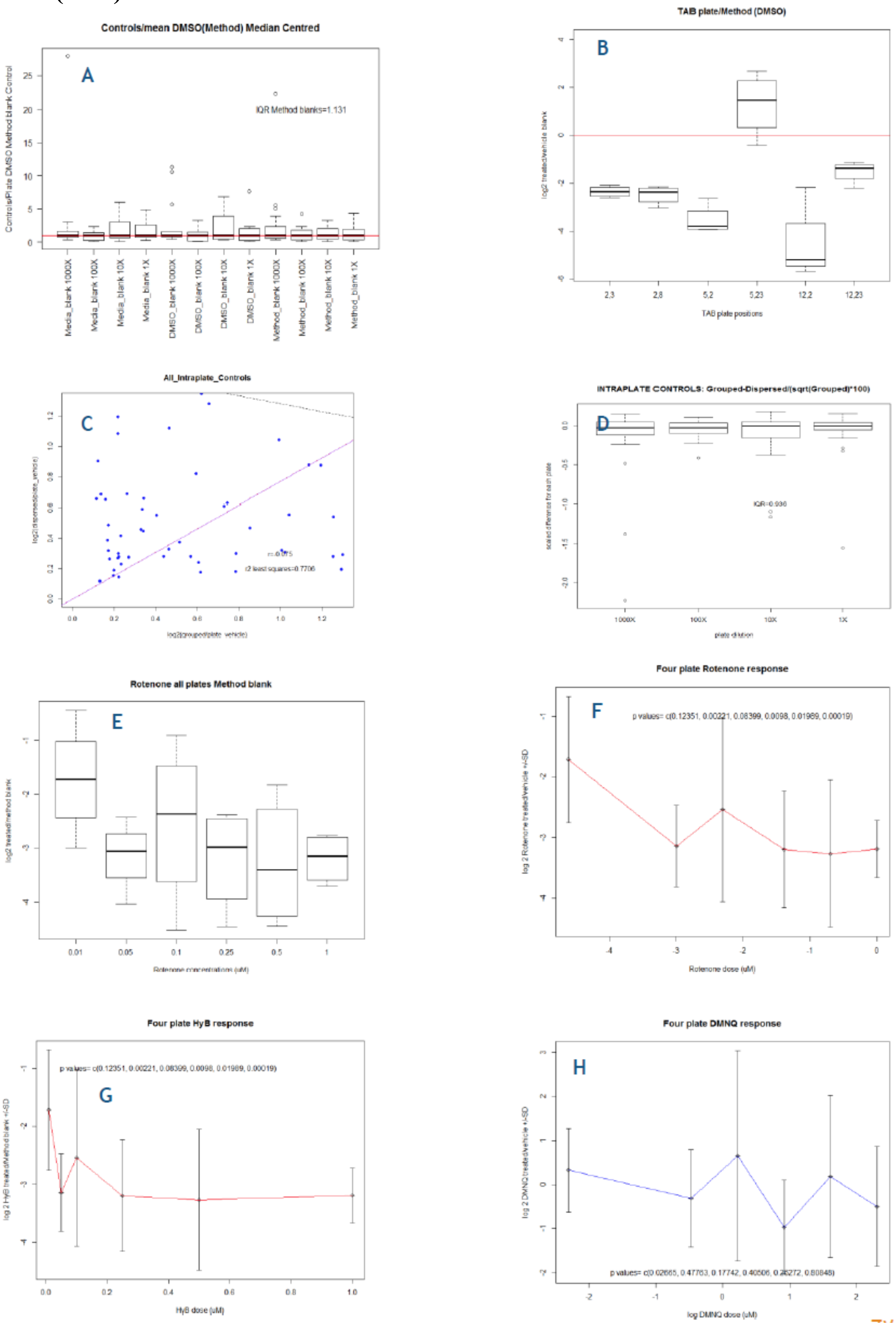

Supplemental File 2. Experiment description and QC data for A375, A549, HepG2, HepaRG, MCF7, HT29, LN229, HLMVEC and SH-SY5Y cells.

4.8. SH-SY5Y (ATP)

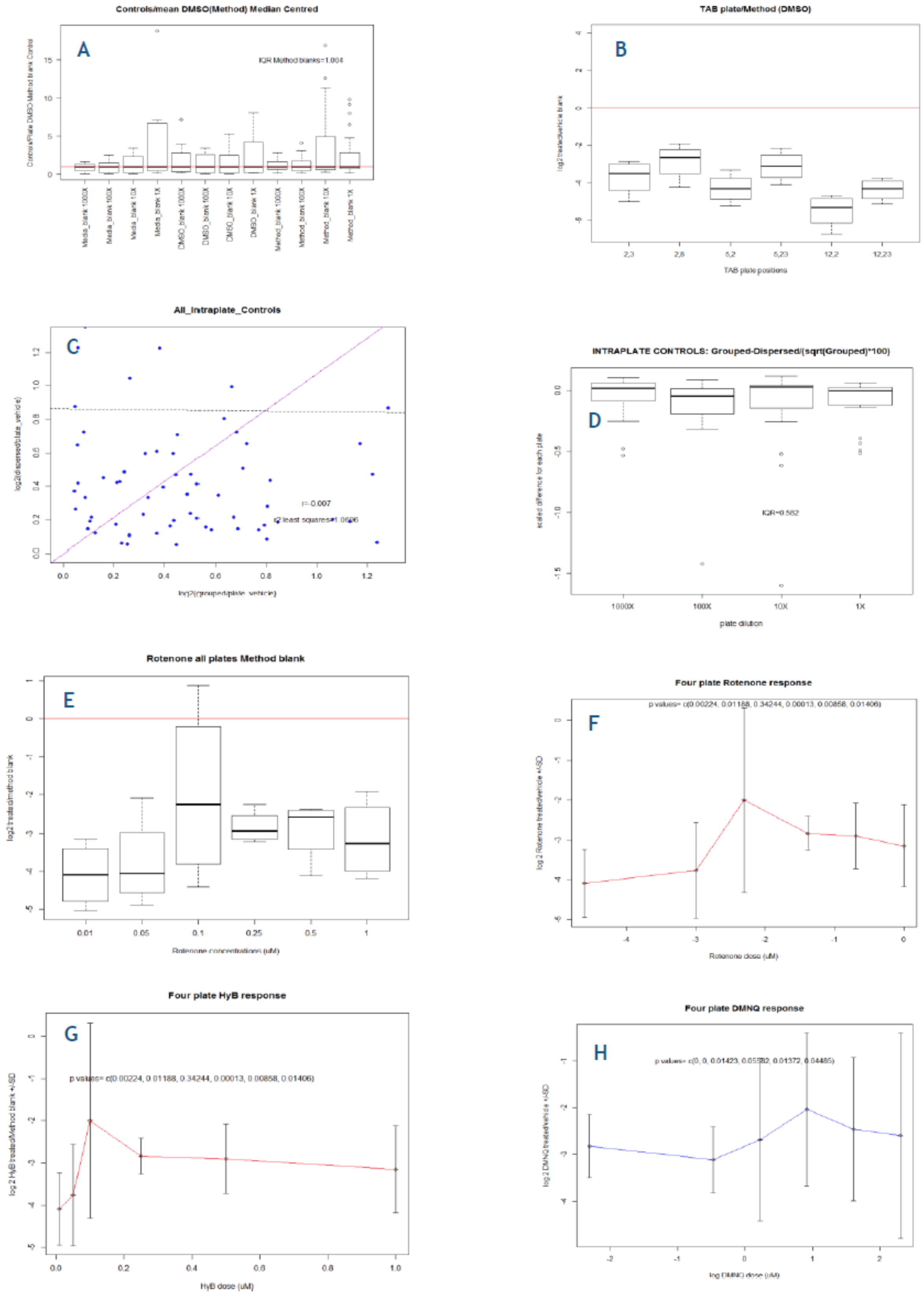

Supplemental File 2. Experiment description and QC data for A375, A549, HepG2, HepaRG, MCF7, HT29, LN229, HLMVEC and SH-SY5Y cells.

4.9. LN229 (ATP)

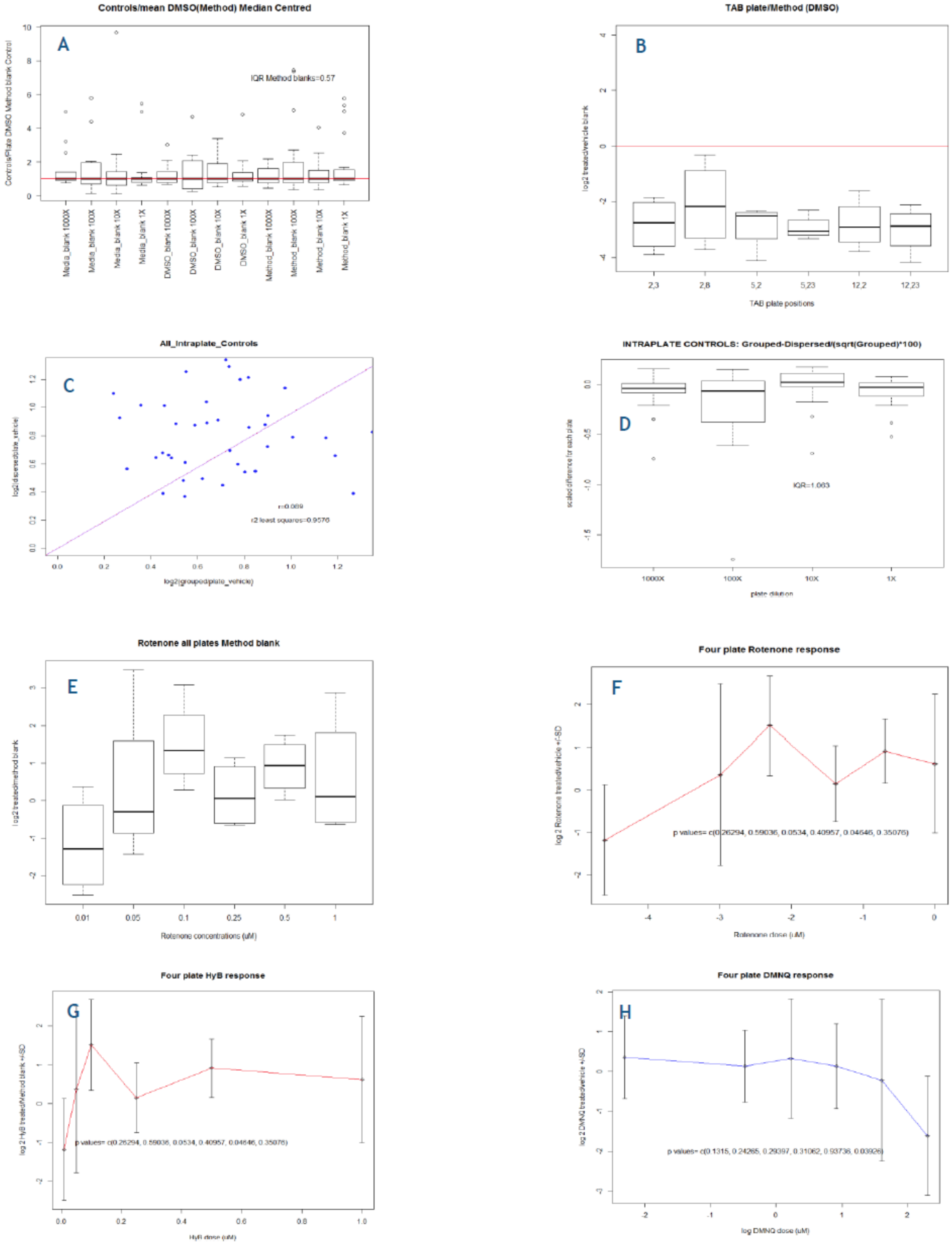

Supplement: Supplemental File 2 [file NIHMS1670764-supplement-Supplemental_File_2.pdf]
