# Supplementary material for: Grouping of UVCB Substances with New Approach Methodologies (NAMs) Data
Source: ALTEX. Author manuscript; Available in PMC 2021 Feb 23. (PMC7900923; doi:10.14573/altex.2006262)

Supplemental File 1. Experiment description, QC data and ToxPI examples for iPSCs and HUVECs.

1. HEPATOCYTES

The liver and its constituent cell, the hepatocyte, play a crucial role in metabolizing exogenous and endogenous agents and are vitally important in understanding the risk or hazard posed by various chemicals. In vitro systems for hepatocytes have included cell lines of cancerous origin as well as primary cells from various species which each have their benefits and disadvantages. With recent advances in stem cell technology, new human stem cell-derived models that better represent the underlying biology are being produced. Their incorporation into toxicity testing is already taking place with several recent publications. [6,1] , Incorporation of this type of cell into the larger chemical-biological grouping approach provides essential biology not present in other tissues as well as being more easily understood by toxicologists and regulators.

1.1. iCELL HEPATOCYTES 2.0

Cellular Dynamics is a leading producing of induced pluripotent stem cell (iPSC)-derived cells, in particular hepatocytes. The iPSC-derived hepatocytes provide phenotypic stability and relevant hepatic function, specifically the expression of cytochrome P450 enzymes essential for xenobiotic metabolism. In addition, they express relevant clinical targets for liver and metabolic diseases as well as being highly quality controlled to ensure consistency over different experiments or screens.

1.1.1. Basal and Altered Morphology

Hepatocytes in culture form a single monolayer with the boundary between cells clearly visible. After plating and several days of culturing, the iCell Hepatocytes 2.0 take on the morphology of mosaic pattern as would be expected for hepatocytes, as seen below. The morphology can be altered with exposure to chemicals indicating changes in overall cellular health, also see below. This aspect is utilized when defining the phenotypic endpoints as those used in the screen.

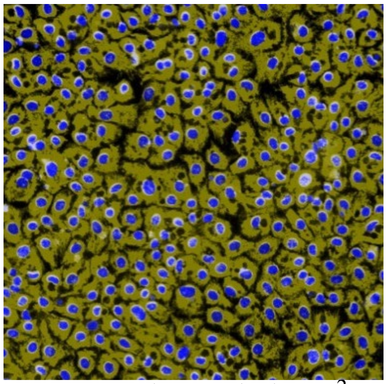

Basal Morphology:[2]

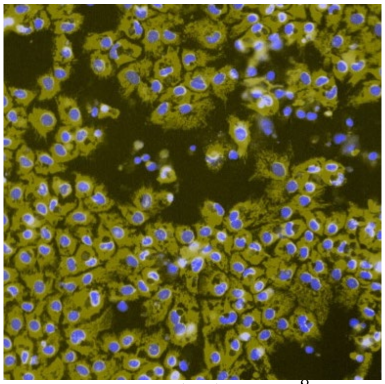

10 nM Amiodarone:[8]

[1] Grimm FA, Iwata Y, Sirenko O, Bittner M, Rusyn I. "High-Content Assay Multiplexing for Toxicity Screening in Induced Pluripotent Stem Cell-Derived Cardiomyocytes and Hepatocytes." *Assay Drug Dev Technol.* 2015, 13(9), 529-46.

[2] Nuclei are indicated in Blue using Hoescht 33342 nuclear stain and Mitochondria are indicated in yellow using Mitotracker fluorescent stain.

1.1.2. Hepatocyte-Specific Positive Controls

Doxorubicin

CasRN: 25316-40-9 Supplier: Sigma

Top Concentration: 10 µM (0.5% DMSO)

Doxorubicin is a chemotherapeutic agent of the anthracycline class which is commonly used for a wide range of cancers including breast, bladder and several lymphatic cancers. A side effect sometimes associated with this compound is hepatotoxicity and clear liver lesions have been observed in rats exposed to Doxorubicin [3,4]. It was used here as a positive control for hepatotoxicity.

Amiodarone

CasRN: 19774-82-4 Supplier: Sigma

Top Concentration: 100 µM (0.5% DMSO)

Amiodarone is a therapeutic used for addressing heart arrhythmia with known hepatotoxicity with long term exposures [5]. Amiodarone-associated hepatotoxicity appears to result from alterations in lipid bilayers of both the cell membrane and mitochondrial membranes. Its inclusion in this screen is for this hepatocyte-specific effect.

Rotenone

CasRN: 83-79-4 Supplier: MP Biomedicals

Top Concentration: 100 µM (0.5% DMSO)

Rotenone is a commonly used insecticide and pesticide with some associated human toxicity. This compound exerts its toxic effects by inhibiting mitochondrial respiration leading to oxidative stress and overall decreases in cellular health [6]. Rotenone was included as a positive control for mitochondrial dysfunction and for general cytotoxicity.

Carbonyl cyanide m-chlorophenyl hydrazine (CCCP)

CasRN: 555-60-2 Supplier: Tocris

Top Concentration: 50 µM (0.5% DMSO)

Carbornyl cyanide m-chlorophenyl hydrazide (CCCP) is a known toxic agent which directly acts on the mitochondria through inhibition of oxidative phosphorylation. This is achieved through multiple mechanisms, mainly the uncoupling of the proton gradient [7]. CCCP was included in this screen for its effects on the mitochondria and general cytotoxic effects.

[3] Damodar G, Smitha T, Gopinath S, Vijatakumar S, Rao YA. "An evaluation of hepatotoxicity in breast cancer patients receiving injection doxorubicin." *Ann Med Health Sci Res*. 2014, 4(1), 74-79.

[4] El-Sayyad HI, Ismail MF, Shalaby FM, Abou-El-Magd RF, Gaur RL, Fernando A, Raj MHG, Ouhtit A. "Histopathological effects of cisplatin, doxorubicin and 5-flurouracil (5-FU) on the liver of male albino rats." *Int J Biol Sci.* 2009, 5(5), 466-473.

[5] Babatin M, Lee SS, Pollak PT. "Amiodarone hepatotoxicity." *Curr Vasc Pharmacol*. 2008, 6(3), 228-36.

[6] Rada K, Rausch WD, Gille G. "Rotenone induces cell death in primary dopaminergic culture by increasing ROS production and inhibiting mitochondrial resipiration." *Neurochem Int*. 2006, 49(4), 379-386.

[7] Burkhardt JK, Argon Y. "Intracellular transport of the glycoprotein of VSV is inhibited by CCCP at a late stage of post-translational processing." *J Cell Sci*. 1989, 92(4), 633-642.

Acetaminophen

CasRN: 103-90-2 Supplier: Cayman Chemical

Top Concentration: 10 mM (0.5% DMSO)

Acetaminophen is a non-steroidal anti-inflammatory drug (NSAID) which is sold over the counter as a pain reliever and fever reducer. Acetaminphen is known to cause hepatotoxicity by a reactive metabolite that produces oxidative stress [8]. This agent was included for the hepatocyte-specific effects associated with it.

Tetraoctylammonium Bromide (TAB)

CasRN: 14866-33-2 Supplier: Sigma

Top Concentration: 50 µM (0.5% DMSO)

Tetraoctylammonium bromide is a compound which consists of long aliphatic chains connected to an ammonium ion. TAB is commonly used in the surface coating of nanoparticles as the aliphatic chains impart specific characteristics onto the nanoparticle [9]. The long aliphatic chains have also been suggested to disrupt the cellular membrane which can serve as a reproducible and useful cytotoxic control. It was included here as a non-cellular specific cytotoxic control.

1.2. SCREEN SPECIFIC INFORMATION

Cell Manufacturer: Cellular Dynamics
Cell line Catalogue Number: R1027 (formerly PHC-100-020-001)
Cell Lot Number: 7000716
Date of Receipt of Cells at Texas A&M University: 6-20-17
Date Screening was performed: 8-2-17 to 8-16-17
Instrumentation used to record high-content imaging phenotypes: ImageXpress® Microconfocal High Content Imaging System (Molecular Devices, Sunnyvale, CA)

1.3. HEPATOCYTE ENDPOINTS FOR BIOACTIVITY PROFILING

High-throughput, live cell imaging was used to assess multiple of parameters of cell health and viability. Cells were selectively stained with different fluorescent probes, and then fluorescent signals were captured in an image by the ImageXpress ® System. The position and intensity of the signals were processed by software to generate numerical data that describe general cell viability/toxicity and certain cell-specific functions in response to chemical treatment.

---

[8] Casarett LJ, Klaassen CD, Doull J. *Casarett and Doull's Toxicology: The Basic Science of Poisons*. 8th ed. New York: McGraw-Hill, Health Professions Division; 1996, 650-653.

[9] Zhang Y, Newton B, Lewis E, Fu PP, Kafoury R, Ray PC, Yu H. “Cytotoxicity of organic surface coating agents used for nanoparticles synthesis and stability.” *Toxicol In Vitro*. 2015, 29(4), 762-768.

Supplemental File 1. Experiment description, QC data and ToxPI examples for iPSCs and HUVECs.

1.3.1. Total Cells

1.3.1.1. Phenotype definition

Total Cells is a count of all cells present in the imaged field. The fluorescent stain Hoechst 33342 was used to bind to DNA in the nucleus and enable the detection and count of all nuclei in the imaged area. Treatments that are cytotoxic were expected to result in a reduced total cell count compared to vehicle treatments.

1.3.1.2. Quality Control

1.3.1.2.1. Vehicle and Positive Controls

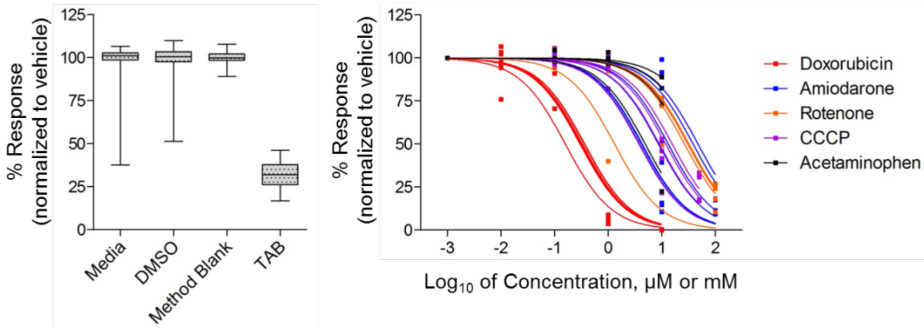

1.3.1.2.2. Intra- and Inter- Plate Replicability

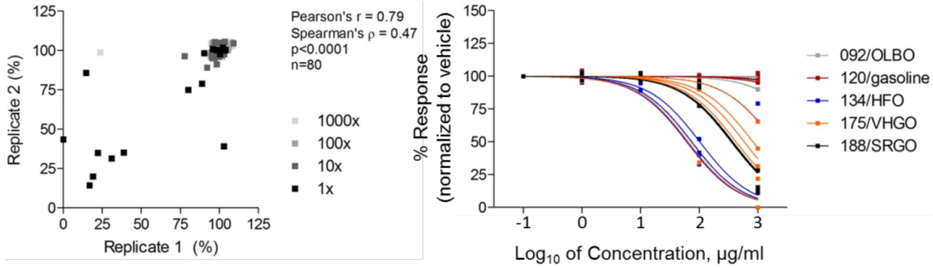

1.3.2. Nuclei Mean Intensity

1.3.2.1. Phenotype Definition

Nuclei Mean Intensity is a measure of the amount of light signal coming from stained nuclei. The fluorescent stain Hoechst 33342 was used to bind to DNA in the nuclei. The intensity of the fluorescent signal reflects the state of the chromosomes in the nucleus. Cells undergoing apoptosis (programmed cell death) exhibit nuclear condensation and more intense staining by Hoechst. An elevated Nuclei Mean Intensity suggests that the chemical treatment triggered apoptosis.

Supplemental File 1. Experiment description, QC data and ToxPI examples for iPSCs and HUVECs.

1.3.2.2. Quality Control

1.3.2.2.1. Vehicle and Positive Controls

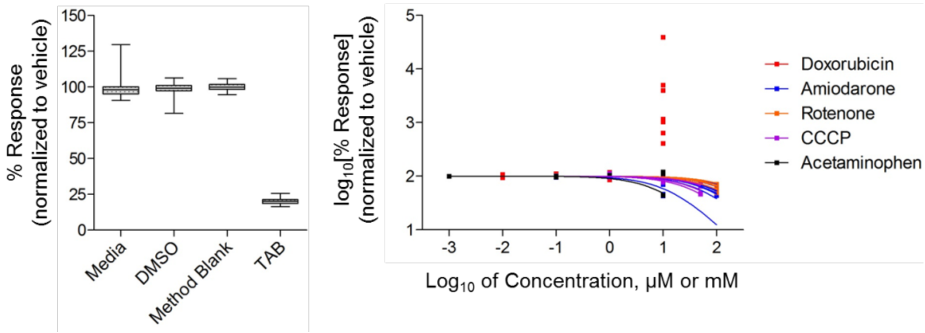

1.3.2.2.2. Intra- and Inter- Plate Replicability

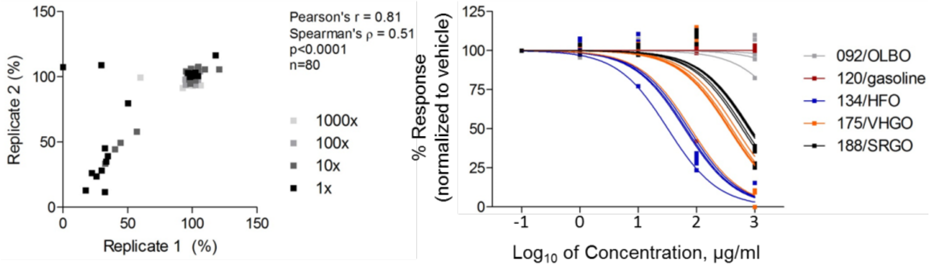

1.3.3. Positive Mitochondrial Staining

1.3.3.1. Phenotype Definition

Positive Mitochondrial Staining indicates the number of cells with intact mitochondria in an imaged field. Mitochondria are essential organelles that provide energy to drive cellular processes. Two key features of healthy, functional mitochondria are ATP synthesis and the generation of membrane potential. MitoTracker Orange was used to stain intact mitochondria. The MitoTracker dye is only fluorescent inside a live cell, and it is only sequestered within mitochondria possessing a membrane potential. Cytotoxic treatments or treatments that interfere with normal mitochondrial function were expected to have a lower Positive Mitochondrial Staining score than healthy cells.

1.3.3.2. Quality Control

1.3.3.2.1. Vehicle and Positive Controls

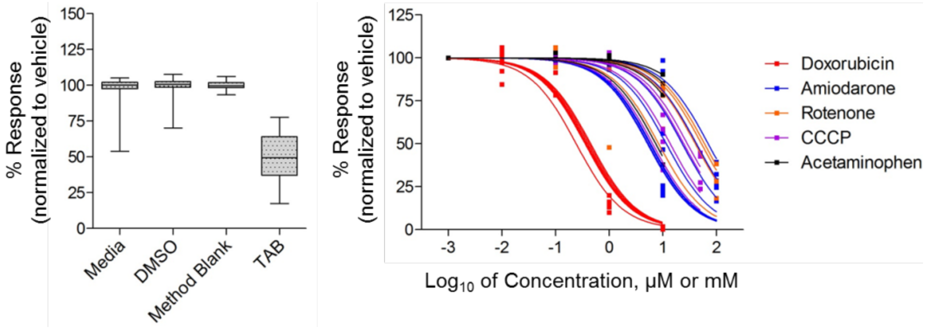

1.3.3.2.2. Intra- and Inter- Plate Replicability

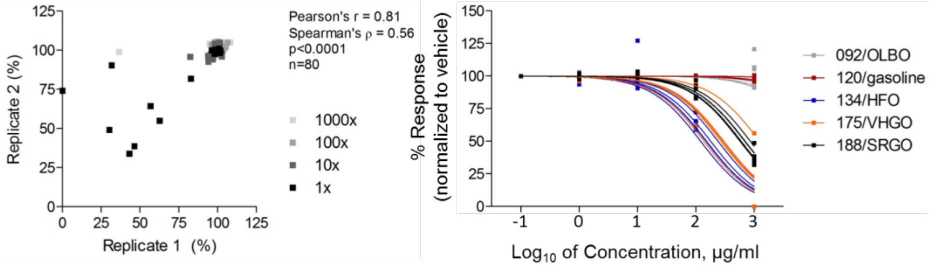

1.3.4. Mitochondrial Integrated Intensity

1.3.4.1. Phenotype Definition

Mitochondrial Integrated Intensity is an aggregate measure for the integrity of individual mitochondria. Due to the importance of the mitochondria to cellular health even slight changes in the integrity of mitochondria can alter the cellular function. Using the MitoTracker fluorescent stain, the degree of mitochondrial integrity can be assessed. Image processing computes an intensity across the image for determine the extent of intact mitochondria.

1.3.4.2. Quality Control

1.3.4.2.1. Vehicle and Positive Controls

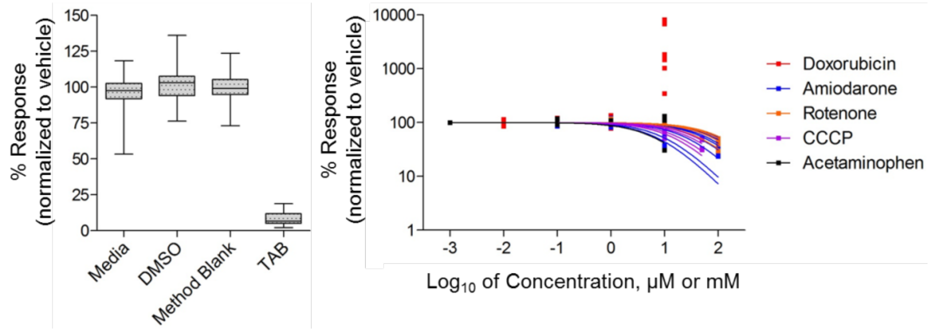

1.3.4.2.2. Intra- and Inter- Plate Replicability

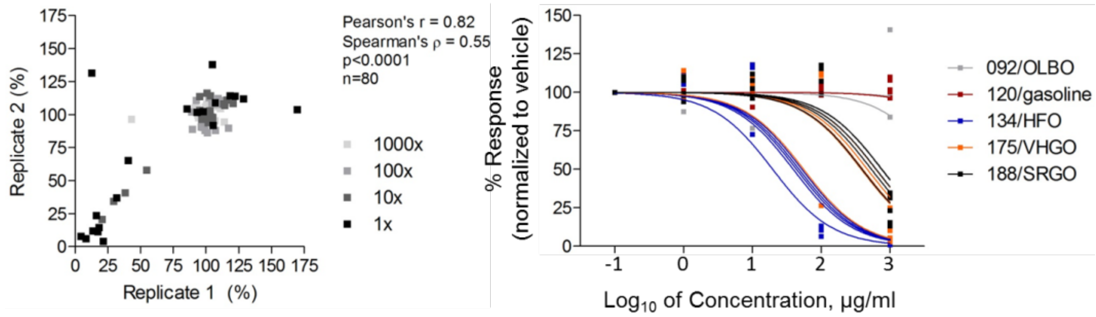

1.3.5. Positive Cytoplasmic Staining

1.3.5.1. Phenotypic Definition

Positive Cytoplasmic Staining indicates the number of cells in an imaged field that have an intact cytoplasm. The cytoplasm is the inner contents of the cell, excluding the nucleus. Calcein AM stain was used to visualize the cytoplasm and the general shape of each cell. This stain freely enters the cytoplasm, but it is only fluorescent and retained in the cytoplasm if active enzymes are present. Cytotoxic treatments that lyse (burst) cells are expected to result in a lower Positive Cytoplasmic Straining score compared to healthy cells with intact, functional cytoplasm.

1.3.5.2. Quality Control

1.3.5.2.1. Vehicle and Positive Controls

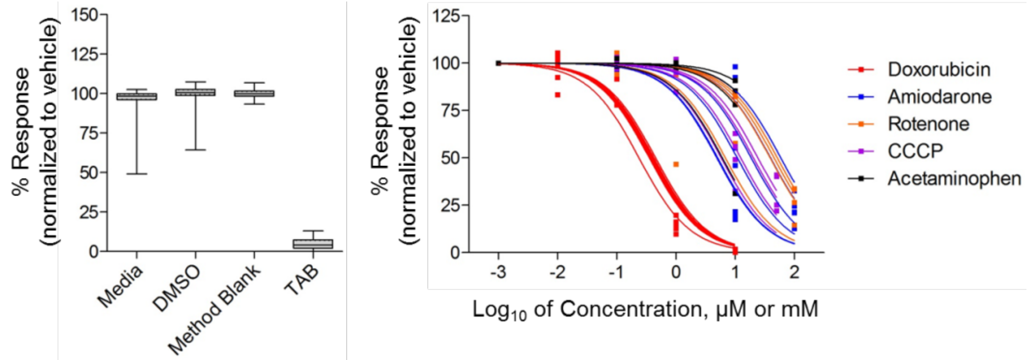

1.3.5.2.2. Intra- and Inter- Plate Replicability

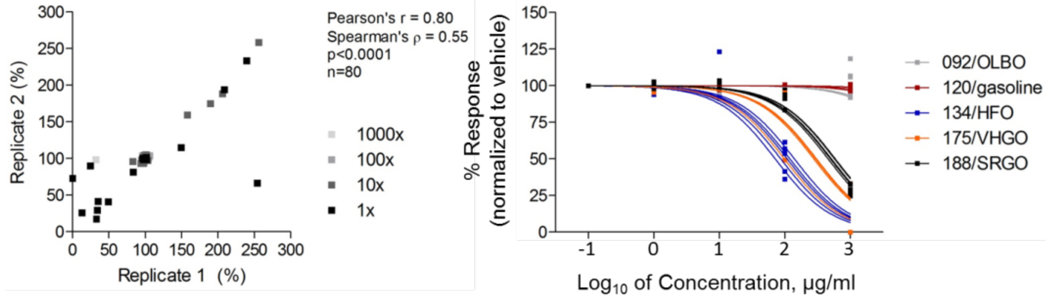

1.3.6. Cellular Mean Area

1.3.6.1. Phenotype Definition

Cellular Mean Area is the average area of all cells in an imaged field. The area of the nucleus (marked by Hoechst stain) plus the area of the cytoplasm (marked by Calcein AM) gives the area of a single cell. Then, the average cell area is calculated for the field. Cellular Mean Area is a general indicator of cell size and may be influenced by changes in cell morphology or health.

Supplemental File 1. Experiment description, QC data and ToxPI examples for iPSCs and HUVECs.

1.3.6.2. Quality Control

1.3.6.2.1. Vehicle and Positive Controls

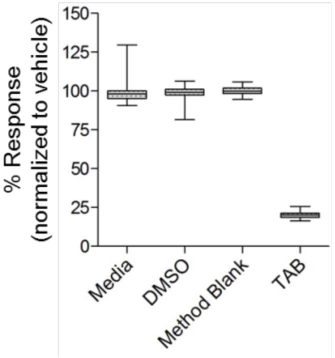

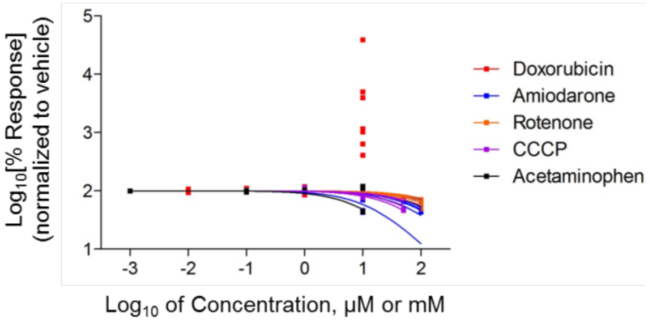

1.3.6.2.2. Intra- and Inter- Plate Replicability

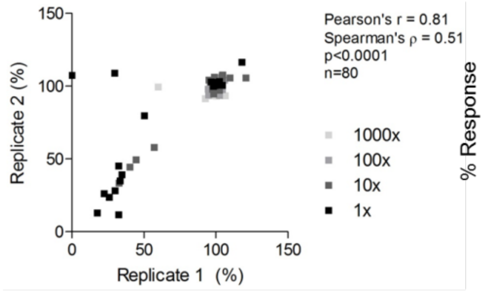

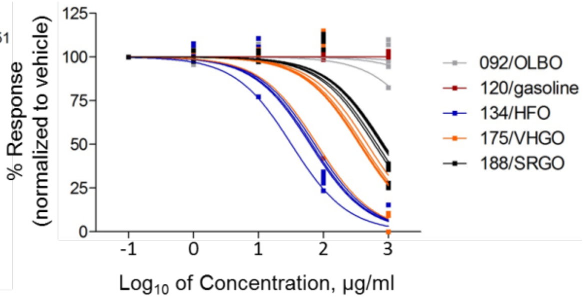

Supplemental File 1. Experiment description, QC data and ToxPI examples for iPSCs and HUVECs.

1.4. TOXPI AGGREGATION AND SUMMARY

1.4.1. ToxPi Key and ToxPi's for Select Petroleum Substances

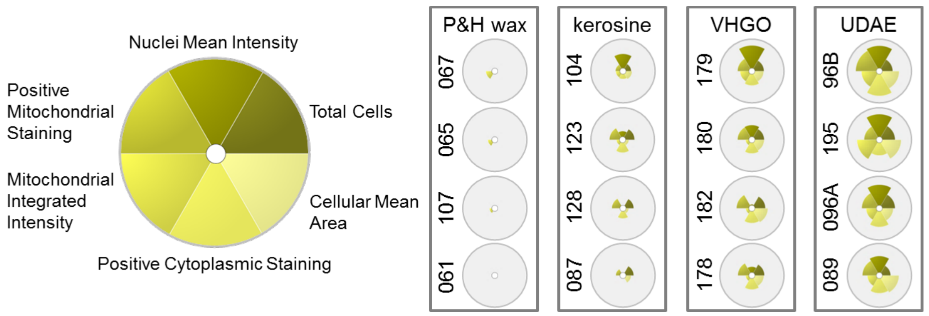

1.4.2. ToxPi Summary Graphs

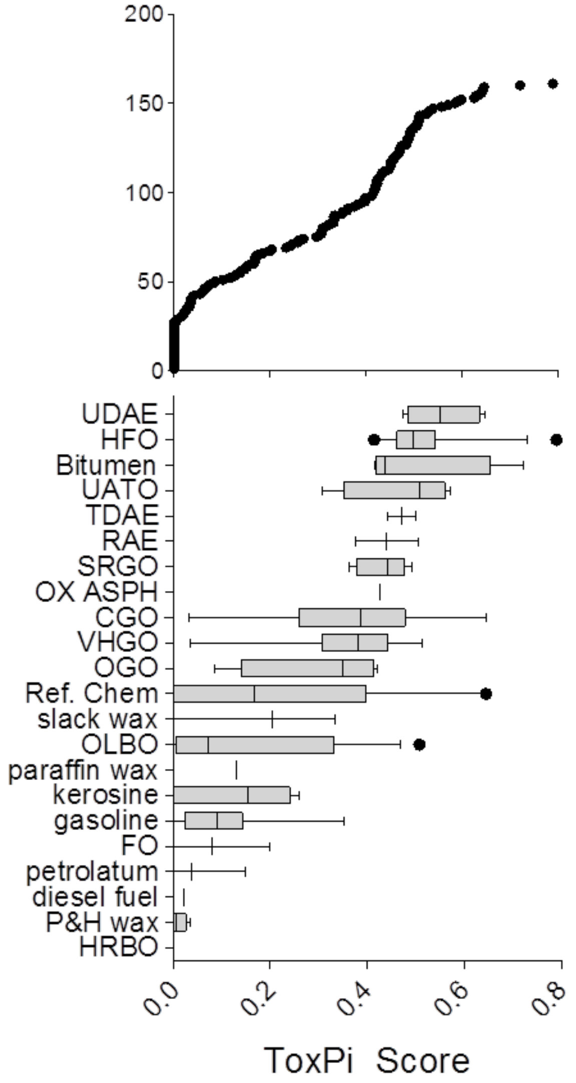

2. ENDOTHELIAL CELLS

Endothelial cells play an essential role in the vascular system by not only providing structure but contributing to overall vascular function through hemostasis and fluid filtration. Given the large number of cardiovascular disease cases associated with environmental exposures, 23% of all cases worldwide, an understanding of the degree of the endothelium-specific contribution is needed for various exposures.[10] Recent assay developments have resulted in high-throughput assays where important information regarding the bioactivity of chemicals on endothelial cells has been generated. These assays include measures of general bioactivity on endothelial cells, as well as the impact of test compounds on angiogenesis (growth of new vessels).[11]

2.1. iCELL ENDOTHELIAL CELLS

Within the catalogue of iPSC-derived cells offered by Cellular Dynamics, the iCell Endothelial Cells provide a model to assess vascular toxicity. Like primary endothelial cells, these iPSC-derived cells are responsive to cell signalling molecules such as VEGF and TNF-α. In addition, the product contains a pure population of endothelial cells to ensure consistency between screens.

2.1.1. Basal and Altered Morphology

Unlike other types of cells which form mosaic-like structures in traditional 2D culture, endothelial cells appear elongated with close associations with neighbouring cells similar to their morphology *in vivo*. Alterations caused by chemical treatment can disrupt normal cell morphology resulting in a punctate appearance of cells. This phenotype can be used as an endpoint for screening approaches. In addition, endothelial cells grown on semi-solid substrates, such as GelTrex®, can form tube-like, three-dimensional networks that model the angiogenesis process. These capillary-like networks can be modulated by chemical exposure, providing an additional set of endpoints to further probe the potential of vascular bioactivity posed by various chemicals.

Basal Morphology:[12]

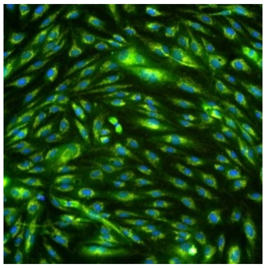

30 µM Colchicine:[18]

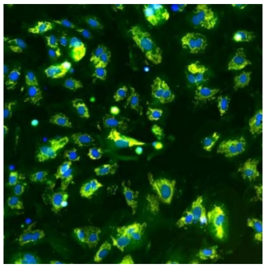

[10] Pruss-Ustun A, Corvalan C. "Preventing Disease Through Health Environments: Towards an Estimate of the Environmental Burden of Disease." *World Health Organization*, Geneva, Switzerland, 2006.
[11] Iwata Y, Klaren WD, Lebakken CS, Grimm FA, Rusyn I. "High-Content Assay Multiplexing for Vascular Toxicity Screening in Induced Pluripotent Stem Cell-Derived Endothelial Cells and Human Umbilical Vein Endothelial Cells." *Assay Drug Dev Technol.* 2017, 15(6).
[12] Nuclei are indicated in blue using Hoescht 33342 nuclear stain, cytoplasm is indicated in green using Calcein AM stain, and Mitochondria are indicated in yellow using Mitotracker fluorescent stain.

Supplemental File 1. Experiment description, QC data and ToxPI examples for iPSCs and HUVECs.

Basal Morphology on Geltrex®:[13] 100 nM Nocodazole:[19]

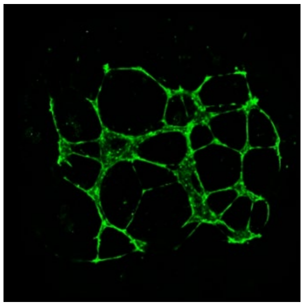

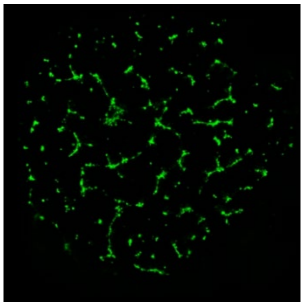

2.1.2. Endothelial Cell-Specific Positive Controls

Nocodazole

CasRN: 31430-18-9 Supplier: Cayman Chemical

Top Concentration: 1 µM (0.5% DMSO)

Nocodazole acts as an antimitotic agent by interfering with microtubule polymerization. This same action has been shown to disrupt the self-assembling of endothelial cells on extracellular matrices and was used here for this effet.[17]

Suramin

CasRN: 129-46-4 Supplier: Cayman Chemical

Top Concentration: 50 µM (0.5% DMSO)

Suramin was once believed to interfere directly with endothelial cells in their self-assembly into tube-like structures, but recent investigations suggest that suramin disrupts the extracellular matrix thus inhibiting the formation of tubes.[17,14] This compound still works well in predictably inhibiting the angiogenic assay which is why it is used here.

Histamine

CasRN: 51-45-6 Supplier: Alfa Aesar

Top Concentration: 100 µM (0.5% DMSO)

Histamine is an endogenous agent that acts on the endothelium resulting in vasodilation and the secretion of various cytokines. Concomitant treatment with other cytokines results in cytotoxicity to endothelial cells.[17] Here this compound was used as an agent that is known to cause bioactivity but does not cause cytotoxicity.

Colchicine

CasRN: 64-86-8 Supplier: Cayman Chemical

Top Concentration: 100 µM (0.5% DMSO)

Colchicine is a known therapeutic for gout-associated episodes. It works in part by altering the microtubule structure of the cell. In endothelial cells, it has similar microtubule effects as well as altering the surface proteins which

[13] Endothelial Cells grown on Geltrex® extracellular matrix and stained with Calcein AM.
[14] Prigozhina NL, Heisel AJ, Seldeen JR, Cosford ND, Price JH. “Amphiphilic suramin dissolves Matrigel, causing an ‘inhibition’ artefact within in vitro angiogenesis assay.” *Int J Exp Pathol.* 2013, 94.

causes alterations in neighbouring cell-cell interactions.[15] Here this compound was used for these effects on endothelial cells.

Chloroquine Phosphate
CasRN: 50-63-5 Supplier: Cayman Chemical
Top Concentration: 500 μM (0.5% DMSO)

Chloroquine Phosphate is used as an anti-malarial drug but also has effects on endothelial cells. Specifically, chloroquine inhibits endothelial cell proliferation by activating nitric oxide synthesis ultimately resulting the in the cells death.[16] The endothelial cytotoxicity was used here as a measure to ensure our assays were properly functioning.

Tetraoctylammonium Bromide (TAB)
CasRN: 14866-33-2 Supplier: Sigma
Top Concentration: 50 μM (0.5% DMSO)

Tetraoctylammonium bromide is a compound which consists of long aliphatic chains connected to an ammonium ion. TAB is commonly used in the surface coating of nanoparticles as the aliphatic chains impart specific characteristics onto the nanoparticle.[15] The long aliphatic chains have also been suggested to disrupt the cellular membrane which can serve as a reproducible and useful cytotoxic control. It was included here as a non-cellular specific cytotoxic control.

2.2. SCREEN SPECIFIC INFORMATION

Cell Manufacturer: Cellular Dynamics
Cell line Catalogue Number: R1022 (formerly ECC-100-010-001)
Cell Lot Number: 1833921
Date of Receipt of Cells at Texas A&M University: 6-20-17
Date Screening was performed: 6-22-17 to 7-5-17
Instrumentation used to record high-content imaging phenotypes:

- ImageXpress® Microconfocal High Content Imaging System (Molecular Devices, Sunnyvale, CA)
- FLIPR® Tetra High-Throughput Cellular Screening System (Molecular Devices, Sunnyvale, CA)

[15] Leung YY, Yao Hui LL, Kraus VB. "Colchicine-Update on mechanisms of action and therapeutic uses." *Sem Arthritis Rheum*, 2015, 45(3).

[16] Ghigo D, Aldieri E, Todde R, Costamagna C, Garbarino G, Pescarmona G, Bosia A. "Chloroquine stimulates nitric oxide synthesis in murine, porcine, and human endothelial cells." *J Clin Invest*, 1998, 102(3).

2.3. ENDOTHELIAL CELL ENDPOINTS FOR BIOACTIVITY PROFILING

2.3.1. Total Cells

2.3.1.1. Phenotype definition

Total Cells is a count of all cells present in the imaged field. The fluorescent stain Hoechst 33342 was used to bind to DNA in the nucleus and enable the detection and count of all nuclei in the imaged area. Since each cell contains a single nucleus, the number of nuclei reflects the total number of cells present. Treatments that are cytotoxic were expected to result in a reduced total cell count compared to media/vehicle treatments.

2.3.1.2. Quality Control

2.3.1.2.1. Vehicle and Positive Controls

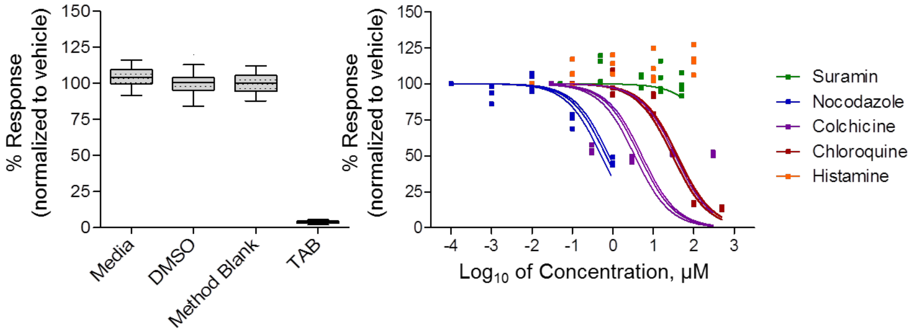

2.3.1.2.2. Intra- and Inter- Plate Replicability

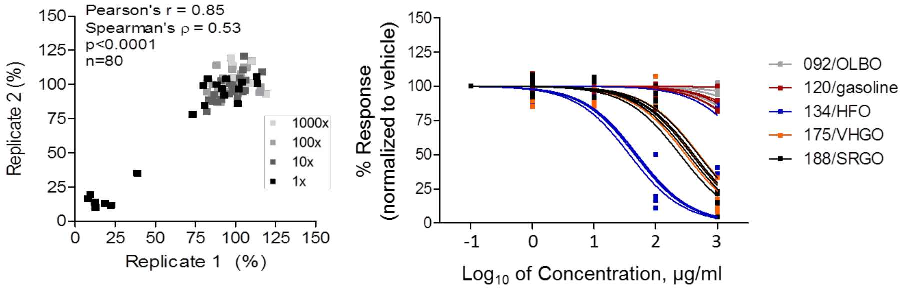

2.3.2. Nuclei Mean Intensity

2.3.2.1. Phenotype Definition

Nuclei Mean Intensity is a measure of the amount of light signal coming from stained nuclei. The fluorescent stain Hoechst 33342 was used to bind to DNA in the nucleus. The intensity of the fluorescent signal reflects the state of the chromosomes in the nucleus. Cells undergoing apoptosis (programmed cell death) exhibit nuclear condensation and more intense staining by Hoechst. An increased Nuclei Mean Intensity suggests that the chemical treatment triggered apoptosis.

Supplemental File 1. Experiment description, QC data and ToxPI examples for iPSCs and HUVECs.

2.3.2.2. Quality Control

2.3.2.2.1. Vehicle and Positive Controls

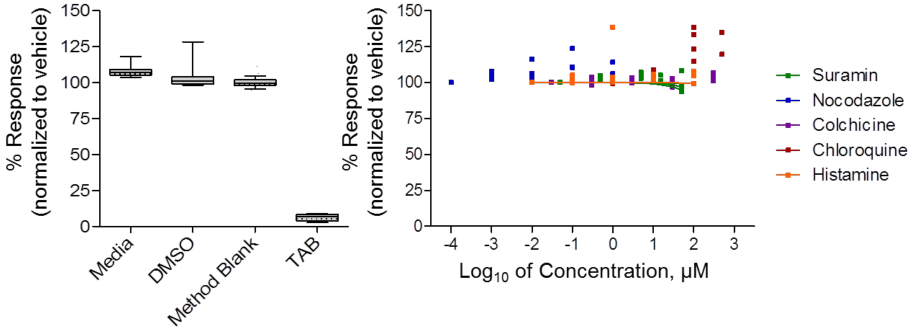

2.3.2.2.2. Intra- and Inter- Plate Replicability

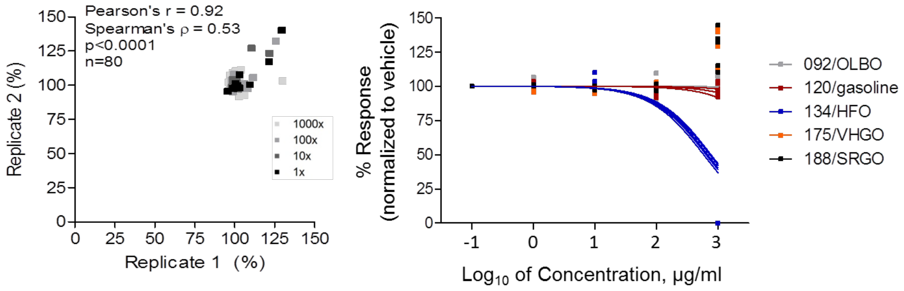

2.3.3. Positive Mitochondrial Staining

2.3.3.1. Phenotype Definition

Positive mitochondrial staining indicates the number of cells in an imaged field with intact mitochondria. Mitochondria are essential organelles that provide energy to drive cellular processes. Two key features of healthy, functional mitochondria are ATP synthesis and the generation of membrane potential. MitoTracker Orange was used to stain intact mitochondria. The MitoTracker dye is only fluorescent inside a live cell, and it is only sequestered within healthy mitochondria possessing a membrane potential. Cytotoxic treatments or treatments that interfere with normal mitochondrial function were expected to have a lower Positive Mitochondrial Staining score than healthy cells.

Supplemental File 1. Experiment description, QC data and ToxPI examples for iPSCs and HUVECs.

2.3.3.2. Quality Control

2.3.3.2.1. Vehicle and Positive Controls

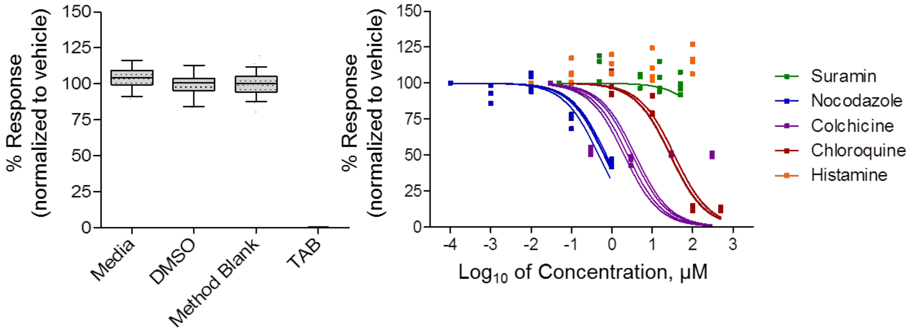

2.3.3.2.2. Intra- and Inter- Plate Replicability

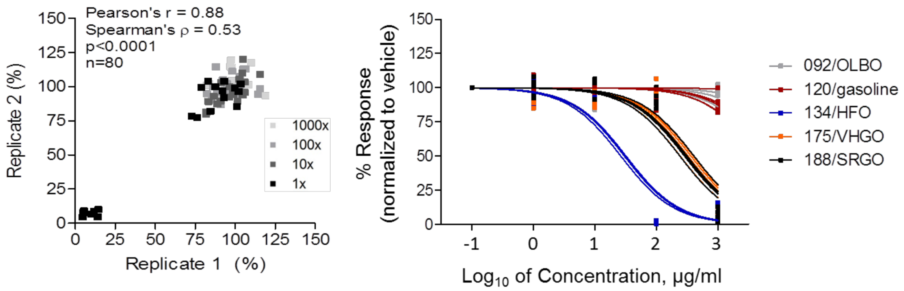

2.3.4. Positive Cytoplasmic Staining

2.3.4.1. Phenotypic Definition

Positive Cytoplasmic Staining indicates the number of cells in an imaged field that have an intact cytoplasm. The cytoplasm is the inner contents of the cell, excluding the nucleus. Calcein AM stain was used to visualize the cytoplasm and the general shape of each cell. This stain freely enters the cytoplasm, but it is only fluorescent and retained in the cytoplasm when active enzymes are present. Cytotoxic treatments that lyse (burst) cells are expected to result in a lower Positive Cytoplasmic Straining score compared to healthy cells with intact, functional cytoplasm.

2.3.4.2. Quality Control

2.3.4.2.1. Vehicle and Positive Controls

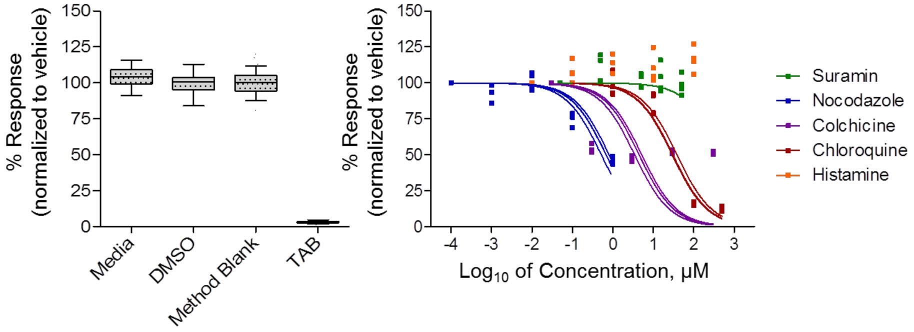

Supplemental File 1. Experiment description, QC data and ToxPI examples for iPSCs and HUVECs.

2.3.4.2.2. Intra- and Inter- Plate Replicability

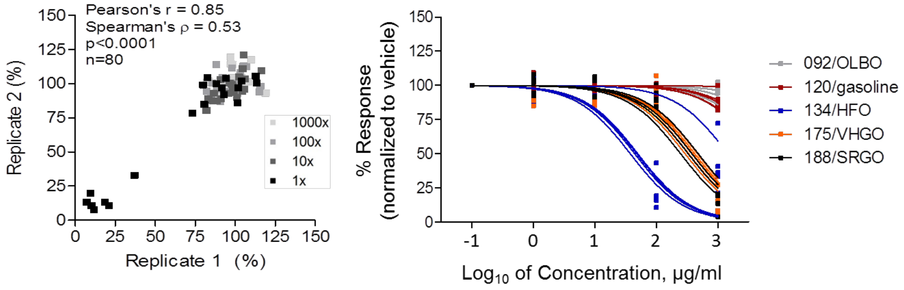

2.3.5. ATP Content

2.3.5.1. Phenotype Definition

ATP Content is the total amount of ATP present in all cells of a given treatment group. ATP is the energy currency of a cell and an indicator of cell metabolic activity. Since only live cells are metabolically active, ATP Content is an indicator of the total number of live cells. ATP content of cells was measured indirectly using the CellTiter-Glo® assay kit and the FLIPR® Tetra Screening System for detection. The CellTiter-Glo® reagent produces a luminescent signal that is proportional to the total amount of ATP present in a treatment group. Cells treated with cytotoxic chemicals were expected to produce little/no ATP signal.

2.3.5.2. Quality Control

2.3.5.2.1. Vehicle and Positive Controls

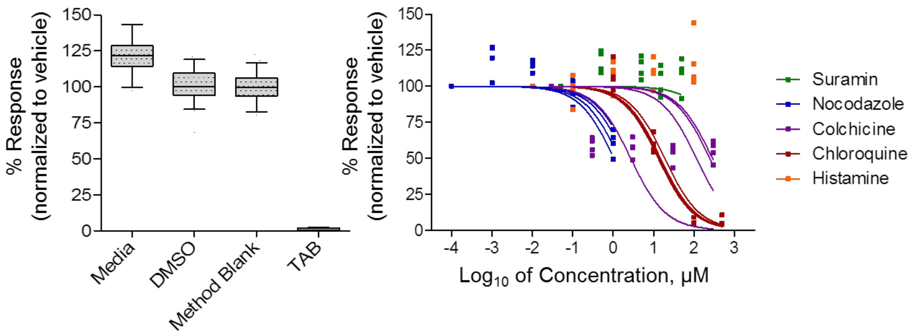

2.3.5.2.2. Intra- and Inter- Plate Replicability

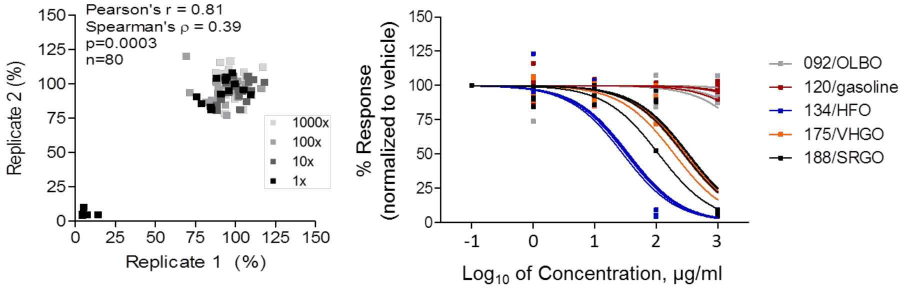

2.3.6. Total Tube Length

2.3.6.1. Phenotype Definition

Endothelial cells cultured on an extracellular matrix, i.e. Geltrex®, form a 3D-network that models the angiogenesis (new vessel formation) process. This unique feature of endothelial cells depends on intracellular signalling cascades, enzyme production and formation of cell-cell contacts. The Tube Formation Assay was conducted in a GelTrex® matrix to monitor the effects of chemical treatments on angiogenesis. Here, cells were stained with Calcien AM so that cytoplasmic extensions (tubes) and their networks could be visualized. Total Tube Length is one measureable feature of angiogenesis that takes into account both the number to tube-like structures and their length. Disruptors of angiogenesis are expected to result in fewer and/or shorter tubes.

2.3.6.2. Quality Control

2.3.6.2.1. Vehicle and Positive Controls

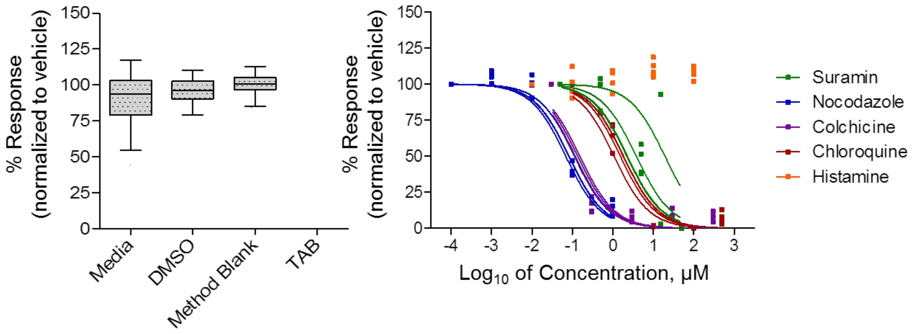

2.3.6.2.2. Intra- and Inter- Plate Replicability

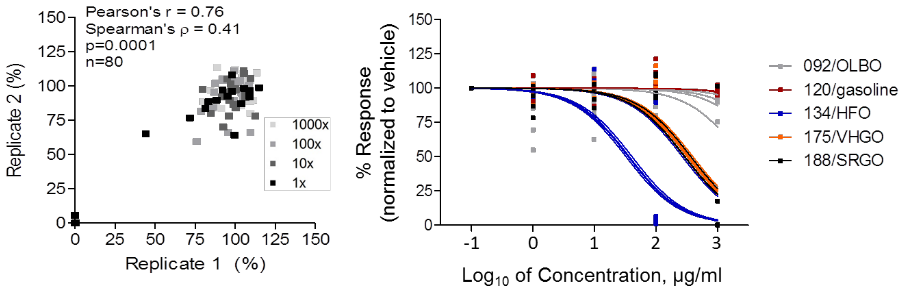

2.3.7. Mean Tube Length

2.3.7.1. Phenotype Definition

Another aspect of angiogenesis that can be measured by the Tube Formation Assay is Mean Tube Length. This is the average length of the tube-like extensions of all cells in the imaged field, and it is independent of the number of extensions present. Disruptors of angiogenesis can work via different mechanisms which may be distinguished by examining different aspects of tube formation.

Supplemental File 1. Experiment description, QC data and ToxPI examples for iPSCs and HUVECs.

2.3.7.2. Quality Control

2.3.7.2.1. Vehicle and Positive Controls

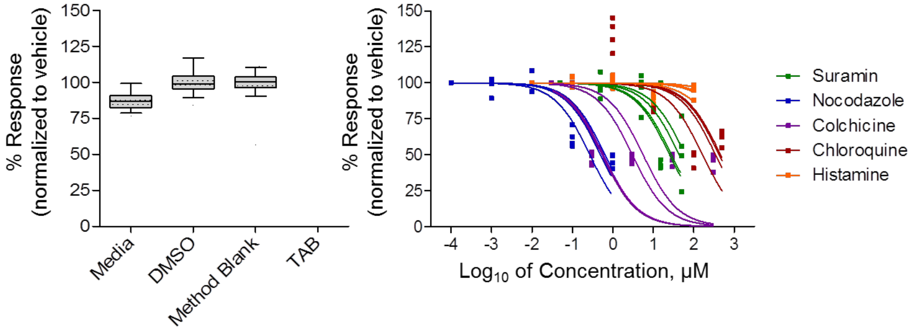

2.3.7.2.2. Intra- and Inter- Plate Replicability

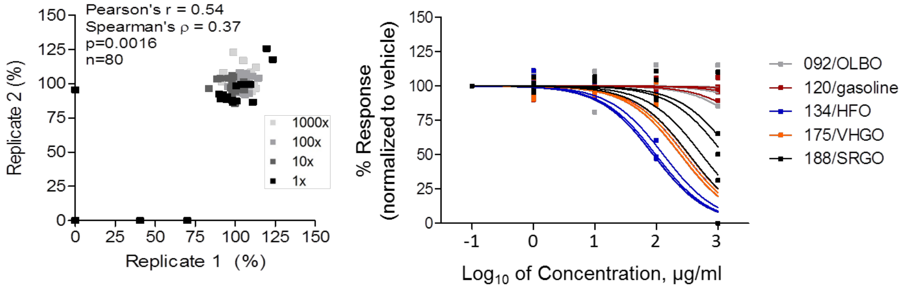

2.3.8. Total Tube Area

2.3.8.1. Phenotype Definition

Total Tube Area provides another means for assessing angiogenesis via the Tube Formation Assay. As the name indicates, this phenotype describes both the length and the width of the tubes formed. The area of all tubes in the imaged field is summed to provide a value that reflects the bulk amount of tube-like structures present. Again, by examining multiple phenotypes of tube formation, bioactive chemicals with diverse mechanisms of action can potentially be detected.

2.3.8.2. Quality Control

2.3.8.2.1. Vehicle and Positive Controls

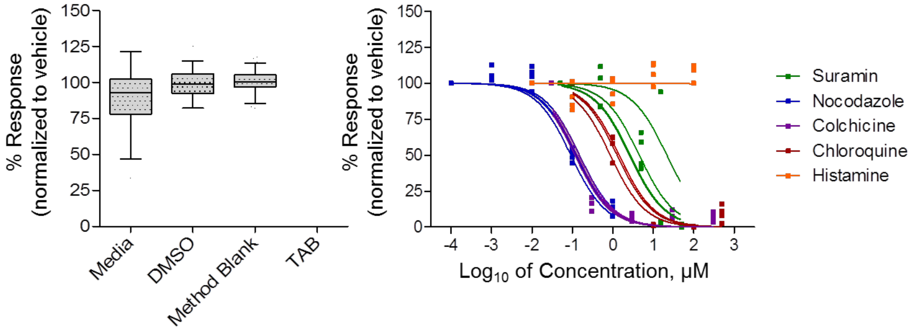

2.3.8.2.2. Intra- and Inter- Plate Replicability

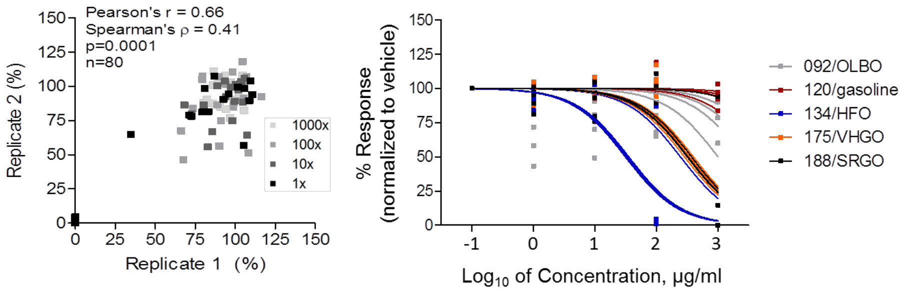

2.3.9. Branch Points

Branch Points provides additional readout for assessing angiogenesis via the Tube Formation Assay. The degree of connections or branches that occur along the tubes can provide an idea of the level of complexity within the network. Agents that disrupt tube branching may result in a less complex network and would have fewer branch points along the tubes.

2.3.9.1. Phenotype Definition

2.3.9.2. Quality Control

2.3.9.2.1. Vehicle and Positive Controls

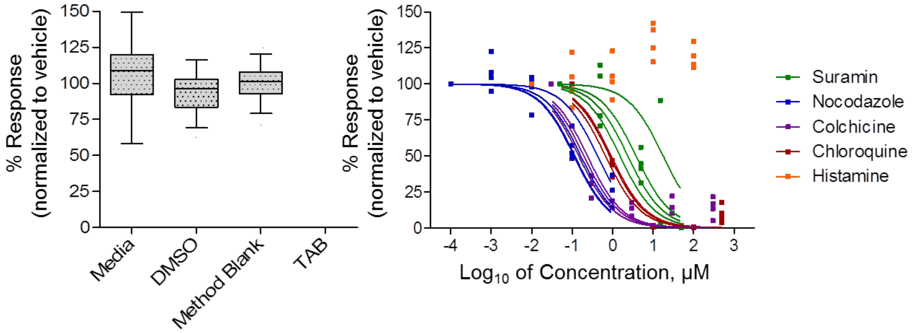

2.3.9.2.2. Intra- and Inter- Plate Replicability

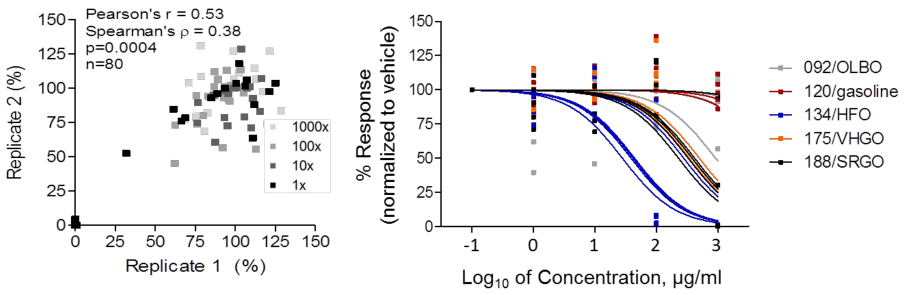

Supplemental File 1. Experiment description, QC data and ToxPI examples for iPSCs and HUVECs.

2.4. TOXPI AGGREGATION AND SUMMARY

2.4.1. ToxPi Key and ToxPi's for Select Petroleum Substances

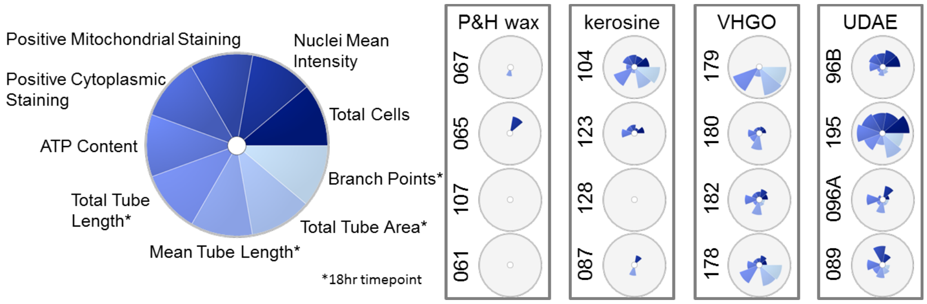

2.4.2. ToxPi Summary Graphs

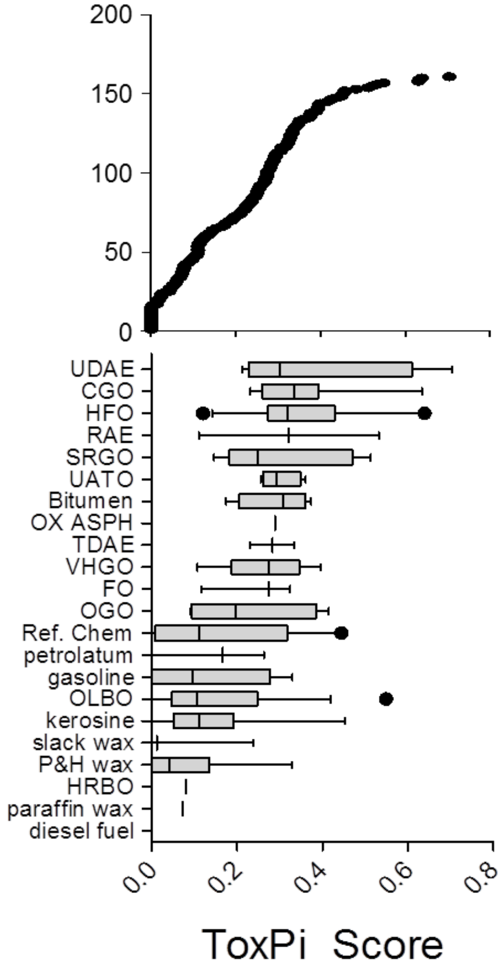

3. HUMAN UMBILICAL VEIN ENDOTHELIAL CELLS

The importance and function of endothelial cells are discussed above but the source of endothelial cells can vary greatly. Whereas induced pluripotent stem cell derived-endothelial cells were used in the last section, an additional primary human endothelial cell type was also used.

3.1. HUMAN UMBILICAL VEIN ENDOTHELIAL CELLS (HUVECS)

Human umbilical vein endothelial cells (HUVECs) are harvested from the resected umbilical cord just after birth and can be obtained easily from several different vendors. This provides a low cost primary human source of cells for scientific research. An unfortunate drawback of HUVECs is the variable nature of the cells as they are isolated from different individuals with varying genetic backgrounds. This is somewhat overcome by using pooled samples where multiple donor cells are combined together resulting in a heterogeneous group of umbilical endothelial cells. HUVECs were used in conjunction with iPSC-derived endothelial cells here to increase the confidence with the endothelial cell type. Previously, it has been shown that although HUVECs and iPSC-derived endothelial cells are of the same type, their response to chemical treatments can vary.[17] Therefore, both cell types were run in parallel to assess vascular bioactivity.

3.1.1. Basal and Altered Morphology

HUVECs in culture display the same characteristic cellular morphology and arrangement as iCell Enodthelial cells. The normally elongated HUVEC bodies can be altered by chemicals that interfere with intracellular structure, making morphology a convenient endpoint for detecting bioactivity of a test chemical.

Basal Morphology:[17]

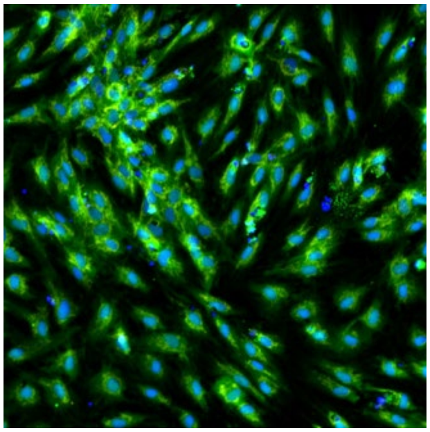

30 µM Colchicine:[23]

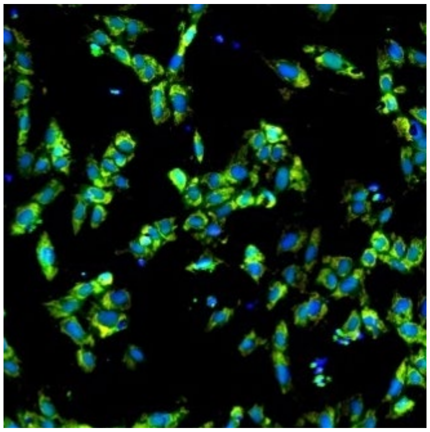

[17] Nuclei are indicated in blue using Hoescht 33342 nuclear stain, cytoplasm is indicated in green using Calcein AM stain, and Mitochondria are indicated in yellow using Mitotracker fluorescent stain.

[24] HUVECs grown on GelTrex® and stained with Calcein AM.

Basal Morphology:[18]

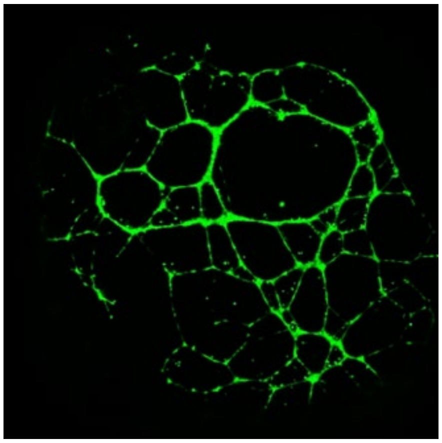

100 nM Nocodazole:[24]

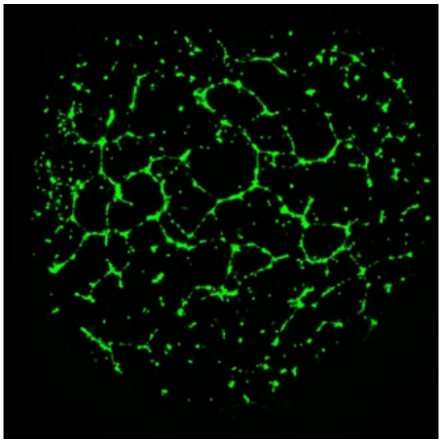

3.1.2. HUVEC-Specific Positive Controls

Nocodazole

CasRN: 31430-18-9 Supplier: Cayman Chemical

Top Concentration: 1 µM (0.5% DMSO)

Nocodazole acts as an antimitotic agent by interfering with microtubule polymerization. This same action has been shown to disrupt the self-assembling of endothelial cells on extracellular matrices and was used here for this effect.[17]

Suramin

CasRN: 129-46-4 Supplier: Cayman Chemical

Top Concentration: 50 µM (0.5% DMSO)

Suramin was once believed to interfere directly with endothelial cells in their self-assembly into tube-like structures, but recent investigations suggest that suramin disrupts the extracellular matrix thus inhibiting the formation of tubes.[17,20] This compound still works well in predictably inhibiting the angiogenic assay which is why it is used here.

Histamine

CasRN: 51-45-6 Supplier: Alfa Aesar

Top Concentration: 100 µM (0.5% DMSO)

Histamine is an endogenous agent that acts on the endothelium resulting in vasodilation and the secretion of various cytokines. Concomitant treatment with other cytokines results in cytotoxicity to endothelial cells.[17] Here this compound was used as an agent that is known to cause bioactivity but does not cause cytotoxicity.

Colchicine

CasRN: 64-86-8 Supplier: Cayman Chemical

Top Concentration: 100 µM (0.5% DMSO)

Colchicine is a known therapeutic for gout-associated episodes. It works in part by altering the microtubule structure of the cell. In endothelial cells, it has similar microtubule effects as well as altering the surface proteins which causes alterations in neighbouring cell-cell interactions.[21] Here this compound was used for these effects on endothelial cells.

Chloroquine Phosphate

CasRN: 50-63-5 Supplier: Cayman Chemical

Top Concentration: 500 µM (0.5% DMSO)

Chloroquine Phosphate is used as an anti-malarial drug but also has effects on endothelial cells. Specifically, chloroquine inhibits endothelial cell proliferation by activating nitric oxide synthesis ultimately resulting the in the cells death.[22] The endothelial cytotoxicity was used here as a measure to ensure our assays were properly functioning.

SU5402

CasRN: 215543-92-3 Supplier: Sigma

Top Concentration: 50 µM (0.5% DMSO)

SU5402 is a known inhibitor of the VEGF receptor. The VEGF receptor plays a crucial role in angiogenesis by directly endothelial cells to grow or aggregate together. Previous reports have shown its ability to disrupt angiogenic ability in *in vitro* screens warranting its inclusion here.[17]

Tetraoctylammonium Bromide (TAB)

CasRN: 14866-33-2 Supplier: Sigma

Top Concentration: 50 µM (0.5% DMSO)

Tetraoctylammonium bromide is a compound which consists of long aliphatic chains connected to an ammonium ion. TAB is commonly used in the surface coating of nanoparticles as the aliphatic chains impart specific characteristics onto the nanoparticle.[15] The long aliphatic chains have also been suggested to disrupt the cellular membrane which can serve as a reproducible and useful cytotoxic control. It was included here as a non-cellular specific cytotoxic control.

3.2. SCREEN SPECIFIC INFORMATION

Cell Manufacturer: Lonza

Cell line Catalogue Number: C2519A

Cell Lot Number: 0000433795

Date of Receipt of Cells at Texas A&M University: 12-07-16

Date Screening was performed: 12-7-16 to 12-21-17

Instrumentation used to record high-content imaging phenotypes:

- ImageXpress® Microconfocal High Content Imaging System (Molecular Devices, Sunnyvale, CA)

Supplemental File 1. Experiment description, QC data and ToxPI examples for iPSCs and HUVECs.

3.3. HUVEC ENDPOINTS FOR BIOACTIVITY PROFILING

3.3.1. Total Cells

3.3.1.1. Phenotype definition

Total Cells is a count of all cells present in the imaged field. The fluorescent stain Hoechst 33342 was used to bind to DNA in the nucleus and enable the detection and count of all nuclei in the imaged area. Since each cell contains a single nucleus, the number of nuclei reflects the total number of cells present. Treatments that are cytotoxic were expected to result in a reduced total cell count compared to media/vehicle treatments.

3.3.1.2. Quality Control

3.3.1.2.1. Vehicle and Positive Controls

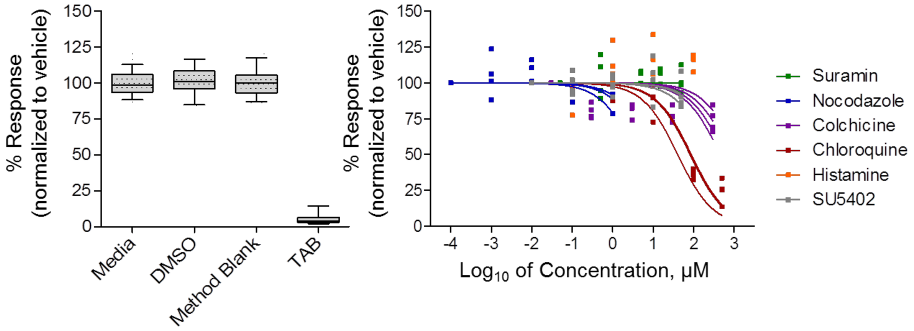

3.3.1.2.2. Intra- and Inter- Plate Replicability

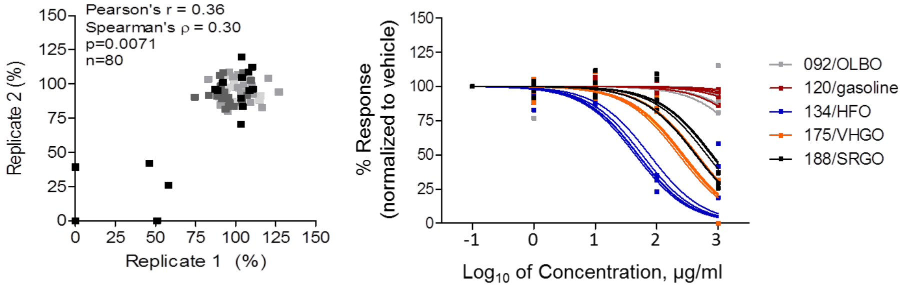

3.3.2. Nuclei Mean Intensity

3.3.2.1. Phenotype Definition

Nuclei Mean Intensity is a measure of the amount of light signal coming from stained nuclei. The fluorescent stain Hoechst 33342 was used to bind to DNA in the nucleus. The intensity of the fluorescent signal reflects the state of the chromosomes in the nucleus. Cells undergoing apoptosis (programmed cell death) exhibit nuclear condensation and more intense staining by Hoechst. An increased Nuclei Mean Intensity suggests that the chemical treatment triggered apoptosis.

Supplemental File 1. Experiment description, QC data and ToxPI examples for iPSCs and HUVECs.

3.3.2.2. Quality Control

3.3.2.2.1. Vehicle and Positive Controls

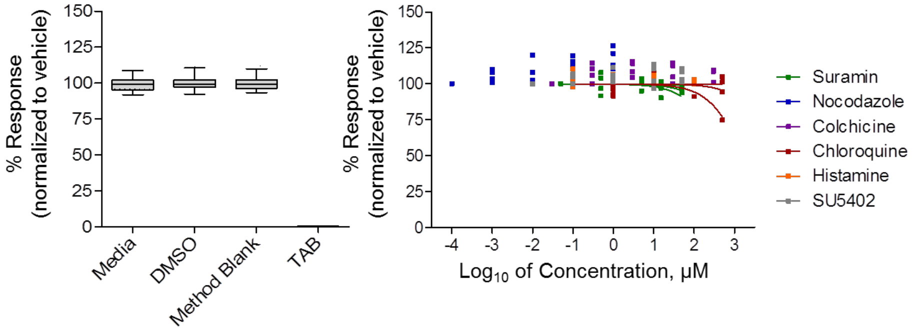

3.3.2.2.2. Intra- and Inter- Plate Replicability

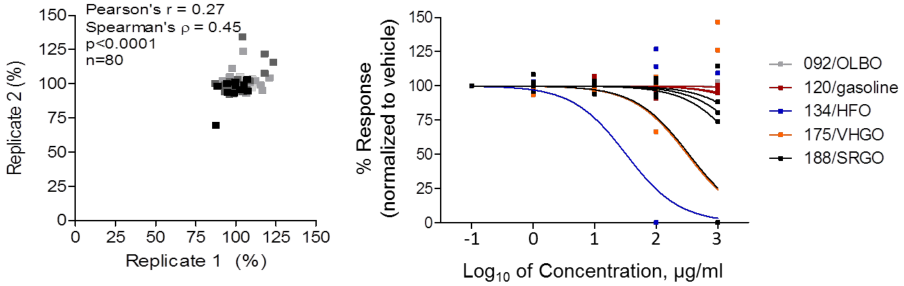

3.3.3. Positive Mitochondrial Staining

3.3.3.1. Phenotype Definition

Positive mitochondrial staining indicates the number of cells in an imaged field with intact mitochondria. Mitochondria are essential organelles that provide energy to drive cellular processes. Two key features of healthy, functional mitochondria are ATP synthesis and the generation of membrane potential. MitoTracker Orange was used to stain intact mitochondria. The MitoTracker dye is only fluorescent inside a live cell, and it is only sequestered within healthy mitochondria possessing a membrane potential. Cytotoxic treatments or treatments that interfere with normal mitochondrial function were expected to have a lower Positive Mitochondrial Staining score than healthy cells.

Supplemental File 1. Experiment description, QC data and ToxPI examples for iPSCs and HUVECs.

3.3.3.2. Quality Control

3.3.3.2.1. Vehicle and Positive Controls

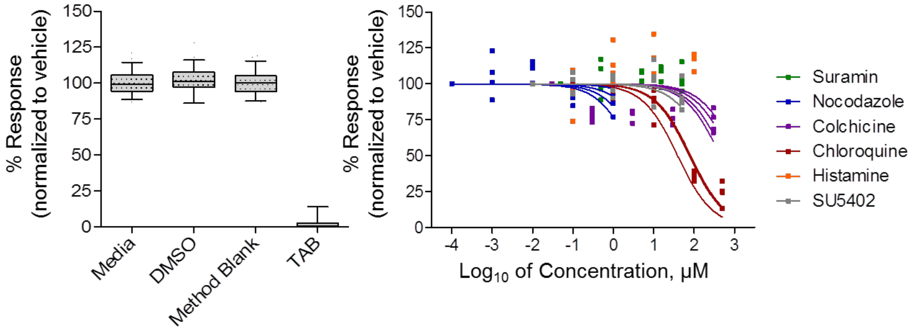

3.3.3.2.2. Intra- and Inter- Plate Replicability

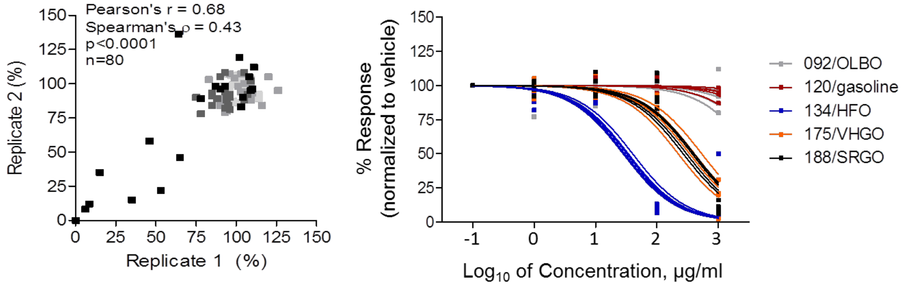

3.3.4. Positive Cytoplasmic Staining

3.3.4.1. Phenotypic Definition

Positive Cytoplasmic Staining indicates the number of cells in an imaged field that have an intact cytoplasm. The cytoplasm is the inner contents of the cell, excluding the nucleus. Calcein AM stain was used to visualize the cytoplasm and the general shape of each cell. This stain freely enters the cytoplasm, but it is only fluorescent and retained in the cytoplasm when active enzymes are present. Cytotoxic treatments that lyse (burst) cells are expected to result in a lower Positive Cytoplasmic Straining score compared to healthy cells with intact, functional cytoplasm.

3.3.4.2. Quality Control

3.3.4.2.1. Vehicle and Positive Controls

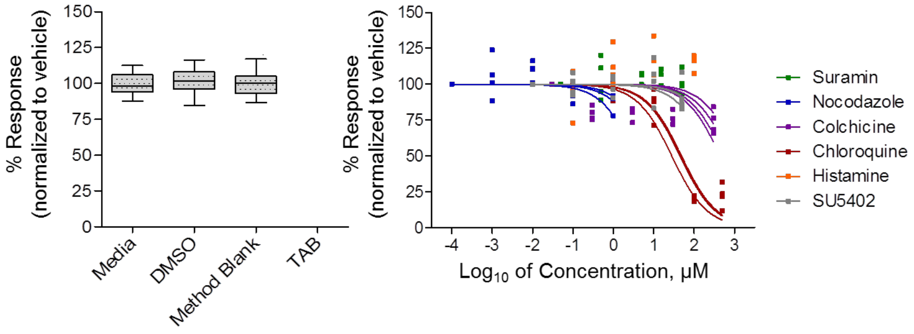

Supplemental File 1. Experiment description, QC data and ToxPI examples for iPSCs and HUVECs.

3.3.4.2.2. Intra- and Inter- Plate Replicability

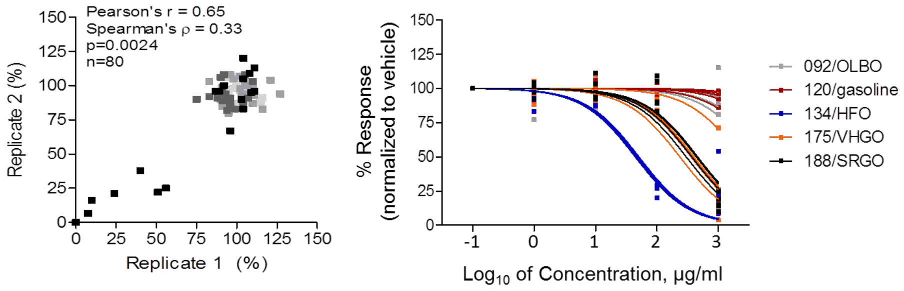

3.3.5. Total Tube Length

3.3.5.1. Phenotype Definition

Endothelial cells cultured on an extracellular matrix, i.e. Geltrex®, form a 3D-network that models the angiogenesis (new vessel formation) process. This unique feature of endothelial cells depends on intracellular signalling cascades, enzyme production and formation of cell-cell contacts. The Tube Formation Assay was conducted in a GelTrex® matrix to monitor the effects of chemical treatments on angiogenesis. Here, cells were stained with Calcein AM so that cytoplasmic extensions (tubes) and their networks can be visualized. Total Tube Length is one measureable feature of angiogenesis that takes into account both the number to tube-like structures and their length. Disruptors of angiogenesis are expected to result in fewer and/or shorter tubes.

3.3.5.2. Quality Control

3.3.5.2.1. Vehicle and Positive Controls

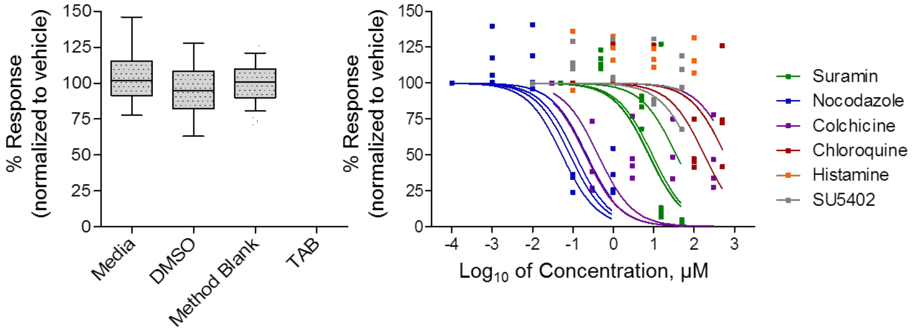

Supplemental File 1. Experiment description, QC data and ToxPI examples for iPSCs and HUVECs.

3.3.5.2.2. Intra- and Inter- Plate Replicability

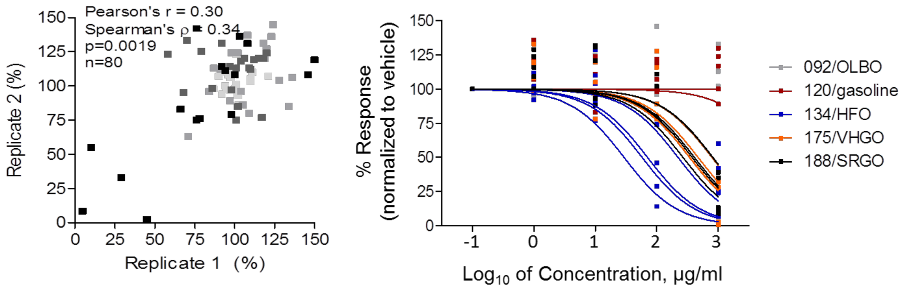

3.3.6. Mean Tube Length

3.3.6.1. Phenotype Definition

Another aspect of angiogenesis that can be measured by the Tube Formation Assay is Mean Tube Length. This is the average length of the tube-like extensions of all cells in the imaged field, and it is independent of the number of extensions present. Disruptors of angiogenesis can work via different mechanisms which may be distinguished by examining multiple parameters of tube formation.

3.3.6.2. Quality Control

3.3.6.2.1. Vehicle and Positive Controls

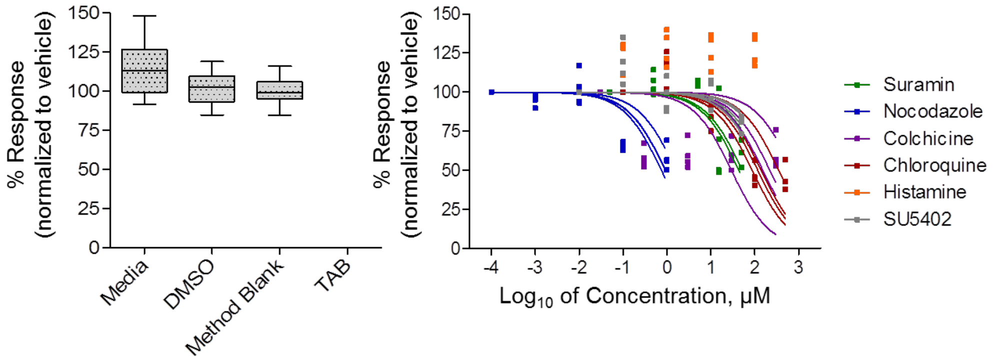

3.3.6.2.2. Intra- and Inter- Plate Replicability

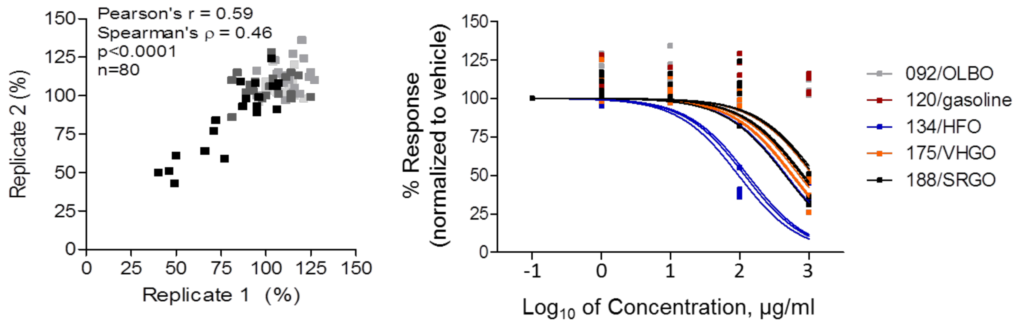

Supplemental File 1. Experiment description, QC data and ToxPI examples for iPSCs and HUVECs.

3.4. TOXPI AGGREGATION AND SUMMARY

3.4.1. ToxPi Key and ToxPi's for Select Petroleum Substances

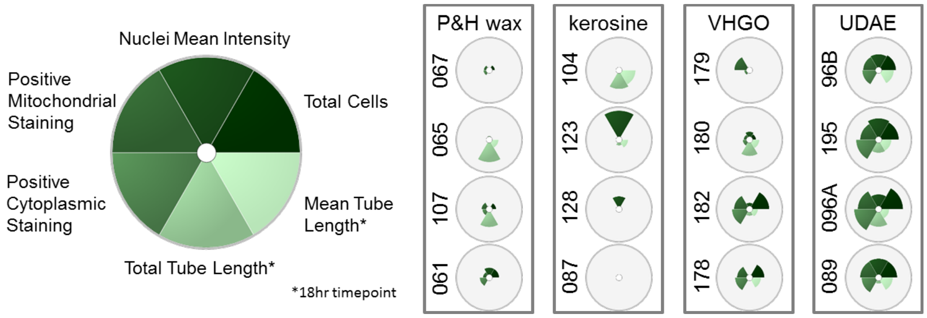

3.4.2. ToxPi Summary Graphs

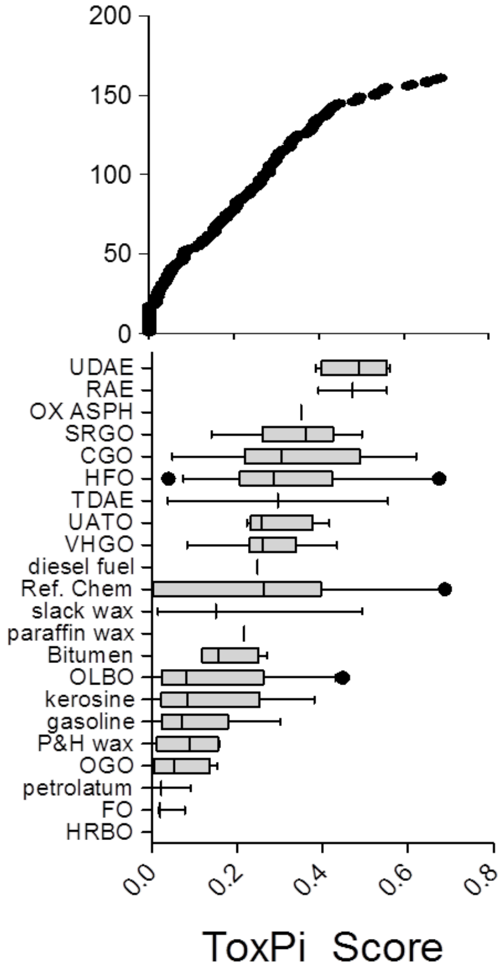

4. CARDIOMYOCYTES

The major organ of the cardiovascular system is the heart. The heart contains various cells types, but largely consists of specialized muscle cells called cardiomyocytes. The continuous contraction of cardiomyocytes allows the heart to beat, providing blood circulation through the human body. Due to the near continuous function, the cardiomyocytes can be susceptible to altered effect by a range of different environmental exposures. *In vitro* models of cardiomyocytes provide similar functionality has cardiomyocytes *in vivo* which can be used in screening approaches to gauge a chemical's bioactivity potential.

4.1. iCELL CARDIOMYOCYTES

A widely used product from Cellular Dynamics is the iCell Cardiomyocytes. Although not unique compared to other two dimensional cardiomyocyte models, these iPSC-derived cells reliably beat in a synchronous fashion. This beating elicits a functional and very sensitive endpoint for the investigation of biological activity of various chemicals. In addition, this product represents a pure population of cardiac cells from various parts of the heart which may better represent the organ as a whole.

4.1.1. Basal and Altered Morphology

Similar to other cells grown in two-dimensional culture, cardiomyocytes develop a confluent monolayer. Their morphology is more elongated than other cells in culture due to the presence of T-tubules which provide the contraction of the muscle cell. Treatment of chemical or physical damage caused by shear force while maintaining the cells can results in destruction of the monolayer and greatly diminishes the functionality of the cell.

Basal Morphology:[19]

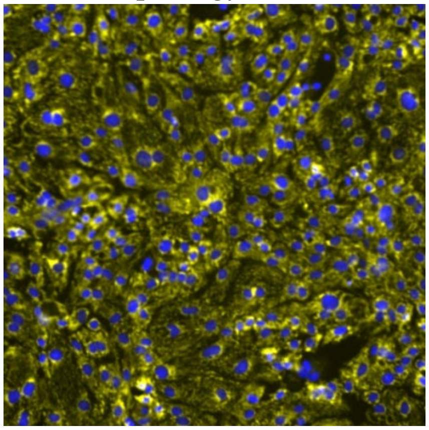

Altered Morphology (Physical damage):[25]

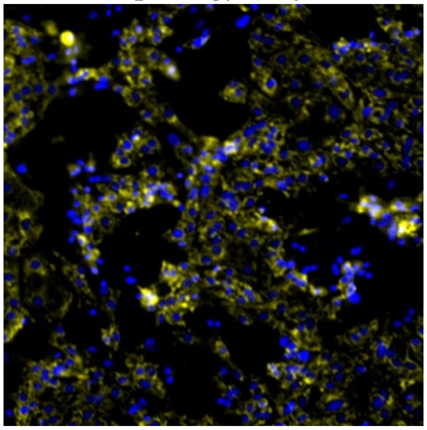

[19] Nuclei are indicated in blue using Hoescht 33342 nuclear stain, and Mitochondria are indicated in yellow using Mitotracker fluorescent stain.

Supplemental File 1. Experiment description, QC data and ToxPI examples for iPSCs and HUVECs.

4.1.2. Cardiomyocyte-Specific Positive Controls

Isoproterenol

CasRN: 5984-95-2 Supplier: Molecular Devices (Assay Kit #: R8210)

Top Concentration: 50 µM (0.5% DMSO)

Isoproterenol is a cardiac medication used for slow heart rate. It has both inotropic (alters muscle contractility) and chronotropic (alters heart rate) effects on the heart. This positive control has been used before for these cells for its chronotropic effects.[1]

Propranolol

CasRN: 318-98-9 Supplier: Molecular Devices (Assay Kit #: R8210)

Top Concentration: 50 µM (0.5% DMSO)

Propranolol is cardiac medication used for the treatment of high blood pressure and irregular heart rate as well as some neurological disorders; although, its current use for hypertension is not recommended. Similar to isoproterenol, it has both ionotropic and chronotropic effects however decreases them. This agent was also used previously with these cells and included for it that reason.[1]

Cisapride

CasRN: 260779-88-2 Supplier: Sigma

Top Concentration: 50 µM (0.5% DMSO)

Cisapride was once a therapeutic for some gastrointestinal disorders but has been withdrawn due to side effects, in particular the development of cardiac arrhythmias. The arrhythmias are associated with long QT syndrome which is am alteration of the normal beat pattern. A similar phenotype was seen with iCell Cardiomyocytes in culture and those effects warranted its inclusion in this screen.[1]

Sotalol

CasRN: 959-24-0 Supplier: Molecular Devices (Assay Kit #: R8210)

Top Concentration: 50 µM (0.5% DMSO)

Similar to cisapride, sotalol can also cause prolongation of the QT interval and is used in some extreme cases of ventricular arrhythmias. In addition, previous studies utilized this chemical and saw the desired effects.[1]

Tetraoctylammonium Bromide (TAB)

CasRN: 14866-33-2 Supplier: Sigma

Top Concentration: 50 µM (0.5% DMSO)

Tetraoctylammonium bromide is a compound which consists of long aliphatic chains connected to an ammonium ion. TAB is commonly used in the surface coating of nanoparticles as the aliphatic chains impart specific characteristics onto the nanoparticle.[15] The long aliphatic chains have also been suggested to disrupt the cellular membrane which can serve as a reproducible and useful cytotoxic control. It was included here as a non-cellular specific cytotoxic control.

Supplemental File 1. Experiment description, QC data and ToxPI examples for iPSCs and HUVECs.

4.2. SCREEN SPECIFIC INFORMATION

Cell Manufacturer: Cellular Dynamics
Cell line Catalogue Number: R1007 (formerly CMC-100-010-001)
Cell Lot Number: 1297667
Date of Receipt of Cells at Texas A&M University: 12-18-2015
Date Screening was performed: 10-6-16 to 10-24-16
Instrumentation used to record high-content imaging phenotypes:

- ImageXpress® Microconfocal High Content Imaging System (Molecular Devices, Sunnyvale, CA)
- FLIPR®Tetra High-Throughput Cellular Screening System (Molecular Devices, Sunnyvale, CA)

4.3. CARDIOMYOCYTE ENDPOINTS FOR BIOACTIVITY PROFILING

4.3.1. Total Cells

4.3.1.1. Phenotype definition

Total Cells is a count of all cells present in the imaged field. The fluorescent stain Hoechst 33342 was used to bind to DNA in the nucleus and enable the detection and count of all nuclei in the imaged area. Since each cell contains a single nucleus, the number of nuclei reflects the total number of cells present. Treatments that are cytotoxic were expected to result in a reduced total cell count compared to media/vehicle treatments.

4.3.1.2. Quality Control – 90 Minutes

4.3.1.2.1. Vehicle and Positive Controls

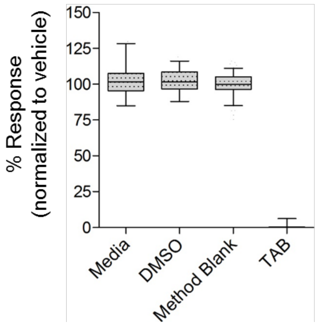

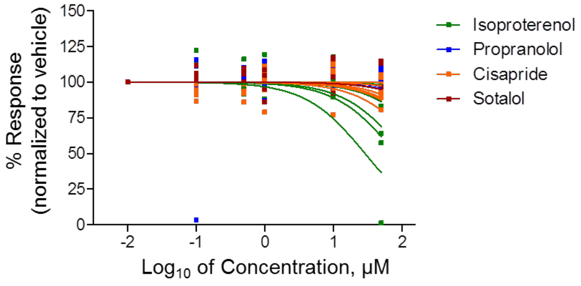

Supplemental File 1. Experiment description, QC data and ToxPI examples for iPSCs and HUVECs.

4.3.1.2.2. Intra- and Inter- Plate Replicability

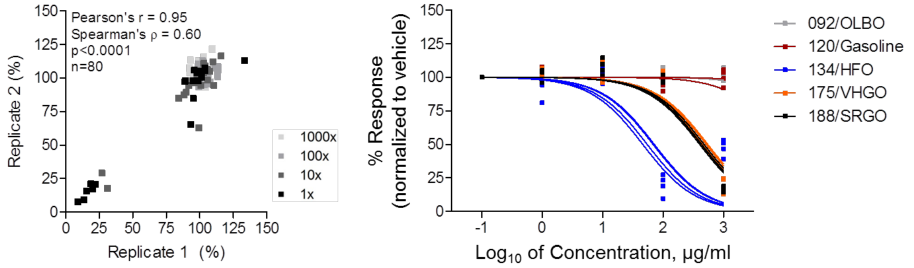

4.3.1.3. Quality Control – 24 hours

4.3.1.3.1. Vehicle and Positive Controls

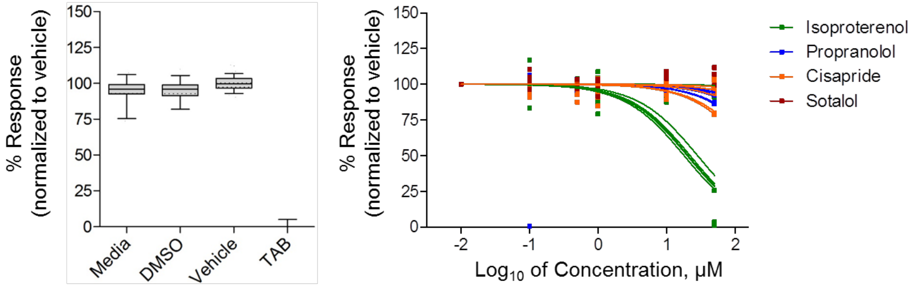

4.3.1.3.2. Intra- and Inter- Plate Replicability

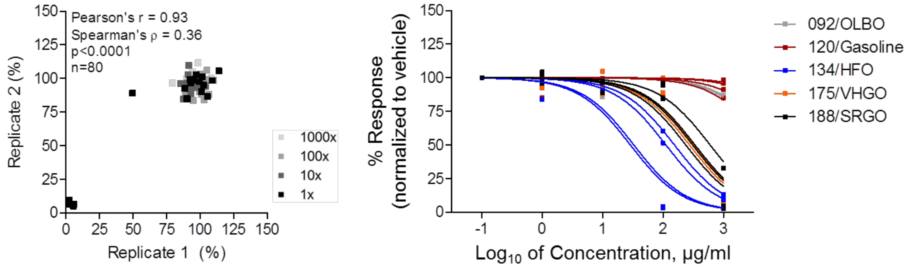

4.3.2. Positive Mitochondrial Staining

4.3.2.1. Phenotype definition

Positive mitochondrial staining indicates the number of cells in an imaged field with intact mitochondria. Mitochondria are essential organelles that provide energy to drive cellular processes. Two key features of healthy, functional mitochondria are ATP synthesis and the generation of membrane potential. MitoTracker Orange was used to stain intact mitochondria. The MitoTracker dye is only fluorescent inside a live cell, and it is only sequestered within healthy mitochondria possessing a membrane potential. Cytotoxic treatments or treatments that interfere with normal mitochondrial function were expected to have a lower Positive Mitochondrial Staining score than healthy cells.

Supplemental File 1. Experiment description, QC data and ToxPI examples for iPSCs and HUVECs.

4.3.2.2. Quality Control – 90 Minutes

4.3.2.2.1. Vehicle and Positive Controls

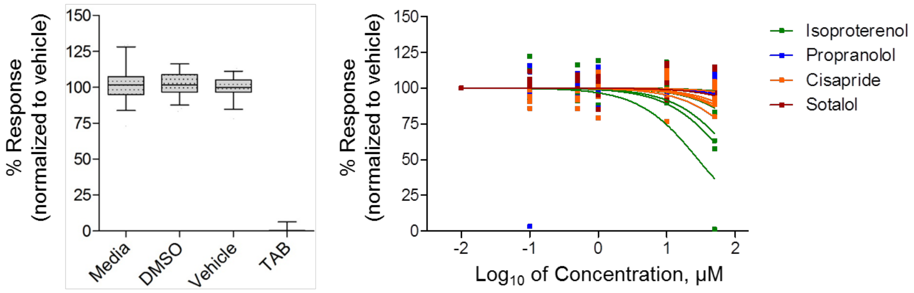

4.3.2.2.2. Intra- and Inter- Plate Replicability

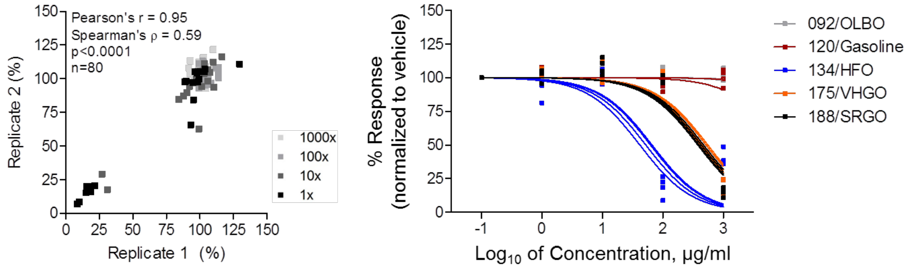

4.3.2.3. Quality Control – 24 hours

4.3.2.3.1. Vehicle and Positive Controls

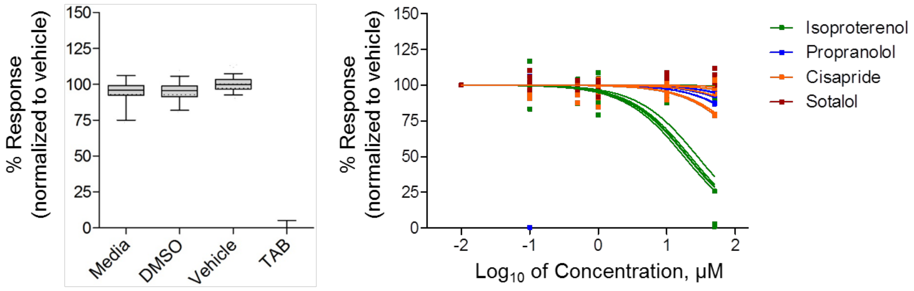

4.3.2.3.2. Intra- and Inter- Plate Replicability

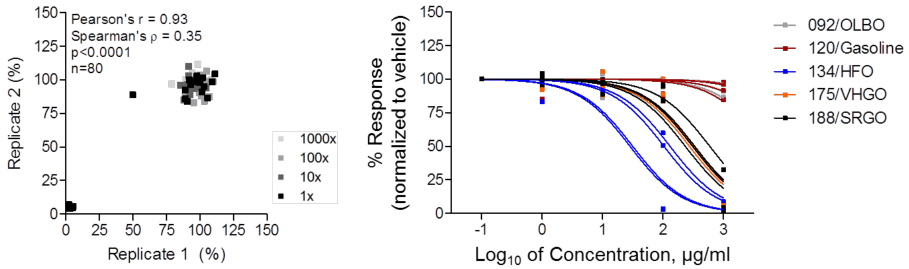

Supplemental File 1. Experiment description, QC data and ToxPI examples for iPSCs and HUVECs.

4.3.3. Beat per Minute

4.3.3.1. Phenotype definition

The cardiomyocyte peak frequency measured in beats per minute is a sensitive phenotype characterizing cardiophysiologic performance of a synchronously beating cardiomyocyte in vitro culture. Changes in the BPM are characteristic for certain types of cardiotoxic chemicals, e.g. positive and negative inotropic agents or potassium channel inhibitors.

4.3.3.2. Quality Control – 90 Minutes

4.3.3.2.1. Vehicle and Positive Controls

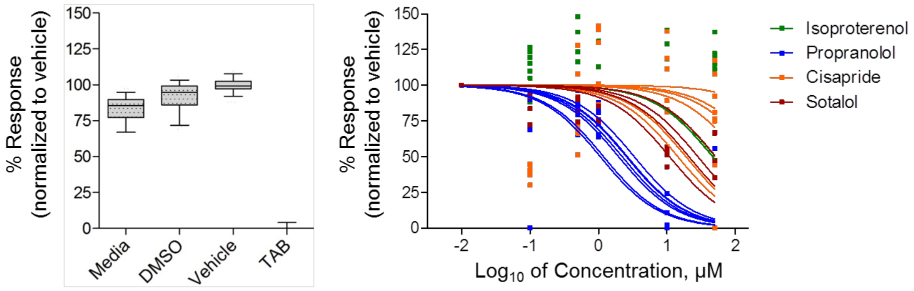

4.3.3.2.2. Intra- and Inter- Plate Replicability

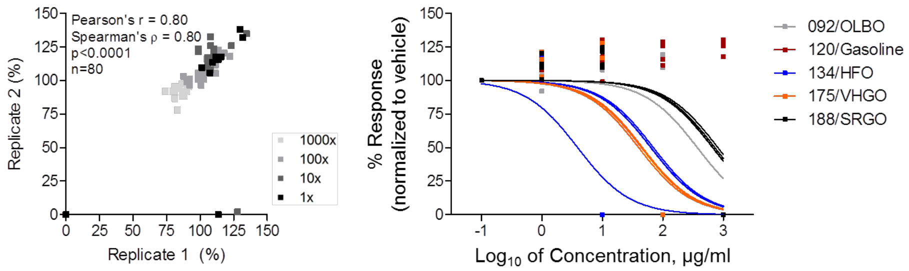

4.3.3.3. Quality Control – 24 hours

4.3.3.3.1. Vehicle and Positive Controls

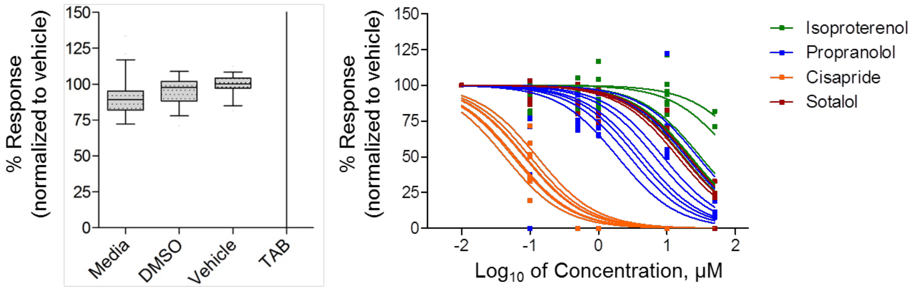

Supplemental File 1. Experiment description, QC data and ToxPI examples for iPSCs and HUVECs.

4.3.3.3.2. Intra- and Inter- Plate Replicability

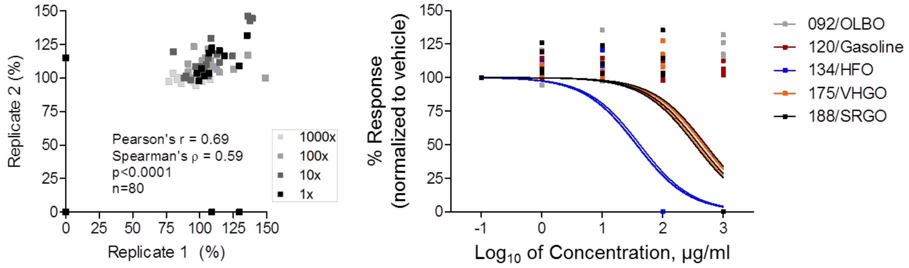

4.3.4. Peak Amplitude Average

4.3.4.1. Phenotype definition

The average Peak Amplitude for a cardiomyocyte culture is determined for the same interval used to derive the BPM and serves as a secondary measure to inotropic effects, i.e. is usually inversely correlated with the average peak frequency.

4.3.4.2. Quality Control – 90 Minutes

4.3.4.2.1. Vehicle and Positive Controls

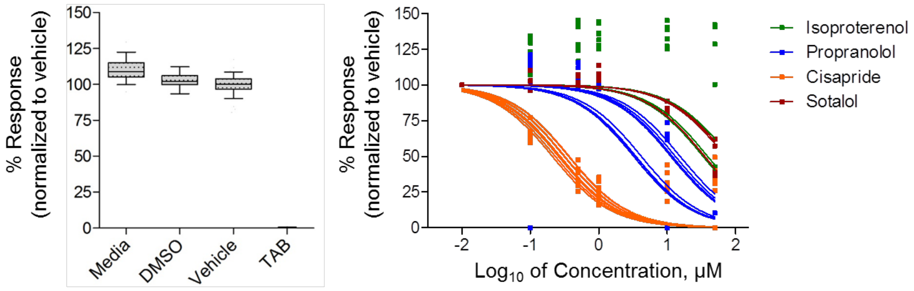

4.3.4.2.2. Intra- and Inter- Plate Replicability

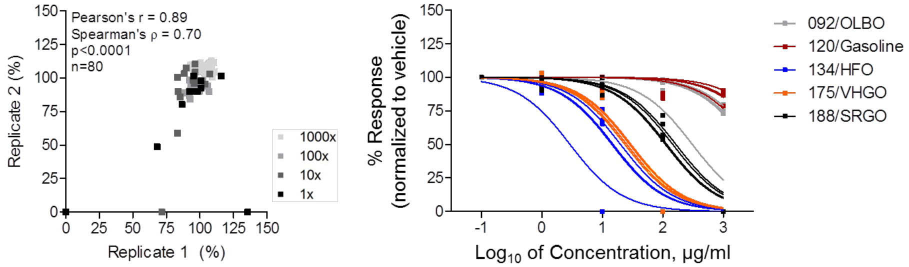

Supplemental File 1. Experiment description, QC data and ToxPI examples for iPSCs and HUVECs.

4.3.4.3. Quality Control – 24 hours

4.3.4.3.1. Vehicle and Positive Controls

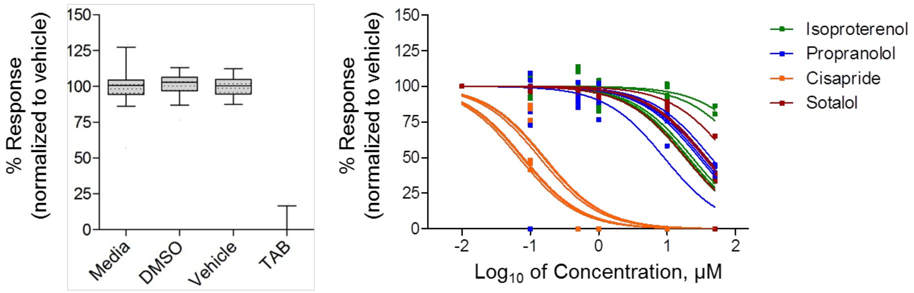

4.3.4.3.2. Intra- and Inter- Plate Replicability

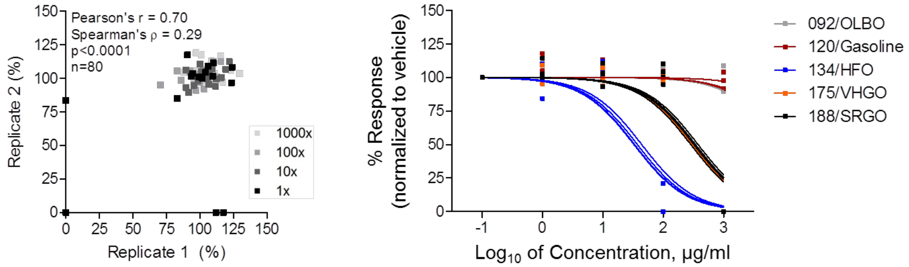

4.3.5. Peak Amplitude Coefficient of Variation

4.3.5.1. Phenotype definition

A major concern for cardiotoxic hazards are cardiac arrhythmias that can be characterized as "torsades de pointes", a clinically significant and potentially life-threatening diffuse irregularity in the heart beat. The coefficient of variation (CV) associated with the peak amplitudes measured in untreated and exposed cardiomyocytes is one characteristic phenotype for evaluating cardiomyocyte beating irregularities *in vitro*, i.e. the higher the amplitude CV value, the more irregular the cells are beating.

4.3.5.2. Quality Control – 90 Minutes

4.3.5.2.1. Vehicle and Positive Controls

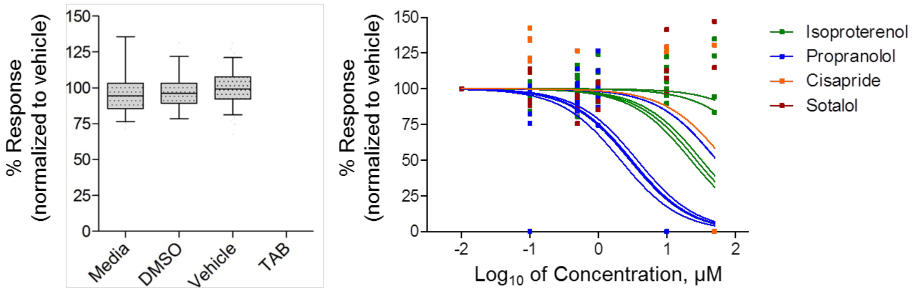

4.3.5.2.2. Intra- and Inter- Plate Replicability

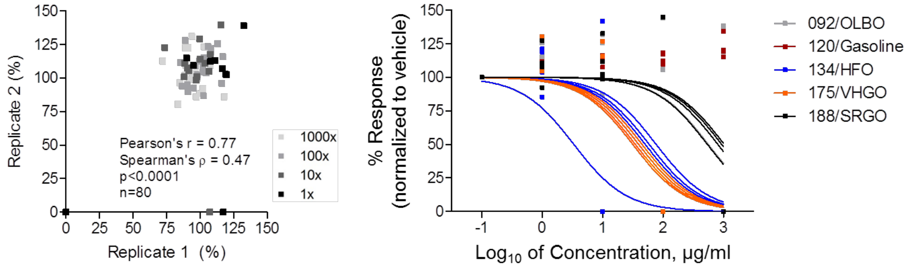

4.3.5.3. Quality Control – 24 hours

4.3.5.3.1. Vehicle and Positive Controls

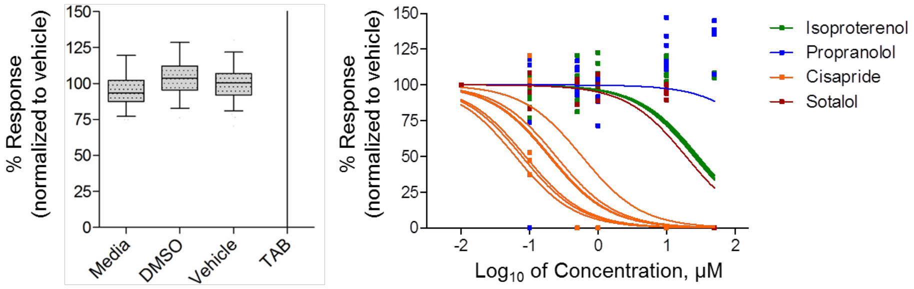

4.3.5.3.2. Intra- and Inter- Plate Replicability

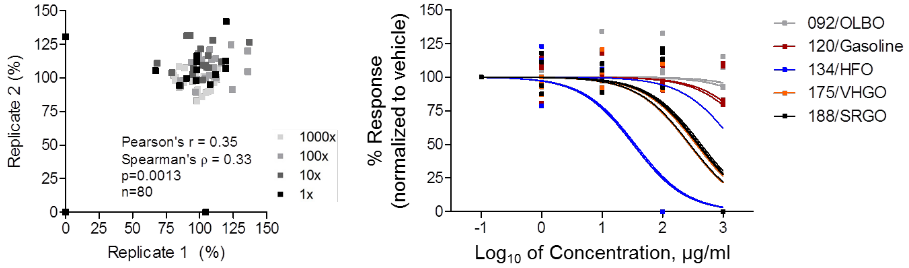

4.3.6. Peak Spacing Coefficient of Variation

4.3.6.1. Phenotype definition

A major concern for cardiotoxic hazards are cardiac arrhythmias that can be characterized as “torsades de pointes”, a clinically significant and potentially life-threatening diffuse irregularity in the heart beat. The coefficient of variation (CV) associated with the peak spacing, i.e. distance or gap measured between cardiomyocyte depolarizations, is a second characteristic phenotype for evaluating cardiomyocyte beating irregularities *in vitro*, i.e. the higher the spacing CV value, the more irregular the cells are beating.

Supplemental File 1. Experiment description, QC data and ToxPI examples for iPSCs and HUVECs.

4.3.6.2. Quality Control – 90 Minutes

4.3.6.2.1. Vehicle and Positive Controls

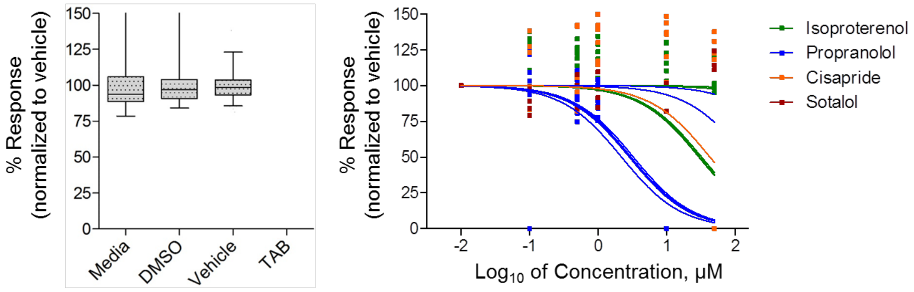

4.3.6.2.2. Intra- and Inter- Plate Replicability

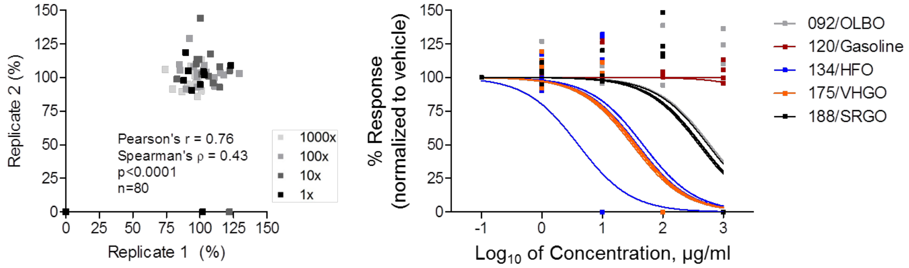

4.3.6.3. Quality Control – 24 hours

4.3.6.3.1. Vehicle and Positive Controls

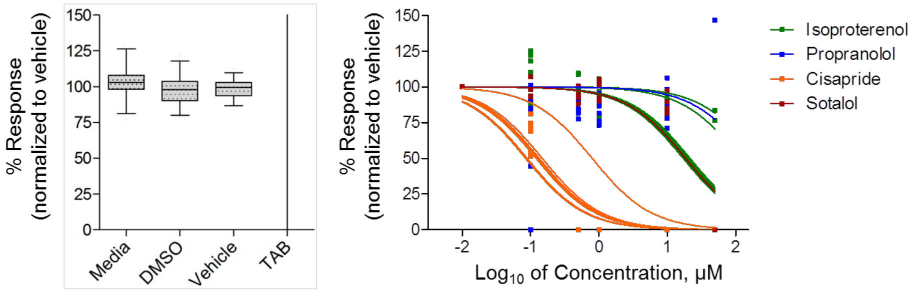

4.3.6.3.2. Intra- and Inter- Plate Replicability

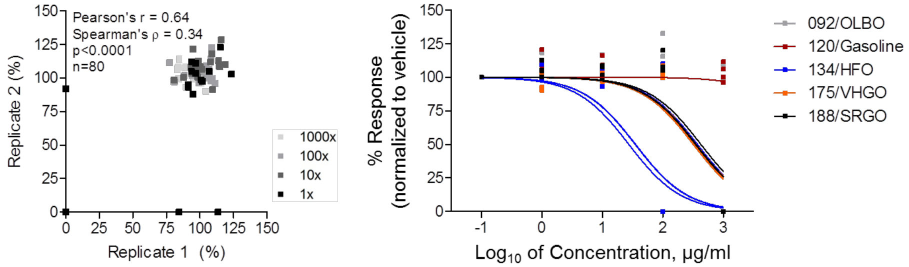

4.3.7. Peak Decay Over Rise Time

4.3.7.1. Phenotype definition

Potassium channel inhibitors, including hERG channels inhibitors such as cisapride and sotalol, are associated with a characteristic cardiophysiologic phenotype referred to as QTc interval prolongation, which is a chemically-induced delayed repolarization that is reflected in the formation of peak shoulder, or notch. This *in vivo* phenotype can be reliably reproduced in cardiomyocyte cell culture. The peak decay over rise time ratio is a quantitative descriptor of this phenotype and QT prolongation is reflected by an increase in the ratio value.

4.3.7.2. Quality Control – 90 Minutes

4.3.7.2.1. Vehicle and Positive Controls

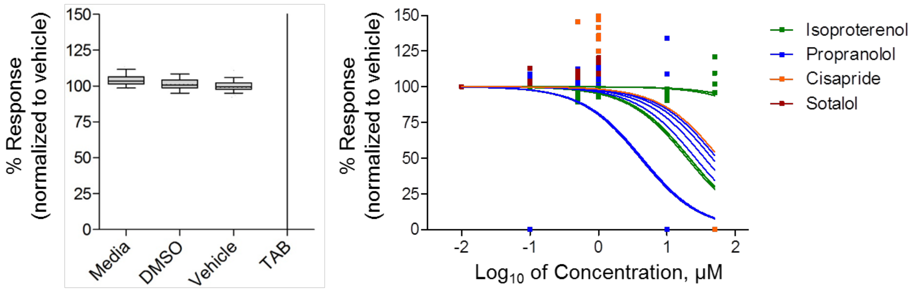

4.3.7.2.2. Intra- and Inter- Plate Replicability

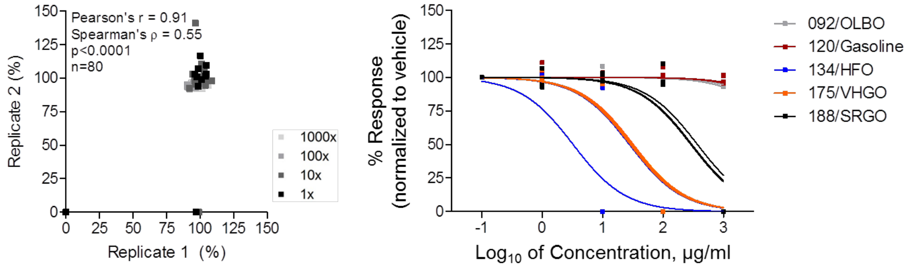

4.3.7.3. Quality Control – 24 hours

4.3.7.3.1. Vehicle and Positive Controls

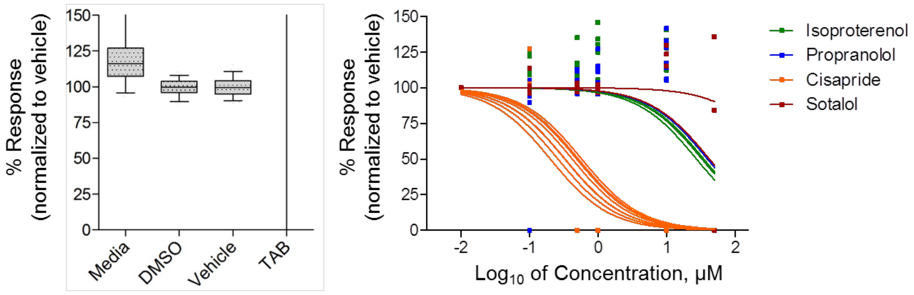

Supplemental File 1. Experiment description, QC data and ToxPI examples for iPSCs and HUVECs.

4.3.7.3.2. Intra- and Inter- Plate Replicability

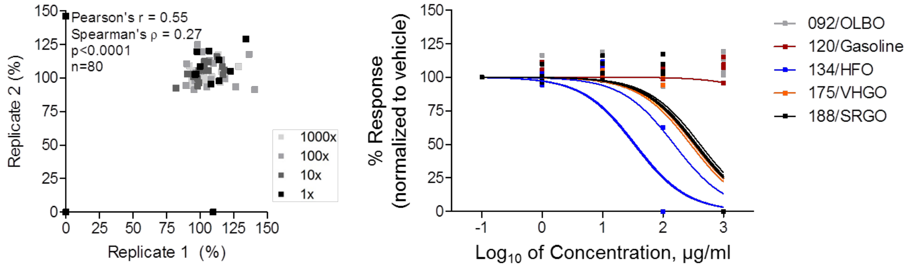

4.4. TOXPI AGGREGATION AND SUMMARY

4.4.1. ToxPi Key and ToxPi's for Select Petroleum Substances

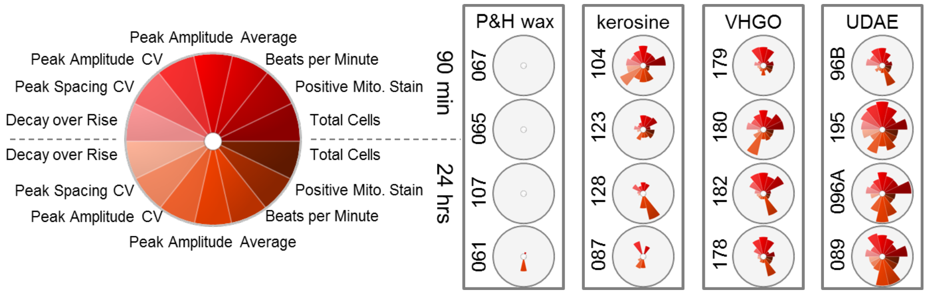

4.4.2. ToxPi Summary Graphs

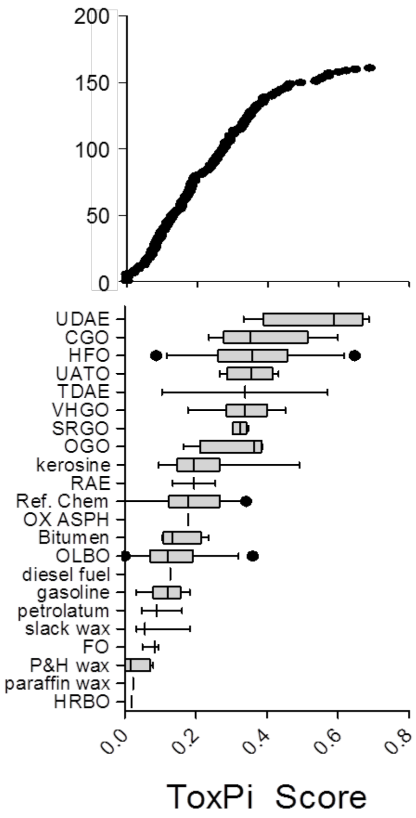

5. NEURONS

A large percentage of the cells that make up the nervous system are neurons. The main function of neuronal cells is to transmit and receive information. While neurons share an overall similar morphology and general function, they are varied in their response to signals, e.g. stimulatory or inhibitory. Given the complex nature of neuronal functions, subtle perturbations in cellular physiology can result in substantial alterations overall health. Recent publications have incorporated neuronal cell types to assess environmental chemicals in an *in vitro* high-throughput screening approach.[20,21] These assays assess general cytotoxicity in addition to the inhibition of neurite development caused by chemical treatment.

5.1. iCELL NEURONS

Traditional *in vitro* systems for neuronal cultures focuses on primary cultures from rodents. Such systems can encounter issues with using primary cultures as well as questions regarding the relevance of this model in human biology. New models developed by Cellular Dynamics address both of these issues with their iCell Neuron product. These iPSC-derived neurons consist of >95% inhibitory or GABAergic neurons with expression and function of neuronal specific pathways. In addition, the ability of these neurons to rapidly develop networks provides a useful model for assessing any neuronal bioactivity in a high-throughput screen.

5.1.1. Basal and Altered Morphology

Neurons are unique in that they have a very distinct morphology that is not seen in other types of cells. This morphology is distinct to facilitate the main function of neurons, cell-cell communication. The main part of the neuronal cell is called the cell body and includes the traditional subcellular organelles. Extending out from the cell body are thin wire-like projections called neurites. It is through these neurites that the signalling and communication between neurons can take place. Because of this unique morphology, neurons can provide additional information about the bioactivity of chemicals. By looking at how neurites change, whether in number, size, or branching, provides a sensitive endpoint for assess neuronal bioactivity.

Basal Morphology:[22] 100 µM Brefeldin A:[28]

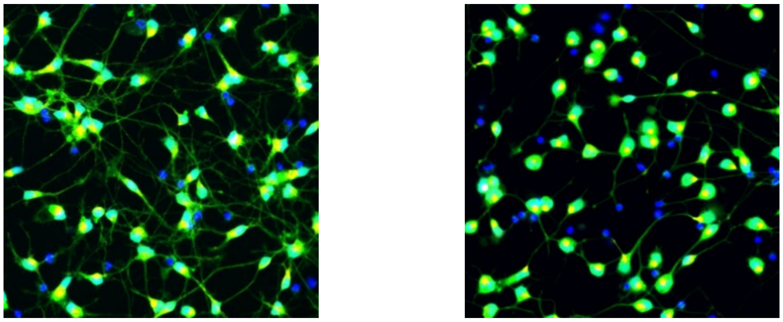

[20] Sirenko O, Hesley J, Rusyn I, Cromwell EF. "High-content high-throughput assays for characterizing the viability and morphology of human iPSC-derived neuronal cultures." *Assay Drug Dev Technol.* 2014, 12(9-10), 536-547.

[21] Ryan KR, Sirenko O, Parham F, Hsieh JH, Cromwell EF, Tice RR, Behl M. "Neurite outgrowth in human induced pluripotent stem cell-derived neurons as a high-throughput screen for developmental neurotoxicity of neurotoxicity." *Neurotoxicology.* 2016, 53, 271-281.

[22] Nuclei are indicated in blue using Hoescht 33342 nuclear stain, cytoplasm is indicated in green using Calcein AM stain, and Mitochondria are indicated in yellow using Mitotracker fluorescent stain.

5.1.2. Neuron-Specific Positive Controls

Mitomycin C

CasRN: 50-07-7 Supplier: Tocris

Top Concentration: 100 µM (0.25% DMSO)

Mitomyocin C is a chemotherapeutic agent used for a wide variety of cancers via it's alkylating ability. Various neuropathies have been associated with Mitomycin C treatments; as well as a previous study has reported neurotoxic effects on iCell Neurons.[26] Its inclusion in this screen was for the previous recorded positive bioactivity in iCell Neurons.

Rotenone

CasRN: 83-79-4 Supplier: MP Biomedicals

Top Concentration: 50 µM (0.25% DMSO)

Rotenone is a commonly used insecticide and pesticide with some associated human toxicity. This compound exerts its toxic effects by inhibiting mitochondrial respiration leading to oxidative stress and overall decreases in cellular health.[26] Rotenone was included as a positive control for mitochondrial dysfunction and for general cytotoxicity.

Carbonyl cyanide m-chlorophenyl hydrazine (CCCP)

CasRN: 555-60-2 Supplier: Tocris

Top Concentration: 130 µM (0.25% DMSO)

Carbornyl cyanide m-chlorophenyl hydrazide (CCCP) is a known toxic agent which directly acts on the mitochondria through inhibition of oxidative phosphorylation. This is achieved through multiple mechanisms, mainly the uncoupling of the proton gradient.[26] CCCP was included in this screen for its effects on the mitochondria and general cytotoxic effects.

Retinoic Acid

CasRN: 302-79-4 Supplier: Tocris

Top Concentration: 250 µM (0.25% DMSO)

Retinoic Acid is a metabolite of vitamin A and has been shown to induce developmental neurological issues. In addition, previous researchers have shown toxicity in iCell Neurons.[26] Its inclusion in this screen was for the previous recorded positive bioactivity in iCell Neurons.

Brefeldin A

CasRN: 20350-15-6 Supplier: Tocris

Top Concentration: 10 µM (0.25% DMSO)

Brefeldin A is a fungal antibiotic that also disrupts protein transport. Previous research has shown the Brefeldin A treatment can decrease the outgrowth of neurites on neuronal populations.[23] It was included as a positive control for inhibiting neurite outgrowth.

[23] Harrill JA, Freudenrich TM, Macheck DW, Stice SL, Mundy WR. "Quantitative assessment of neurite outgrowth in human embryonic stem cell-derived hN2TM cells using automated high-content image analysis." *Neurotoxicology*, 2010, 31, 277-290.

Tetraoctylammonium Bromide (TAB)
CasRN: 14866-33-2   Supplier: Sigma
Top Concentration: 50 µM (0.25% DMSO)

Tetraoctylammonium bromide is a compound which consists of long aliphatic chains connected to an ammonium ion. TAB is commonly used in the surface coating of nanoparticles as the aliphatic chains impart specific characteristics onto the nanoparticle.[15] The long aliphatic chains have also been suggested to disrupt the cellular membrane which can serve as a reproducible and useful cytotoxic control. It was included here as a non-cellular specific cytotoxic control.

5.2. SCREEN SPECIFIC INFORMATION

<u>Cell Manufacturer:</u> Cellular Dynamics
<u>Cell line Catalogue Number:</u> R1013 (formerly NRC-100-010-001)
<u>Cell Lot Number:</u> 1227535 & 1221529
<u>Date of Receipt of Cells at Texas A&M University:</u> 6-20-17
<u>Date Screening was performed:</u> 6-30-17 to 7-5-17
<u>Instrumentation used to record high-content imaging phenotypes:</u>

- ImageXpress® Microconfocal High Content Imaging System (Molecular Devices, Sunnyvale, CA)

5.3. NEURON ENDPOINTS FOR BIOACTIVITY PROFILING

5.3.1. Total Cells

5.3.1.1. Phenotype definition

Total Cells is a count of all cells present in the imaged field. The fluorescent stain Hoechst 33342 was used to bind to DNA in the nucleus and enable the detection and count of all nuclei in the imaged area. Since each cell contains a single nucleus, the number of nuclei reflects the total number of cells present. Treatments that are cytotoxic were expected to result in a reduced total cell count compared to media/vehicle treatments.

5.3.1.2. Quality Control

5.3.1.2.1. Vehicle and Positive Controls

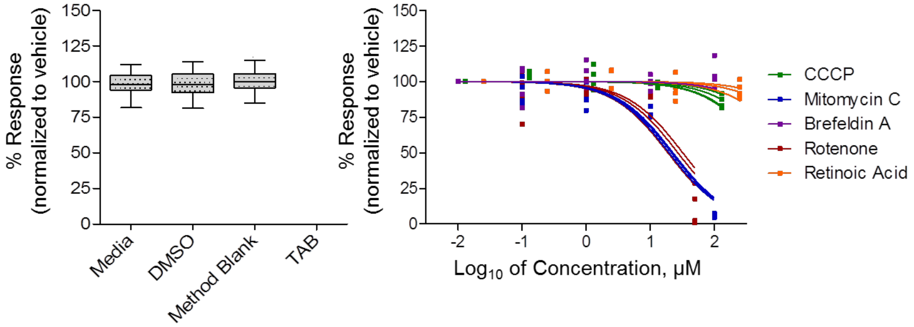

5.3.1.2.2. Intra- and Inter- Plate Replicability

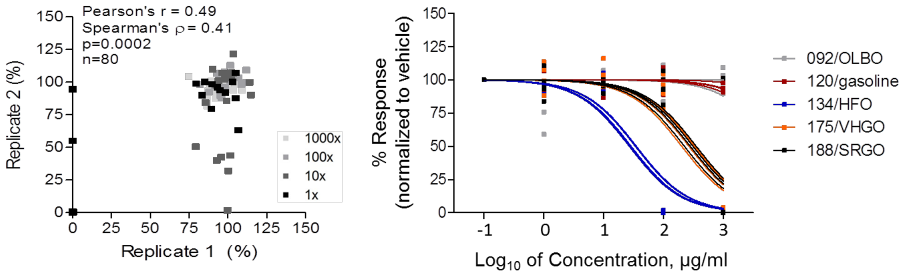

5.3.2. Total Processes

5.3.2.1. Phenotype definition

Total Processes refers to the number of outgrowths that are connected to cell bodies within the imaged field. Neurons were stained with the cytoplasmic stain Calcein AM, allowing both the outgrowths and the cell bodies to be visualized. Within the Image Xpress software, parameters that define an outgrowth and a cell body were specified so that the total number of outgrowths could be counted. Given the importance of neurites in cell communication, chemical treatments that reduce the number of outgrowths are likely to interfere with normal neuronal function.

5.3.2.2. Quality Control

5.3.2.2.1. Vehicle and Positive Controls

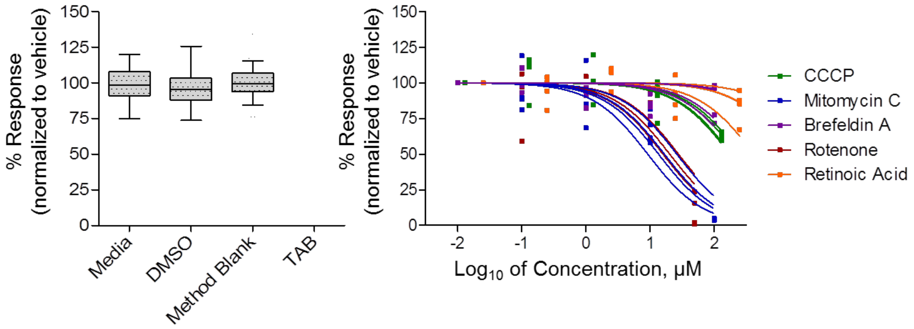

5.3.2.2.2. Intra- and Inter- Plate Replicability

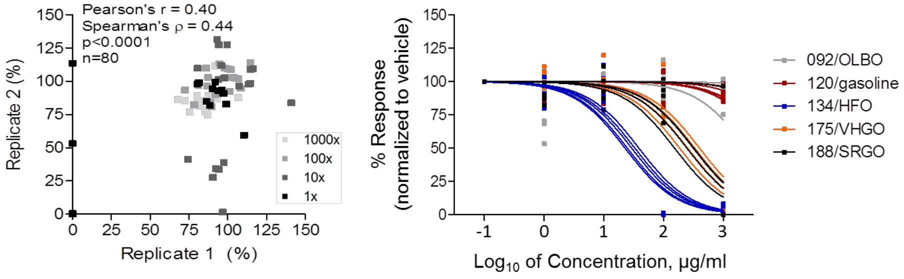

5.3.3. Mean Cell Body Area

5.3.3.1. Phenotype definition

Mean Cell Body Area is defined as the average area of cell bodies across the image being analysed. Cell bodies were stained with Calcein AM, and the stained area was imaged and measured by ImageXpress®. Changes in the cell body area may reflect general perturbations in the neurons by mechanisms that don't necessarily affect neurite formation.

5.3.3.2. Quality Control

5.3.3.2.1. Vehicle and Positive Controls

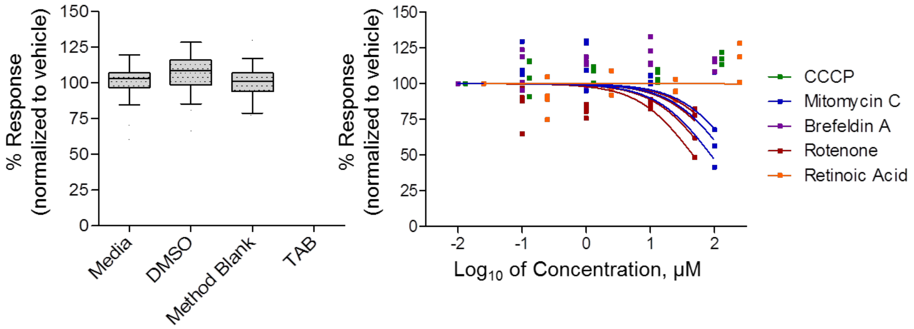

5.3.3.2.2. Intra- and Inter- Plate Replicability

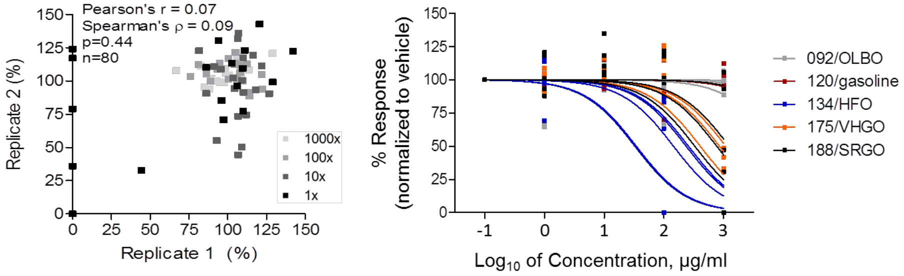

5.3.4. Cells with Significant Growth

5.3.4.1. Phenotype definition

Cells with Significant Growth is simply a count of cells having outgrowth greater than a specified threshold. Here, outgrowths greater than 25 um in lengths were considered to be "significant growth". This threshold is in line with previous publications[26]. As before, neuronal cell bodies and outgrowths were stained with Calcein AM, imaged and counted in ImageXpress®. A population of healthy neurons were expected to include more neurons with significant growth than neurons treated with a bioactive chemical that negatively affects neurite outgrowth.

Supplemental File 1. Experiment description, QC data and ToxPI examples for iPSCs and HUVECs.

5.3.4.2. Quality Control

5.3.4.2.1. Vehicle and Positive Controls

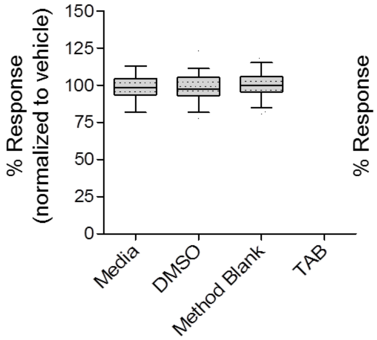

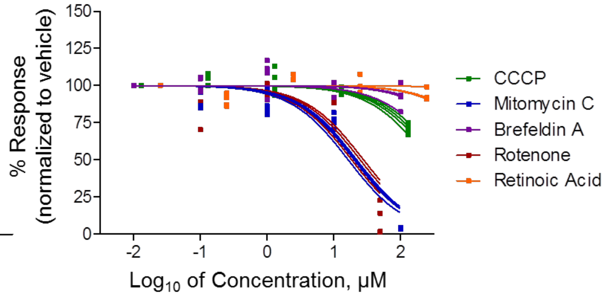

5.3.4.2.2. Intra- and Inter- Plate Replicability

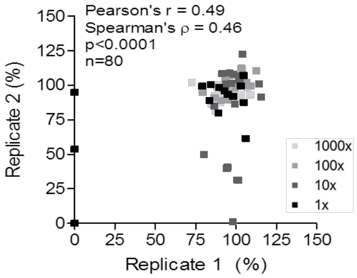

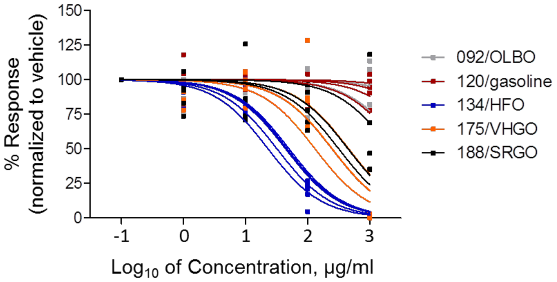

Supplemental File 1. Experiment description, QC data and ToxPI examples for iPSCs and HUVECs.

5.4. TOXPI AGGREGATION AND SUMMARY

5.4.1. ToxPi Key and ToxPi's for Select Petroleum Substances

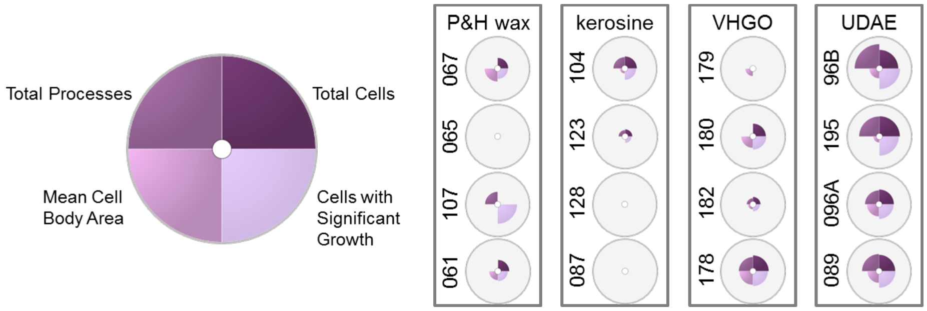

5.4.2. ToxPi Summary Graphs

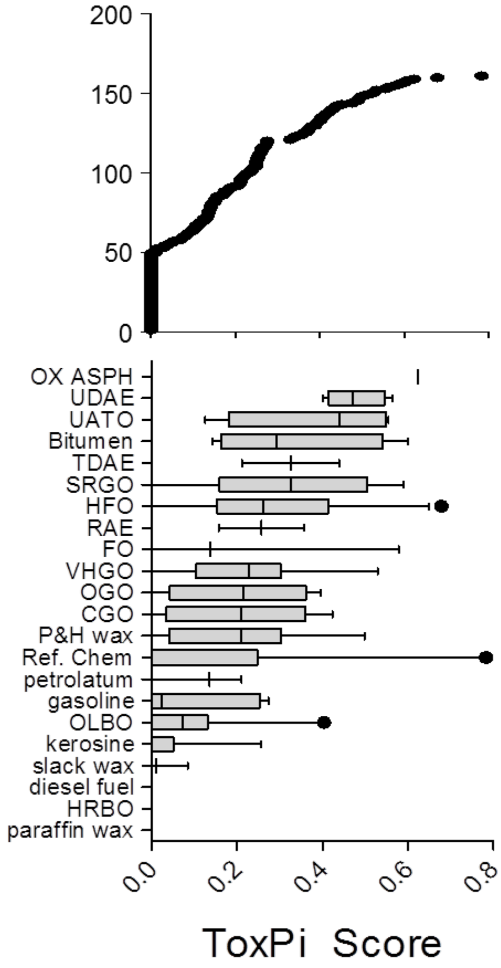

6. MACROPHAGES

While a lot of organ systems have been covered by the various other cell types used in this investigation, the immune system is lacking. The immune system is comprised of a wide range of different cell types. One common cell type is the macrophage. This cell serves as a scavenger which engulfs foreign and endogenous objects as well as secretes cytokines all to facility the innate and adaptive immune response. Given its highly mobile and flexible nature, the morphology of a macrophage can be varied from a simple round shape to exhibiting arm-link projections used to engulf objects.

6.1. iCELL MACROPHAGES

One of the only immune cells offered by Cellular Dynamics is the iCell Macrophage. This shares the same benefits of other iPSC-derived cells, that of genetically reproducible and unlimited supply. Other *in vitro* models of macrophages utilize primary human monocytes which need to be further differentiated into macrophages. iCell Macrophages avoid this additional step while also providing a pure population of CD68+ cells which are reportedly capable of cytokine secretion and phagocytosis of various particles. The development of various other endpoints for this cell type were pursued; however, they failed to reach an acceptable level of reproducibility.

6.1.1. Macrophage-Specific Positive Controls

Cytochalasin D
CasRN: 22144-77-0 Supplier: Cayman Chemical
Top Concentration: 100 µM (0.5% DMSO)

Cytochalasin D is a mycotoxin that potently inhibits actin polymerization. This property has been used previously to alter macrophage function by inhibiting phagocytosis.[24] Here, cytochalasin D's more general cytotoxic effects on macrophages were used.

Phorbol 12-myristate 13-acetate (PMA)
CasRN: 16561-29-8 Supplier: Cayman Chemical
Top Concentration: 0.1 µM (0.5% DMSO)

Phorbol 12-myristate 13-acetate (PMA) is a tumor promoting agent that specifically activates the protein kinase C. Previous studies have shown that PMA alters macrophage cytokine secretion as well as induces apoptosis at moderate concentrations.[25] These properties warranted its inclusion in the screen.

SB203582
CasRN: 152121-47-6 Supplier: Cayman Chemical
Top Concentration: 1000 µM (0.5% DMSO)

[24] Kapellos TS, Taylor L, Lee H, Cowley SA, James WS, Iqbal AJ, Greaves DR. "A novel real time imaging platform to quantify macrophage phagocytosis." *Biochem Pharmaol*. 2016, 116, 107-119.

[25] Smith ME, van der Maesen K, Somera FP, Sobel RA. "Effects of phorbol myristate acetate (PMA) on functions of macrophages and microglia in vitro." *Neurochem Res.* 1998, 23(3), 427-434.

SB203582 is a specific inhibitor of the P38 and the downstream MAP kinase pathway. This agent is used to inhibit macrophage activation and was included for that purpose.

Lipopolysaccharide (LPS)
CasRN: NA Supplier: Sigma
Top Concentration: 500 ng/ml (0.5% DMSO)

Lipopolysaccharide (LPS) is a product of gram-negative bacteria that is found in their outer membrane. LPS is known to activate the immune system at large but also specifically macrophages through specific receptors. LPS's ability to alter macrophage functions has been extensively shown and for this reason, LPS was included in this screen.

Tetraoctylammonium Bromide (TAB)
CasRN: 14866-33-2 Supplier: Sigma
Top Concentration: 50 µM (0.5% DMSO)

Tetraoctylammonium bromide is a compound which consists of long aliphatic chains connected to an ammonium ion. TAB is commonly used in the surface coating of nanoparticles as the aliphatic chains impart specific characteristics onto the nanoparticle.[15] The long aliphatic chains have also been suggested to disrupt the cellular membrane which can serve as a reproducible and useful cytotoxic control. It was included here as a non-cellular specific cytotoxic control.

6.2. SCREEN SPECIFIC INFORMATION

Cell Manufacturer: Cellular Dynamics
Cell line Catalogue Number: R1099 (formerly MAC-301-010-001-PT)
Cell Lot Number: 11415 & 11496
Date of Receipt of Cells at Texas A&M University: 6-20-17
Date Screening was performed: 7-13-17 to 7-17-17
Instrumentation used to record the phenotype:

- Flipr Tetra High-Throughput Cellular Screening System (Molecular Devices, Sunnyvale, CA)

6.3. MACROPHAGE ENDPOINT FOR BIOACTIVITY PROFILING

6.3.1. ATP Content

6.3.1.1. Phenotype Definition

ATP Content is the total amount of ATP present in all cells of a given treatment group. ATP is the energy currency of a cell and an indicator of cell metabolic activity. Since only live cells are metabolically active, ATP Content is an indicator of the total number of live cells. ATP content of cells was measured indirectly using the CellTiter-Glo®

assay kit and the FLIPR® Tetra Screening System for detection. The CellTiter-Glo® reagent produces a luminescent signal that is proportional to the total amount of ATP present in a treatment group. Cells treated with cytotoxic chemicals were expected to produce little/no ATP signal.

6.3.1.2. Quality Control

6.3.1.2.1. Vehicle and Positive Controls

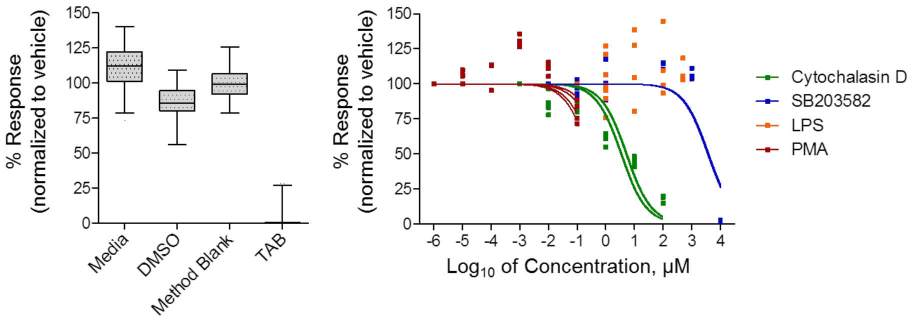

6.3.1.2.2. Intra- and Inter- Plate Replicability

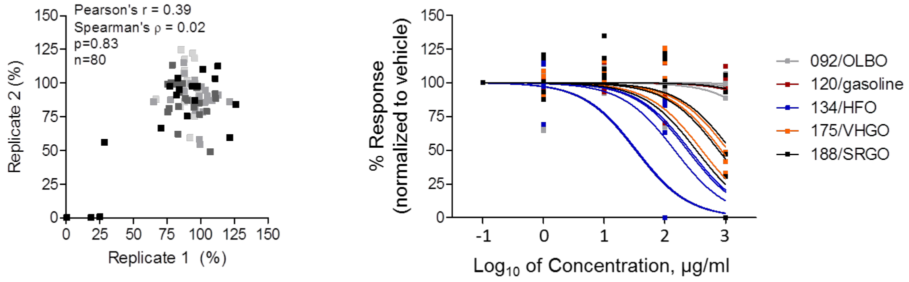

Supplement: Supplemental File 1 [file NIHMS1670764-supplement-Supplemental_File_1.pdf]
